# Multi-Variant Accuracy Evaluation of UAV Imaging Surveys: A Case Study on Investment Area

**DOI:** 10.3390/s19235229

**Published:** 2019-11-28

**Authors:** Grzegorz Gabara, Piotr Sawicki

**Affiliations:** Institute of Geodesy, University of Warmia and Mazury in Olsztyn, 10-719 Olsztyn, Poland

**Keywords:** UAV imagery, bundle block adjustment, digital surface model, orthomosaic, data collection, accuracy, technical guidelines

## Abstract

The main focus of the presented study is a multi-variant accuracy assessment of a photogrammetric 2D and 3D data collection, whose accuracy meets the appropriate technical requirements, based on the block of 858 digital images (4.6 cm ground sample distance) acquired by Trimble^®^ UX5 unmanned aircraft system equipped with Sony NEX-5T compact system camera. All 1418 well-defined ground control and check points were a posteriori measured applying Global Navigation Satellite Systems (GNSS) using the real-time network method. High accuracy of photogrammetric products was obtained by the computations performed according to the proposed methodology, which assumes multi-variant images processing and extended error analysis. The detection of blurred images was preprocessed applying Laplacian operator and Fourier transform implemented in Python using the Open Source Computer Vision library. The data collection was performed in Pix4Dmapper suite supported by additional software: in the bundle block adjustment (results verified using RealityCapure and PhotoScan applications), on the digital surface model (CloudCompare), and georeferenced orthomosaic in GeoTIFF format (AutoCAD Civil 3D). The study proved the high accuracy and significant statistical reliability of unmanned aerial vehicle (UAV) imaging 2D and 3D surveys. The accuracy fulfills Polish and US technical requirements of planimetric and vertical accuracy (root mean square error less than or equal to 0.10 m and 0.05 m).

## 1. Introduction

Due to significant advances in the research field of digital photogrammetry, computer vision, and unmanned aerial vehicles/unmanned aerial systems/remotely piloted aircraft systems (UAVs/UASs/RPASs) [1,2] construction, there has been an important change in geoinformation acquisition and the workflow of photogrammetric data collection [3]. This has been achieved by essential developments in the UASs components [4], in sensors production [5], and in the image-based surface reconstruction, e.g., structure-from-motion (SfM) [6,7], multi-view stereo (MVS) pipeline [8] and dense image-matching techniques [9]. 

The commercial off-the-shelf (COTS) software packages, focusing on UAV imagery applications in geomatics, are based on a large number of variously oriented digital images and work in the composed and often an autonomous processing chain [10,11,12]. They allow an automatic computation of spatial orientation of photos and the parameters of camera interior orientation with self-calibration [13] using the bundle-block adjustment (BBA) method, generation of dense point clouds [11], a 3D mesh model and, finally, the digital surface model (DSM) [10,14] and orthomosaic [15].

The spectrum of problems and research tasks in the new area of photogrammetry, the so-called “UAV Photogrammetry”, is very wide [5,9,16,17,18,19,20,21]. The research and development mainly concerns, among other areas: small UAS [22], low-cost hardware solution [23], onboard sensors integration [24], sensors calibration [13,25,26], images orientation and direct georeferencing [27], data fusion obtained from optical sensors in various spectral ranges [24] and LiDAR (Light Detection and Ranging) [28], automation of flight planning [29], automation of digital images processing [30], testing and comparison of software dedicated to UASs [10,11]. An important research area is also testing the accuracy potential [3,31,32,33,34] of new measurement technologies based on UAV imagery, searching for new areas of its practical implementation [35,36,37], civil law regulations regarding the use of RPASs [18,38].

However, in our opinion, the operational use of UAV imagery acquired by means of single-lens reflex or compact system camera instead of medium or large format aerial digital cameras for data collection in order to undertake large-scale basic, thematic [39,40,41,42] and inventory mapping is one of the fundamental issues for the application of UAVs in geomatics and in particular in geodesy. For example, in Germany, the USA and also in Poland, there are no detailed technical guidelines regarding the use of UAV imagery in geodetic surveys. The only criterion for taking photogrammetric data into a geodetic database is the accuracy requirements [43,44,45,46] for the final products. 

The use of RPASs in national mapping was described previously in state-of-art by the EuroSDR organization [38], in orthophoto quality assessment performed by Mesas-Carrascosa et al. [15] and Martínez-Carricondo et al. [47], in photogrammetric mapping evaluation by Agüera-Vega et al. [48] and in an investigation of the optimal number of ground control points (GCPs) for DSM by Tonkin and Midgley [49]. This current situation consequently limits the use of RPAS in geodesy. Researchers reported before that the regulation is the most limiting factor that hinders the thorough application of RPASs [18,38,50]. 

According to the authors’ knowledge, the complex accuracy assessment of the computing pipeline of particular photogrammetric products (bundle-block adjustment with camera self-calibration, dense point cloud, digital surface model and orthomosaic) which we present in this paper, do not exist at present. Current studies concerning the accuracy assessment of UAV photogrammetric products are realized in incomparable technical conditions of projects and with the use of various statistical parameters of results assessment [39,40] relating to the particular stages of the processing [36,41,42,51]. The most commonly used metrical information of accuracy were root mean square errors RMSE (XYZ) on ground control and check points, which characterized bundle block adjustment results. The additional parameters of sensor interior orientation estimated in the self-calibration procedure were described by standard deviations. In addition, the quality and accuracy of DSMs and orthomosaics were analyzed using RMS (Z) and RMS (XY) deviations, respectively.

The issues mentioned above were the inspiration for defining the following main aims of this study:

- Definition of the computation pipeline methodology for measurement accuracy assessment under the real conditions of a posteriori ground control and check points measurements
- A multi-variant accuracy assessment of UAV imaging surveys performed in Pix4Dmapper suite [32,52]—a case of small investment area.
- Investigation of the impact of photos overlap and georeferences from UAV Global Navigation Satellite Systems (GNSS) receiver on the BBA results.
- Examination of the usefulness of UAV imagery acquired with the use of a medium spatial resolution [1] system camera for the development of full-featured photogrammetric products whose accuracy meets the appropriate technical requirements.
- Checking the possibilities of replacing geodetic field surveys by the UAV photogrammetric measurements.

The initial approach to the UAV mapping was signaled and presented by us in the publication [53], in which we found the possibility of using UAV imagery for inventory mapping in the case of the industrial estate. In the presented paper, we extend our considerations to the investment area and we discuss a new testing approach and other research results. The tests were conducted using a new input data set that contains a large number of low-altitude images and ground control points, additional ground check points and height ground check points. Also, we used another study methodology, which led us to pose new final results, and extended accuracy assessment (tabular summary of findings, histograms, screens) of respective stages of computation and image processing (multi-variant BBA, DSM and georeferenced orthomosaic generation). 

The discussion of the results and the accuracy assessment takes into account the Polish [43], technical requirements (accuracy defined with errors m_XY_ ≤ 0.10 m and m_Z_ ≤ 0.05 m) for the land surveys of planar-elevation measurements of well-defined terrain details and other practical applications.

## 2. Materials and Methods 

### 2.1. Study Area and Data Acquisition

The study site was an industrial area in the center of Poland in the Stryków municipality (51°52′38″ N, 19°35′21″ E), around 16 km northeast of the city of Łódź. This study site is representative for newly created industrial zones in the agricultural area because it contains different topographic objects, i.e., industrial buildings and their surroundings, access roads, a roundabout, and agricultural lands. In other study cases, the different localization and higher number of terrain objects may appear. 

The flight mission design and photo capturing were made by the Geoplan Polish company in Zgierz. A block of archival low-altitude uncalibrated photos acquired on 15 April 2016, at 8:35–9:08 local time as photographic documentation of the industrial investment [53], was used in the case study. The images were acquired by Trimble^®^ UX5 UAS equipped with Sony NEX-5T 16 megapixels digital compact system (mirrorless) camera with a CMOS (complementary metal-oxide semiconductor) sensor (size 23.5 × 15.6 mm, pixel count 4912 × 3264, pixel p_xy_ = 4.78 µm) and focal length f = 15.5 mm. Initial camera preferences were defined by shutter priority (1/2000 sec). The lens focus was set to infinity. The light source and white balance were set to Auto. The prevailing weather conditions on the day of flight mission were: mostly sunny sky with some clouds and the west wind speed was equal approximately to 5 m/sec and in gusts to 9 m/sec. Due to this fact, there were apparent drift and rotation (ω, φ, κ) of some images. 

The image block was characterized by the following photogrammetric parameters: flight mission area was P = 1.0253 km^2^, 858 photos in 41 photo strips oriented in direction N-S, crosswise to the long axis of the object, forward and side overlap p_%_ = q_%_ = 85%, projected flight height h = 150 m, ground sample distance (GSD) = 4.6 cm and base-to-height ratio ν = 0.15. The GNSS (single frequency receiver C/A, L1) of Trimble^®^ UX5 UAS flight data were saved in LOG file. The flight path is shown in supplementary files (Figure S2 in Appendix 1–6).

**Figure 1:**
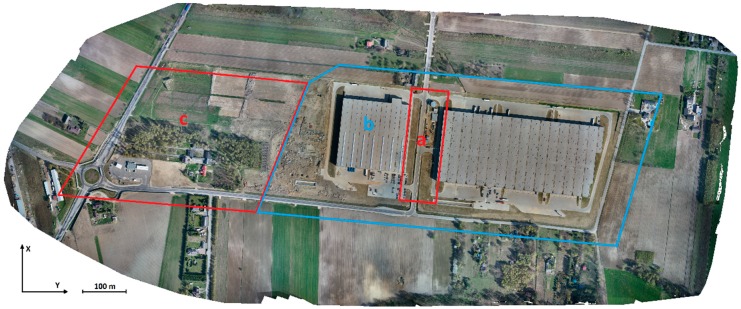
The test object Stryków with the defined study sections (**a**), (**b**), (**c**) on the orthomosaic generated in Pix4Dmapper.

The test field includes 5 artificially signalized GCPs, placed before the flight mission. For study purposes in 2019 the 295 natural, accurately defined ground points (water gate valves, manholes, curbstones) were identified on images, mainly used as check points (ChPs). The distribution of the GCPs and ChPs was a consequence of the field details placement, i.e., industrial buildings and elements of the utilities network. The GCPs and ChPs were distributed more densely in the area which was critical for matching (Figure 1a,b) and more sparsely in the remaining interest area (Figure 1c). Due to the thin sheet metal of the hall’s roofing it was impossible to place GCPs there. For tests we used the previous researchers’ experiences described in [27,49,54,55].

Furthermore, for the verification of the photogrammetric-generated DSM, the 1118 height ground ChPs in the form of dense cross-sections localized on scarps and terrain slopes were surveyed. The direct measurement of ground and height points was realized by applying the GNSS using the real-time network (RTN) method executed by virtual reference stations from the Polish Active Geodetic Network ASG EUPOS (virtual reference station solution). At every point, 10 fixed epochs were averaged in the RTN mode.

We decided to use the RTN solution because according to the ASG EUPOS (http://www.asgeupos.pl/index.php?wpg_type=serv&sub=gen) the advantage of RTN over solutions based on a single reference station RTK (real-time kinematic), is the possibility of better modeling of systematic errors related to, e.g., the work of satellite clocks and delays related to the signal propagation in atmosphere. Additionally, RTN solutions enable to achieve the coordinates determination repeatability regardless of the distance between the receiver and physical station. In the RTK mode the error in coordinate determination increases along with the distance to the physical station from which the corrections originate. Also, ASG EUPOS recommends using the RTN in precise positioning wherever possible. Another argument for using the RTN was the distance of apploximately 16 km to the nearest physical station of ASG EUPOS (LODZ). According to the Geospatial Positioning Accuracy Standards, the independent source of higher accuracy should be the highest accuracy feasible and practicable to evaluate the accuracy of the dataset. The RTN solution fulfills these guidelines.

The coordinates of the points were determined using a Trimble SPS882 survey-grade GNSS receiver in the PL-2000 zone 6 coordinate system (EPSG: 2177), and in the PL-KRON86-NH elevation system, with the estimated accuracy m_XY_ = 0.03 m and m_Z_ = 0.05 m. The measured GNSS points fell within the range:

- Position Dilution of Precision (PDOP) 1.0 ÷ 4.8 (<5), mean PDOP = 1.6
- Horizontal Dilution of Precision (HDOP) 0.6 ÷ 3.3 (<4), mean HDOP = 0.9
- Vertical Dilution of Precision (VDOP) 0.8 ÷ 3.4 (<4), mean VDOP = 1.3

which proves a good configuration of satellite geometry with am effect on positioning accuracy of GNSS points: mean σN = 0.012 m, mean σE = 0.008 m, mean σH = 0.020 m and root mean square value RMS = 0.025 m.

Because of the surface properties and a variety of factors that may impact the accuracy and quality of digital photogrammetric processing, the test field was divided into the following sections (Figure 1):

- The major part which includes the area between industrial buildings (**a**);
- The terrain around the industrial buildings (**b**);
- The roundabout, access road, gas station (**c**).

### 2.2. Used Methods and Software 

Due to the use of an archival image block in the study with the potential blur of images caused by wind gusts during Trimble^®^ UX5 UAS flight, their radiometric quality verification was carried out. The graphical contents and a large number of images indicate that the detection of blur on images should be realized using automatic methods [56]. In this case, for preprocessing, the implementation of the Open Source Computer Vision Library (OpenCV) in Python was used. In the beginning, the variance of the absolute values using the Laplacian operator (linear high-pass filter) by Pech-Pacheco et al. [57] (Equation (1)) was calculated:

(1) $$LAP\_VAR\left(I\right)=\overset{M}{\underset{m}{\sum }}\overset{N}{\underset{n}{\sum }}{\left[\left|L\left(m,n\right)\right|-\bar{L}\right]}^{2}$$

where $\bar{L}$ is the mean of absolute values; *L*(*m*, *n*) is the convolution of the image *Img*(*m*, *n*) with the mask *L*; *M*, *N* is the function size.

Since some of the images were tagged as potentially blurred, the decision was made to use the second approach for blur detection, which involves the frequency domain. The greyscale intensity images were transformed using Fourier transform *F*(*u*, *v*) accordingly to Equation (2):

(2) $$F\left(u,v\right)=\frac{1}{MN}\sum_{x=0}^{M-1}\sum_{y=0}^{N-1}f\left(x,y\right)e^{-j2\pi \left(\frac{ux}{M}+\frac{vy}{N}\right)}$$

where *M*, *N* is the number of columns and rows, *f*(*x*, *y*) is the pixel value [27]. The zero-frequency component has to be shifted to the center of the spectrum, and then the normalization from 0 to 255 has to be done (Equation (3)).

(3) $$FF\left(u,v\right)=255\frac{\log\left(1+\left|F\left(u,v\right)\right|\right)}{\max\left(\log\left(1+\left|F\left(u,v\right)\right|\right)\right)}$$

where *FF*(*u*, *v*) is Fourier transform component [27]. To determine the level of blur in the images, the skewness of the normalized 2D Fourier transform was computed according to Ribeiro-Gomes et al. [58] (Equation (4)):

(4) $$skew=\frac{\frac{1}{M\cdot N}\sum_{u=0}^{M-1}\sum_{v=0}^{N-1}{\left(FF\left(u,v\right)-\bar{FF\left(u,v\right)}\right)}^{3}}{{\left(\sqrt{\frac{1}{M\cdot N}\sum_{u=0}^{M-1}\sum_{v=0}^{N-1}{\left(FF\left(u,v\right)-\bar{FF\left(u,v\right)}\right)}^{2}}\right)}^{3}}$$

where *FF*(*u*, *v*) is each Fourier transform component shifted to the center of the spectrum and normalized and $\bar{FF\left(u,v\right)}$ is the mean value.

The photogrammetric measurements and advanced digital images processing were performed in the following software (in the order in which they were used):

- Matching (own application), Poland [59]
- IrfanView v. 4.41 (application by Irfan Skiljan), Vienna Austria (https://www.irfanview.com/).
- Pix4Dmapper Pro v. 4.3.31 (full license) of Pix4D S.A., Prilly Switzerland [32,52].
- RealityCapture v. 1.0.3.6310 (commercial license) of Capturing Reality s.r.o, Bratislava Slovakia [60,61].
- PhotoScan v. 1.5.1 build 7618 (commercial license) of Agisoft LLC, St. Petersburg Russia [10,62].
- CloudCompare v. 2.10.2 (GNU General Public License) of Telecom ParisTech and the research and development (R&D) division of EDF, Paris France [40,63].
- AutoCAD Civil 3D 2019 v.13.0.613.0 (academic license) of Autodesk, Inc., San Rafael USA (https://www.autodesk.pl).

**Figure 2:**
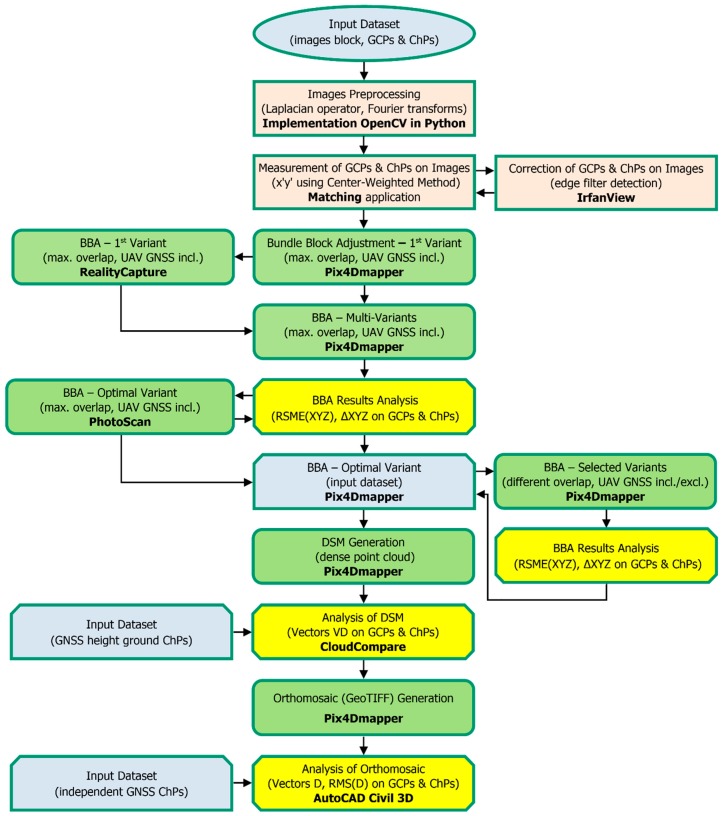
The workflow of measurement and processing proposed for unmanned aerial vehicle (UAV) surveys.

Figure 2 demonstrates the proposed workflow of this study for the accuracy evaluation of data collection based on low-altitude images. This scheme is an application of the original methodology for complex photogrammetric data collection using Pix4Dmapper as the main software for processing.

The signalized ground control points and natural ground points were measured on images using the center-weighted method (centroid operator) by means of Matching, the original software [59] with mean subpixel accuracy s_x’y’_ = 0.15 pixel. On the images, where points could not be measured automatically, the edge filter detection using IrfanView application was applied, and in some cases, the measurement was performed manually. Each ground control point (GCP) was identified on average in 38 images. 

At the first stage of computation in Pix4Dmapper, the combined BBA with camera self-calibration, including 218 ground points (5 signalized GCPs and 213 ChPs in three interest areas, Figure 1) were carried out with an automatic calculation of interior (standard designation: c, x’_0_, y’_0_, K_1_, K_2_, K_3_, P_1_, P_2_) and exterior (X_0_, Y_0_, Z_0_, ω, φ, κ) orientation parameters [64]. In the adjustment, the approximated initial camera positions in the WGS84 coordinate system (*.LOG file) were used, and the output coordinate system was PL-2000 zone 6 (EPSG: 2177).

Due to the limited possibilities of changing settings in Pix4Dmapper, it is not discussed in this paper. The individual settings could be checked in reports from computations, which are in supplementary information files (Appendix 1–6).

The Pix4Dmapper calculates the global projection error for points measured on images without signalizing blunders. To avoid manual inspection of 218 points (each one separately) on mean 38 images in Pix4Dmapper, the RealityCapture software with its fast signalization of blunders was used. This step allows the identification of 4 faulty points numbering on images and their correcting. 

**Figure 3:**
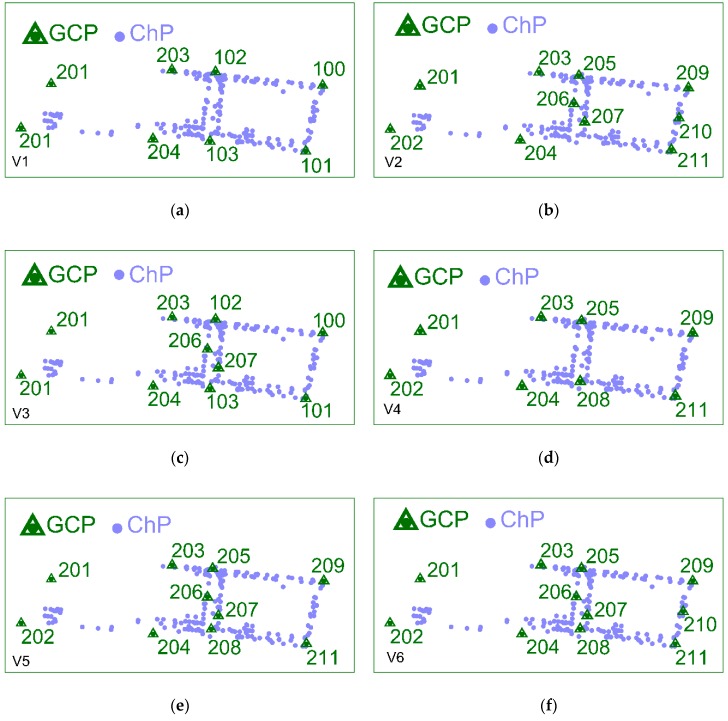
Configuration of ground control points (GCPs) and check points (ChPs) on the test object Stryków in the 6 BBA variants: (**a**) Variant 1 which contains 8 GCPs (4 signalized) and 210 ChPs; (**b**) Variant 2 which includes 10 GCPs and 208 ChPs; (**c**) Variant 3 consists of 10 GCPs (4 signalized) and 208 ChPs; (**d**) Variant 4 defined as 8 GCPs and 210 ChPs; (**e**) Variant 5 which is the extension of (**d**) by 2 GCPs between industrial buildings; (**f**) Variant 6 (it is characterized by 11 GCPs and 207 ChPs) which is the extension of (**e**) by 1 GCP at the right side of industrial building and close to it.

To find the optimal solution of the BBA, 44 computing variants were executed in Pix4Dmapper with various numbers and configuration of GCPs and ChPs. In research, it was assumed that for each variant a new project was set up. This approach led to computing each input dataset independently. The 6 selected configuration variants of GCPs (no. 100 ÷ 103—artificially signalized, no. 201 ÷ 211—natural signalized points), optimal in terms of the number and adjustment accuracy, are presented below in Figure 3.

To prepare the accuracy assessment methodology, i.e., statistical test parameters, number and distribution of ground points, the guidelines described in [65] were used. As a statistical parameter the RMSE values are described, which are interpreted as a “square root of the average of the set of squared differences between dataset coordinate values and coordinate values from an independent source of higher accuracy for identical points”. In this paper the 95% confidence level was not applied for accuracy assessment, because used values of RMSE are lower.

In the photogrammetry, various statistical tests are applied for accuracy evaluation. The statistical tests like Student’s t-distribution and Fisher-Snedecor’s F-distribution are used to verify the significance of camera interior orientation parameters computed by the bundle block adjustment with self-calibration or simultaneous (on-the-job) calibration [66]. The Cochran test is used to check the error significance of the 3D coordinates estimation (homogeneity of many standard deviations of independent samples). The blunder detection in the reference dataset, e.g., GCPs coordinates is possible using statistical tests, the Student *t*-test (used to assess the presence of bias) and chi-square test (used to determine whether random errors are adequate) [15,67]. In our study, for the measurement of GCPs and ChPs, we have used the Polish Active Geodetic Network ASG EUPOS, which is regularly tested and the correctness of its precision was proved before. According to commonly known adjustment computation principles [68] using statistical tests like Student *t*-test or chi-square was no justified in our case study.

To define reference values of limiting errors for planar-elevation measurements of well-defined terrain details, the ASPRS Positional Accuracy Standards for Digital Geospatial Data [44] and Polish [43] technical requirements were taken into account. The first document recommends the horizontal accuracy class RMSEx and RMSEy for orthoimagery purposes, and the vertical accuracy class RMSE_Z_ for digital elevation data. The second regulation describes the point position errors in the cartesian and elevation coordinate systems. Polish technical requirements were adopted in the paper as more rigorous. To analyze the accuracy of photogrammetric products in this study, the horizontal accuracy RMSE(X) and RMSE(Y) and vertical accuracy RMSE(Z) respectively to 0.10 m and 0.05 m were used.

The results of the BBA in Pix4Dmapper were analyzed in terms of GCPs and ChPs number, RMSE(XYZ) on GCPs and RMSE(XYZ) on ChPs. Based on the accuracy assessment of the BBA for further works, variant 6 (Figure 3f) was chosen as optimal. The use of one GCP (210) in section b (Figure 1) and two GCPs (206, 207) in section a (Figure 1) was caused by significant matching errors on roof surfaces and in their immediate surroundings, and as a consequence, the significant errors of photogrammetrically calculated ChPs coordinates. Since it is possible to obtain different results in different “black-box” applications [10,11,69], for an independent verification of the results, variant 6 was computed in the PhotoScan application. It is a well-known suite and uses other computation algorithms for photogrammetric product generation. 

The next aspect was the evaluation of the impact of less forward and side overlap, and reduction of image number on the significant decrease of the BBA accuracy. Based on the BBA optimal variant, the computations were carried out in two options: with and without approximated UAV GNSS coordinates of images’ projection center (position accuracy of several meters). The forward overlap p_%_ = 65% was obtained by removing every second photo in each strip. The side overlap q_%_ = 65% and q_%_ = 45% were received by removing from the initial image block every second, and every second and third strips, respectively.

Advanced images processing in Pix4Dmapper included the generation of dense point clouds, the DSM and finally a georeferenced orthomosaic in GeoTIFF format. 

The 1118 height points and dense point cloud generated in Pix4Dmapper were imported to the CloudCompare application [63] for comparison of Z component using the least squares planar fitting of 3D points [40]. For fitting the mathematical model on the nearest point and its 4 neighbors the function (Equation (5)) was applied, which assumes that the sum of squared errors between the Z_i_ and the plane values Ax_i_ + By_i_ + C is minimized.

(5) $$E\left(A,B,C\right)=\sum_{i=1}^{m}{\left[\left(Ax_{i}+By_{i}+C\right)-Z_{i}\right]}^{2}$$

As a result, the vertical distance from the compared point cloud to the computed DSM is determined. The assessment of vertical distances distribution was analyzed. 

For the orthorectification in Pix4Dmapper, the same GSD size of the orthomosaic and acquired images (GSD_ortho_ = GSD = 4.6 cm) was assumed. The received georeferenced orthomosaic and XY coordinates of 11 GCPs and 289 ChPs (incl. 207 ChPs from the BBA) were imported to AutoCAD Civil 3D. The 2D data were compared, and the values and directions of deviation vectors were determined.

## 3. Results

The data processing in all of the used software was carried out on a workstation with the processor Intel^®^ Core™ i9-7940X, 128 GB RAM DDR4-3200 MHz memory, MSI 1080 Ti graphic card and Samsung 960 Pro SSD hard drive. 

### 3.1. Blur Detection on the Images

At the first step, Laplacian operator (Equation (1)) shows 196 images that were tagged as potentially blurred. The Fourier Transform (Equations (2)–(4)) indicated that 124 images could be blurred. The manual inspection was performed, to check if the blur occurred on the images. It was found that the images pointed out by algorithms included the roofs of industrial buildings, which were characterized by the same color and amorphous texture. There were no images that contain motion and optical blur, so all of the images were allowed for further computation.

### 3.2. Bundle Block Adjustment and Camera Self-Calibration

For our tests, the in-flight self-calibration, which describes the real acquisition condition and gives more accurate camera calibration [34], was used. The results of the 6 BBA solutions with reports of the camera standard parameter calibration are included in supplementary information files (Appendix 1–6). The main difference obtained in the 6 BBA variants were the values of reprojection error, which are understood as the distance between the marked and the reprojected point on one image. Due to very similar results (max. differences in variants 1 ÷ 6 are Δc = 1.3 pix, Δx’_0_ = 0.3 pix, Δy’_0_ = 0.2 pix, ΔK_1_ = 0.000, ΔK_2_ = 0.001, ΔK_3_ = 0.000, ΔP_1_ = 0.000, ΔP_2_ = 0.000) of interior orientation parameters calculated in Pix4Dmapper suite, which does not allow the interference in computation process, and because camera self-calibration is well described in the literature [13,25,26], the parameters are not discussed here. 

Table 1 presents only the results of the final 6 BBA solutions (218 GCPs and ChPs in various combinations) obtained in the Pix4Dmapper suite. The maximum differences between root mean square errors on GCPs obtained in 6 variants of the BBA in horizontal and vertical planes are respectively to RMSE(X) = 0.002 m, RMSE(Y) = 0.007 m, RMSE(Z) = 0.013 m. In the case of ChPs, the RMSE are following RMSE(X) = 0.003 m, RMSE(Y) = 0.001 m and RMSE(Z) = 0.013 m. 

**Figure 4:**
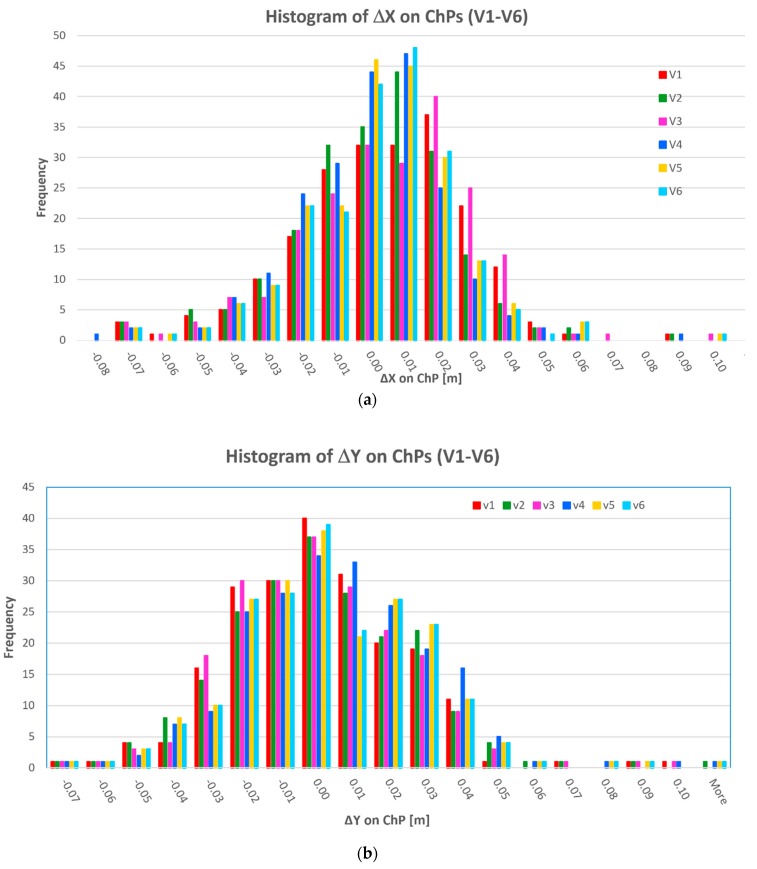

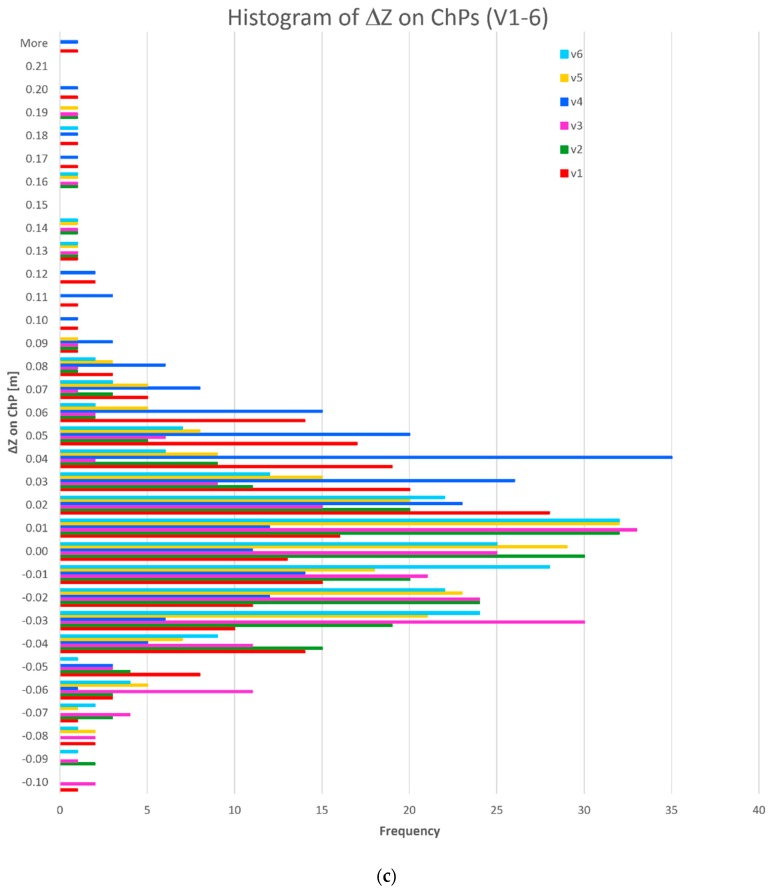
Histograms of deviations ΔX, ΔY, ΔZ on ChPs in computed variants (V1 ÷ V6) of the bundle-block adjustment (BBA): (**a**) ΔX deviation on ChPs; (**b**) ΔY deviation on ChPs; (**c**) ΔZ deviation on ChPs.

The distribution of deviations ΔX, ΔY, ΔZ (differences between coordinates measured using GNSS and obtained in the BBA) on ChPs for 6 variants are presented in Figure 4.

Table 2 includes the analysis of ΔZ deviations in each computation BBA variants in more detail. In the analysis, the 5 intervals of the ΔZ deviations were assumed. The ΔZ thresholds are the result of the current Polish survey technical regulations, where the N_ΔZ_ is the number of absolute deviations values [ΔZ] for defined intervals.

**Figure 5:**
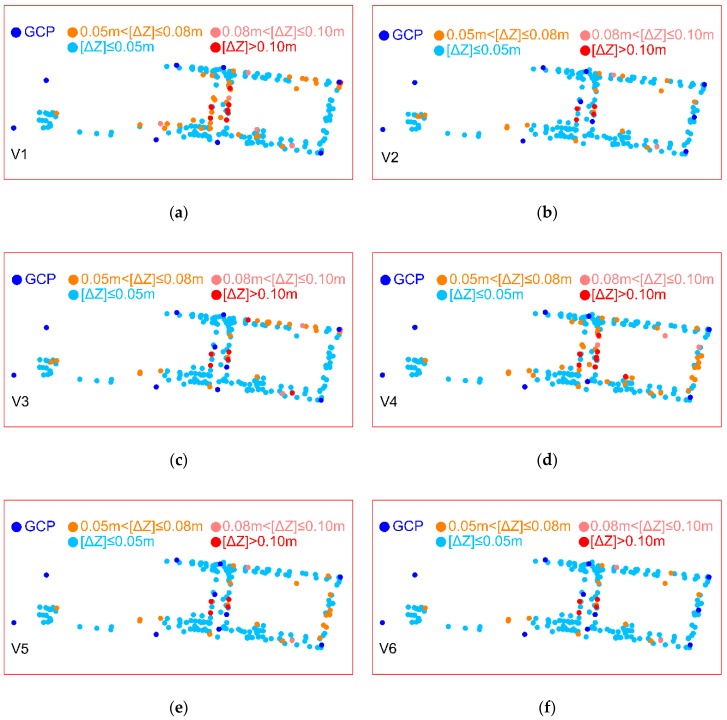
Localization of GCPs and ChPs in the BBA with representation of [ΔZ] in deviation intervals: (**a**) Variant 1; (**b**) Variant 2; (**c**) Variant 3; (**d**) Variant 4; (**e**) Variant 5; (**f**) Variant 6.

The localization of the points (GCPs, ChPs), which belong to defined ΔZ intervals, is shown in Figure 5.

Upon the analysis of particular histograms (Figure 4) and the values of [ΔZ] deviation (Table 2) can be concluded that the most advantageous distribution of absolute values deviation, especially for [ΔZ] values occurred in variant 6. Furthermore, this variant poses the least percentage (9.7%) of [ΔZ] values higher than 0.05 m. The use of one GCP (210) in section b (Figure 1) and two GCPs (206, 207) in section a (Figure 1) enhanced the accuracy of Z coordinate determination. The differences of [ΔZ] deviation between variants 4 and 6 in individual sections a, b, c are, respectively, to 0.024 m, 0.008 m, 0.002 m. 

To check the correctness and reliability of the optimal variant results (V6), the BBA was performed in an additional research and computation tool PhotoScan. This approach allows us to do an independent verification of digital processing results and to receive some additional information about applications’ reliability and functionalities. Supplementary files (Appendix 7) contains the report of computations.

Table 3 contains the comparison of variant 6 of the BBA results obtained in Pix4Dmapper and PhotoScan. The RMSE differences received on GCPs are respectively to RMSE(X_GCP_) = 0.009 m, RMSE(Y_GCP_) = 0.005 m and on ChPs amount to RMSE(X_ChP_) = 0.002 m, RMSE(Y_ChP_) = 0.002 m, RMSE(Z_ChP_) = 0.004 m. 

**Figure 6:**
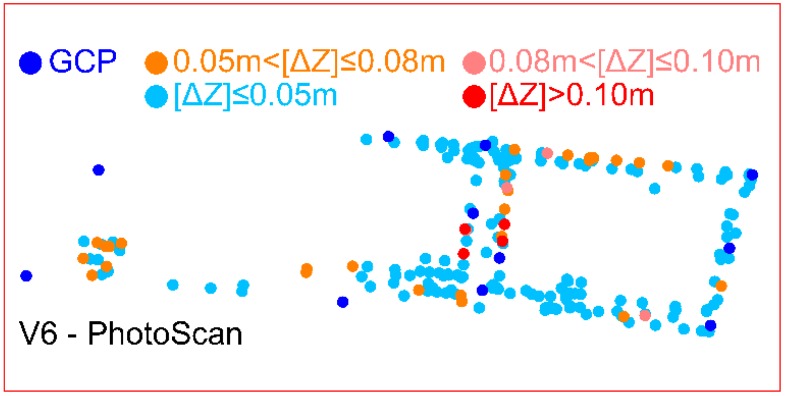
Localization of GCPs and ChPs in the variant 6 of the BBA performed in PhotoScan with the representation of number N_ΔZ_ in [ΔZ] deviation intervals.

**Figure 7:**
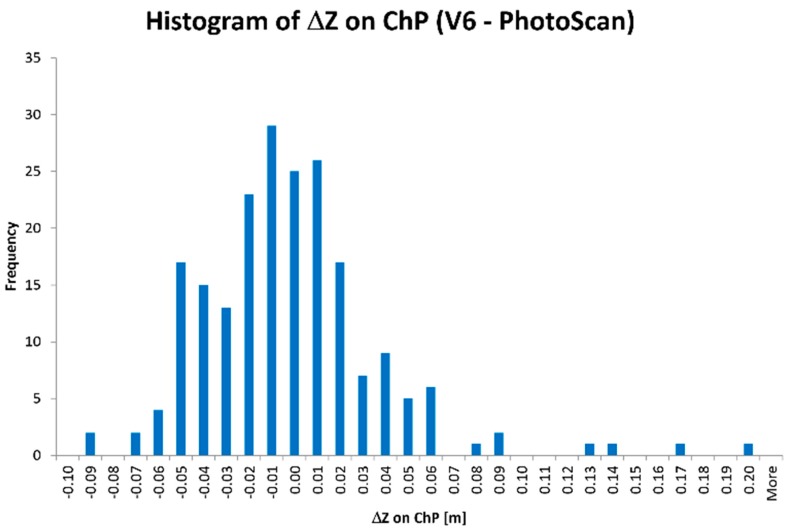
Histogram of ΔZ deviations on ChPs calculated in the BBA in PhotoScan application.

Table 4 presents the comparison of variant 6 of the BBA results with an extended analysis of the ΔZ deviations on ChPs. The main difference between the obtained outcomes is the number N_ΔZ_ of [ΔZ] deviations in the range of 0.05 ÷ 0.08 m to the detriment of the PhotoScan. The ChPs localization determined in the PhotoScan with the information about the [ΔZ] value is displayed in Figure 6. Figure 7 shows the histogram of ΔZ deviations on ChPs computed by the PhotoScan software.

The difference between number N_ΔZ_ of [ΔZ] deviations above threshold 0.05 m is 16 (ca. 8%). It is a consequence of a deviations number (6—Pix4Dmapper and 17—PhotoScan), which belongs in the 0.05 ÷ 0.06 m range. Therefore, the solutions in both applications are comparable.

In the authors’ opinion, the differences in values and in the number N_ΔZ_ of [ΔZ] deviations which are higher than 0.05 m are probably caused by another stochastic (weight) model used in computation algorithms in both types of software, e.g., for tie points measured automatically.

### 3.3. Influence of Image Overlap and Global Navigation Satellite Systems (GNSS) Exterior Orientation 

The assessment of the influence of image overlap value and the use of UAV GNSS external orientation in the bundle adjustment was carried out in multi-variant (7 new variants) computation using the optimal BBA variant 6 as reference. The results of the reference and additional solutions with reports of the camera standard parameter calibration are included in supplementary information files (Appendix 6, 8–14). Table 5 contains the calculation results for four variants (V6_p%-q%_) of image overlap, respectively to p_%_ = q_%_ = 85% (V6_85-85_), p_%_ = 85% and q_%_ = 45% (V6_85-45_), p_%_ = q_%_ = 65% (V6_65-65_), p_%_ = 65% and q_%_ = 45% (V6_65-45_) with additional observations of exterior orientation parameters included in the adjustment. Table 6 presents the same adjustment variants, but with observations of projection centers coordinates excluded in the adjustment. 

In the case (V6_65-45_) of minimal forward p_%_ = 65% and side q_%_ = 45% overlap (ca. 6.2-fold reduction of image number) the RMSE(Z_ChP_) is a little bit over the critical value 0.05 m and the percentage of points with the absolute deviations values [ΔZ] > 0.05 m rise 3.5-fold. In two other variants (V6_85-65_ and V6_65-65_), the absolute deviations values [ΔZ] are lower than 0.05 m. The percentage of points with the absolute deviations values [ΔZ] > 0.05 m rise 2.5-fold. The exclusion of approximated coordinates of projection centers in the BBA process is not causing significant changes in obtained accuracy.

In regard to variant V6_85-85_ (incl. UAV GNSS) the max. differences of interior orientation parameters calculated for 3 variants of the BBA with UAV GNSS data and reduced overlap are the following: Δc = −7.3 pix, Δx’_0_ = 1.3 pix, Δy’_0_ = 3.0 pix, ΔK_1_ = 0.000, ΔK_2_ = 0.000, ΔK_3_ = 0.001, ΔP_1_ = 0.001, ΔP_2_ = 0.000. Max. differences of parameters between variant V6_85-85_ (excl. UAV GNSS) and 3 variants of the BBA without UAV GNSS data and reduced overlap are respectively: Δc = 9.3 pix, Δx’_0_ = 1.4 pix, Δy’_0_ = 3.0 pix, ΔK_1_ = 0.000, ΔK_2_ = 0.000, ΔK_3_ = 0.000, ΔP_1_ = 0.001, ΔP_2_ = 0.000. 

The value differences of parameters calculated between relevant variants with and without UAV GNSS data are very similar except for variant V6_65-45_ (p_%_ = 65% and q_%_ = 45% overlap), respectively: Δc = 3.3 pix, Δx’_0_ = 0.1 pix, Δy’_0_ = 2.4 pix, ΔK_1_ = 0.000, ΔK_2_ = 0.000, ΔK_3_ = 0.001, ΔP_1_ = 0.001, ΔP_2_ = 0.000. The standard deviation of determined parameters are in range s_c_ ∈ <0.5; 0.8> pix, sx’_o_ = sy’_o_ ∈ <0.07; 0.14> pix.

### 3.4. Comparison of Photogrammetric-Generated Digital Surface Model (DSM) and GNSS Point Cloud

**Figure 8:**
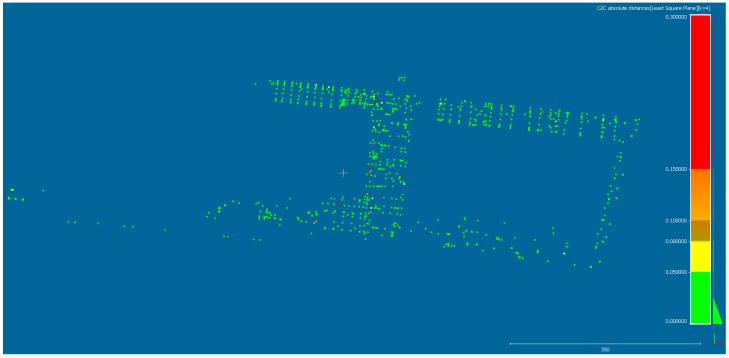
Localization and histogram of the vertical distances (VD) between computed digital surface model (DSM) in Pix4Dmapper and GNSS point cloud.

Due to the requirements of height accuracy (RMSE(Z) ≤ 0.05 m), the generated DSM in Pix4Dmapper (variant 6) was compared to the point cloud obtained using GNSS. The determination of DSM undulation is realized by Equation (5) and is presented in Figure 8.

For most of the 1418 points (300 ChPs and 1118 height ground ChPs), the values of the vertical distance VD (height differences) belong to 0 ÷ 0.05 m range. Only for some random points, the estimated vertical distances are higher than 0.05 m. The vertical distances outliers comprise 4.3% (61) of the total number of points (1418). This fact confirms that DSM is correctly generated even in the area with the discontinuity of the terrain surface (scarps and terrain slope).

### 3.5. Comparison of the Orthomosaic and GNSS Data

The photogrammetric planar data collection was performed using orthomosaic. The outcome GeoTIFF ortomosaic (Figure 1) of Pix4Dmapper is 37058 × 15284 pixels (855 MB). To verify if the georeferenced orthomosaic comply with the mapping requirement (RMSE(XY) ≤ 0.10 m) of the point position in the cartesian coordinate system, the 2D reference coordinates of 11 GCPs, 207 ChPs and 82 supplementary ChPs measured by the GNNS method were applied. The 82 additional ChPs were the densification of the ChPs set and allowed an independent accuracy verification of the orthomosaic.

**Figure 9:**
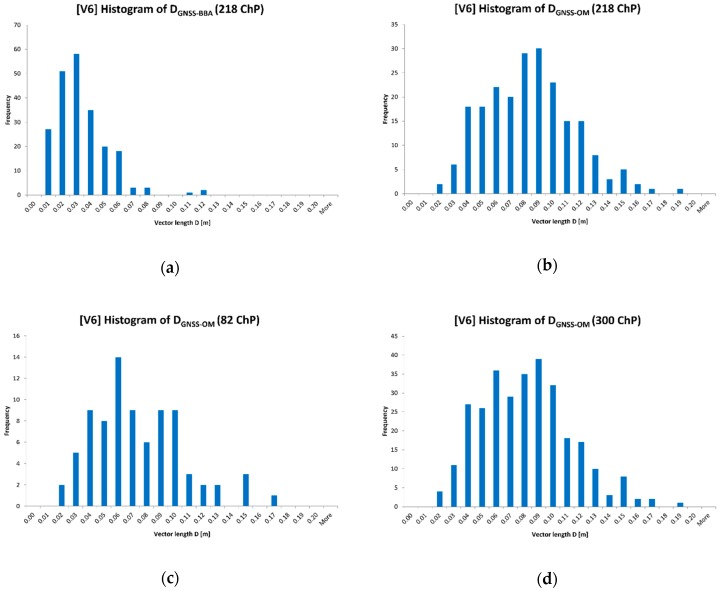
Length distribution of deviation vectors D: (**a**) D_GNSS-BBA_ (218 ChPs); (**b**) D_GNSS-OM_ (218 ChPs); (**c**) D_GNSS-OM_ (82 ChPs); (**d**) D_GNSS-OM_ (300 ChPs).

The photogrammetric 2D data collection (object vectorization) on the georeferenced orthomosaic can be treated as detailed land surveys. On the basis of the manual measurement on the GeoTIFF raster in AutoCAD Civil 3D, the deviation vectors D_GNSS-BBA_ and D_GNSS-OM_ were defined (GNSS—coordinates from GNSS measurement, BBA—coordinates computed in the bundle block adjustment, OM—coordinates from orthomosaic measurement). The a priori accuracy of manual pixel coordinates measurement was set at the level of 0.33 pixel size, which corresponds to 0.015 m in the terrain. Table 7 describes the accuracy evaluation of georeferenced orthomosaic. The length distributions of deviation vectors D in the plane are presented in Figure 9. 

The measurement on the orthomosaic founds that 61 (20.3%) vectors exceed the threshold value of 0.10 m, where the calculated measurement accuracy for 300 ChPs was RMS(D_GNSS-OM_) = 0.082 m. In comparison with the reference data (2D coordinates) obtained from the BBA, the number of length vectors outliers increases up to 20-fold. The analysis of the histograms confirms that trend. 

To check if the constant shift on the generated orthomosaic exists, the directions of deviation vectors were studied. The vectors’ directions were assigned to 4 quarters of the coordinate system. Table 8 contains the results of the verification.

**Figure 10:**
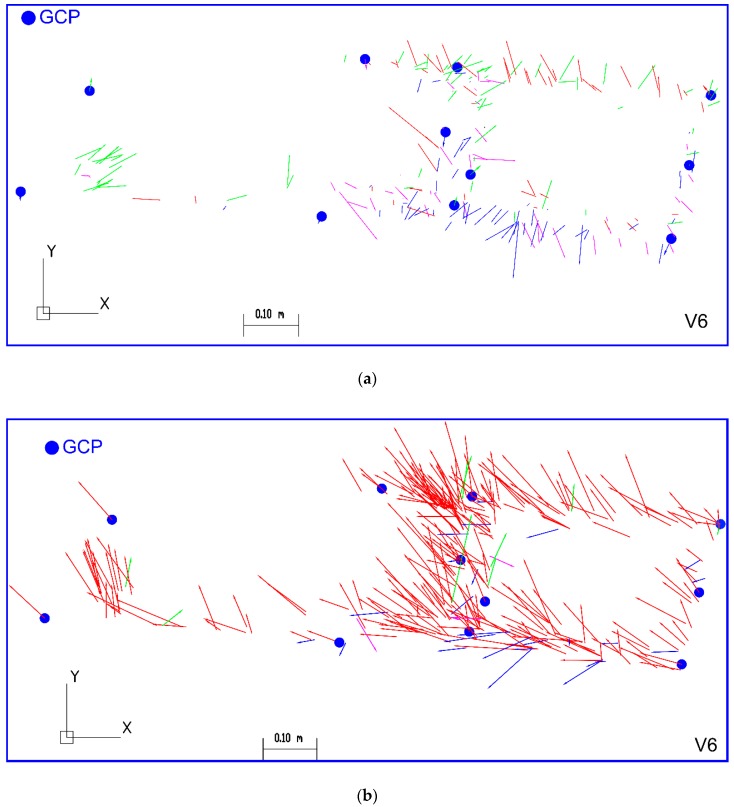
Localization of vectors D which include length and direction: (**a**) D_GNSS-BBA_ (218 ChPs); (**b**) D_GNSS-OM_ (300 ChPs).

Figure 10 presents the localization of ChPs with the information about vectors’ length and directions, where each direction is marked with the following colors: N–E: green, N–W: red, S–W: blue, S–E: purple.

The analysis of the analytical BBA data presented a similar direction distribution of the vectors D_GNSS-BBA_. On this distribution, the systematic errors are not found. However, directions distribution of vectors D_GNSS-OM_ measured on georeferenced orthomosaic has a systematic shift in the N–W (**↖**) direction. The analysis of vectors (D_GNSS-BBA_ and D_GNSS-OM_) localization in both cases shows that the similarity of distributions does not exist. The values of deviation vectors on the orthomosaic were affected by the orthorectification process. In the authors’ opinion, the direction of shadow projection and different lighting conditions on image sequences in flight strips may influence on the length and the direction of error vectors.

### 3.6. Inspection of the Existing Digital Maps

**Figure 11:**
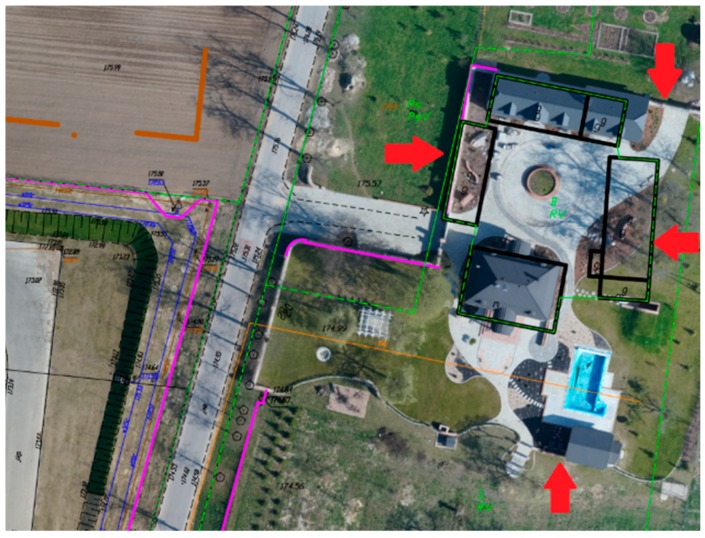
Visualization of the outdated information on the vector base map using orthomosaic in AutoCAD.

**Figure 12:**
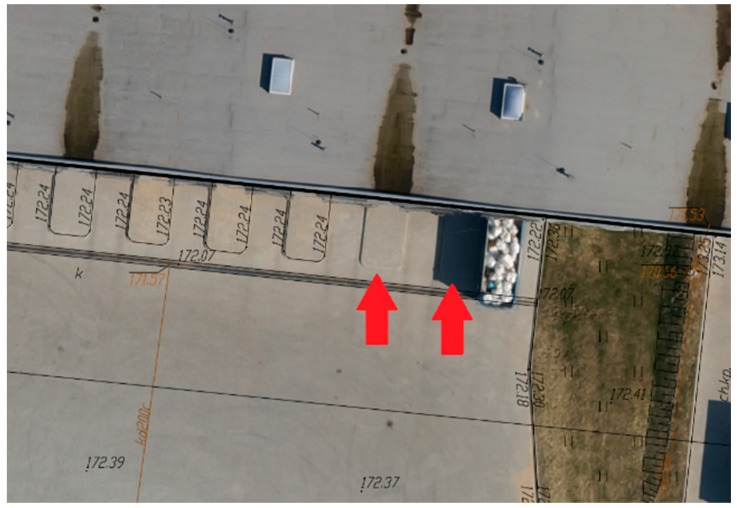
Visualization of the deficiencies on the vector base map using orthomosaic in AutoCAD.

The orthomosaic generated from UAV images is a relatively low-cost and quickly obtained photogrammetric product. Its actual photographic content allows the easy and fast visual detection of outdated information (Figure 11) and incompleteness (Figure 12) of existing vector digital maps, which are the result of geodetic detailed field surveys.

## 4. Discussion

For accuracy analysis, the current Polish technical standards for the land surveys (planar-elevation measurements of well-defined terrain details) in order to undertake large-scale mapping, included in the Regulation of the Minister of the Interior and Administration in Poland, No. 263 of 9 November 2011 [43], were used as the reference. They precisely describe the accuracy of field geodetic surveys of the ground details, which are uniquely identifiable, retaining the long-term invariability of shape and position. The measurements should achieve the point position in the cartesian coordinate system with error m_XY_ ≤ 0.10 m and point height position in the elevation system with error m_Z_ ≤ 0.05 m. The geodetic control network points used as a reference for dedicated field surveys are treated as errorless. 

The planimetric mapping (land survey) is performed on the assumption that the accuracy (error m_XY_) of a point location of terrain details relative to the nearest horizontal geodetic control network is not less than:

- 0.10 m: Ist survey group, i.e., survey marks of the control network, boundary points, building objects, and construction equipment, including elements of the utilities network, directly available for measurement;
- 0.30 m: IInd survey group, i.e., buildings and earth devices in the form of embankments, excavations, dikes, dams, ditches, canals and artificial water reservoirs; invisible parts of buildings and construction equipment, including objects of covered infrastructural networks; land use objects, in particular: parks, green areas, lawns, playgrounds and rest, squares, single trees as well sport fields;
- 0.50 m: IIIrd survey group, i.e., land use contours and soil outcrops for the needs of the land soil classification; watercourses and water reservoirs with natural boundaries; section boundary in the forests and national parks.

The geodetic height measurement of terrain elements is performed on the assumption that the accuracy of point height (error m_Z_) in relation to the nearest height (vertical) geodetic control network is not less than:

- 0.02 m: for underground pipes and sewage devices;
- 0.05 m: for building objects and construction equipment as well as height spots marked in the field;
- 0.10 m: for terrain structures and flexible lines or electromagnetically measured underground objects of the infrastructural network; and non-marked height spots of characteristic points of the ground elevation.

The specification of terrain detail types and their planar-elevation survey accuracy contained in the regulation [43] mentioned above in principle applies to the direct geodetic survey and does not strictly concern the photogrammetric measurement. Due to the lack of the technical guidelines for practical usage of UAVs images in geodesy, the listed max. errors for surveys of well-defined over ground points were adopted in tests for the accuracy assessment of 2D and 3D photogrammetric measurement. 

In this paper, we have compared conventional survey quality considerations with a photogrammetric approach to evaluate the accuracy of UAV imagery data collection. Our case study was performed according to Federal Geographic Data Committee mapping accuracy standards [65]. For testing purposes, features with known horizontal position (high degree of accuracy) and position with respect to the geodetic datum were used. Also, the independent source of higher accuracy was acquired separately from data used in the aerotriangulation solution. According to [65], check points were distributed more densely in the area of interest and more sparsely in that of little or no interest. 

In the study, the set of images used for processing was radiometric proper; they did not contain motion and optical blur. The process of the complex photogrammetric data collection was performed in Pix4Dmapper software in three stages. The first computation stage was the bundle block adjustment. Empirical tests have shown the need to apply 11 GCPs (variant 6 of the BBA) in the configuration (Figure 3f) related to the buildings localization. The use of 3 additional GCPs was necessary to minimize the reprojection errors on the stage of the BBA and to eliminate the influence of amorphous surfaces on the image matching. The interior orientation parameters computed in self-calibration depend significantly on the configuration of the image block (image number, block and strip geometry, forward and side overlap, drift, shift), GCPs (point signalize type, shape, size, color, number, distribution, resolution, accuracy) and UAV GNSS accuracy. The accuracy of camera calibration parameters determined using BBA affects the accuracy and reliability of estimated 3D ground point coordinates. The simultaneous self-calibration method most accurately corresponds to the real conditions of images acquisition and computation, and provides the optimal functional model of the solution by bundle method.

The 3D ground coordinates of all the pass points (natural signalized terrain details) estimated using the BBA can be the background for a 3D vector digital map production. The external accuracy measure, i.e., the RSME on 207 ground ChPs (XYZ) were respectively to RMSE(X) = 0.023 m, RMSE(Y) = 0.026 m and RMSE(Z) = 0.036 m. In respect of theoretical inhomogeneous horizontal and vertical accuracy of the estimated coordinates in the bundle adjustment, the ΔZ deviations were analyzed. It turned out that ca 90% of ΔZ deviations on 207 ChPs were to ΔZ ≤ 0.05 m and only ca. 2% to ΔZ > 0.10 m. Barry and Coakley [39] used in their study 10 GCPs and 45 ChPs on the small field area (0.3250 ha). They acquired 95% reliably within 0.041 mm horizontally and 0.068 m vertically with the 1.17 cm size of GSD. Benassi et al. [70] in their tests used eBee RPAS with direct georeferencing on the test field (20 ha) with 12 GCPs, 14 ChPs. They declare standard deviations of planar camera positions between 0.01 ÷ 0.02 m and 0.03 ÷ 0.05 m in vertical direction, and acquired average horizontal RMSE on ChPs of 0.022 m and 0.055 m in elevation. In our opinion, the small sample of ChPs, their localization far from high objects and no independent points render them inadequate to do the accuracy assessment of RTK RPAS measurements. Besides with fewer GCPs used as a control, error magnitudes on control and check points diverged, which was confirmed by James et al. [41].

Another study aspect relative to the initial dataset was the assessment of the impact of less overlap and, as a consequence, a reduction of the images number in the bundle adjustment. We found that the optimal variant in terms of accuracy and computing time using standard grade workstation is that with reduced photos overlap at the level of p_%_ = q_%_ = 65% (ca. 4-fold decrease of image number). However, the optimal configuration of the GCPs is a condition for reducing the photos number, which is confirmed by the comparison of BBA results for variants V1 and V4 (Table 1), and V6_65-65_ (Table 5). Tests shown that for block of nadir images with decreased overlap to p_%_ = q_%_ = 65%, it is possible to achieve the horizontal and vertical accuracy of ground objects at the level of RMSE(X_ChP_,Y_ChP_) = 0.5 GSD and RMSE(Z_ChP_) = 1 GSD (0.046 m), but in this case the number N_ΔZ_ of [ΔZ] deviations higher than 0.05 m increases ca. 2.5-fold. As expected due to the low position accuracy (horizontal 5 m and vertical 10 m) of the GNSS single-frequency receiver (C/A, L1) located on the Trimble^®^ UX5 UAS platform, the inclusion of approximated coordinates of projection centers in the bundle adjustment in all tested variants did not affect on the computation results. For the BBA results evaluation, the number and distribution of ChPs, which are treated as tie points in computation should be taken into consideration. Mostafa in his research [71] state that changing the forward and side overlap from p_%_ = q_%_ = 80% to p_%_ = 80% and q_%_ = 40% resulted in the same accuracy within the measurement noise. The investigations were conducted with GSD = 0.008 m and GSD = 0.016 m. Furthermore, it was indicated [71] that ground object positioning accuracy is about 2 ÷ 3 GSD value, and height accuracy is about 4 ÷ 5 GSD value.

The DSM which was generated in the second processing stage has been validated by 1418 GNSS height check points in CloudCompare application. The approach was realized successfully before [53]. The ca. 96% of the height differences are related to ΔZ ≤ 0.05 m and only ca. 4% are outliers ΔZ > 0.05 m. Oniga et al. [55] compared results with terrestrial laser scanning (TLS) data and obtained a standard deviation equal to 0.098 m. Wierzbicki and Nienaltowski [42] created the triangulated irregular network (TIN) model based on points measured using the GNSS RTK technique, and they achieved a standard deviation of 0.090 m for height differences. To avoid the interpolation of the irregular shape of the ground, our study contains height differences only for points measured in the field.

Finally, the manual measurement of the well-defined points on the GeoTIFF orthomosaic (GSD_ortho_ = 4.6 cm) was carried out in AutoCAD Civil 3D. The photogrammetric 2D data collection (pointwise object vectorization) on the georeferenced orthomosaic can be treated as detailed land surveys. To take into account the limiting error (m_L_ = 2 m_0_) of the GeoTIFF manual measurement, it could be considered that ca. 90% of deviation vectors D on the set of 300 ChPs fulfill the planar accuracy requirements RMSE(XY) ≤ 0.10 m. According to the authors’ knowledge, this procedure has not been used before. For describing the accuracy of orthomosaics, researchers used the BBA results [36,72]. Hung et al. [12] and James et al. [41] analyzed the directions and length of deviations vectors for GCPs and ChPs from the BBA. In our study, we also checked them on the orthomosaic, which showed that the constant shift occurs despite the random distribution of vectors from the BBA.

It is worth noting that in a complex process of orthomosaic generation, error propagation occurs. The final geometric accuracy and radiometric quality of orthomosaic is influenced by the many factors caused by, e.g., target marking (GCPs) and coordinate measurement errors, incorrect stochastic (weighted) model in the BBA, image-matching errors, estimated parameters errors of interior and exterior orientation, insufficient correction of image distortions, densified point cloud noise, final density and quality of DSM, applied orthorectification and digital interpolation method during the resampling, radiometric correction of respective orthoimages, quality of mosaicking.

Analyzing the real, not ideal conditions of the input dataset, it can be assumed that the following factors could have a significant impact on the accuracy of the final results: a relatively large size of GSD, a small number and incorrect artificial signalization of GCPs, inappropriate orientation of the strips relative to the shape of the photo block, and difficult conditions of dense matching as a consequence of the amorphous texture of the industrial object surface.

The positive experimental results of the presented study encourage the authors to continue research on the accuracy evaluation of photogrammetric products based on UAV imagery. Therefore, further work will be carried out on a topographically diverse test field, a representative for the general case of study, and the negative factors will be eliminated, which decreases the accuracy of the UAVs survey. 

## 5. Conclusions

The use of unmanned aerial vehicles (UAV) for image-based surveys is becoming increasingly widespread across the geosciences and in industries such as engineering and surveying. However, the increasing availability of such data has not been paralleled by an equivalent increase in research about the data quality. The aim of the study is to maximize the accuracy and reliability of UAV survey results, whilst optimizing the input dataset and digital-processing requirements.

The presented study evaluates the accuracy of photogrammetric 2D and 3D data collection based on processed medium resolution images acquired from Trimble®UX5 UAS production flight mission, in the case of a small investment area with a focus on the influence of ground control points configuration. The study site is representative for other areas with similar characteristic and location of terrain objects.

The initial dataset was characterized by a block of 858 digital images with 16 megapixel resolution, 4.6 cm ground sample distance, 5 artificially signalized ground control points. For the verification of the results, the 295 ground check points and 1118 height check points were measured applying the GNSS using the real-time network method with the mean sigma (NEH) respectively to 0.012 m, 0.008 m, 0.020 m. 

The photogrammetric data collection for well-defined points was carried out using the Pix4Dmapper suite in three processing stages: in the bundle block adjustment, on the generated digital surface model and the georeferenced orthomosaic. Furthermore, the bundle block adjustment was performed using RealityCapure and PhotoScan packages. In addition, the extended accuracy analyses were achieved on the digital surface model using CloudCompare application and on the orthomosaic supported by AutoCAD Civil 3D.

The main original research contributions of this paper are the following:

- Creating a new methodology for complex accuracy assessment of computing pipeline of particular photogrammetric products;
- Proving high accuracy and significant statistical reliability of photogrammetric 2D and 3D data collection based on UAV medium spatial resolution imagery with lower than 5 cm ground sample distance;
- Stating the absolute need for a multi-variant bundle block adjustment and error assessment to search for optimal ground control points configuration for further advanced digital processing chain, which allows minimizing image-matching errors caused by the amorphous texture;
- Proving the high horizontal and vertical accuracy (root mean square error less than or equal to 0.10 m and 0.05 m) for well-defined terrain points of photogrammetric products based on UAV imagery, which fulfills Polish and US technical requirements;
- Finding the optimal photos overlap in terms of accuracy and computing time;
- Proving the lack of influence of low-accuracy image georeferences on the bundle block adjustment results;
- Confirming the appropriateness of UAV imagery and dedicated photogrammetric software packages for 2D and 3D data collection in vector, raster and hybrid form in order to undertake large-scale basic, thematic and inventory mapping;
- Proving the possibilities of replacing direct geodetic field measurements by the UAVs photogrammetric measurements with the accuracy for large-scale mapping;
- Verifying the usefulness of UAV imagery and their products for regular updates existing digital base maps and geodetic database.

The accuracy of the achieved products shows the full practical utility of a digital compact system camera for geodata collection. High accuracy could be obtained only under the conditions of multi-variant image processing and extended error analysis, according to the proposed workflow. In the case of a smaller ground sample distance, the accuracy of data collection will be significantly higher. 

When the Pix4Dmapper package is applied for the bundle-block adjustment, additional software should be used in the post processing to find the image coordinates measurement blunders and to verify primary adjustment results. For the accuracy assessment of the georeferenced orthomosaic, we propose to analyze the distribution and length of deviation vectors. 

The more frequent application of UAV imagery in geodesy and geomatics is, unfortunately, limited by the lack of dedicated national technical regulations and guidelines.

Further study will be carried out on the specially designed test field characterized by a high number of artificially signalized and evenly distributed ground control and check points which have been measured with high accuracy using static GNSS method and precise leveling. In addition, the research area should have a block of nadir and oblique images with a small ground sample distance (1 ÷ 2 cm level), acquired with a medium or large-format digital camera with high-resolution and a digital surface model based on dense point clouds obtained from airborne laser scanning, which will be used as a reference. A new test field will allow for testing the quality and accuracy of standard products (bundle-block adjustment, digital surface model, georeferenced orthomosaic) and other products derived from UAV imagery that were not the subject of the described study, i.e., true orthophoto maps, 3D object reconstruction, and 3D city models in the two-level of detail (LOD3 and LOD4).

## Figures and Tables

**Table 1:** Result of 6 variants of bundle block adjustment in Pix4Dmapper.

Parameters	V1	V2	V3	V4	V5	V6
No. of GCPs	8	10	10	8	10	11
No. of ChPs	210	208	208	210	208	207
RMSE(X) on GCPs [m]	0.011	0.011	0.011	0.009	0.010	0.010
RMSE(Y) on GCPs [m]	0.016	0.022	0.018	0.023	0.023	0.022
RMSE(Z) on GCPs [m]	0.022	0.035	0.030	0.025	0.032	0.033
RMSE(X) on ChPs [m]	0.025	0.024	0.025	0.022	0.022	0.023
RMSE(Y) on ChPs [m]	0.025	0.026	0.025	0.026	0.026	0.026
RMSE(Z) on ChPs [m]	0.048	0.037	0.040	0.049	0.037	0.036

**Table 2:** Analysis of N_ΔZ_ in the ΔZ deviation intervals obtained from the BBA.

Parameters	N_ΔZ_ V1	N_ΔZ_ V2	N_ΔZ_ V3	N_ΔZ_ V4	N_ΔZ_ V5	N_ΔZ_ V6
No. of ChPs	210	208	208	210	208	207
[ΔZ] ≤ 0.05 m	163	185	176	164	182	187
0.05 < [ΔZ] ≤ 0.08 m	33	16	22	33	19	14
0.08 < [ΔZ] ≤ 0.10 m	5	3	4	4	3	2
[ΔZ] > 0.10 m	9	4	6	9	4	4
[%] of [ΔZ] > 0.05 m	22.4%	11.1%	15.4%	21.9%	12.5%	9.7%

**Table 3:** Results of the variant 6 of the BBA in Pix4Dmapper and PhotoScan.

Parameters	V6—Pix4Dmapper	V6—PhotoScan
No. of GCPs	11	11
No. of ChPs	207	207
RMSE(X) on GCPs [m]	0.010	0.019
RMSE(Y) on GCPs [m]	0.022	0.027
RMSE(Z) on GCPs [m]	0.033	0.033
RMSE(X) on ChPs [m]	0.023	0.025
RMSE(Y) on ChPs [m]	0.026	0.024
RMSE(Z) on ChPs [m]	0.036	0.040

**Table 4:** Analysis of N_ΔZ_ (variant 6) calculated in the BBA in Pix4Dmapper and PhotoScan.

Parameters	N_ΔZ_ V6—Pix4Dmapper	N_ΔZ_ V6—PhotoScan
No. of ChPs	207	207
[ΔZ] ≤ 0.05 m	187	169
0.050 < [ΔZ] ≤ 0.08 m	14	28
0.080 < [ΔZ] ≤ 0.10 m	2	4
[ΔZ] > 0.10 m	4	4
[%] of [ΔZ] > 0.05 m	9.7%	17.4%

**Table 5:** Results of the BBA with different image overlap and exterior orientation parameters of unmanned aerial vehicle position from Global Navigation Satellite Systems receiver (UAV GNSS) included in the adjustment.

Parameters	V6_85-85_ incl. UAV GNSS	V6_85-45_ incl. UAV GNSS	V6_65-65_ incl. UAV GNSS	V6_65-45_ incl. UAV GNSS
No. of images	858	293	205	138
No. of GCPs	11	11	11	11
No. of ChPs	207	207	207	207
RMSE(X) on GCPs [m]	0.010	0.016	0.010	0.012
RMSE(Y) on GCPs [m]	0.022	0.022	0.019	0.014
RMSE(Z) on GCPs [m]	0.033	0.028	0.020	0.024
RMSE(X) on ChPs [m]	0.023	0.029	0.024	0.037
RMSE(Y) on ChPs [m]	0.026	0.031	0.029	0.034
RMSE(Z) on ChPs [m]	0.036	0.048	0.046	0.055
[ΔZ] ≤ 0.05 m	187	155	159	134
0.050 < [ΔZ] ≤ 0.08 m	14	33	31	49
0.080 < [ΔZ] ≤ 0.10 m	2	10	9	10
[ΔZ] > 0.10 m	4	9	8	14
[%] of [ΔZ] > 0.05 m	9.7	25.1	23.2	35.3

**Table 6:** Results of the BBA with different image overlap and exterior orientation parameters excluded in the adjustment.

Parameters	V6_85-85_ excl. UAV GNSS	V6_85-45_ excl. UAV GNSS	V6_65-65_ excl. UAV GNSS	V6_65-45_ excl. UAV GNSS
No. of images	858	293	205	138
No. of GCPs	11	11	11	11
No. of ChPs	207	207	207	207
RMSE(X) on GCPs [m]	0.010	0.015	0.010	0.012
RMSE(Y) on GCPs [m]	0.022	0.022	0.019	0.016
RMSE(Z) on GCPs [m]	0.029	0.027	0.019	0.023
RMSE(X) on ChPs [m]	0.023	0.029	0.024	0.038
RMSE(Y) on ChPs [m]	0.026	0.031	0.029	0.035
RMSE(Z) on ChPs [m]	0.036	0.047	0.047	0.056
[ΔZ] ≤ 0.05 m	182	156	154	133
0.050 < [ΔZ] ≤ 0.08 m	18	32	32	42
0.080 < [ΔZ] ≤ 0.10 m	2	10	13	18
[ΔZ] > 0.10 m	5	9	8	14
[%] of [ΔZ] > 0.05 m	12.1	24.6	25.6	35.7

**Table 7:** Length analysis of deviation vectors D.

Parameters	V6—Pix4Dmapper
No. of vectors D_GNSS-BBA_ > 0.10 m (218 ChPs)	3
No. of vectors D_GNSS-OM_ > 0.10 m (218 ChPs)	50
No. of vectors D_GNSS-OM_ > 0.10 m (82 ChPs)	11
No. of vectors D_GNSS-OM_ > 0.10 m (300 ChPs)	61
[%] of vectors D_GNSS-BBA_ > 0.10 m (218 ChPs)	1.4
[%] of vectors D_GNSS-OM_ > 0.10 m (218 ChPs)	22.9
[%] of vectors D_GNSS-OM_ > 0.10 m (82 ChPs)	13.4
[%] of vectors D_GNSS-OM_ > 0.10 m (300 ChPs)	20.3
RMS(D_GNSS-BBA_) (218 ChPs) [m]	0.034
RMS(D_GNSS-OM_) (218 ChPs) [m]	0.084
RMS(D_GNSS-OM_) (82 ChPs) [m]	0.076
RMS(D_GNSS-OM_) (300 ChPs) [m]	0.082

**Table 8:** Analysis of direction of deviation vectors D.

Vector Direction	↗	↖	↙	↘
No. of vectors D_GNSS-BBA_ (218 ChPs)	69	53	54	42
No. of vectors D_GNSS-OM_ (218 ChPs)	6	195	15	2
No. of vectors D_GNSS-OM_ (82 ChPs)	3	67	10	2
No. of vectors D_GNSS-OM_ (300 ChPs)	9	262	25	4

## Supplementary Materials

The following are available online at https://www.mdpi.com/1424-8220/19/23/5229/s1.
