# Supplementary material for: Multi-Variant Accuracy Evaluation of UAV Imaging Surveys: A Case Study on Investment Area
Source: Sensors (Basel). 2019 Nov 28;19(23):5229. doi: 10.3390/s19235229 (PMC6929115; doi:10.3390/s19235229)
Supplement: Supplementary file 1 [file sensors-19-05229-s001.zip › supplementary_files/App10_V6_p65q65_GPS_report.pdf]

Quality Report

Generated with Pix4Ddiscovery version 4.3.31

Important: Click on the different icons for:

Help to analyze the results in the Quality Report

Additional information about the sections

Click here for additional tips to analyze the Quality Report

Summary

Project	V6_p65q65_GPS
Processed	2019-10-12 23:41:06
Camera Model Name(s)	NEX-5T_0.0_4912x3264 (RGB)
Average Ground Sampling Distance (GSD)	4.55 cm / 1.79 in
Time for Initial Processing (without report)	20m:27s

Quality Check

**Images**	median of 45246 keypoints per image	
**Dataset**	205 out of 205 images calibrated (100%), all images enabled	
**Camera Optimization**	0.12% relative difference between initial and optimized internal camera parameters	
**Matching**	median of 21815.5 matches per calibrated image	
**Georeferencing**	yes, 11 GCPs (11 3D), mean RMS error = 0.016 m	

Calibration Details

Number of Calibrated Images	205 out of 205
Number of Geolocated Images	205 out of 205

Initial Image Positions

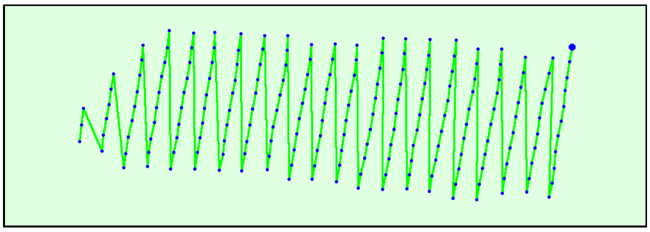

Figure 2: Top view of the initial image position. The green line follows the position of the images in time starting from the large blue dot.

Computed Image/GCPs/Manual Tie Points Positions

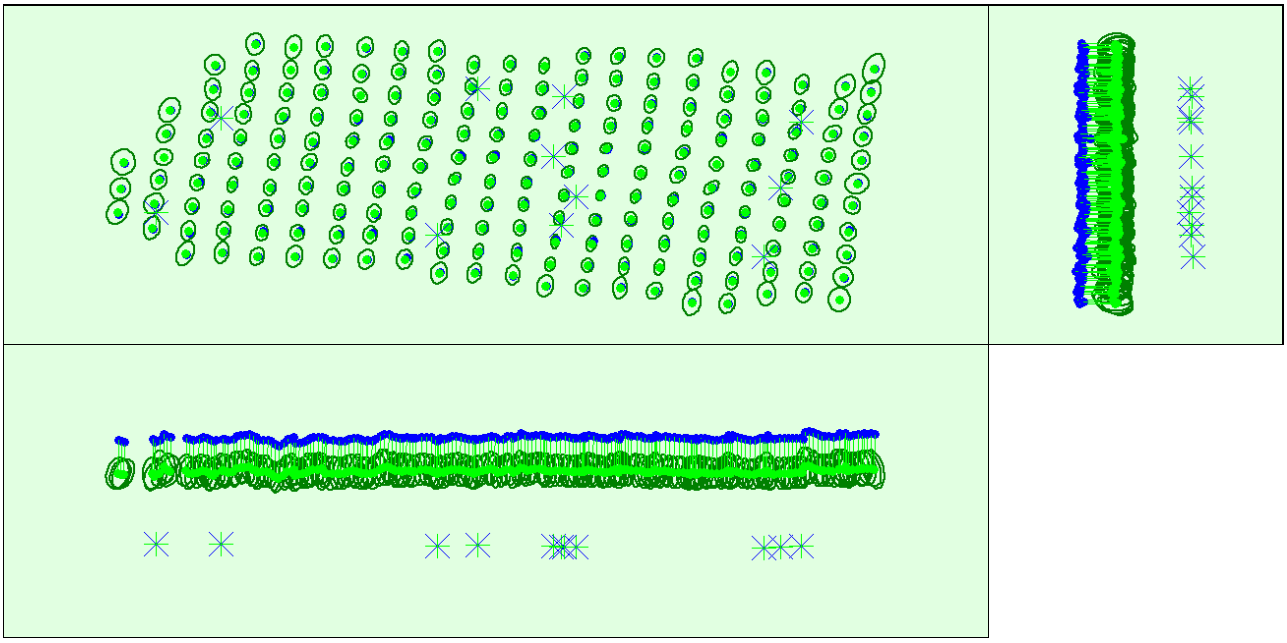

Uncertainty ellipses 1000x magnified

Figure 3: Offset between initial (blue dots) and computed (green dots) image positions as well as the offset between the GCPs initial positions (blue crosses) and their computed positions (green crosses) in the top-view (XY plane), front-view (XZ plane), and side-view (YZ plane). Dark green ellipses indicate the absolute position uncertainty of the bundle block adjustment result.

Absolute camera position and orientation uncertainties

	X [m]	Y [m]	Z [m]	Omega [degree]	Phi [degree]	Kappa [degree]
Mean	0.014	0.017	0.031	0.005	0.005	0.002
Sigma	0.003	0.003	0.001	0.001	0.001	0.001

Bundle Block Adjustment Details

Number of 2D Keypoint Observations for Bundle Block Adjustment	4560025
Number of 3D Points for Bundle Block Adjustment	1387826
Mean Reprojection Error [pixels]	0.230

Internal Camera Parameters

NEX-5T_0.0_4912x3264 (RGB). Sensor Dimensions: 23.400 [mm] x 15.549 [mm]

EXIF ID: NEX-5T_0.0_4912x3264

	Focal Length	Principal Point x	Principal Point y	R1	R2	R3	T1	T2
Initial Values	3256.554 [pixel] 15.514 [mm]	2456.002 [pixel] 11.700 [mm]	1632.003 [pixel] 7.775 [mm]	-0.043	0.028	-0.005	0.000	-0.001
Optimized Values	3252.643 [pixel] 15.495 [mm]	2465.688 [pixel] 11.746 [mm]	1586.781 [pixel] 7.559 [mm]	-0.047	0.035	-0.009	0.000	0.000
Uncertainties (Sigma)	0.674 [pixel] 0.003 [mm]	0.137 [pixel] 0.001 [mm]	0.142 [pixel] 0.001 [mm]	0.000	0.000	0.000	0.000	0.000

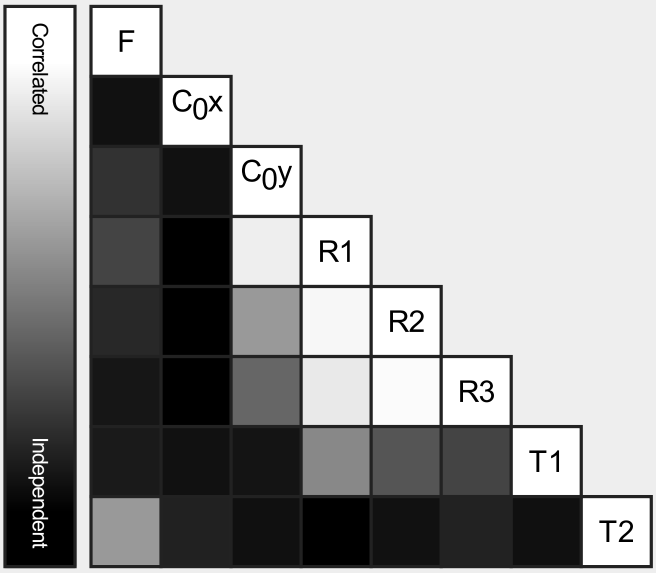

The correlation between camera internal parameters determined by the bundle adjustment. White indicates a full correlation between the parameters, ie. any change in one can be fully compensated by the other. Black indicates that the parameter is completely independent, and is not affected by other parameters.

The number of Automatic Tie Points (ATPs) per pixel, averaged over all images of the camera model, is color coded between black and white. White indicates that, on average, more than 16 ATPs have been extracted at the pixel location. Black indicates that, on average, 0 ATPs have been extracted at the pixel location. Click on the image to the see the average direction and magnitude of the re-projection error for each pixel. Note that the vectors are scaled for better visualization. The scale bar indicates the magnitude of 1 pixel error.

2D Keypoints Table

	Number of 2D Keypoints per Image	Number of Matched 2D Keypoints per Image
Median	45246	21816
Min	20002	7592
Max	65940	43798
Mean	42585	22244

3D Points from 2D Keypoint Matches

	Number of 3D Points Observed
In 2 Images	698070
In 3 Images	268889
In 4 Images	150435
In 5 Images	97194
In 6 Images	66352
In 7 Images	43729
In 8 Images	28664
In 9 Images	18085
In 10 Images	9932
In 11 Images	4403
In 12 Images	1592
In 13 Images	386
In 14 Images	84
In 15 Images	11

2D Keypoint Matches

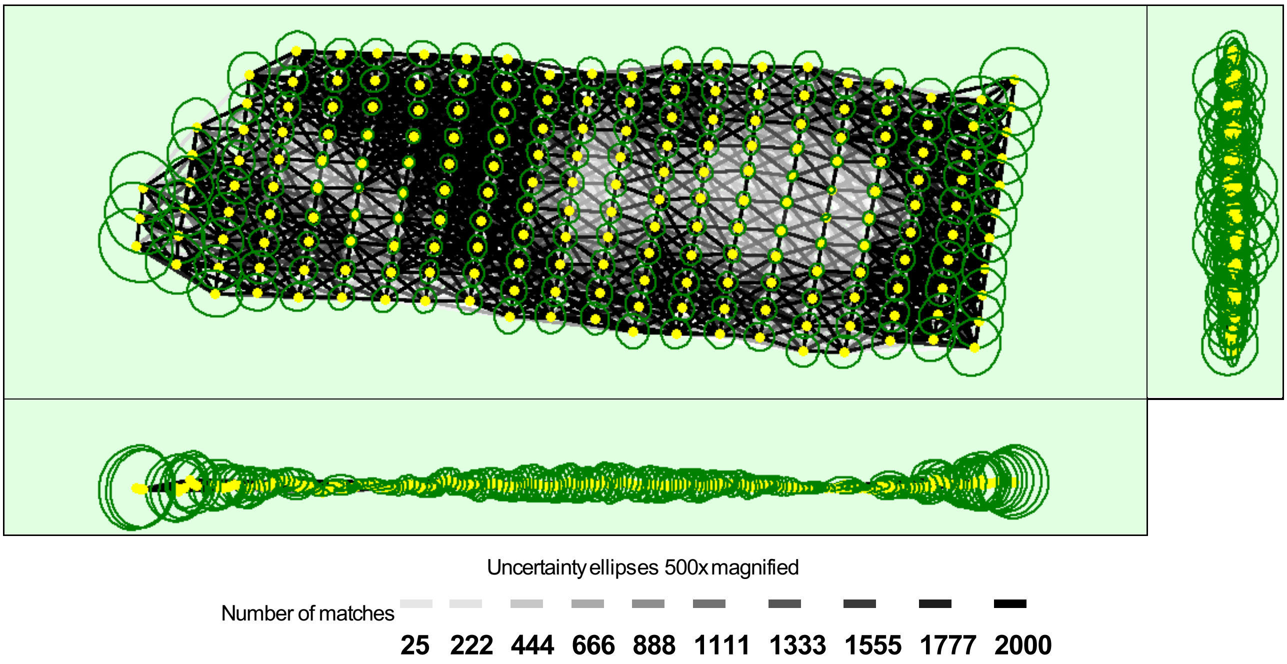

Figure 5: Computed image positions with links between matched images. The darkness of the links indicates the number of matched 2D keypoints between the images. Bright links indicate weak links and require manual tie points or more images. Dark green ellipses indicate the relative camera position uncertainty of the bundle block adjustment result.

Relative camera position and orientation uncertainties

	X[m]	Y[m]	Z[m]	Omega [degree]	Phi [degree]	Kappa [degree]
Mean	0.046	0.048	0.046	0.013	0.013	0.012
Sigma	0.021	0.021	0.027	0.005	0.005	0.006

Geolocation Details

Ground Control Points

GCP Name	Accuracy XY/Z [m]	Error X[m]	Error Y[m]	Error Z[m]	Projection Error [pixel]	Verified/Marked
201 (3D)	0.030/ 0.050	0.003	-0.020	0.020	0.566	8 / 8
202 (3D)	0.030/ 0.050	-0.007	0.012	-0.007	0.561	8 / 8
203 (3D)	0.030/ 0.050	-0.000	0.016	-0.009	0.476	10 / 10
208 (3D)	0.030/ 0.050	-0.016	-0.013	-0.011	0.512	9 / 9
209 (3D)	0.030/ 0.050	0.014	-0.003	-0.017	0.359	11 / 11
207 (3D)	0.030/ 0.050	-0.006	-0.016	0.051	0.298	8 / 8
211 (3D)	0.030/ 0.050	0.002	0.029	0.009	0.490	10 / 10
210 (3D)	0.030/ 0.050	-0.007	-0.016	0.005	0.338	11 / 11
206 (3D)	0.030/ 0.050	0.017	0.031	-0.001	0.264	9 / 9
205 (3D)	0.030/ 0.050	-0.012	-0.025	0.002	0.834	8 / 8
204 (3D)	0.030/ 0.050	0.008	0.004	-0.028	0.474	8 / 8
**Mean [m]**		-0.000386	-0.000166	0.001300		
**Sigma [m]**		0.010135	0.018901	0.020093		
**RMS Error [m]**		0.010142	0.018902	0.020135		

0 out of 207 check points have been labeled as inaccurate.

Check Point Name	Accuracy XY/Z [m]	Error X[m]	Error Y[m]	Error Z[m]	Projection Error [pixel]	Verified/Marked
103		0.0099	0.0324	-0.0177	0.3334	11 / 11
101		-0.0232	0.0134	0.0433	0.2960	10 / 10
100		-0.0117	-0.0047	0.0458	0.2828	11 / 11
104		-0.0550	-0.0007	0.0358	0.2149	8 / 8

102		-0.0176	-0.0051	0.0205	0.3772	11 / 11
k1		0.0496	-0.0586	0.1156	0.4079	8 / 8
k2		0.0051	0.0218	-0.0197	0.5140	10 / 10
k3		0.0932	-0.0813	0.1500	0.4614	6 / 6
k4		0.0411	-0.0335	0.0394	0.3557	7 / 7
k5		-0.0186	0.0265	-0.0567	0.4608	7 / 7
k6		-0.0214	0.0134	0.0955	0.9128	8 / 8
k7		-0.0730	0.0055	0.1792	0.6096	7 / 7
k8		-0.0270	-0.0379	0.1079	0.4628	8 / 8
k11		0.0028	-0.0151	-0.0418	0.4285	10 / 10
k13		0.0006	-0.0025	-0.0220	0.3369	9 / 9
k14		-0.0015	-0.0222	-0.0218	0.4293	8 / 8
10000		-0.0291	-0.0093	0.0834	0.5991	7 / 7
10001		0.0140	-0.0182	0.0366	0.3700	8 / 8
10002		0.0102	-0.0282	-0.0038	0.2555	9 / 9
10003		0.0105	-0.0315	-0.0294	0.3247	10 / 10
10004		0.0252	0.0011	-0.0082	0.4224	10 / 10
10005		0.0370	-0.0058	-0.0225	0.3623	11 / 11
10006		0.0167	-0.0118	-0.0396	0.3632	11 / 11
10007		0.0099	-0.0034	-0.0225	0.4046	9 / 9
10008		0.0224	-0.0185	-0.0275	0.3551	7 / 7
10009		0.0142	-0.0291	-0.0035	0.4374	11 / 11
10010		0.0253	-0.0227	-0.0249	0.3783	11 / 11
10011		0.0097	-0.0467	-0.0380	0.4083	9 / 9
10012		-0.0288	0.0035	0.0153	0.3501	11 / 11
10013		-0.0257	0.0137	0.0255	0.4610	10 / 10
10014		0.0004	0.0165	0.0157	0.3878	10 / 10
10015		0.0218	0.0094	0.0495	0.3709	10 / 10
10016		-0.0079	0.0087	0.0173	0.5273	12 / 12
10017		-0.0004	-0.0052	-0.0184	0.6613	11 / 11
10018		-0.0216	0.0058	0.0165	0.2783	6 / 6
10019		-0.0268	0.0227	0.0497	0.2615	10 / 10
10020		-0.0218	0.0340	0.0095	0.5404	8 / 8
10021		0.0021	0.0008	0.0015	0.3495	9 / 9
10022		0.0107	0.0367	-0.0019	0.3451	10 / 10
10023		0.0213	-0.0011	-0.0314	0.4253	9 / 9
10024		-0.0286	0.0032	-0.0716	0.3402	11 / 11
10025		-0.0049	0.0070	-0.0341	0.5236	10 / 10
10026		-0.0205	0.0167	-0.0400	0.2688	8 / 8
10027		-0.0154	0.0094	-0.0299	0.3902	10 / 10
10028		0.0002	0.0011	-0.0174	0.3839	11 / 11
10030		0.0011	0.0301	-0.0268	0.3732	11 / 11
10031		0.0282	0.0385	0.0008	0.4200	7 / 7
10032		0.0423	0.0306	-0.0270	0.3425	7 / 7
10033		0.0067	0.0393	0.0065	0.3461	11 / 11
10034		0.0276	-0.0017	-0.0299	0.4216	11 / 11
10035		0.0177	-0.0194	0.0220	0.5477	9 / 9
10036		0.0215	0.0101	0.0013	0.3949	6 / 6
10037		-0.0031	-0.0093	-0.0149	0.3433	9 / 9
10038		-0.0138	0.0119	-0.0047	0.2768	10 / 10
10039		0.0009	-0.0123	-0.0282	0.3454	10 / 10
10040		-0.0250	0.0062	0.0022	0.5188	8 / 8
10041		-0.0331	-0.0157	0.0502	0.9516	9 / 9
10044		0.0116	-0.0278	-0.0357	0.2810	9 / 9
10045		0.0227	-0.0160	-0.0877	0.3433	9 / 9
10046		-0.0147	0.0083	-0.0706	0.3531	10 / 10
10047		-0.0349	-0.0032	0.0864	0.8906	9 / 9
10048		0.0012	-0.0370	0.0363	0.4588	10 / 10
10049		-0.0227	0.0508	-0.0193	0.8450	9 / 9

10051		0.0042	0.0148	-0.0122	0.3916	11 / 11
10052		0.0113	-0.0124	-0.0031	0.2742	7 / 7
10053		-0.0024	0.0182	0.0068	0.3748	10 / 10
10054		0.0026	-0.0234	-0.0031	0.3485	10 / 10
10055		-0.0209	0.0125	-0.0273	0.3454	8 / 8
10056		-0.0466	0.0164	-0.0067	0.3227	10 / 10
10058		-0.0779	0.0930	0.0376	0.4720	11 / 11
10059		-0.0089	-0.0080	-0.0591	0.3655	11 / 11
10060		-0.0252	-0.0160	-0.0107	0.3628	11 / 11
10061		0.0110	0.0260	0.0380	0.4398	7 / 7
10062		0.0028	-0.0279	0.0251	0.3138	10 / 10
10063		0.0153	-0.0361	-0.0107	0.5673	10 / 10
10064		0.0412	0.0277	-0.0342	0.3587	11 / 11
10065		0.0235	0.0441	-0.0260	0.4005	11 / 11
10066		0.0521	0.0429	-0.0428	0.4864	13 / 13
10067		0.0267	0.0414	-0.0198	0.3080	12 / 12
10068		0.0113	0.1194	-0.0445	0.4659	12 / 12
10074		-0.0180	0.0505	-0.0636	0.5695	11 / 11
10075		-0.0145	0.0586	0.0023	0.3824	10 / 10
10076		0.0039	0.0325	-0.0208	0.4050	10 / 10
10077		-0.0271	0.0593	-0.0239	0.7669	10 / 10
10078		0.0196	0.0129	-0.0140	0.3705	10 / 10
10080		0.0321	-0.0722	-0.1037	0.3736	9 / 9
10081		-0.0222	-0.0143	-0.0098	0.3548	10 / 10
10082		0.0186	-0.0420	-0.0322	0.3329	11 / 11
10083		0.0464	-0.0048	-0.1239	0.5224	11 / 11
10085		0.0382	-0.0311	0.0124	0.3681	12 / 12
15000		0.0112	-0.0130	-0.0630	0.3165	12 / 12
15001		-0.0240	-0.0278	-0.0573	0.3412	11 / 11
15002		0.0187	0.0141	-0.0746	0.3000	10 / 10
15003		-0.0028	0.0376	0.0007	0.4852	12 / 12
15004		-0.0132	0.0262	-0.0264	0.3470	12 / 12
15005		-0.0078	0.0217	0.0038	0.4668	9 / 9
15006		-0.0078	0.0342	-0.0091	0.5369	11 / 11
15007		-0.0025	-0.0039	0.0300	0.4436	8 / 8
15008		-0.0178	0.0101	0.0656	0.9455	10 / 10
15009		-0.0125	-0.0089	0.0286	0.3373	9 / 9
15010		0.0201	0.0103	0.0211	0.2366	9 / 9
20000		-0.0054	0.0095	0.0425	0.4980	10 / 10
20001		-0.0143	-0.0081	0.0350	0.4713	9 / 9
20002		0.0040	-0.0005	0.0112	0.4024	9 / 9
20003		-0.0121	-0.0202	-0.0245	0.3252	8 / 8
20004		0.0106	-0.0225	-0.0054	0.2477	8 / 8
20005		-0.0571	-0.0218	0.0183	0.4513	9 / 9
20008		0.0044	0.0043	0.0038	0.5378	12 / 12
20009		0.0281	0.0033	0.0025	0.3981	10 / 10
20010		-0.0196	0.0280	-0.0296	0.3421	12 / 12
20011		-0.0184	-0.0016	-0.0386	0.4391	12 / 12
20012		0.0101	0.0111	-0.0415	0.2927	11 / 11
20013		0.0084	0.0158	-0.0355	0.3838	10 / 10
20014		0.0027	0.0190	-0.0336	0.4427	11 / 11
20016		-0.0163	0.0296	-0.0799	0.2686	10 / 10
20017		-0.0023	0.0258	-0.0580	0.3032	8 / 8
20018		0.0133	0.0317	-0.0654	0.3277	12 / 12
20019		0.0160	0.0233	-0.0579	0.3764	13 / 13
20020		0.0140	0.0380	-0.0012	0.5062	11 / 11
20021		0.0057	0.0162	-0.0144	0.2843	10 / 10
20022		-0.0154	-0.0330	-0.0165	0.3120	10 / 10
20023		0.0050	0.0249	-0.0161	0.3531	13 / 13

20024		-0.0045	0.0560	-0.0426	0.4565	10 / 10
20025		0.0236	0.0578	-0.0371	0.2733	10 / 10
20026		0.0280	0.0537	-0.0197	0.4554	11 / 11
20027		0.0041	0.0751	-0.0190	0.4085	11 / 11
20028		-0.0054	0.0106	-0.0034	0.3558	12 / 12
20029		0.0197	-0.0262	-0.0171	0.6876	9 / 9
20031		-0.0207	0.0272	-0.0259	0.5212	10 / 10
25000		0.0091	0.0177	-0.0198	0.2543	9 / 9
25001		-0.0060	0.0129	0.0338	0.2946	10 / 10
25003		0.0079	-0.0068	0.0233	0.3893	10 / 10
25004		-0.0011	0.0196	-0.0094	0.3894	10 / 10
25005		0.0034	0.0084	0.0143	0.2343	9 / 9
25006		-0.0232	-0.0158	-0.0784	0.3993	10 / 10
25008		-0.0342	0.0259	-0.0438	0.2530	10 / 10
25009		0.0279	-0.0287	-0.0468	0.2495	11 / 11
25010		0.0025	0.0085	-0.0518	0.2332	11 / 11
25013		0.0022	0.0265	-0.0169	0.4307	10 / 10
25014		-0.0090	-0.0028	-0.0309	0.3500	10 / 10
25015		-0.0179	-0.0001	-0.0331	0.3848	11 / 11
25016		-0.0218	-0.0015	-0.0305	0.2749	11 / 11
25017		0.0185	-0.0108	0.0460	0.6079	10 / 10
25018		0.0055	0.0002	0.0298	0.3586	9 / 9
25019		-0.0112	-0.0102	0.0168	0.3229	9 / 9
25020		-0.0078	0.0133	-0.0096	0.3647	6 / 6
25021		0.0184	0.0214	-0.0280	0.3960	9 / 9
25022		0.0156	0.0164	-0.0036	0.6621	9 / 9
25023		-0.0040	0.0273	0.0019	0.3217	8 / 8
25024		0.0058	-0.0038	-0.0343	0.3649	11 / 11
25029		0.0182	0.0083	0.0097	0.3956	10 / 10
25030		-0.0109	0.0069	0.0244	0.3045	11 / 11
25032		-0.0667	-0.0154	-0.0327	0.4039	11 / 11
25033		-0.0137	-0.0122	0.0101	0.4737	10 / 10
25034		-0.0043	-0.0125	-0.0062	0.3655	9 / 9
40000		-0.0027	-0.0107	-0.1010	0.3681	12 / 12
40001		0.0217	0.0357	-0.0120	0.3240	10 / 10
40002		0.0074	0.0572	-0.0445	0.3122	12 / 12
40003		0.0042	0.0665	-0.0406	0.4799	9 / 9
40004		0.0057	0.0251	-0.0372	0.2711	10 / 10
40005		0.0144	0.0468	-0.0316	0.3555	7 / 7
45000		0.0432	-0.0300	0.0108	0.4046	10 / 10
45001		0.0014	-0.0021	0.0018	0.2683	9 / 9
45002		0.0230	-0.0354	0.0098	0.4652	11 / 11
10043		-0.0220	-0.0089	0.0367	0.3798	8 / 8
10084		0.0030	-0.0032	0.0247	0.3750	7 / 7
20015		-0.0153	0.0046	-0.0763	0.3224	11 / 11
20007		-0.0024	0.0071	0.0020	0.3172	4 / 4
25027		-0.0098	-0.0001	-0.0512	0.2943	9 / 9
k9		-0.0085	-0.0078	-0.0167	0.4340	11 / 11
6600		-0.0020	-0.0080	-0.0484	0.4139	12 / 12
6602		0.0157	0.0421	-0.0384	0.3784	11 / 11
6603		0.0035	-0.0174	-0.0123	0.4445	10 / 10
6605		0.0162	0.0047	0.0040	0.4495	9 / 9
6606		0.0175	0.0221	-0.0446	0.4824	7 / 7
6607		0.0129	0.0195	-0.0488	0.5169	11 / 11
6608		-0.0101	-0.0502	-0.0998	0.3952	3 / 3
6609		-0.0188	0.0095	0.0043	0.3428	8 / 8
6610		-0.0012	0.0031	0.0236	0.6707	7 / 7
6611		-0.0448	-0.0201	-0.0562	0.3482	9 / 9
6612		-0.0242	-0.0440	-0.0911	0.5076	8 / 8

6616		-0.0020	-0.0122	-0.0712	0.4213	8 / 8
6617		-0.0200	0.0137	0.0530	0.3342	4 / 4
6618		-0.0098	0.0007	-0.0092	0.5010	10 / 10
6619		0.0144	0.0090	0.0220	0.2763	10 / 10
6620		0.0265	-0.0378	0.0101	0.5035	9 / 9
6621		-0.0136	0.0035	0.0086	0.3373	11 / 11
6622		-0.0191	0.0126	0.0257	0.4753	9 / 9
6623		-0.0163	-0.0158	0.0014	0.5597	10 / 10
6624		0.0216	-0.0158	-0.0347	0.7329	6 / 6
6625		-0.0296	-0.0410	-0.0442	0.4307	8 / 8
6626		-0.0274	-0.0197	-0.1038	0.4301	9 / 9
6627		-0.0288	-0.0443	-0.0513	0.4332	8 / 8
6628		-0.0314	-0.0632	-0.0739	0.2900	9 / 9
6629		-0.0455	-0.0514	-0.0784	0.3747	9 / 9
6630		-0.0582	-0.0319	-0.0852	0.3196	8 / 8
6631		-0.0272	-0.0463	-0.0667	0.4523	7 / 7
6632		-0.0549	-0.0317	-0.0705	0.6443	9 / 9
6633		-0.0212	-0.0279	-0.0867	0.5387	8 / 8
6634		-0.0212	-0.0153	-0.0839	0.2521	8 / 8
6635		-0.0025	-0.0026	-0.0599	0.4283	9 / 9
6636		-0.0477	-0.0547	-0.0710	0.4961	10 / 10
6637		-0.0134	-0.0387	-0.0692	0.5124	8 / 8
6638		-0.0241	-0.0281	-0.0644	0.2780	7 / 7
6639		-0.0539	-0.0224	-0.0531	0.4453	10 / 10
6640		-0.0471	-0.0335	-0.0555	0.3911	8 / 8
6641		-0.0431	-0.0289	-0.0426	0.5493	10 / 10
**Mean [m]**		-0.002858	0.001825	-0.014509		
**Sigma [m]**		0.024073	0.029107	0.043872		
**RMS Error [m]**		0.024242	0.029165	0.046209		

Localisation accuracy per GCP and mean errors in the three coordinate directions. The last column counts the number of calibrated images where the GCP has been automatically verified vs. manually marked.

Absolute Geolocation Variance

Min Error [m]	Max Error [m]	Geolocation Error X [%]	Geolocation Error Y [%]	Geolocation Error Z [%]
-	-15.00	0.00	0.00	0.00
-15.00	-12.00	0.00	0.00	0.00
-12.00	-9.00	0.00	0.00	0.00
-9.00	-6.00	0.00	0.00	0.00
-6.00	-3.00	0.00	0.98	5.85
-3.00	0.00	50.73	52.68	41.46
0.00	3.00	49.27	42.93	47.80
3.00	6.00	0.00	3.41	4.88
6.00	9.00	0.00	0.00	0.00
9.00	12.00	0.00	0.00	0.00
12.00	15.00	0.00	0.00	0.00
15.00	-	0.00	0.00	0.00
**Mean [m]**		1.764221	0.356890	65.757635
**Sigma [m]**		0.381525	1.354293	1.953607
**RMS Error [m]**		1.805003	1.400529	65.786648

Min Error and Max Error represent geolocation error intervals between -1.5 and 1.5 times the maximum accuracy of all the images. Columns X, Y, Z show the percentage of images with geolocation errors within the predefined error intervals. The geolocation error is the difference between the initial and computed image positions. Note that the image geolocation errors do not correspond to the accuracy of the observed 3D points.

Geolocation Bias	X	Y	Z
Translation [m]	1.764221	0.356890	65.757635

Bias between image initial and computed geolocation given in output coordinate system.

Relative Geolocation Variance

Relative Geolocation Error	Images X [%]	Images Y [%]	Images Z [%]
[-1.00, 1.00]	100.00	100.00	100.00
[-2.00, 2.00]	100.00	100.00	100.00
[-3.00, 3.00]	100.00	100.00	100.00
**Mean of Geolocation Accuracy [m]**	5.000000	5.000000	10.000000
**Sigma of Geolocation Accuracy [m]**	0.000000	0.000000	0.000000

Images X, Y, Z represent the percentage of images with a relative geolocation error in X, Y, Z.

Initial Processing Details

System Information

Hardware	CPU: Intel(R) Core(TM) i9-7940X CPU @ 3.10GHz<br>RAM: 128GB<br>GPU: NVIDIA GeForce GTX 1080 Ti (Driver: 26.21.14.3615), RDPDD Chained DD (Driver: unknown), RDP Encoder Mirror Driver (Driver: unknown), RDP Reflector Display Driver (Driver: unknown)
Operating System	Windows 7 Ultimate, 64-bit

Coordinate Systems

Image Coordinate System	WGS 84 (EGM96 Geoid)
Ground Control Point (GCP) Coordinate System	ETRS89 / Poland CS2000 zone 6
Output Coordinate System	ETRS89 / Poland CS2000 zone 6

Processing Options

Detected Template	No Template Available
Keypoints Image Scale	Full, Image Scale: 1
Advanced: Matching Image Pairs	Aerial Grid or Corridor
Advanced: Matching Strategy	Use Geometrically Verified Matching: yes
Advanced: Keypoint Extraction	Targeted Number of Keypoints: Automatic
Advanced: Calibration	Calibration Method: Standard<br>Internal Parameters Optimization: All<br>External Parameters Optimization: All<br>Rematch: Auto, yes
