# Supplementary material for: Multi-Variant Accuracy Evaluation of UAV Imaging Surveys: A Case Study on Investment Area
Source: Sensors (Basel). 2019 Nov 28;19(23):5229. doi: 10.3390/s19235229 (PMC6929115; doi:10.3390/s19235229)
Supplement: Supplementary file 1 [file sensors-19-05229-s001.zip › supplementary_files/App11_V6_p65q65_noGPS_report.pdf]

Quality Report

Generated with Pix4Ddiscovery version 4.3.31

Important: Click on the different icons for:

Help to analyze the results in the Quality Report

Additional information about the sections

Click here for additional tips to analyze the Quality Report

Summary

Project	V6_p65q65_noGPS
Processed	2019-10-17 01:00:46
Camera Model Name(s)	NEX-5T_0.0_4912x3264 (RGB)
Average Ground Sampling Distance (GSD)	4.54 cm / 1.79 in
Time for Initial Processing (without report)	16m:24s

Quality Check

**Images**	median of 45246 keypoints per image	✓
**Dataset**	205 out of 205 images calibrated (100%), all images enabled	✓
**Camera Optimization**	0.16% relative difference between initial and optimized internal camera parameters	✓
**Matching**	median of 19844.5 matches per calibrated image	✓
**Georeferencing**	yes, 11 GCPs (11 3D), mean RMS error = 0.016 m	✓

Calibration Details

Number of Calibrated Images	205 out of 205
Number of Geolocated Images	0 out of 205

Initial Image Positions

The preview is not generated for images without geolocation.

Computed Image/GCPs/Manual Tie Points Positions

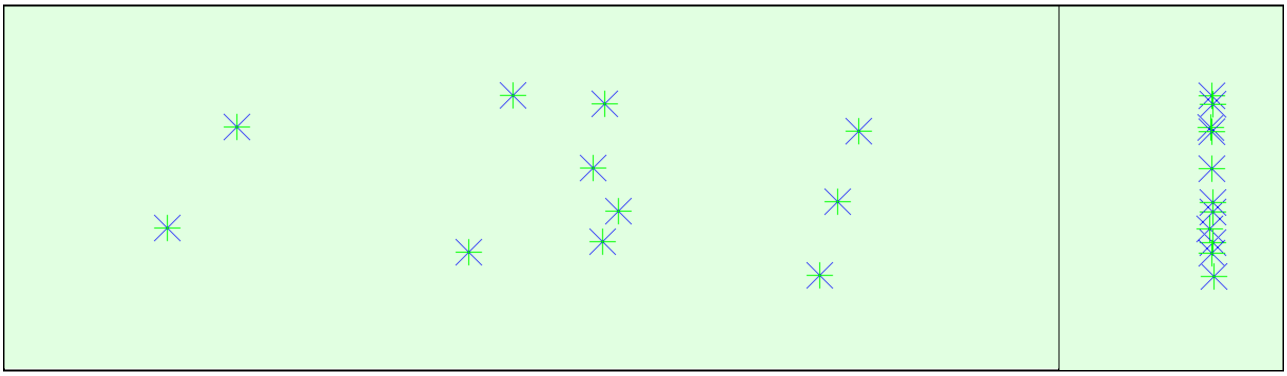

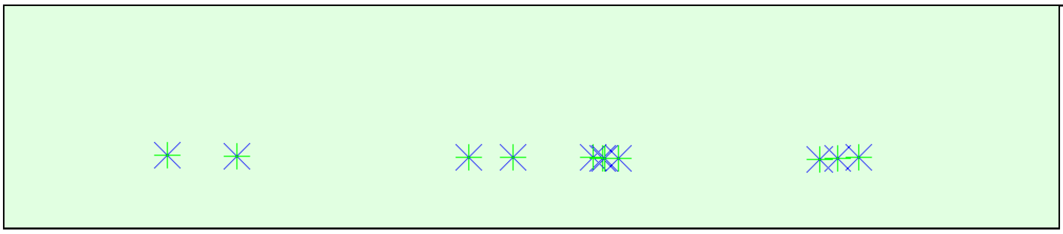

Uncertainty ellipses 1000x magnified

Figure 3: Offset between initial (blue dots) and computed (green dots) image positions as well as the offset between the GCPs initial positions (blue crosses) and their computed positions (green crosses) in the top-view (XY plane), front-view (XZ plane), and side-view (YZ plane). Dark green ellipses indicate the absolute position uncertainty of the bundle block adjustment result.

Absolute camera position and orientation uncertainties

	X [m]	Y [m]	Z [m]	Omega [degree]	Phi [degree]	Kappa [degree]
Mean	0.014	0.016	0.031	0.005	0.005	0.002
Sigma	0.003	0.003	0.001	0.001	0.001	0.001

Bundle Block Adjustment Details

Number of 2D Keypoint Observations for Bundle Block Adjustment	4180622
Number of 3D Points for Bundle Block Adjustment	1381721
Mean Reprojection Error [pixels]	0.210

Internal Camera Parameters

NEX-5T_0.0_4912x3264 (RGB). Sensor Dimensions: 23.400 [mm] x 15.549 [mm]

EXIF ID: NEX-5T_0.0_4912x3264

	Focal Length	Principal Point x	Principal Point y	R1	R2	R3	T1	T2
Initial Values	3256.554 [pixel] 15.514 [mm]	2456.002 [pixel] 11.700 [mm]	1632.003 [pixel] 7.775 [mm]	-0.043	0.028	-0.005	0.000	-0.001
Optimized Values	3251.047 [pixel] 15.487 [mm]	2465.594 [pixel] 11.746 [mm]	1587.047 [pixel] 7.560 [mm]	-0.047	0.035	-0.010	0.000	0.000
Uncertainties (Sigma)	0.669 [pixel] 0.003 [mm]	0.136 [pixel] 0.001 [mm]	0.141 [pixel] 0.001 [mm]	0.000	0.000	0.000	0.000	0.000

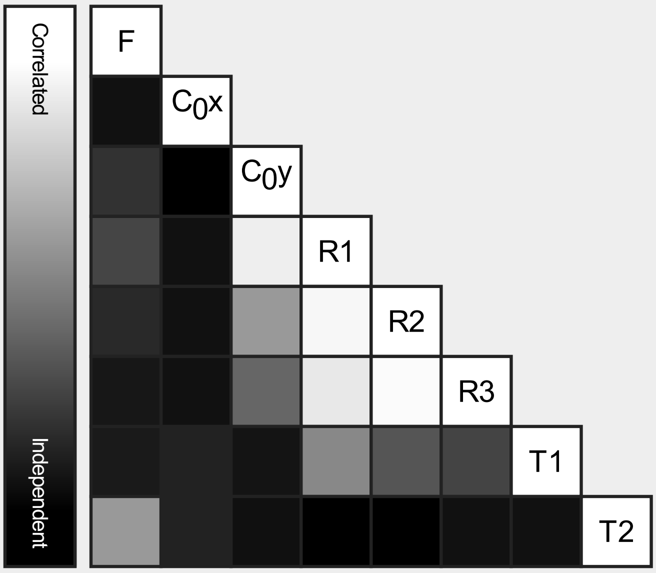

The correlation between camera internal parameters determined by the bundle adjustment. White indicates a full correlation between the parameters, ie. any change in one can be fully compensated by the other. Black indicates that the parameter is completely independent, and is not affected by other parameters.

The number of Automatic Tie Points (ATPs) per pixel, averaged over all images of the camera model, is color coded between black and white. White indicates that, on average, more than 16 ATPs have been extracted at the pixel location. Black indicates that, on average, 0 ATPs have been extracted at the pixel location. Click on the image to the see the average direction and magnitude of the re-projection error for each pixel. Note that the vectors are scaled for better visualization. The scale bar indicates the magnitude of 1 pixel error.

2D Keypoints Table

	Number of 2D Keypoints per Image	Number of Matched 2D Keypoints per Image
Median	45246	19844
Min	20002	6516
Max	65940	41388
Mean	42585	20393

3D Points from 2D Keypoint Matches

	Number of 3D Points Observed
In 2 Images	774867
In 3 Images	272476
In 4 Images	132139
In 5 Images	77924
In 6 Images	51853
In 7 Images	31597
In 8 Images	19466
In 9 Images	11733
In 10 Images	6034
In 11 Images	2546
In 12 Images	854
In 13 Images	187
In 14 Images	38
In 15 Images	7

2D Keypoint Matches

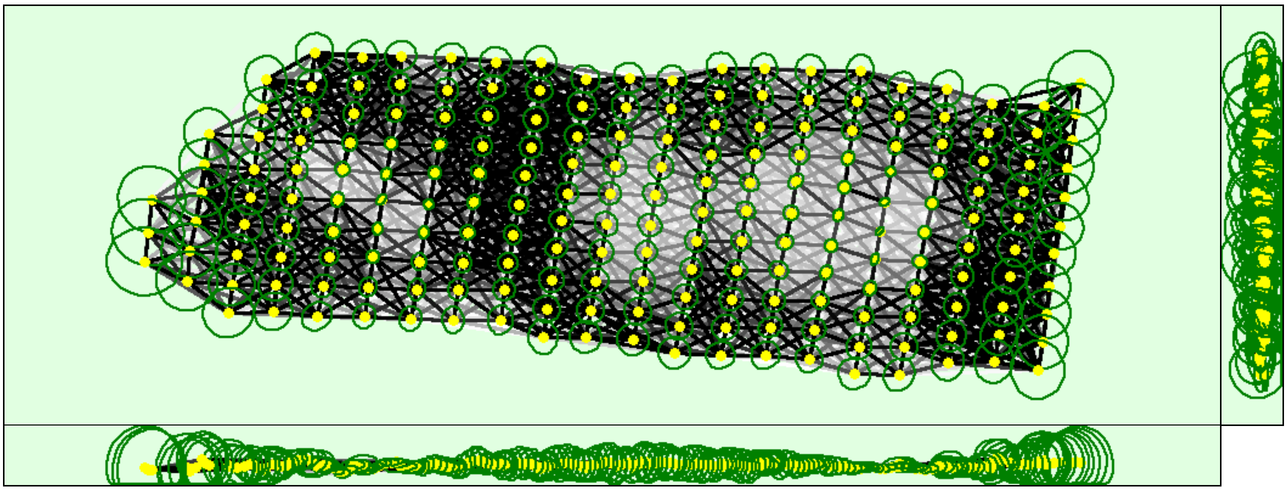

Uncertainty ellipses 500x magnified

Number of matches

25 222 444 666 888 1111 1333 1555 1777 2000

Figure 5: Computed image positions with links between matched images. The darkness of the links indicates the number of matched 2D keypoints between the images. Bright links indicate weak links and require manual tie points or more images. Dark green ellipses indicate the relative camera position uncertainty of the bundle block adjustment result.

Relative camera position and orientation uncertainties

	X [m]	Y [m]	Z [m]	Omega [degree]	Phi [degree]	Kappa [degree]
Mean	0.044	0.044	0.044	0.013	0.012	0.011
Sigma	0.020	0.019	0.026	0.005	0.005	0.005

Geolocation Details

Ground Control Points

GCP Name	Accuracy XY/Z [m]	Error X [m]	Error Y [m]	Error Z [m]	Projection Error [pixel]	Verified/Marked
201 (3D)	0.030/ 0.050	-0.000	-0.021	0.016	0.544	8 / 8
202 (3D)	0.030/ 0.050	-0.005	0.013	-0.006	0.579	8 / 8
203 (3D)	0.030/ 0.050	0.000	0.015	-0.006	0.458	10 / 10
208 (3D)	0.030/ 0.050	-0.016	-0.014	-0.012	0.500	9 / 9
209 (3D)	0.030/ 0.050	0.016	-0.002	-0.019	0.342	11 / 11
207 (3D)	0.030/ 0.050	-0.006	-0.017	0.050	0.312	8 / 8
211 (3D)	0.030/ 0.050	-0.002	0.029	0.007	0.508	10 / 10
210 (3D)	0.030/ 0.050	-0.005	-0.016	0.010	0.339	11 / 11
206 (3D)	0.030/ 0.050	0.019	0.031	-0.003	0.266	9 / 9
205 (3D)	0.030/ 0.050	-0.013	-0.027	0.001	0.830	8 / 8
204 (3D)	0.030/ 0.050	0.008	0.005	-0.026	0.482	8 / 8
**Mean [m]**		-0.000402	-0.000181	0.001076		
**Sigma [m]**		0.010314	0.019312	0.019259		
**RMS Error [m]**		0.010322	0.019313	0.019289		

0 out of 207 check points have been labeled as inaccurate.

Check Point Name	Accuracy XY/Z [m]	Error X [m]	Error Y [m]	Error Z [m]	Projection Error [pixel]	Verified/Marked
103		0.0114	0.0321	-0.0195	0.3252	11 / 11
101		-0.0264	0.0140	0.0399	0.3115	10 / 10
100		-0.0102	-0.0029	0.0436	0.2732	11 / 11
104		-0.0560	-0.0009	0.0242	0.2080	8 / 8
102		-0.0193	-0.0065	0.0175	0.3988	11 / 11
k1		0.0512	-0.0601	0.1204	0.4137	8 / 8
k2		0.0067	0.0211	-0.0185	0.5250	10 / 10
k3		0.0939	-0.0812	0.1490	0.4745	6 / 6
k4		0.0418	-0.0337	0.0383	0.3772	7 / 7
k5		-0.0179	0.0262	-0.0579	0.4655	7 / 7
k6		-0.0205	0.0131	0.0918	0.9381	8 / 8
k7		-0.0727	0.0045	0.1779	0.6181	7 / 7
k8		-0.0258	-0.0391	0.1042	0.4561	8 / 8
k11		0.0034	-0.0171	-0.0438	0.4264	10 / 10
k13		0.0019	-0.0020	-0.0190	0.3540	9 / 9
k14		-0.0013	-0.0221	-0.0175	0.4089	8 / 8
10000		-0.0328	-0.0093	0.0850	0.5903	7 / 7
10001		0.0153	-0.0175	0.0387	0.3774	8 / 8
10002		0.0094	-0.0278	-0.0045	0.2528	9 / 9
10003		0.0089	-0.0313	-0.0292	0.3193	10 / 10
10004		0.0219	-0.0007	-0.0031	0.4088	10 / 10
10005		0.0337	-0.0077	-0.0176	0.3574	11 / 11
10006		0.0127	-0.0132	-0.0367	0.3476	11 / 11
10007		0.0072	-0.0042	-0.0221	0.4082	9 / 9
10008		0.0197	-0.0190	-0.0267	0.3427	7 / 7
10009		0.0122	-0.0294	-0.0030	0.4368	11 / 11
10010		0.0235	-0.0227	-0.0238	0.3727	11 / 11

10011		0.0084	-0.0474	-0.0399	0.4049	9 / 9
10012		-0.0294	0.0028	0.0136	0.3363	11 / 11
10013		-0.0263	0.0130	0.0244	0.4547	10 / 10
10014		-0.0001	0.0157	0.0146	0.3920	10 / 10
10015		0.0222	0.0076	0.0465	0.3741	10 / 10
10016		-0.0075	0.0065	0.0121	0.5250	12 / 12
10017		0.0001	-0.0072	-0.0210	0.6600	11 / 11
10018		-0.0210	0.0054	0.0152	0.2620	6 / 6
10019		-0.0257	0.0240	0.0615	0.2720	10 / 10
10020		-0.0201	0.0351	0.0222	0.5377	8 / 8
10021		0.0037	-0.0002	0.0134	0.3487	9 / 9
10022		0.0126	0.0361	0.0042	0.3453	10 / 10
10023		0.0231	-0.0020	-0.0264	0.4237	9 / 9
10024		-0.0271	0.0025	-0.0644	0.3373	11 / 11
10025		-0.0032	0.0062	-0.0278	0.5211	10 / 10
10026		-0.0183	0.0163	-0.0356	0.2611	8 / 8
10027		-0.0138	0.0088	-0.0260	0.3769	10 / 10
10028		0.0016	0.0005	-0.0130	0.3801	11 / 11
10030		0.0013	0.0299	-0.0303	0.3400	11 / 11
10031		0.0289	0.0382	-0.0006	0.4153	7 / 7
10032		0.0428	0.0307	-0.0322	0.3364	7 / 7
10033		0.0068	0.0376	-0.0008	0.3473	11 / 11
10034		0.0275	-0.0033	-0.0390	0.4236	11 / 11
10035		0.0177	-0.0212	0.0133	0.5317	9 / 9
10036		0.0215	0.0083	-0.0075	0.3795	6 / 6
10037		-0.0042	-0.0107	-0.0206	0.3290	9 / 9
10038		-0.0154	0.0110	-0.0106	0.2904	10 / 10
10039		-0.0006	-0.0121	-0.0324	0.3574	10 / 10
10040		-0.0228	0.0063	0.0006	0.5060	8 / 8
10041		-0.0234	-0.0134	0.0268	0.6667	9 / 9
10044		0.0092	-0.0280	-0.0348	0.2893	9 / 9
10045		0.0197	-0.0173	-0.0840	0.3367	9 / 9
10046		-0.0156	0.0073	-0.0715	0.3580	10 / 10
10047		-0.0333	-0.0060	0.0813	0.8687	9 / 9
10048		0.0026	-0.0395	0.0318	0.4585	10 / 10
10049		-0.0215	0.0491	-0.0237	0.8436	9 / 9
10051		0.0060	0.0138	-0.0072	0.3833	11 / 11
10052		0.0122	-0.0127	-0.0069	0.2733	7 / 7
10053		-0.0022	0.0168	0.0039	0.3811	10 / 10
10054		0.0029	-0.0250	-0.0057	0.3433	10 / 10
10055		-0.0203	0.0118	-0.0196	0.3458	8 / 8
10056		-0.0475	0.0173	0.0062	0.3377	10 / 10
10058		-0.0770	0.0940	0.0483	0.4685	11 / 11
10059		-0.0110	-0.0086	-0.0576	0.3555	11 / 11
10060		-0.0256	-0.0180	-0.0130	0.3627	11 / 11
10061		0.0113	0.0239	0.0354	0.4392	7 / 7
10062		0.0030	-0.0302	0.0225	0.2977	10 / 10
10063		0.0152	-0.0384	-0.0156	0.5683	10 / 10
10064		0.0419	0.0270	-0.0406	0.3725	11 / 11
10065		0.0238	0.0430	-0.0317	0.3890	11 / 11
10066		0.0521	0.0415	-0.0489	0.4743	13 / 13
10067		0.0266	0.0397	-0.0266	0.3087	12 / 12
10068		0.0112	0.1178	-0.0514	0.4586	12 / 12
10074		-0.0183	0.0487	-0.0710	0.5681	11 / 11
10075		-0.0145	0.0570	-0.0049	0.3807	10 / 10
10076		0.0037	0.0307	-0.0282	0.3966	10 / 10
10077		-0.0272	0.0577	-0.0325	0.7419	10 / 10
10078		0.0191	0.0120	-0.0216	0.3604	10 / 10
10080		0.0309	-0.0730	-0.1050	0.3743	9 / 9

10081		-0.0232	-0.0153	-0.0113	0.3709	10 / 10
10082		0.0177	-0.0430	-0.0339	0.3203	11 / 11
10083		0.0490	-0.0056	-0.1203	0.5092	11 / 11
10085		0.0384	-0.0332	0.0097	0.3653	12 / 12
15000		0.0076	-0.0153	-0.0573	0.3174	12 / 12
15001		-0.0261	-0.0291	-0.0554	0.3268	11 / 11
15002		0.0182	0.0132	-0.0822	0.2963	10 / 10
15003		-0.0035	0.0372	-0.0072	0.4937	12 / 12
15004		-0.0150	0.0263	-0.0338	0.3368	12 / 12
15005		-0.0092	0.0229	0.0013	0.4587	9 / 9
15006		-0.0106	0.0349	-0.0123	0.5544	11 / 11
15007		-0.0051	-0.0044	0.0331	0.4596	8 / 8
15008		-0.0197	0.0095	0.0695	0.9694	10 / 10
15009		-0.0119	-0.0094	0.0320	0.3588	9 / 9
15010		0.0216	0.0105	0.0240	0.2367	9 / 9
20000		-0.0091	0.0095	0.0436	0.5011	10 / 10
20001		-0.0164	-0.0085	0.0400	0.4763	9 / 9
20002		0.0042	-0.0002	0.0166	0.3779	9 / 9
20003		-0.0108	-0.0196	-0.0200	0.3322	8 / 8
20004		0.0120	-0.0217	-0.0035	0.2391	8 / 8
20005		-0.0570	-0.0225	0.0176	0.4528	9 / 9
20008		0.0048	0.0027	0.0013	0.5330	12 / 12
20009		0.0286	0.0014	-0.0009	0.3921	10 / 10
20010		-0.0181	0.0274	-0.0197	0.3389	12 / 12
20011		-0.0166	-0.0019	-0.0313	0.4355	12 / 12
20012		0.0119	0.0110	-0.0358	0.2929	11 / 11
20013		0.0100	0.0150	-0.0300	0.3865	10 / 10
20014		0.0044	0.0182	-0.0292	0.4373	11 / 11
20016		-0.0140	0.0287	-0.0721	0.2529	10 / 10
20017		-0.0006	0.0248	-0.0527	0.2856	8 / 8
20018		0.0151	0.0309	-0.0602	0.3298	12 / 12
20019		0.0178	0.0225	-0.0543	0.3772	13 / 13
20020		0.0140	0.0378	-0.0062	0.4780	11 / 11
20021		0.0058	0.0145	-0.0220	0.2669	10 / 10
20022		-0.0153	-0.0347	-0.0252	0.3002	10 / 10
20023		0.0049	0.0230	-0.0247	0.3577	13 / 13
20024		-0.0045	0.0542	-0.0507	0.4582	10 / 10
20025		0.0236	0.0560	-0.0451	0.2712	10 / 10
20026		0.0281	0.0521	-0.0277	0.4486	11 / 11
20027		0.0035	0.0740	-0.0264	0.4028	11 / 11
20028		-0.0071	0.0101	-0.0098	0.3528	12 / 12
20029		0.0183	-0.0258	-0.0210	0.6873	9 / 9
20031		-0.0211	0.0278	-0.0157	0.5180	10 / 10
25000		0.0075	0.0177	-0.0234	0.2592	9 / 9
25001		-0.0087	0.0125	0.0364	0.2855	10 / 10
25003		0.0101	-0.0064	0.0206	0.4071	10 / 10
25004		0.0003	0.0213	-0.0114	0.3851	10 / 10
25005		0.0022	0.0093	0.0151	0.2361	9 / 9
25006		-0.0265	-0.0169	-0.0747	0.3847	10 / 10
25008		-0.0366	0.0249	-0.0432	0.2490	10 / 10
25009		0.0258	-0.0299	-0.0452	0.2384	11 / 11
25010		0.0004	0.0073	-0.0501	0.2217	11 / 11
25013		0.0014	0.0255	-0.0175	0.4302	10 / 10
25014		-0.0099	-0.0038	-0.0319	0.3440	10 / 10
25015		-0.0188	-0.0010	-0.0340	0.3873	11 / 11
25016		-0.0227	-0.0024	-0.0314	0.2706	11 / 11
25017		0.0187	-0.0116	0.0453	0.6151	10 / 10
25018		0.0071	-0.0027	0.0237	0.3761	9 / 9
25019		-0.0096	-0.0132	0.0107	0.3279	9 / 9

25020		-0.0079	0.0128	-0.0107	0.3707	6 / 6
25021		0.0197	0.0202	-0.0320	0.4010	9 / 9
25022		0.0169	0.0151	-0.0076	0.6770	9 / 9
25023		-0.0033	0.0261	0.0017	0.3206	8 / 8
25024		0.0066	-0.0044	-0.0362	0.3760	11 / 11
25029		0.0187	0.0063	0.0073	0.4027	10 / 10
25030		-0.0108	0.0048	0.0216	0.3204	11 / 11
25032		-0.0665	-0.0175	-0.0355	0.3935	11 / 11
25033		-0.0137	-0.0134	0.0074	0.4738	10 / 10
25034		-0.0036	-0.0135	-0.0085	0.3769	9 / 9
40000		-0.0043	-0.0112	-0.1074	0.3568	12 / 12
40001		0.0218	0.0342	-0.0174	0.3208	10 / 10
40002		0.0073	0.0555	-0.0515	0.3043	12 / 12
40003		0.0041	0.0652	-0.0474	0.4790	9 / 9
40004		0.0059	0.0234	-0.0451	0.2690	10 / 10
40005		0.0146	0.0451	-0.0382	0.3367	7 / 7
45000		0.0425	-0.0309	0.0100	0.3947	10 / 10
45001		0.0008	-0.0025	-0.0006	0.2716	9 / 9
45002		0.0231	-0.0375	0.0070	0.4646	11 / 11
10043		-0.0234	-0.0085	0.0367	0.3871	8 / 8
10084		-0.0001	-0.0055	0.0277	0.3684	7 / 7
20015		-0.0135	0.0040	-0.0679	0.3017	11 / 11
20007		-0.0025	0.0059	0.0007	0.3099	4 / 4
25027		-0.0104	0.0006	-0.0392	0.2999	9 / 9
k9		-0.0082	-0.0086	-0.0114	0.3801	11 / 11
6600		-0.0022	-0.0093	-0.0541	0.4089	12 / 12
6602		0.0158	0.0418	-0.0422	0.3540	11 / 11
6603		0.0044	-0.0178	-0.0144	0.4328	10 / 10
6605		0.0173	0.0032	0.0053	0.4527	9 / 9
6606		0.0191	0.0210	-0.0494	0.4795	7 / 7
6607		0.0146	0.0188	-0.0457	0.5190	11 / 11
6608		-0.0083	-0.0505	-0.0887	0.3871	3 / 3
6609		-0.0183	0.0095	0.0158	0.3399	8 / 8
6610		-0.0006	0.0038	0.0343	0.6650	7 / 7
6611		-0.0449	-0.0205	-0.0542	0.3661	9 / 9
6612		-0.0245	-0.0444	-0.0891	0.4962	8 / 8
6616		-0.0053	-0.0141	-0.0644	0.4133	8 / 8
6617		-0.0205	0.0127	0.0520	0.3201	4 / 4
6618		-0.0104	-0.0001	-0.0101	0.5074	10 / 10
6619		0.0139	0.0079	0.0214	0.2803	10 / 10
6620		0.0261	-0.0400	0.0069	0.4847	9 / 9
6621		-0.0134	0.0012	0.0041	0.3263	11 / 11
6622		-0.0199	0.0121	0.0130	0.4501	9 / 9
6623		-0.0163	-0.0159	-0.0079	0.5437	10 / 10
6624		0.0228	-0.0152	-0.0460	0.7230	6 / 6
6625		-0.0283	-0.0415	-0.0535	0.4335	8 / 8
6626		-0.0263	-0.0200	-0.1122	0.4344	9 / 9
6627		-0.0278	-0.0446	-0.0598	0.4347	8 / 8
6628		-0.0304	-0.0637	-0.0831	0.3042	9 / 9
6629		-0.0446	-0.0520	-0.0876	0.3736	9 / 9
6630		-0.0575	-0.0326	-0.0945	0.3158	8 / 8
6631		-0.0264	-0.0468	-0.0768	0.4493	7 / 7
6632		-0.0548	-0.0320	-0.0769	0.6291	9 / 9
6633		-0.0211	-0.0282	-0.0943	0.5408	8 / 8
6634		-0.0202	-0.0158	-0.0932	0.2544	8 / 8
6635		-0.0011	-0.0035	-0.0698	0.4097	9 / 9
6636		-0.0460	-0.0559	-0.0824	0.4908	10 / 10
6637		-0.0118	-0.0397	-0.0800	0.5083	8 / 8
6638		-0.0222	-0.0294	-0.0755	0.2780	7 / 7

6639		-0.0521	-0.0239	-0.0652	0.4468	10 / 10
6640		-0.0453	-0.0353	-0.0685	0.4063	8 / 8
6641		-0.0414	-0.0314	-0.0568	0.5459	10 / 10
**Mean [m]**		-0.002802	0.001015	-0.016160		
**Sigma [m]**		0.023973	0.029103	0.044557		
**RMS Error [m]**		0.024136	0.029121	0.047397		

Localisation accuracy per GCP and mean errors in the three coordinate directions. The last column counts the number of calibrated images where the GCP has been automatically verified vs. manually marked.

Initial Processing Details

System Information

Hardware	CPU: Intel(R) Core(TM) i9-7940X CPU @ 3.10GHz<br>RAM: 128GB<br>GPU: NVIDIA GeForce GTX 1080 Ti (Driver: 26.21.14.3615), RDPDD Chained DD (Driver: unknown), RDP Encoder Mirror Driver (Driver: unknown), RDP Reflector Display Driver (Driver: unknown)
Operating System	Windows 7 Ultimate, 64-bit

Coordinate Systems

Ground Control Point (GCP) Coordinate System	ETRS89 / Poland CS2000 zone 6
Output Coordinate System	ETRS89 / Poland CS2000 zone 6

Processing Options

Detected Template	No Template Available
Keypoints Image Scale	Full, Image Scale: 1
Advanced: Matching Image Pairs	Aerial Grid or Corridor
Advanced: Matching Strategy	Use Geometrically Verified Matching: yes
Advanced: Keypoint Extraction	Targeted Number of Keypoints: Automatic
Advanced: Calibration	Calibration Method: Standard<br>Internal Parameters Optimization: All<br>External Parameters Optimization: All<br>Rematch: Auto, yes
