# Supplementary material for: Multi-Variant Accuracy Evaluation of UAV Imaging Surveys: A Case Study on Investment Area
Source: Sensors (Basel). 2019 Nov 28;19(23):5229. doi: 10.3390/s19235229 (PMC6929115; doi:10.3390/s19235229)
Supplement: Supplementary file 1 [file sensors-19-05229-s001.zip › supplementary_files/App12_V6_p85q45_GPS_report.pdf]

Quality Report

Generated with Pix4Ddiscovery version 4.3.31

Important: Click on the different icons for:

Help to analyze the results in the Quality Report

Additional information about the sections

Click here for additional tips to analyze the Quality Report

Summary

Project	V6_p85q45_GPS
Processed	2019-10-17 02:31:52
Camera Model Name(s)	NEX-5T_0.0_4912x3264 (RGB)
Average Ground Sampling Distance (GSD)	4.57 cm / 1.80 in
Area Covered	0.970 km^2 / 96.9643 ha / 0.37 sq. mi. / 239.7279 acres
Time for Initial Processing (without report)	13m:36s

Quality Check

**Images**	median of 45820 keypoints per image	✓
**Dataset**	293 out of 293 images calibrated (100%), all images enabled	✓
**Camera Optimization**	0.02% relative difference between initial and optimized internal camera parameters	✓
**Matching**	median of 22015.8 matches per calibrated image	✓
**Georeferencing**	yes, 11 GCPs (11 3D), mean RMS error = 0.022 m	✓

Preview

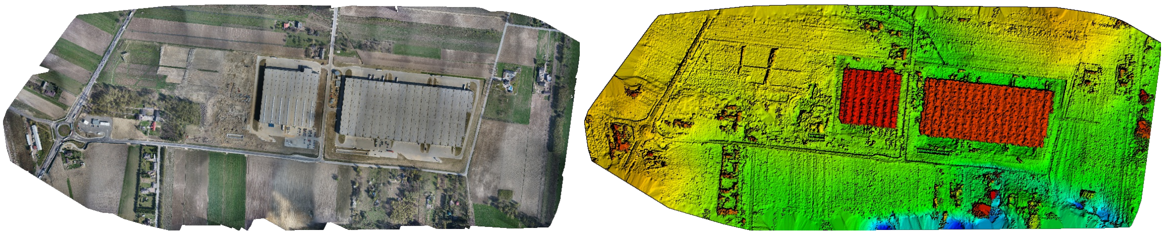

Figure 1: Orthomosaic and the corresponding sparse Digital Surface Model (DSM) before densification.

Calibration Details

Number of Calibrated Images	293 out of 293
Number of Geolocated Images	293 out of 293

Initial Image Positions

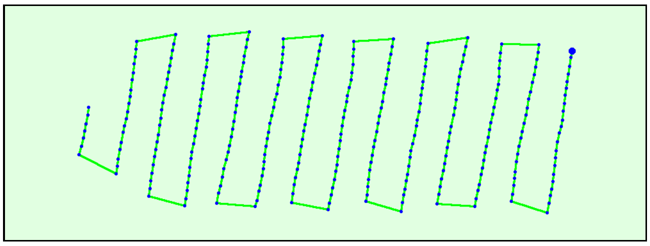

Figure 2: Top view of the initial image position. The green line follows the position of the images in time starting from the large blue dot.

Computed Image/GCPs/Manual Tie Points Positions

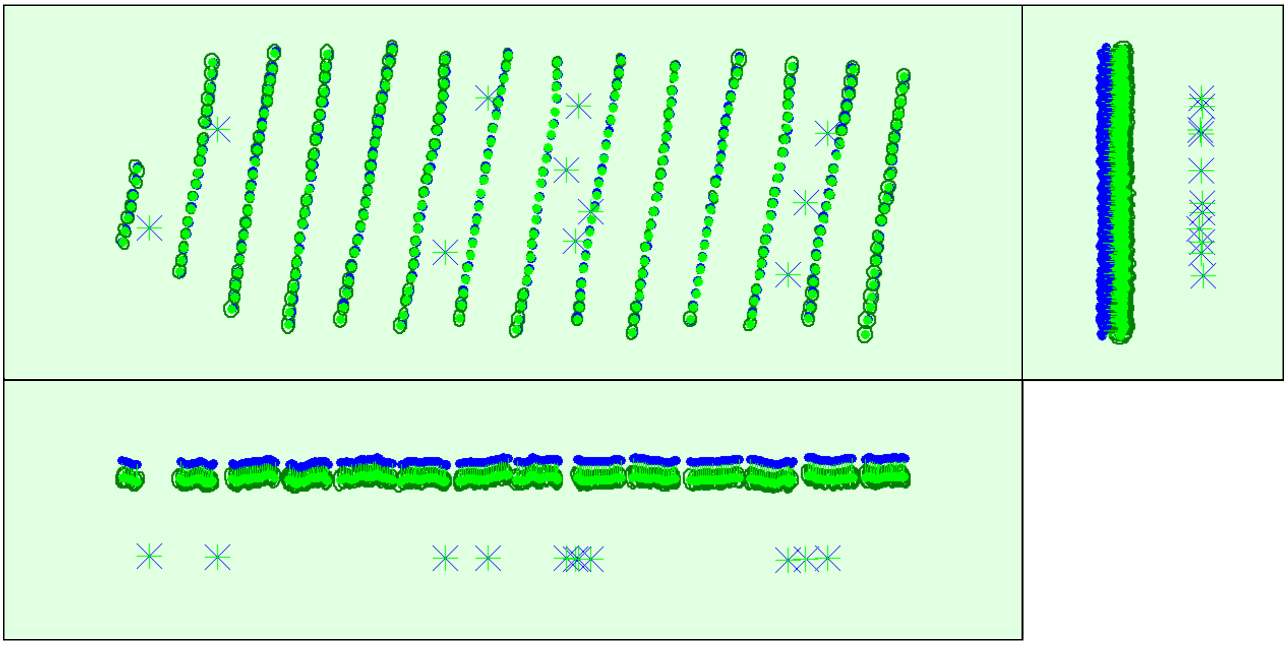

Uncertainty ellipses 500x magnified

Figure 3: Offset between initial (blue dots) and computed (green dots) image positions as well as the offset between the GCPs initial positions (blue crosses) and their computed positions (green crosses) in the top-view (XY plane), front-view (XZ plane), and side-view (YZ plane). Dark green ellipses indicate the absolute position uncertainty of the bundle block adjustment result.

Absolute camera position and orientation uncertainties

	X[m]	Y[m]	Z[m]	Omega [degree]	Phi [degree]	Kappa [degree]
Mean	0.017	0.021	0.036	0.007	0.006	0.002
Sigma	0.004	0.005	0.001	0.002	0.002	0.001

Overlap

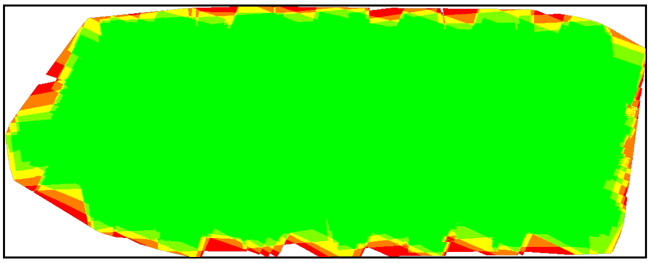

Number of overlapping images: 1 2 3 4 5+

Figure 4: Number of overlapping images computed for each pixel of the orthomosaic.
Red and yellow areas indicate low overlap for which poor results may be generated. Green areas indicate an overlap of over 5 images for every pixel. Good quality results will be generated as long as the number of keypoint matches is also sufficient for these areas (see Figure 5 for keypoint matches).

Bundle Block Adjustment Details

Number of 2D Keypoint Observations for Bundle Block Adjustment	6516718
Number of 3D Points for Bundle Block Adjustment	2149825
Mean Reprojection Error [pixels]	0.190

Internal Camera Parameters

NEX-5T_0.0_4912x3264 (RGB). Sensor Dimensions: 23.400 [mm] x 15.549 [mm]

EXIF ID: NEX-5T_0.0_4912x3264

	Focal Length	Principal Point x	Principal Point y	R1	R2	R3	T1	T2
Initial Values	3256.554 [pixel] 15.514 [mm]	2456.002 [pixel] 11.700 [mm]	1632.003 [pixel] 7.775 [mm]	-0.043	0.028	-0.005	0.000	-0.001
Optimized Values	3257.442 [pixel] 15.518 [mm]	2466.767 [pixel] 11.751 [mm]	1589.426 [pixel] 7.572 [mm]	-0.047	0.035	-0.010	0.001	0.000
Uncertainties (Sigma)	0.761 [pixel] 0.004 [mm]	0.135 [pixel] 0.001 [mm]	0.127 [pixel] 0.001 [mm]	0.000	0.000	0.000	0.000	0.000

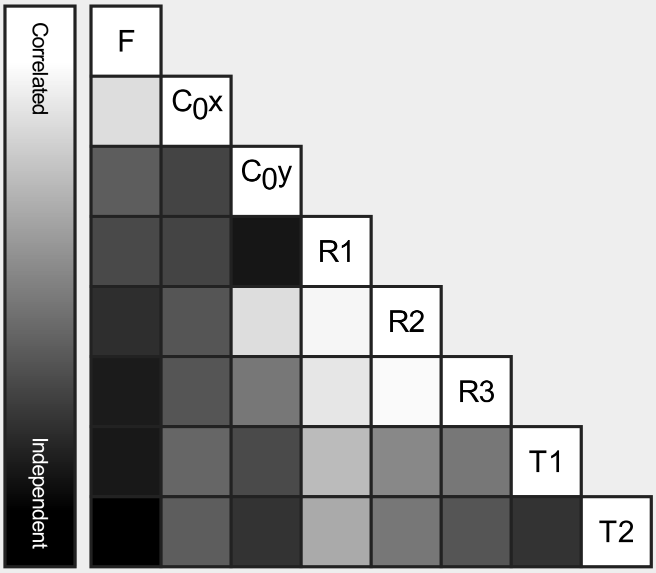

The correlation between camera internal parameters determined by the bundle adjustment. White indicates a full correlation between the parameters, ie. any change in one can be fully compensated by the other. Black indicates that the parameter is completely independent, and is not affected by other parameters.

The number of Automatic Tie Points (ATPs) per pixel, averaged over all images of the camera model, is color coded between black and white. White indicates that, on average, more than 16 ATPs have been extracted at the pixel location. Black indicates that, on average, 0 ATPs have been extracted at the pixel location. Click on the image to the see the average direction and magnitude of the re-projection error for each pixel. Note that the vectors are scaled for better visualization. The scale bar indicates the magnitude of 1 pixel error.

2D Keypoints Table

	Number of 2D Keypoints per Image	Number of Matched 2D Keypoints per Image
Median	45820	22016
Min	20057	6200
Max	68781	40198
Mean	42855	22241

3D Points from 2D Keypoint Matches

	Number of 3D Points Observed
In 2 Images	1233120

In 3 Images	407108
In 4 Images	203196
In 5 Images	118829
In 6 Images	75277
In 7 Images	43774
In 8 Images	23863
In 9 Images	14755
In 10 Images	10704
In 11 Images	7700
In 12 Images	5292
In 13 Images	3349
In 14 Images	1776
In 15 Images	701
In 16 Images	250
In 17 Images	86
In 18 Images	35
In 19 Images	7
In 20 Images	2
In 21 Images	1

2D Keypoint Matches

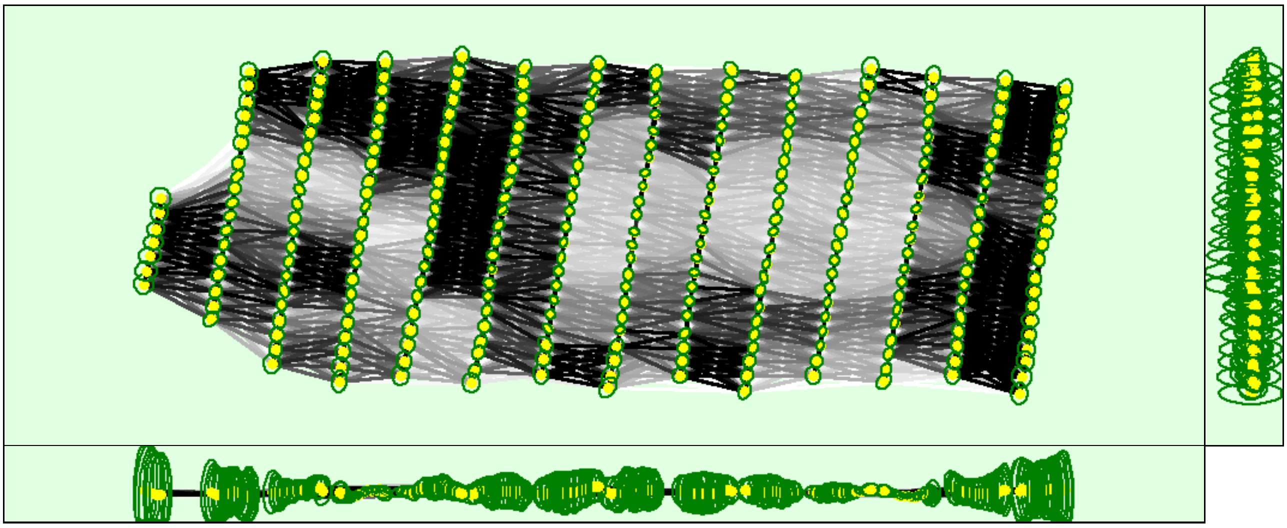

Uncertainty ellipses 500x magnified

Number of matches

25 222 444 666 888 1111 1333 1555 1777 2000

Figure 5: Computed image positions with links between matched images. The darkness of the links indicates the number of matched 2D keypoints between the images. Bright links indicate weak links and require manual tie points or more images. Dark green ellipses indicate the relative camera position uncertainty of the bundle block adjustment result.

Relative camera position and orientation uncertainties

	X[m]	Y[m]	Z[m]	Omega [degree]	Phi [degree]	Kappa [degree]
Mean	0.019	0.021	0.050	0.012	0.014	0.003
Sigma	0.004	0.005	0.028	0.005	0.007	0.001

Geolocation Details

Ground Control Points

GCP Name	Accuracy XY/Z [m]	Error X [m]	Error Y [m]	Error Z [m]	Projection Error [pixel]	Verified/Marked
201 (3D)	0.030/ 0.050	-0.007	-0.022	0.017	0.510	11 / 11
202 (3D)	0.030/ 0.050	-0.001	0.018	-0.008	0.544	10 / 10
203 (3D)	0.030/ 0.050	-0.008	0.025	-0.030	0.684	13 / 13
208 (3D)	0.030/ 0.050	0.001	-0.006	-0.031	0.519	12 / 12
209 (3D)	0.030/ 0.050	0.008	-0.013	-0.014	0.364	13 / 13
207 (3D)	0.030/ 0.050	-0.031	-0.004	0.073	0.333	11 / 11
211 (3D)	0.030/ 0.050	0.019	0.024	-0.010	0.442	13 / 13
210 (3D)	0.030/ 0.050	0.005	-0.010	0.009	0.235	13 / 13
206 (3D)	0.030/ 0.050	0.013	0.018	0.017	0.294	14 / 14
205 (3D)	0.030/ 0.050	-0.024	-0.050	-0.001	0.777	15 / 15
204 (3D)	0.030/ 0.050	0.023	0.020	-0.018	0.809	11 / 12
**Mean [m]**		-0.000219	0.000078	0.000278		
**Sigma [m]**		0.015861	0.022405	0.027832		
**RMS Error [m]**		0.015863	0.022405	0.027833		

0 out of 207 check points have been labeled as inaccurate.

Check Point Name	Accuracy XY/Z [m]	Error X [m]	Error Y [m]	Error Z [m]	Projection Error [pixel]	Verified/Marked
103		0.0091	0.0276	-0.0209	0.4827	12 / 12
101		-0.0068	0.0150	0.0143	0.3398	13 / 13
100		-0.0083	-0.0204	0.0465	0.2954	12 / 12
104		-0.0473	0.0103	-0.0089	0.4434	13 / 13
102		-0.0197	-0.0306	0.0112	0.3285	14 / 14
k1		0.0410	-0.0440	0.1212	0.4544	14 / 14
k2		0.0072	0.0260	-0.0282	0.4552	14 / 14
k3		0.0993	-0.0791	0.1636	0.6484	13 / 13
k4		0.0350	-0.0257	0.0859	0.3845	12 / 12
k5		-0.0141	0.0269	-0.0350	0.4959	13 / 13
k6		-0.0280	0.0226	0.0608	0.4906	10 / 10
k7		-0.0868	0.0102	0.1652	0.4892	8 / 8
k8		-0.0487	-0.0306	0.1605	0.4984	12 / 12
k11		-0.0087	-0.0407	-0.1275	0.5660	15 / 15
k13		-0.0008	0.0073	-0.0040	0.3812	12 / 12
k14		0.0075	-0.0012	-0.0134	0.4359	13 / 13
10000		-0.0192	-0.0164	0.0225	0.4896	12 / 12
10001		-0.0012	0.0015	0.0746	0.6324	12 / 12
10002		0.0092	-0.0294	0.0253	0.2792	11 / 11
10003		-0.0042	-0.0426	-0.0006	0.5963	15 / 15
10004		-0.0012	-0.0053	0.0804	0.4786	12 / 12
10005		0.0092	-0.0095	0.0818	0.5484	13 / 13
10006		-0.0042	-0.0171	0.0719	0.3639	13 / 13
10007		0.0229	-0.0197	0.0386	0.2920	14 / 14
10008		0.0312	-0.0323	0.0271	0.3247	16 / 16
10009		0.0189	-0.0379	0.0165	0.4576	14 / 14
10010		0.0237	-0.0440	0.0086	0.3675	13 / 13
10011		0.0154	-0.0493	-0.0045	0.5365	12 / 12
10012		-0.0470	-0.0003	0.0362	0.4230	15 / 15
10013		-0.0259	-0.0141	0.0046	0.3822	13 / 13
10014		0.0027	-0.0002	-0.0033	0.4014	13 / 13
10015		0.0115	-0.0162	0.0012	0.4671	15 / 15
10016		-0.0206	-0.0200	-0.0242	0.3594	13 / 13
10017		0.0104	-0.0312	-0.0719	0.5051	14 / 14
10018		0.0107	-0.0118	-0.0234	0.3306	6 / 6
10019		-0.0130	0.0182	0.0472	0.6695	14 / 14
10020		-0.0335	0.0100	0.0186	0.4519	13 / 13
10021		-0.0057	0.0025	-0.0005	0.3815	18 / 18
10022		0.0177	0.0404	-0.0552	0.5419	15 / 15
10023		0.0173	0.0069	-0.0682	0.4353	12 / 12
10024		-0.0112	0.0093	-0.0804	0.4467	18 / 18
10025		0.0034	0.0121	-0.0549	0.3325	16 / 16

10026		-0.0122	0.0293	-0.0583	0.4039	13 / 13
10027		-0.0084	0.0273	-0.0408	0.4775	14 / 14
10028		0.0011	0.0112	-0.0281	0.4445	15 / 15
10030		-0.0144	0.0445	-0.0182	0.3486	13 / 13
10031		0.0160	0.0411	0.0349	0.5263	7 / 7
10032		0.0442	0.0632	-0.0170	0.4239	6 / 6
10033		0.0013	0.0341	0.0183	0.3681	15 / 15
10034		0.0221	-0.0051	0.0070	0.3606	13 / 13
10035		0.0079	-0.0247	0.0616	0.3339	11 / 11
10036		0.0053	-0.0057	0.0034	0.6005	9 / 9
10037		-0.0186	-0.0006	-0.0096	0.3280	11 / 11
10038		-0.0169	0.0136	-0.0017	0.3210	14 / 14
10039		0.0071	-0.0094	-0.0136	0.3972	15 / 15
10040		-0.0207	-0.0035	0.0059	0.3278	11 / 11
10041		-0.0194	-0.0184	0.0398	0.8898	14 / 14
10044		0.0058	-0.0446	-0.0263	0.3475	12 / 12
10045		0.0153	-0.0335	-0.0559	0.3619	14 / 14
10046		-0.0254	-0.0175	-0.0706	0.3867	13 / 13
10047		-0.0437	-0.0154	0.0851	0.5026	12 / 12
10048		-0.0275	-0.0344	0.0766	0.6872	14 / 14
10049		-0.0191	0.0196	0.0318	0.7563	14 / 14
10051		0.0047	0.0233	0.0035	0.3125	14 / 14
10052		0.0060	-0.0041	0.0402	0.4892	13 / 13
10053		-0.0180	-0.0032	-0.0302	0.4660	13 / 13
10054		-0.0077	-0.0333	-0.0266	0.5019	13 / 13
10055		-0.0132	0.0108	-0.0683	0.5603	11 / 11
10056		-0.0525	0.0118	0.0070	0.4441	12 / 12
10058		-0.0792	0.0846	0.0433	0.4729	15 / 15
10059		0.0055	-0.0292	-0.0164	0.3479	15 / 15
10060		-0.0257	-0.0434	-0.0266	0.3400	15 / 15
10061		0.0215	0.0030	-0.0111	0.4264	9 / 9
10062		0.0020	-0.0489	-0.0412	0.4165	15 / 15
10063		0.0189	-0.0656	-0.0567	0.5299	14 / 14
10064		0.0299	0.0297	-0.0527	0.4462	15 / 15
10065		0.0281	0.0387	-0.0131	0.3325	12 / 12
10066		0.0620	0.0397	-0.0586	0.3672	11 / 11
10067		0.0289	0.0460	-0.0416	0.4088	12 / 12
10068		0.0080	0.1157	-0.0277	0.4656	14 / 14
10074		-0.0177	0.0502	-0.0217	0.5460	13 / 13
10075		-0.0211	0.0530	0.0212	0.3076	13 / 13
10076		-0.0010	0.0348	-0.0147	0.4216	13 / 13
10077		-0.0318	0.0669	-0.0425	0.6311	12 / 12
10078		-0.0052	0.0296	-0.0280	0.2178	11 / 11
10080		0.0161	-0.0636	-0.0899	0.7897	14 / 14
10081		-0.0246	-0.0344	0.0145	0.3667	15 / 15
10082		0.0102	-0.0424	-0.0260	0.3969	16 / 16
10083		0.0482	-0.0036	-0.0939	0.5099	13 / 13
10085		0.0390	-0.0548	-0.0141	0.3242	15 / 15
15000		0.0033	-0.0414	0.0223	0.2700	14 / 14
15001		-0.0149	-0.0437	0.0197	0.3402	14 / 14
15002		-0.0136	0.0294	-0.0918	0.3139	14 / 14
15003		-0.0290	0.0467	-0.0226	0.3435	16 / 16
15004		-0.0179	0.0176	-0.0286	0.2960	14 / 14
15005		-0.0058	0.0253	-0.0409	0.6980	11 / 11
15006		0.0116	0.0371	-0.0357	0.3395	13 / 13
15007		0.0201	-0.0213	-0.0176	0.2731	11 / 11
15008		-0.0258	0.0093	0.0763	0.8076	11 / 11
15009		0.0036	-0.0007	0.0134	0.4722	13 / 13
15010		0.0115	0.0380	0.0331	0.5862	12 / 12

20000		0.0010	0.0202	0.0409	0.6169	13 / 13
20001		-0.0039	-0.0051	0.0337	0.3526	11 / 11
20002		0.0092	0.0064	0.0110	0.2878	12 / 12
20003		-0.0198	-0.0150	0.0044	0.3845	13 / 13
20004		-0.0033	-0.0140	0.0230	0.4008	12 / 12
20005		-0.0655	-0.0439	0.0174	0.3419	13 / 13
20008		-0.0056	-0.0146	-0.0108	0.3797	14 / 14
20009		0.0169	-0.0287	-0.0486	0.4339	16 / 16
20010		-0.0279	0.0299	-0.0700	0.3832	16 / 16
20011		-0.0181	0.0089	-0.0531	0.3568	19 / 19
20012		0.0078	0.0025	-0.0644	0.3656	15 / 15
20013		0.0130	0.0054	-0.0306	0.3779	12 / 12
20014		0.0035	0.0145	-0.0309	0.3593	14 / 14
20016		-0.0057	0.0198	-0.1020	0.2479	17 / 17
20017		0.0009	0.0326	-0.0865	0.2953	13 / 13
20018		0.0170	0.0259	-0.0640	0.3308	14 / 14
20019		0.0268	0.0247	-0.0314	0.3161	13 / 13
20020		0.0049	0.0479	-0.0029	0.4331	13 / 13
20021		-0.0044	0.0059	0.0157	0.2202	14 / 14
20022		-0.0272	-0.0445	0.0158	0.2438	14 / 14
20023		-0.0184	0.0089	-0.0114	0.2370	14 / 14
20024		-0.0078	0.0499	-0.0172	0.4190	13 / 13
20025		0.0074	0.0534	-0.0146	0.2883	13 / 13
20026		0.0049	0.0483	0.0007	0.3591	14 / 14
20027		-0.0261	0.0855	-0.0306	0.4179	11 / 11
20028		-0.0232	0.0217	-0.0142	0.3568	12 / 12
20029		0.0124	-0.0290	-0.0402	0.6737	15 / 15
20031		-0.0249	0.0227	-0.0595	0.5160	14 / 14
25000		0.0119	0.0126	-0.0263	0.2377	15 / 15
25001		0.0075	-0.0006	-0.0035	0.2072	11 / 11
25003		0.0038	-0.0198	0.0206	0.3357	13 / 13
25004		0.0047	-0.0007	-0.0005	0.3095	12 / 12
25005		0.0058	-0.0123	0.0321	0.2879	13 / 13
25006		-0.0353	-0.0347	-0.0538	0.4184	13 / 13
25008		-0.0166	-0.0029	0.0207	0.1837	13 / 13
25009		0.0397	-0.0495	0.0114	0.2799	16 / 16
25010		0.0171	-0.0187	0.0026	0.2545	16 / 16
25013		-0.0072	0.0078	-0.0286	0.3375	12 / 12
25014		-0.0199	-0.0196	-0.0355	0.2679	13 / 13
25015		-0.0294	-0.0222	-0.0312	0.3540	13 / 13
25016		-0.0328	-0.0177	-0.0257	0.1865	13 / 13
25017		0.0242	-0.0389	0.0422	0.3439	12 / 12
25018		0.0021	-0.0126	0.0258	0.3859	13 / 13
25019		-0.0218	-0.0265	0.0290	0.4330	13 / 13
25020		-0.0188	-0.0128	-0.0345	0.2556	8 / 8
25021		0.0061	0.0185	0.0234	0.3318	13 / 13
25022		-0.0014	0.0024	0.0459	0.5176	12 / 12
25023		0.0018	0.0246	0.0100	0.4979	13 / 13
25024		-0.0019	-0.0043	-0.0208	0.3311	13 / 13
25029		0.0151	-0.0225	-0.0684	0.4072	15 / 15
25030		-0.0066	-0.0303	0.0024	0.4579	14 / 14
25032		-0.0637	-0.0373	-0.0404	0.5397	11 / 11
25033		-0.0195	-0.0407	-0.0328	0.3330	14 / 14
25034		-0.0100	-0.0378	-0.0183	0.3736	13 / 13
40000		-0.0087	-0.0094	-0.0926	0.2450	14 / 14
40001		0.0052	0.0327	-0.0187	0.2964	15 / 15
40002		0.0135	0.0559	-0.0473	0.3743	13 / 13
40003		0.0064	0.0570	0.0046	0.2859	12 / 12
40004		0.0028	0.0096	0.0002	0.3020	12 / 12

40005		0.0264	0.0311	-0.0034	0.2915	6 / 6
45000		0.0200	-0.0353	0.0261	0.3280	15 / 15
45001		-0.0178	-0.0018	0.0042	0.2626	9 / 9
45002		0.0239	-0.0556	0.0014	0.5503	13 / 13
10043		-0.0112	-0.0113	0.0416	0.6791	12 / 12
10084		-0.0290	-0.0014	0.0252	0.5586	9 / 9
20015		-0.0102	-0.0130	-0.1022	0.2600	14 / 14
20007		-0.0095	-0.0081	-0.0055	0.4894	10 / 10
25027		-0.0152	-0.0103	-0.0813	0.3641	15 / 15
k9		0.0057	-0.0118	-0.0445	0.4486	14 / 14
6600		0.0018	-0.0062	-0.0677	0.3492	11 / 11
6602		-0.0030	0.0422	-0.0272	0.4340	13 / 13
6603		-0.0118	-0.0031	-0.0318	0.5426	13 / 13
6605		0.0153	0.0256	0.0021	0.4214	13 / 13
6606		0.0167	0.0286	0.0194	0.7201	10 / 10
6607		0.0216	0.0276	-0.0669	0.4025	12 / 12
6608		0.0022	-0.0232	-0.0889	0.7141	7 / 7
6609		-0.0283	0.0075	-0.0598	0.2877	15 / 15
6610		-0.0214	0.0172	0.0081	0.7091	8 / 8
6611		-0.0281	-0.0245	-0.1138	0.5343	13 / 13
6612		-0.0053	-0.0494	-0.1061	1.0208	13 / 13
6616		-0.0302	-0.0253	0.0001	0.3373	10 / 10
6617		-0.0080	-0.0177	0.0268	0.5752	8 / 8
6618		-0.0030	-0.0214	-0.0063	0.4040	13 / 13
6619		0.0136	-0.0137	0.0036	0.1945	13 / 13
6620		0.0130	-0.0622	-0.0248	0.3379	14 / 14
6621		-0.0124	-0.0260	-0.0338	0.2622	14 / 14
6622		-0.0083	0.0295	-0.0175	0.5806	11 / 11
6623		0.0024	-0.0078	-0.0155	0.7376	11 / 11
6624		0.0535	-0.0059	-0.0275	0.8226	10 / 10
6625		-0.0917	-0.0095	0.0214	0.5250	10 / 10
6626		-0.0533	0.0016	-0.0275	0.3003	11 / 11
6627		-0.0670	-0.0138	0.0134	0.6700	9 / 9
6628		-0.0656	-0.0154	-0.0074	0.4724	12 / 12
6629		-0.0808	-0.0127	0.0016	0.4442	12 / 12
6630		-0.0939	0.0017	0.0116	0.3680	13 / 13
6631		-0.0629	-0.0188	0.0244	0.4234	11 / 11
6632		-0.0709	-0.0179	0.0450	0.5225	10 / 10
6633		-0.0296	-0.0050	0.0243	0.3299	15 / 15
6634		-0.0389	0.0101	0.0282	0.4213	14 / 14
6635		-0.0276	0.0103	0.0382	0.3976	15 / 15
6636		-0.0769	-0.0192	0.0360	0.3673	13 / 13
6637		-0.0472	0.0150	0.0632	0.4673	10 / 10
6638		-0.0519	0.0063	0.0488	0.3194	10 / 10
6639		-0.0795	0.0188	0.0542	0.4542	12 / 12
6640		-0.0563	0.0012	0.0558	0.5460	10 / 10
6641		-0.0533	0.0006	0.0571	0.4484	12 / 12
**Mean [m]**		-0.007459	-0.001100	-0.005633		
**Sigma [m]**		0.028235	0.030630	0.047287		
**RMS Error [m]**		0.029204	0.030650	0.047621		

Localisation accuracy per GCP and mean errors in the three coordinate directions. The last column counts the number of calibrated images where the GCP has been automatically verified vs. manually marked.

Absolute Geolocation Variance

Min Error [m]	Max Error [m]	Geolocation Error X [%]	Geolocation Error Y [%]	Geolocation Error Z [%]

-	-15.00	0.00	0.00	0.00
-15.00	-12.00	0.00	0.00	0.00
-12.00	-9.00	0.00	0.00	0.00
-9.00	-6.00	0.00	0.00	0.00
-6.00	-3.00	0.00	6.83	10.58
-3.00	0.00	47.10	43.34	38.23
0.00	3.00	52.90	41.30	43.69
3.00	6.00	0.00	8.53	7.51
6.00	9.00	0.00	0.00	0.00
9.00	12.00	0.00	0.00	0.00
12.00	15.00	0.00	0.00	0.00
15.00	-	0.00	0.00	0.00
**Mean [m]**		0.925433	1.651269	32.612015
**Sigma [m]**		1.022034	2.153651	2.156984
**RMS Error [m]**		1.378760	2.713835	32.683270

Min Error and Max Error represent geolocation error intervals between -1.5 and 1.5 times the maximum accuracy of all the images. Columns X, Y, Z show the percentage of images with geolocation errors within the predefined error intervals. The geolocation error is the difference between the initial and computed image positions. Note that the image geolocation errors do not correspond to the accuracy of the observed 3D points.

Geolocation Bias	X	Y	Z
Translation [m]	0.925433	1.651269	32.612015

Bias between image initial and computed geolocation given in output coordinate system.

Relative Geolocation Variance

Relative Geolocation Error	Images X [%]	Images Y [%]	Images Z [%]
[-1.00, 1.00]	100.00	100.00	100.00
[-2.00, 2.00]	100.00	100.00	100.00
[-3.00, 3.00]	100.00	100.00	100.00
**Mean of Geolocation Accuracy [m]**	5.000000	5.000000	10.000000
**Sigma of Geolocation Accuracy [m]**	0.000000	0.000000	0.000000

Images X, Y, Z represent the percentage of images with a relative geolocation error in X, Y, Z.

Initial Processing Details

System Information

Hardware	CPU: Intel(R) Core(TM) i9-7940X CPU @ 3.10GHz<br>RAM: 128GB<br>GPU: NVIDIA GeForce GTX 1080 Ti (Driver: 26.21.14.3615), RDPDD Chained DD (Driver: unknown), RDP Encoder Mirror Driver (Driver: unknown), RDP Reflector Display Driver (Driver: unknown)
Operating System	Windows 7 Ultimate, 64-bit

Coordinate Systems

Image Coordinate System	WGS 84 (EGM 96 Geoid)
Ground Control Point (GCP) Coordinate System	ETRS89 / Poland CS2000 zone 6 (EGM 96 Geoid)
Output Coordinate System	ETRS89 / Poland CS2000 zone 6 (EGM 96 Geoid)

Processing Options

Detected Template	3D Maps

Keypoints Image Scale	Full, Image Scale: 1
Advanced: Matching Image Pairs	Aerial Grid or Corridor
Advanced: Matching Strategy	Use Geometrically Verified Matching: no
Advanced: Keypoint Extraction	Targeted Number of Keypoints: Automatic
Advanced: Calibration	Calibration Method: Standard<br>Internal Parameters Optimization: All<br>External Parameters Optimization: All<br>Rematch: Auto, yes
