# Supplementary material for: Multi-Variant Accuracy Evaluation of UAV Imaging Surveys: A Case Study on Investment Area
Source: Sensors (Basel). 2019 Nov 28;19(23):5229. doi: 10.3390/s19235229 (PMC6929115; doi:10.3390/s19235229)
Supplement: Supplementary file 1 [file sensors-19-05229-s001.zip › supplementary_files/App13_V6_p85q45_noGPS_report.pdf]

Quality Report

Generated with Pix4Ddiscovery version 4.3.31

Important: Click on the different icons for:

Help to analyze the results in the Quality Report

Additional information about the sections

Click here for additional tips to analyze the Quality Report

Summary

Project	V6_p85q45_noGPS
Processed	2019-10-12 21:33:35
Camera Model Name(s)	NEX-5T_0.0_4912x3264 (RGB)
Average Ground Sampling Distance (GSD)	4.58 cm / 1.80 in
Area Covered	0.969 km^2 / 96.8976 ha / 0.37 sq. mi. / 239.5632 acres
Time for Initial Processing (without report)	16m:19s

Quality Check

**Images**	median of 45820 keypoints per image	✓
**Dataset**	293 out of 293 images calibrated (100%), all images enabled	✓
**Camera Optimization**	0.03% relative difference between initial and optimized internal camera parameters	✓
**Matching**	median of 21946.1 matches per calibrated image	✓
**Georeferencing**	yes, 11 GCPs (11 3D), mean RMS error = 0.021 m	✓

Preview

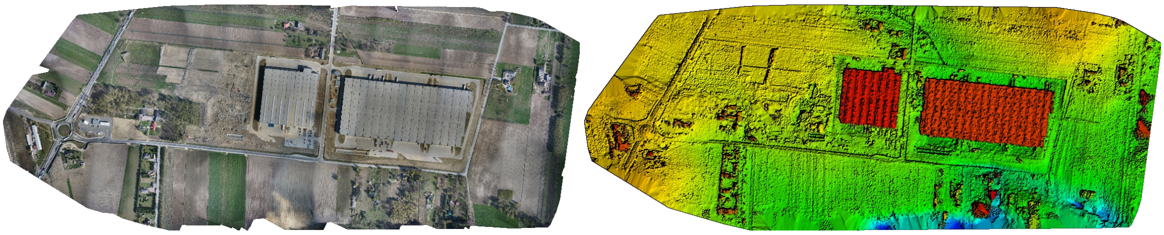

Figure 1: Orthomosaic and the corresponding sparse Digital Surface Model (DSM) before densification.

Calibration Details

Number of Calibrated Images	293 out of 293
Number of Geolocated Images	0 out of 293

Initial Image Positions

The preview is not generated for images without geolocation.

Computed Image/GCPs/Manual Tie Points Positions

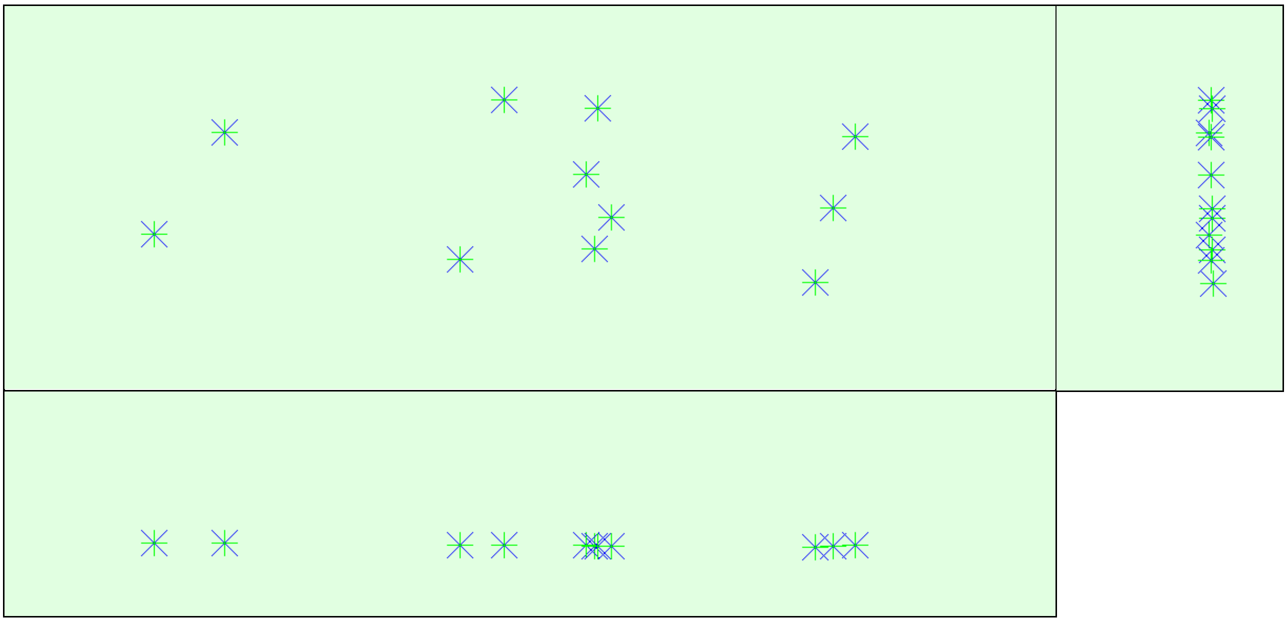

Uncertainty ellipses 500x magnified

Figure 3: Offset between initial (blue dots) and computed (green dots) image positions as well as the offset between the GCPs initial positions (blue crosses) and their computed positions (green crosses) in the top-view (XY plane), front-view (XZ plane), and side-view (YZ plane). Dark green ellipses indicate the absolute position uncertainty of the bundle block adjustment result.

Absolute camera position and orientation uncertainties

	X [m]	Y [m]	Z [m]	Omega [degree]	Phi [degree]	Kappa [degree]
Mean	0.017	0.021	0.036	0.007	0.006	0.002
Sigma	0.004	0.005	0.001	0.002	0.002	0.001

Overlap

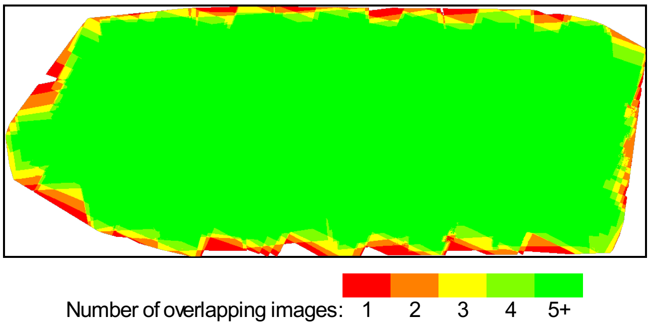

**Figure 4: Number of overlapping images computed for each pixel of the orthomosaic.
Red and yellow areas indicate low overlap for which poor results may be generated. Green areas indicate an overlap of over 5 images for every pixel. Good quality results will be generated as long as the number of keypoint matches is also sufficient for these areas (see Figure 5 for keypoint matches).**

Bundle Block Adjustment Details

Number of 2D Keypoint Observations for Bundle Block Adjustment	6504123
Number of 3D Points for Bundle Block Adjustment	2148307
Mean Reprojection Error [pixels]	0.189

Internal Camera Parameters

NEX-5T_0.0_4912x3264 (RGB). Sensor Dimensions: 23.400 [mm] x 15.549 [mm]

EXIF ID: NEX-5T_0.0_4912x3264

	Focal Length	Principal Point x	Principal Point y	R1	R2	R3	T1	T2
Initial Values	3256.554 [pixel] 15.514 [mm]	2456.002 [pixel] 11.700 [mm]	1632.003 [pixel] 7.775 [mm]	-0.043	0.028	-0.005	0.000	-0.001
Optimized Values	3255.510 [pixel] 15.509 [mm]	2466.799 [pixel] 11.751 [mm]	1589.928 [pixel] 7.574 [mm]	-0.047	0.035	-0.010	0.001	0.000
Uncertainties (Sigma)	0.766 [pixel] 0.004 [mm]	0.134 [pixel] 0.001 [mm]	0.132 [pixel] 0.001 [mm]	0.000	0.000	0.000	0.000	0.000

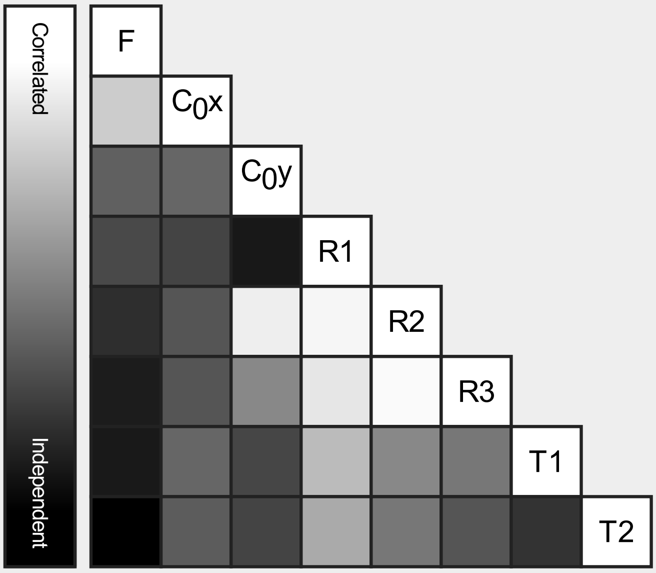

The correlation between camera internal parameters determined by the bundle adjustment. White indicates a full correlation between the parameters, ie. any change in one can be fully compensated by the other. Black indicates that the parameter is completely independent, and is not affected by other parameters.

The number of Automatic Tie Points (ATPs) per pixel, averaged over all images of the camera model, is color coded between black and white. White indicates that, on average, more than 16 ATPs have been extracted at the pixel location. Black indicates that, on average, 0 ATPs have been extracted at the pixel location. Click on the image to the see the average direction and magnitude of the re-projection error for each pixel. Note that the vectors are scaled for better visualization. The scale bar indicates the magnitude of 1 pixel error.

2D Keypoints Table

	Number of 2D Keypoints per Image	Number of Matched 2D Keypoints per Image
Median	45820	21946
Min	20057	6134
Max	68781	40208
Mean	42855	22198

3D Points from 2D Keypoint Matches

	Number of 3D Points Observed
In 2 Images	1233443
In 3 Images	406999
In 4 Images	202893
In 5 Images	118550
In 6 Images	75062
In 7 Images	43623
In 8 Images	23645
In 9 Images	14625
In 10 Images	10576
In 11 Images	7580
In 12 Images	5197
In 13 Images	3315

In 14 Images	1744
In 15 Images	680
In 16 Images	247
In 17 Images	85
In 18 Images	33
In 19 Images	7
In 20 Images	2
In 21 Images	1

2D Keypoint Matches

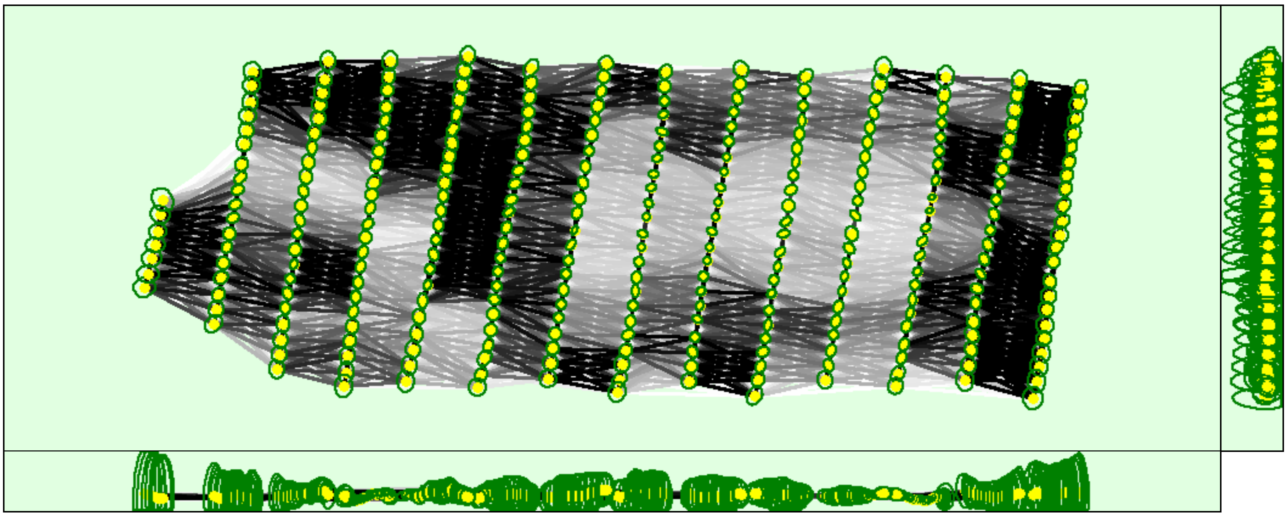

Uncertainty ellipses 500x magnified

Number of matches

25 222 444 666 888 1111 1333 1555 1777 2000

Figure 5: Computed image positions with links between matched images. The darkness of the links indicates the number of matched 2D keypoints between the images. Bright links indicate weak links and require manual tie points or more images. Dark green ellipses indicate the relative camera position uncertainty of the bundle block adjustment result.

Relative camera position and orientation uncertainties

	X[m]	Y[m]	Z[m]	Omega [degree]	Phi [degree]	Kappa [degree]
Mean	0.019	0.021	0.050	0.012	0.015	0.003
Sigma	0.004	0.005	0.029	0.005	0.007	0.001

Geolocation Details

Ground Control Points

GCP Name	Accuracy XY/Z [m]	Error X [m]	Error Y [m]	Error Z [m]	Projection Error [pixel]	Verified/Marked
201 (3D)	0.030/ 0.050	-0.006	-0.021	0.016	0.519	11 / 11
202 (3D)	0.030/ 0.050	-0.001	0.017	-0.008	0.549	10 / 10
203 (3D)	0.030/ 0.050	-0.008	0.026	-0.028	0.685	13 / 13
208 (3D)	0.030/ 0.050	0.001	-0.005	-0.030	0.519	12 / 12
209 (3D)	0.030/ 0.050	0.007	-0.013	-0.013	0.368	13 / 13
207 (3D)	0.030/ 0.050	-0.031	-0.003	0.072	0.331	11 / 11
211 (3D)	0.030/ 0.050	0.019	0.024	-0.008	0.440	13 / 13
210 (3D)	0.030/ 0.050	0.006	-0.009	0.008	0.240	13 / 13
206 (3D)	0.030/ 0.050	0.013	0.018	0.016	0.294	14 / 14
205 (3D)	0.030/ 0.050	-0.023	-0.050	-0.001	0.778	15 / 15

204 (3D)	0.030/ 0.050	0.020	0.020	-0.019	0.813	11 / 12
**Mean [m]**		-0.000229	0.000083	0.000284		
**Sigma [m]**		0.015430	0.022410	0.027128		
**RMS Error [m]**		0.015432	0.022410	0.027129		

0 out of 207 check points have been labeled as inaccurate.

Check Point Name	Accuracy XY/Z [m]	Error X [m]	Error Y [m]	Error Z [m]	Projection Error [pixel]	Verified/Marked
103		0.0087	0.0289	-0.0173	0.4816	12 / 12
101		-0.0069	0.0149	0.0171	0.3373	13 / 13
100		-0.0088	-0.0207	0.0467	0.2990	12 / 12
104		-0.0485	0.0103	-0.0130	0.4450	13 / 13
102		-0.0187	-0.0314	0.0107	0.3304	14 / 14
k1		0.0406	-0.0434	0.1194	0.4539	14 / 14
k2		0.0068	0.0265	-0.0300	0.4550	14 / 14
k3		0.0989	-0.0787	0.1622	0.6479	13 / 13
k4		0.0347	-0.0252	0.0844	0.3841	12 / 12
k5		-0.0144	0.0273	-0.0363	0.4951	13 / 13
k6		-0.0281	0.0229	0.0590	0.4904	10 / 10
k7		-0.0869	0.0105	0.1637	0.4885	8 / 8
k8		-0.0486	-0.0304	0.1589	0.5002	12 / 12
k11		-0.0076	-0.0404	-0.1256	0.5655	15 / 15
k13		0.0002	0.0076	-0.0063	0.3865	12 / 12
k14		0.0081	-0.0010	-0.0144	0.4297	13 / 13
10000		-0.0192	-0.0169	0.0243	0.4872	12 / 12
10001		-0.0004	0.0017	0.0722	0.6290	12 / 12
10002		0.0095	-0.0299	0.0229	0.2759	11 / 11
10003		-0.0037	-0.0431	-0.0026	0.5967	15 / 15
10004		-0.0006	-0.0066	0.0779	0.4819	12 / 12
10005		0.0098	-0.0109	0.0792	0.5558	13 / 13
10006		-0.0041	-0.0186	0.0683	0.3628	13 / 13
10007		0.0227	-0.0206	0.0365	0.2887	14 / 14
10008		0.0311	-0.0332	0.0255	0.3250	16 / 16
10009		0.0190	-0.0383	0.0156	0.4518	14 / 14
10010		0.0240	-0.0445	0.0075	0.3671	13 / 13
10011		0.0156	-0.0497	-0.0056	0.5376	12 / 12
10012		-0.0463	-0.0007	0.0349	0.4223	15 / 15
10013		-0.0252	-0.0143	0.0037	0.3828	13 / 13
10014		0.0034	-0.0004	-0.0040	0.4031	13 / 13
10015		0.0121	-0.0162	0.0010	0.4670	15 / 15
10016		-0.0196	-0.0199	-0.0231	0.3603	13 / 13
10017		0.0113	-0.0308	-0.0707	0.5034	14 / 14
10018		0.0115	-0.0113	-0.0233	0.3287	6 / 6
10019		-0.0149	0.0178	0.0422	0.6748	14 / 14
10020		-0.0353	0.0095	0.0144	0.4524	13 / 13
10021		-0.0063	0.0030	-0.0034	0.3817	18 / 18
10022		0.0169	0.0410	-0.0565	0.5390	15 / 15
10023		0.0168	0.0075	-0.0689	0.4359	12 / 12
10024		-0.0118	0.0098	-0.0810	0.4475	18 / 18
10025		0.0030	0.0126	-0.0545	0.3327	16 / 16
10026		-0.0126	0.0301	-0.0579	0.4059	13 / 13
10027		-0.0088	0.0280	-0.0415	0.4820	14 / 14
10028		0.0007	0.0119	-0.0287	0.4480	15 / 15
10030		-0.0147	0.0456	-0.0182	0.3482	13 / 13
10031		0.0158	0.0421	0.0348	0.5261	7 / 7
10032		0.0441	0.0641	-0.0157	0.4221	6 / 6
10033		0.0015	0.0350	0.0190	0.3676	15 / 15
10034		0.0221	-0.0040	0.0067	0.3589	13 / 13
10035		0.0079	-0.0236	0.0613	0.3289	11 / 11
10036		0.0057	-0.0046	0.0024	0.5980	9 / 9
10037		-0.0171	-0.0001	-0.0093	0.3274	11 / 11

10038		-0.0160	0.0139	-0.0010	0.3213	14 / 14
10039		0.0071	-0.0094	-0.0136	0.3986	15 / 15
10040		-0.0210	-0.0036	0.0055	0.3243	11 / 11
10041		-0.0101	-0.0194	0.0067	0.4788	14 / 14
10044		0.0062	-0.0454	-0.0268	0.3455	12 / 12
10045		0.0156	-0.0342	-0.0562	0.3616	14 / 14
10046		-0.0245	-0.0181	-0.0717	0.3852	13 / 13
10047		-0.0431	-0.0153	0.0832	0.5013	12 / 12
10048		-0.0269	-0.0343	0.0748	0.6910	14 / 14
10049		-0.0187	0.0196	0.0298	0.7578	14 / 14
10051		0.0042	0.0238	0.0023	0.3140	14 / 14
10052		0.0058	-0.0038	0.0391	0.4887	13 / 13
10053		-0.0174	-0.0032	-0.0306	0.4645	13 / 13
10054		-0.0071	-0.0333	-0.0270	0.4999	13 / 13
10055		-0.0142	0.0110	-0.0685	0.5590	11 / 11
10056		-0.0544	0.0120	0.0050	0.4433	12 / 12
10058		-0.0811	0.0844	0.0396	0.4779	15 / 15
10059		0.0056	-0.0300	-0.0175	0.3437	15 / 15
10060		-0.0248	-0.0438	-0.0267	0.3419	15 / 15
10061		0.0222	0.0031	-0.0103	0.4255	9 / 9
10062		0.0031	-0.0490	-0.0400	0.4168	15 / 15
10063		0.0200	-0.0656	-0.0551	0.5288	14 / 14
10064		0.0301	0.0309	-0.0524	0.4511	15 / 15
10065		0.0286	0.0397	-0.0114	0.3332	12 / 12
10066		0.0624	0.0409	-0.0554	0.3641	11 / 11
10067		0.0291	0.0472	-0.0381	0.4084	12 / 12
10068		0.0083	0.1168	-0.0241	0.4705	14 / 14
10074		-0.0175	0.0511	-0.0183	0.5470	13 / 13
10075		-0.0208	0.0538	0.0225	0.3087	13 / 13
10076		-0.0008	0.0357	-0.0115	0.4216	13 / 13
10077		-0.0307	0.0676	-0.0405	0.6310	12 / 12
10078		-0.0038	0.0304	-0.0254	0.2192	11 / 11
10080		0.0164	-0.0642	-0.0912	0.7917	14 / 14
10081		-0.0243	-0.0350	0.0133	0.3674	15 / 15
10082		0.0104	-0.0428	-0.0271	0.3952	16 / 16
10083		0.0478	-0.0026	-0.0915	0.5120	13 / 13
10085		0.0399	-0.0550	-0.0139	0.3235	15 / 15
15000		0.0036	-0.0427	0.0211	0.2747	14 / 14
15001		-0.0150	-0.0450	0.0180	0.3397	14 / 14
15002		-0.0121	0.0301	-0.0895	0.3110	14 / 14
15003		-0.0276	0.0475	-0.0197	0.3424	16 / 16
15004		-0.0174	0.0185	-0.0269	0.2972	14 / 14
15005		-0.0060	0.0258	-0.0396	0.7040	11 / 11
15006		0.0115	0.0372	-0.0331	0.3410	13 / 13
15007		0.0202	-0.0222	-0.0159	0.2732	11 / 11
15008		-0.0253	0.0086	0.0774	0.8047	11 / 11
15009		0.0046	-0.0007	0.0129	0.4706	13 / 13
15010		0.0126	0.0382	0.0311	0.5889	12 / 12
20000		0.0009	0.0198	0.0427	0.6195	13 / 13
20001		-0.0037	-0.0057	0.0341	0.3463	11 / 11
20002		0.0098	0.0065	0.0104	0.2895	12 / 12
20003		-0.0188	-0.0146	0.0023	0.3876	13 / 13
20004		-0.0024	-0.0139	0.0205	0.3947	12 / 12
20005		-0.0649	-0.0439	0.0159	0.3421	13 / 13
20008		-0.0050	-0.0146	-0.0113	0.3772	14 / 14
20009		0.0178	-0.0288	-0.0483	0.4322	16 / 16
20010		-0.0287	0.0304	-0.0720	0.3835	16 / 16
20011		-0.0187	0.0094	-0.0547	0.3568	19 / 19
20012		0.0072	0.0030	-0.0658	0.3669	15 / 15

20013		0.0125	0.0059	-0.0314	0.3767	12 / 12
20014		0.0030	0.0152	-0.0317	0.3577	14 / 14
20016		-0.0062	0.0202	-0.1015	0.2480	17 / 17
20017		0.0007	0.0333	-0.0857	0.2942	13 / 13
20018		0.0165	0.0268	-0.0636	0.3352	14 / 14
20019		0.0264	0.0256	-0.0307	0.3186	13 / 13
20020		0.0047	0.0491	-0.0027	0.4327	13 / 13
20021		-0.0041	0.0068	0.0161	0.2213	14 / 14
20022		-0.0268	-0.0437	0.0161	0.2440	14 / 14
20023		-0.0178	0.0097	-0.0111	0.2401	14 / 14
20024		-0.0073	0.0507	-0.0155	0.4197	13 / 13
20025		0.0079	0.0543	-0.0130	0.2876	13 / 13
20026		0.0057	0.0491	0.0024	0.3595	14 / 14
20027		-0.0246	0.0864	-0.0282	0.4155	11 / 11
20028		-0.0225	0.0224	-0.0124	0.3572	12 / 12
20029		0.0123	-0.0286	-0.0399	0.6743	15 / 15
20031		-0.0263	0.0231	-0.0616	0.5115	14 / 14
25000		0.0119	0.0128	-0.0258	0.2371	15 / 15
25001		0.0075	-0.0013	-0.0012	0.2112	11 / 11
25003		0.0040	-0.0200	0.0193	0.3348	13 / 13
25004		0.0040	-0.0010	0.0003	0.3064	12 / 12
25005		0.0057	-0.0129	0.0316	0.2877	13 / 13
25006		-0.0349	-0.0354	-0.0539	0.4185	13 / 13
25008		-0.0168	-0.0042	0.0188	0.1849	13 / 13
25009		0.0394	-0.0506	0.0095	0.2806	16 / 16
25010		0.0169	-0.0198	0.0010	0.2553	16 / 16
25013		-0.0064	0.0073	-0.0293	0.3373	12 / 12
25014		-0.0191	-0.0201	-0.0362	0.2677	13 / 13
25015		-0.0286	-0.0227	-0.0319	0.3542	13 / 13
25016		-0.0320	-0.0182	-0.0264	0.1874	13 / 13
25017		0.0248	-0.0388	0.0407	0.3445	12 / 12
25018		0.0026	-0.0125	0.0243	0.3865	13 / 13
25019		-0.0211	-0.0263	0.0273	0.4327	13 / 13
25020		-0.0183	-0.0126	-0.0361	0.2560	8 / 8
25021		0.0062	0.0186	0.0217	0.3319	13 / 13
25022		-0.0011	0.0026	0.0441	0.5173	12 / 12
25023		0.0016	0.0248	0.0084	0.4971	13 / 13
25024		-0.0022	-0.0032	-0.0208	0.3318	13 / 13
25029		0.0162	-0.0222	-0.0671	0.4062	15 / 15
25030		-0.0057	-0.0307	0.0024	0.4613	14 / 14
25032		-0.0628	-0.0376	-0.0405	0.5384	11 / 11
25033		-0.0189	-0.0407	-0.0334	0.3324	14 / 14
25034		-0.0097	-0.0375	-0.0190	0.3756	13 / 13
40000		-0.0080	-0.0087	-0.0911	0.2461	14 / 14
40001		0.0067	0.0334	-0.0165	0.2932	15 / 15
40002		0.0138	0.0570	-0.0439	0.3781	13 / 13
40003		0.0065	0.0580	0.0068	0.2787	12 / 12
40004		0.0028	0.0106	0.0007	0.3004	12 / 12
40005		0.0266	0.0323	-0.0032	0.2950	6 / 6
45000		0.0207	-0.0357	0.0253	0.3271	15 / 15
45001		-0.0172	-0.0018	0.0033	0.2617	9 / 9
45002		0.0247	-0.0559	0.0014	0.5465	13 / 13
10043		-0.0112	-0.0121	0.0413	0.6798	12 / 12
10084		-0.0287	-0.0018	0.0252	0.5546	9 / 9
20015		-0.0108	-0.0126	-0.1024	0.2590	14 / 14
20007		-0.0096	-0.0078	-0.0071	0.4890	10 / 10
25027		-0.0165	-0.0098	-0.0839	0.3617	15 / 15
k9		0.0054	-0.0110	-0.0443	0.4460	14 / 14
6600		0.0021	-0.0050	-0.0646	0.3503	11 / 11

6602		-0.0033	0.0434	-0.0270	0.4343	13 / 13
6603		-0.0121	-0.0021	-0.0329	0.5421	13 / 13
6605		0.0152	0.0261	0.0004	0.4190	13 / 13
6606		0.0167	0.0287	0.0183	0.7182	10 / 10
6607		0.0212	0.0285	-0.0650	0.4025	12 / 12
6608		0.0016	-0.0227	-0.0915	0.7154	7 / 7
6609		-0.0292	0.0080	-0.0620	0.2898	15 / 15
6610		-0.0228	0.0175	0.0048	0.7128	8 / 8
6611		-0.0304	-0.0245	-0.1187	0.5327	13 / 13
6612		-0.0077	-0.0494	-0.1110	1.0228	13 / 13
6616		-0.0296	-0.0266	-0.0008	0.3408	10 / 10
6617		-0.0074	-0.0180	0.0257	0.5757	8 / 8
6618		-0.0022	-0.0217	-0.0069	0.4041	13 / 13
6619		0.0144	-0.0142	0.0031	0.1939	13 / 13
6620		0.0139	-0.0627	-0.0250	0.3370	14 / 14
6621		-0.0114	-0.0259	-0.0325	0.2622	14 / 14
6622		-0.0095	0.0299	-0.0212	0.5879	11 / 11
6623		0.0018	-0.0077	-0.0208	0.7430	11 / 11
6624		0.0537	-0.0053	-0.0343	0.8272	10 / 10
6625		-0.0901	-0.0095	0.0139	0.5246	10 / 10
6626		-0.0518	0.0017	-0.0354	0.2970	11 / 11
6627		-0.0657	-0.0137	0.0062	0.6710	9 / 9
6628		-0.0643	-0.0154	-0.0138	0.4711	12 / 12
6629		-0.0796	-0.0128	-0.0044	0.4444	12 / 12
6630		-0.0929	0.0016	0.0061	0.3662	13 / 13
6631		-0.0620	-0.0189	0.0191	0.4241	11 / 11
6632		-0.0702	-0.0185	0.0418	0.5224	10 / 10
6633		-0.0287	-0.0054	0.0210	0.3309	15 / 15
6634		-0.0378	0.0098	0.0241	0.4229	14 / 14
6635		-0.0263	0.0100	0.0333	0.3976	15 / 15
6636		-0.0752	-0.0193	0.0303	0.3651	13 / 13
6637		-0.0454	0.0148	0.0574	0.4686	10 / 10
6638		-0.0502	0.0062	0.0433	0.3197	10 / 10
6639		-0.0777	0.0187	0.0484	0.4532	12 / 12
6640		-0.0545	0.0010	0.0508	0.5482	10 / 10
6641		-0.0516	0.0002	0.0533	0.4485	12 / 12
**Mean [m]**		-0.007171	-0.000960	-0.006623		
**Sigma [m]**		0.028066	0.030987	0.046530		
**RMS Error [m]**		0.028968	0.031002	0.046999		

Localisation accuracy per GCP and mean errors in the three coordinate directions. The last column counts the number of calibrated images where the GCP has been automatically verified vs. manually marked.

Initial Processing Details

System Information

Hardware	CPU: Intel(R) Core(TM) i9-7940X CPU @ 3.10GHz<br>RAM: 128GB<br>GPU: NVIDIA GeForce GTX 1080 Ti (Driver: 26.21.14.3615), RDPDD Chained DD (Driver: unknown), RDP Encoder Mirror Driver (Driver: unknown), RDP Reflector Display Driver (Driver: unknown)
Operating System	Windows 7 Ultimate, 64-bit

Coordinate Systems

Ground Control Point (GCP) Coordinate System	ETRS89 / Poland CS2000 zone 6 (EGM96 Geoid)
Output Coordinate System	ETRS89 / Poland CS2000 zone 6 (EGM96 Geoid)

Processing Options

Detected Template	3D Maps
Keypoints Image Scale	Full, Image Scale: 1
Advanced: Matching Image Pairs	Aerial Grid or Corridor
Advanced: Matching Strategy	Use Geometrically Verified Matching: no
Advanced: Keypoint Extraction	Targeted Number of Keypoints: Automatic
Advanced: Calibration	Calibration Method: Standard<br>Internal Parameters Optimization: All<br>External Parameters Optimization: All<br>Rematch: Auto, yes
