# Supplementary material for: Multi-Variant Accuracy Evaluation of UAV Imaging Surveys: A Case Study on Investment Area
Source: Sensors (Basel). 2019 Nov 28;19(23):5229. doi: 10.3390/s19235229 (PMC6929115; doi:10.3390/s19235229)
Supplement: Supplementary file 1 [file sensors-19-05229-s001.zip › supplementary_files/App14_V6_report_noGPS.pdf]

Quality Report

Generated with Pix4Ddiscovery version 4.3.31

Important: Click on the different icons for:

Help to analyze the results in the Quality Report

Additional information about the sections

Click here for additional tips to analyze the Quality Report

Summary

Project	Sosnowiec_2_new_ver_11GCP_noGPS
Processed	2019-10-17 10:40:05
Camera Model Name(s)	NEX-5T_0.0_4912x3264 (RGB)
Average Ground Sampling Distance (GSD)	4.59 cm / 1.81 in
Area Covered	1.012 km^2 / 101.1952 ha / 0.39 sq. mi. / 250.1883 acres
Time for Initial Processing (without report)	29m:54s

Quality Check

**Images**	median of 45097 keypoints per image	✓
**Dataset**	858 out of 858 images calibrated (100%), all images enabled	✓
**Camera Optimization**	0.22% relative difference between initial and optimized internal camera parameters	✓
**Matching**	median of 12009.4 matches per calibrated image	✓
**Georeferencing**	yes, 11 GCPs (11 3D), mean RMS error = 0.02 m	✓

Preview

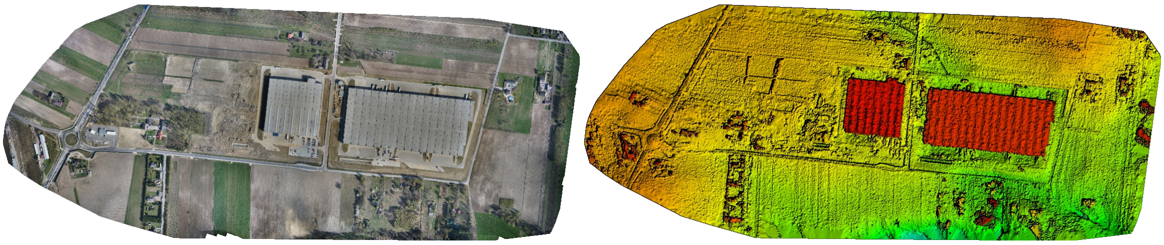

Figure 1: Orthomosaic and the corresponding sparse Digital Surface Model (DSM) before densification.

Calibration Details

Number of Calibrated Images	858 out of 858
Number of Geolocated Images	0 out of 858

Initial Image Positions

The preview is not generated for images without geolocation.

Computed Image/GCPs/Manual Tie Points Positions

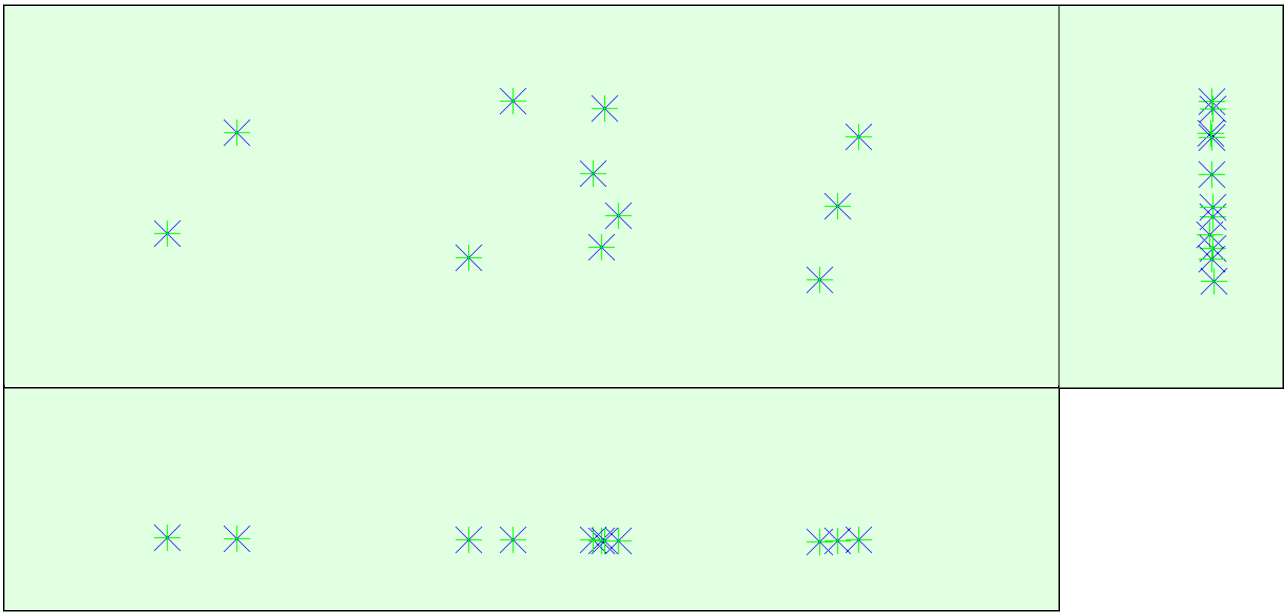

Uncertainty ellipses 500x magnified

Figure 3: Offset between initial (blue dots) and computed (green dots) image positions as well as the offset between the GCPs initial positions (blue crosses) and their computed positions (green crosses) in the top-view (XY plane), front-view (XZ plane), and side-view (YZ plane). Dark green ellipses indicate the absolute position uncertainty of the bundle block adjustment result.

Absolute camera position and orientation uncertainties

	X [m]	Y [m]	Z [m]	Omega [degree]	Phi [degree]	Kappa [degree]
Mean	0.018	0.023	0.027	0.008	0.006	0.002
Sigma	0.006	0.007	0.002	0.002	0.002	0.001

Overlap

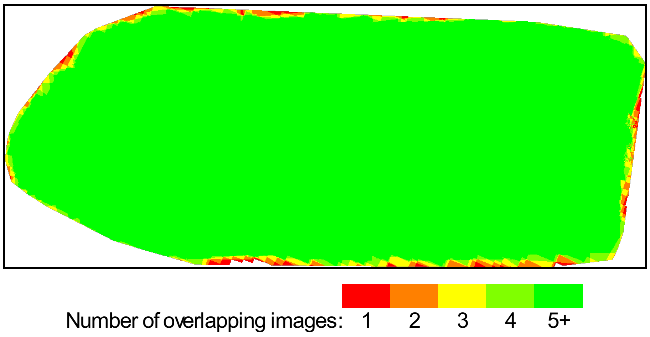

Figure 4: Number of overlapping images computed for each pixel of the orthomosaic.
Red and yellow areas indicate low overlap for which poor results may be generated. Green areas indicate an overlap of over 5 images for every pixel. Good quality results will be generated as long as the number of keypoint matches is also sufficient for these areas (see Figure 5 for keypoint matches).

Bundle Block Adjustment Details

Number of 2D Keypoint Observations for Bundle Block Adjustment	10438926
Number of 3D Points for Bundle Block Adjustment	3627708
Mean Reprojection Error [pixels]	0.145

Internal Camera Parameters

NEX-5T_0.0_4912x3264 (RGB). Sensor Dimensions: 23.400 [mm] x 15.549 [mm]

EXIF ID: NEX-5T_0.0_4912x3264

	Focal Length	Principal Point x	Principal Point y	R1	R2	R3	T1	T2
Initial Values	3256.554 [pixel] 15.514 [mm]	2456.002 [pixel] 11.700 [mm]	1632.003 [pixel] 7.775 [mm]	-0.043	0.028	-0.005	0.000	-0.001
Optimized Values	3249.359 [pixel] 15.479 [mm]	2467.033 [pixel] 11.753 [mm]	1590.101 [pixel] 7.575 [mm]	-0.047	0.035	-0.010	0.000	0.000
Uncertainties (Sigma)	0.547 [pixel] 0.003 [mm]	0.082 [pixel] 0.000 [mm]	0.083 [pixel] 0.000 [mm]	0.000	0.000	0.000	0.000	0.000

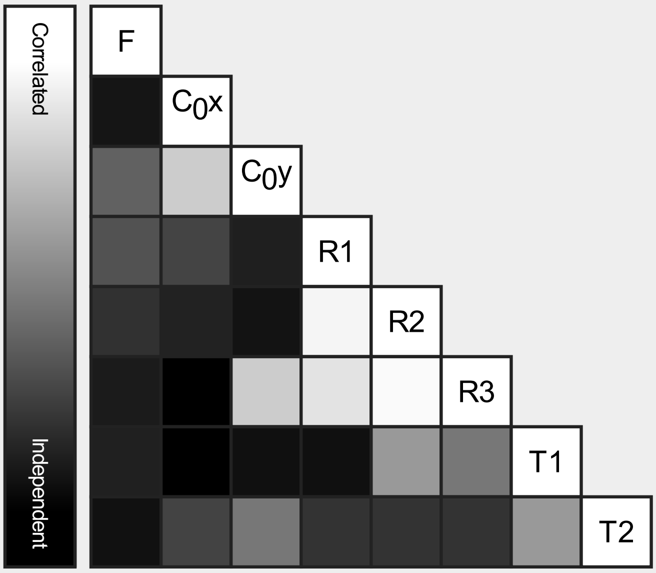

The correlation between camera internal parameters determined by the bundle adjustment. White indicates a full correlation between the parameters, ie. any change in one can be fully compensated by the other. Black indicates that the parameter is completely independent, and is not affected by other parameters.

The number of Automatic Tie Points (ATPs) per pixel, averaged over all images of the camera model, is color coded between black and white. White indicates that, on average, more than 16 ATPs have been extracted at the pixel location. Black indicates that, on average, 0 ATPs have been extracted at the pixel location. Click on the image to the see the average direction and magnitude of the re-projection error for each pixel. Note that the vectors are scaled for better visualization. The scale bar indicates the magnitude of 1 pixel error.

2D Keypoints Table

	Number of 2D Keypoints per Image	Number of Matched 2D Keypoints per Image
Median	45097	12009
Min	20000	1508
Max	68781	32484
Mean	42501	12167

3D Points from 2D Keypoint Matches

	Number of 3D Points Observed
In 2 Images	2243065
In 3 Images	695820
In 4 Images	300861
In 5 Images	152862
In 6 Images	86988
In 7 Images	51576
In 8 Images	30756
In 9 Images	19678
In 10 Images	13105
In 11 Images	8846
In 12 Images	6367
In 13 Images	4564
In 14 Images	3218

In 15 Images	2440
In 16 Images	1713
In 17 Images	1321
In 18 Images	1018
In 19 Images	783
In 20 Images	627
In 21 Images	506
In 22 Images	402
In 23 Images	316
In 24 Images	231
In 25 Images	184
In 26 Images	123
In 27 Images	82
In 28 Images	71
In 29 Images	53
In 30 Images	29
In 31 Images	26
In 32 Images	20
In 33 Images	19
In 34 Images	14
In 35 Images	10
In 36 Images	6
In 38 Images	2
In 39 Images	1
In 40 Images	4
In 43 Images	1

2D Keypoint Matches

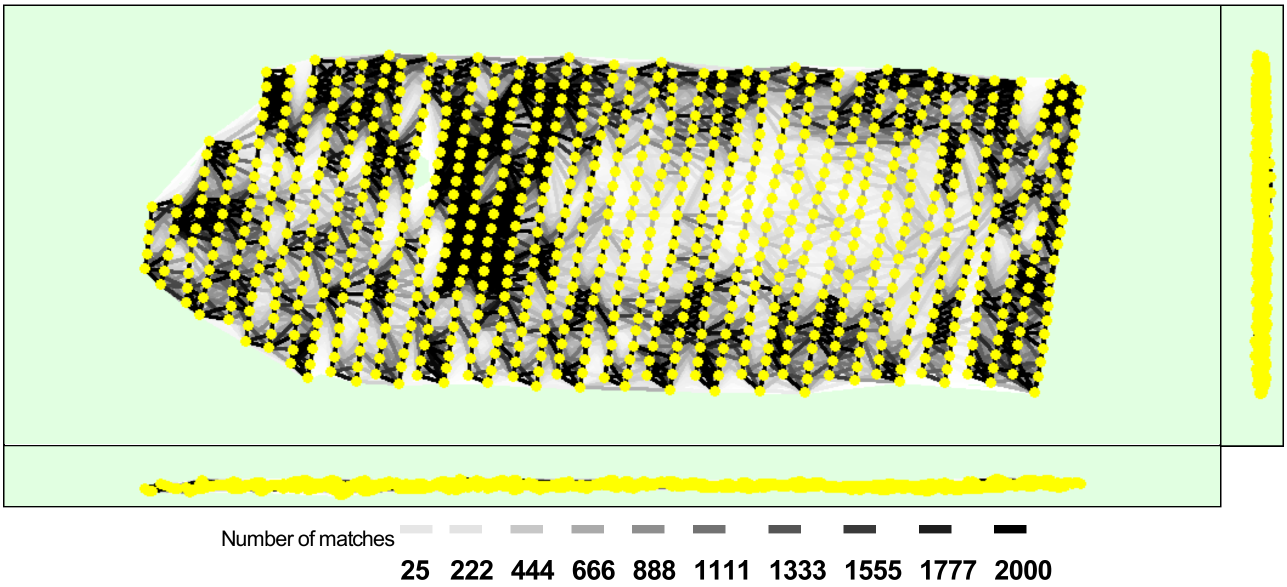

Figure 5: Computed image positions with links between matched images. The darkness of the links indicates the number of matched 2D keypoints between the images. Bright links indicate weak links and require manual tie points or more images.

Geolocation Details

Ground Control Points

GCP Name	Accuracy XY/Z [m]	Error X [m]	Error Y [m]	Error Z [m]	Projection Error [pixel]	Verified/Marked
201 (3D)	0.030/ 0.050	-0.004	-0.021	0.001	0.565	34 / 34

202 (3D)	0.030/ 0.050	-0.001	0.020	0.006	0.669	31 / 31
203 (3D)	0.030/ 0.050	-0.006	0.018	-0.008	0.735	39 / 39
208 (3D)	0.030/ 0.050	-0.002	-0.018	-0.006	0.606	38 / 38
209 (3D)	0.030/ 0.050	0.010	-0.018	-0.040	0.431	37 / 37
207 (3D)	0.030/ 0.050	-0.018	-0.019	0.060	0.459	35 / 35
211 (3D)	0.030/ 0.050	0.017	0.033	-0.016	0.455	44 / 44
210 (3D)	0.030/ 0.050	-0.004	0.000	0.038	0.275	43 / 43
206 (3D)	0.030/ 0.050	0.007	0.026	0.008	0.409	35 / 35
205 (3D)	0.030/ 0.050	-0.010	-0.035	0.003	0.839	40 / 40
204 (3D)	0.030/ 0.050	0.009	0.013	-0.046	0.805	38 / 39
**Mean [m]**		-0.000031	-0.000040	0.000147		
**Sigma [m]**		0.009535	0.022042	0.029032		
**RMS Error [m]**		0.009535	0.022042	0.029032		

0 out of 207 check points have been labeled as inaccurate.

Check Point Name	Accuracy XY/Z [m]	Error X [m]	Error Y [m]	Error Z [m]	Projection Error [pixel]	Verified/Marked
103		0.0149	0.0136	-0.0101	0.3929	42 / 42
101		-0.0030	0.0179	0.0071	0.3598	38 / 38
100		-0.0127	-0.0259	0.0374	0.3279	36 / 36
104		-0.0411	-0.0117	0.0472	0.4497	39 / 39
102		-0.0132	-0.0187	0.0041	0.3494	43 / 43
k1		0.0365	-0.0523	0.1275	0.4936	38 / 38
k2		0.0061	0.0236	0.0050	0.4328	43 / 43
k3		0.0925	-0.0764	0.1740	0.5994	26 / 26
k4		0.0266	-0.0246	0.0751	0.4763	36 / 36
k5		-0.0199	0.0310	-0.0216	0.4165	38 / 38
k6		-0.0254	0.0084	0.0505	0.7257	29 / 29
k7		-0.0758	0.0013	0.1421	0.5399	28 / 28
k8		-0.0369	-0.0342	0.1105	0.5553	30 / 30
k11		-0.0113	-0.0242	-0.0329	0.4926	43 / 43
k13		-0.0064	0.0143	0.0103	0.3807	29 / 29
k14		-0.0026	0.0137	0.0031	0.4345	38 / 38
10000		-0.0272	-0.0015	0.0355	0.5634	32 / 32
10001		-0.0023	0.0049	0.0592	0.5334	35 / 35
10002		0.0059	-0.0269	-0.0063	0.3964	37 / 37
10003		0.0017	-0.0393	-0.0245	0.4818	40 / 40
10004		0.0184	-0.0130	0.0117	0.4643	39 / 39
10005		0.0248	-0.0196	0.0071	0.5202	43 / 43
10006		0.0002	-0.0213	-0.0051	0.3643	42 / 42
10007		0.0071	-0.0196	-0.0241	0.3815	41 / 41
10008		0.0162	-0.0299	-0.0292	0.3454	38 / 38
10009		0.0087	-0.0276	-0.0148	0.4446	42 / 42
10010		0.0075	-0.0286	-0.0254	0.4481	44 / 44
10011		0.0096	-0.0392	-0.0261	0.5097	33 / 33
10012		-0.0330	0.0066	-0.0019	0.5479	40 / 40
10013		-0.0173	-0.0008	-0.0057	0.4192	38 / 38
10014		0.0074	0.0096	0.0054	0.3836	42 / 42
10015		0.0165	0.0014	0.0282	0.4230	40 / 40
10016		-0.0226	-0.0035	0.0289	0.5020	43 / 43
10017		0.0033	-0.0166	-0.0239	0.4000	42 / 42
10018		0.0023	-0.0074	-0.0001	0.3847	22 / 22
10019		-0.0096	0.0123	0.0586	0.5966	42 / 42
10020		-0.0234	0.0061	0.0272	0.4410	33 / 33
10021		0.0014	-0.0028	0.0452	0.3997	45 / 45
10022		0.0218	0.0271	0.0174	0.5226	42 / 42
10023		0.0263	-0.0087	0.0036	0.4152	38 / 38
10024		-0.0057	-0.0032	-0.0151	0.3879	46 / 46
10025		0.0106	-0.0029	0.0111	0.3801	44 / 44
10026		-0.0053	0.0205	0.0041	0.4518	36 / 36
10027		-0.0098	0.0157	-0.0019	0.4973	41 / 41

10028		0.0023	0.0016	0.0104	0.4134	43 / 43
10030		-0.0051	0.0290	-0.0185	0.3735	37 / 37
10031		0.0360	0.0246	-0.0016	0.5390	27 / 27
10032		0.0427	0.0340	0.0170	0.4427	27 / 27
10033		0.0069	0.0295	0.0119	0.3759	40 / 40
10034		0.0299	-0.0106	-0.0036	0.3924	38 / 38
10035		0.0165	-0.0285	0.0505	0.3840	35 / 35
10036		0.0184	-0.0078	-0.0004	0.5516	27 / 27
10037		0.0012	-0.0102	-0.0130	0.3627	39 / 39
10038		-0.0043	0.0092	-0.0133	0.3577	42 / 42
10039		0.0033	-0.0026	-0.0161	0.3830	43 / 43
10040		-0.0278	-0.0071	0.0079	0.4697	31 / 31
10041		-0.0167	-0.0208	-0.0066	0.4448	35 / 35
10044		0.0119	-0.0427	-0.0353	0.4194	38 / 38
10045		0.0175	-0.0385	-0.0631	0.3539	36 / 36
10046		-0.0112	-0.0123	-0.0792	0.3704	38 / 38
10047		-0.0270	-0.0083	0.0629	0.7051	31 / 31
10048		-0.0127	-0.0251	0.0330	0.6080	32 / 32
10049		-0.0196	0.0250	0.0303	0.8386	32 / 32
10051		0.0029	0.0161	0.0149	0.3672	44 / 44
10052		-0.0009	0.0008	0.0171	0.5097	35 / 35
10053		-0.0115	0.0119	0.0043	0.4015	40 / 40
10054		-0.0099	-0.0185	-0.0100	0.4182	38 / 38
10055		0.0034	0.0092	-0.0093	0.5350	39 / 39
10056		-0.0365	0.0077	0.0116	0.4994	38 / 38
10058		-0.0698	0.0825	0.0446	0.5057	44 / 44
10059		-0.0098	-0.0251	-0.0581	0.3554	40 / 40
10060		-0.0271	-0.0258	-0.0271	0.3478	43 / 43
10061		0.0077	0.0235	0.0405	0.4797	29 / 29
10062		-0.0020	-0.0266	-0.0032	0.4510	41 / 41
10063		0.0171	-0.0477	-0.0288	0.4670	38 / 38
10064		0.0375	0.0172	-0.0403	0.4300	43 / 43
10065		0.0285	0.0251	-0.0225	0.3601	44 / 44
10066		0.0568	0.0289	-0.0330	0.4254	41 / 41
10067		0.0312	0.0303	-0.0227	0.3498	42 / 42
10068		0.0130	0.1010	-0.0311	0.4354	43 / 43
10074		-0.0202	0.0462	-0.0436	0.6679	41 / 41
10075		-0.0154	0.0441	0.0001	0.3089	41 / 41
10076		0.0045	0.0213	-0.0130	0.3467	40 / 40
10077		-0.0191	0.0559	-0.0253	0.7157	38 / 38
10078		0.0236	0.0145	-0.0230	0.3283	39 / 39
10080		0.0169	-0.0631	-0.0965	0.6537	35 / 35
10081		-0.0214	-0.0264	-0.0035	0.3412	37 / 37
10082		0.0121	-0.0391	-0.0355	0.3988	39 / 39
10083		0.0537	-0.0189	-0.0630	0.5878	37 / 37
10085		0.0332	-0.0351	0.0006	0.3340	44 / 44
15000		0.0064	-0.0391	-0.0446	0.3326	46 / 46
15001		-0.0258	-0.0431	-0.0547	0.3392	39 / 39
15002		0.0103	0.0192	-0.0858	0.4433	41 / 41
15003		-0.0027	0.0382	-0.0219	0.4479	45 / 45
15004		-0.0059	0.0182	-0.0310	0.3753	43 / 43
15005		-0.0043	0.0262	-0.0264	0.4930	38 / 38
15006		0.0098	0.0387	-0.0352	0.4099	43 / 43
15007		0.0113	0.0028	0.0093	0.4333	38 / 38
15008		-0.0224	0.0243	0.0456	0.7326	38 / 38
15009		-0.0123	0.0158	0.0386	0.4835	40 / 40
15010		0.0023	0.0412	0.0282	0.5830	38 / 38
20000		-0.0127	0.0251	0.0098	0.6963	42 / 42
20001		-0.0117	0.0138	0.0303	0.4810	38 / 38

20002		-0.0014	0.0275	0.0297	0.3947	38 / 38
20003		-0.0260	-0.0031	0.0238	0.3993	39 / 39
20004		-0.0060	-0.0074	0.0198	0.3757	36 / 36
20005		-0.0469	-0.0281	-0.0140	0.5012	35 / 35
20008		-0.0025	-0.0014	-0.0035	0.3847	44 / 44
20009		0.0124	-0.0085	-0.0169	0.3873	43 / 43
20010		-0.0219	0.0211	-0.0059	0.3969	45 / 45
20011		-0.0153	-0.0039	0.0106	0.3619	47 / 47
20012		0.0103	-0.0034	-0.0029	0.3383	44 / 44
20013		0.0125	-0.0037	0.0106	0.3498	41 / 41
20014		0.0019	0.0049	-0.0032	0.3523	43 / 43
20016		0.0033	0.0126	-0.0325	0.3257	46 / 46
20017		0.0148	0.0217	-0.0189	0.3617	40 / 40
20018		0.0248	0.0193	-0.0089	0.3175	44 / 44
20019		0.0301	0.0110	0.0033	0.4086	39 / 39
20020		0.0109	0.0346	-0.0093	0.4336	40 / 40
20021		0.0093	-0.0008	0.0055	0.2695	40 / 40
20022		-0.0141	-0.0523	0.0043	0.3153	42 / 42
20023		0.0030	0.0026	-0.0113	0.3518	45 / 45
20024		-0.0003	0.0390	-0.0371	0.4253	41 / 41
20025		0.0155	0.0468	-0.0286	0.3494	42 / 42
20026		0.0148	0.0376	-0.0264	0.3936	43 / 43
20027		0.0082	0.0727	-0.0282	0.3701	40 / 40
20028		-0.0056	0.0136	-0.0084	0.3943	41 / 41
20029		0.0134	-0.0133	-0.0210	0.7493	41 / 41
20031		-0.0089	0.0199	0.0038	0.3981	45 / 45
25000		0.0142	0.0136	-0.0246	0.2608	40 / 40
25001		0.0051	0.0119	0.0154	0.2847	40 / 40
25003		-0.0003	-0.0161	0.0199	0.3602	40 / 40
25004		0.0006	0.0036	-0.0052	0.3307	33 / 33
25005		0.0043	-0.0082	0.0075	0.2750	39 / 39
25006		-0.0324	-0.0422	-0.0464	0.4458	38 / 38
25008		-0.0296	-0.0008	-0.0436	0.2230	38 / 38
25009		0.0295	-0.0509	-0.0521	0.2356	42 / 42
25010		0.0066	-0.0188	-0.0568	0.2540	41 / 41
25013		-0.0005	0.0144	-0.0382	0.3945	38 / 38
25014		-0.0084	-0.0092	-0.0513	0.2957	37 / 37
25015		-0.0222	-0.0109	-0.0451	0.4212	41 / 41
25016		-0.0229	-0.0098	-0.0415	0.2776	42 / 42
25017		0.0257	-0.0183	0.0300	0.4299	37 / 37
25018		0.0093	-0.0011	0.0180	0.3937	38 / 38
25019		-0.0117	-0.0112	0.0084	0.4665	39 / 39
25020		-0.0072	0.0052	-0.0228	0.3780	33 / 33
25021		0.0204	0.0234	-0.0073	0.3555	36 / 36
25022		0.0178	0.0044	0.0071	0.4884	34 / 34
25023		-0.0016	0.0222	-0.0085	0.4294	40 / 40
25024		0.0046	-0.0150	-0.0110	0.3445	40 / 40
25029		0.0105	-0.0076	0.0081	0.3135	42 / 42
25030		-0.0069	-0.0111	0.0092	0.4069	44 / 44
25032		-0.0712	-0.0264	-0.0338	0.4164	36 / 36
25033		-0.0199	-0.0205	-0.0067	0.3737	41 / 41
25034		-0.0140	-0.0206	-0.0138	0.3994	36 / 36
40000		0.0065	-0.0150	-0.1047	0.3069	42 / 42
40001		0.0237	0.0191	-0.0252	0.2848	43 / 43
40002		0.0211	0.0392	-0.0391	0.3902	42 / 42
40003		0.0127	0.0431	-0.0120	0.4032	39 / 39
40004		0.0073	0.0043	-0.0208	0.3020	37 / 37
40005		0.0211	0.0231	-0.0390	0.2758	29 / 29
45000		0.0362	-0.0293	0.0007	0.3975	44 / 44

45001		-0.0023	0.0031	-0.0066	0.3895	34 / 34
45002		0.0146	-0.0336	0.0007	0.4830	41 / 41
10043		-0.0161	-0.0055	0.0336	0.6103	35 / 35
10084		-0.0112	-0.0030	0.0565	0.5341	30 / 30
20015		0.0023	-0.0131	-0.0356	0.3167	45 / 45
20007		0.0011	-0.0112	-0.0212	0.3856	24 / 24
25027		-0.0003	-0.0119	-0.0352	0.2948	43 / 43
k9		-0.0100	-0.0158	-0.0166	0.5422	42 / 42
6600		0.0027	-0.0200	-0.0451	0.3958	41 / 41
6602		0.0041	0.0265	-0.0336	0.3795	37 / 37
6603		-0.0014	-0.0216	-0.0081	0.4259	40 / 40
6605		0.0132	0.0145	0.0028	0.4739	40 / 40
6606		0.0186	0.0265	-0.0038	0.5617	25 / 25
6607		0.0040	0.0086	-0.0402	0.7010	35 / 35
6608		-0.0041	-0.0190	0.0047	0.6547	21 / 21
6609		-0.0141	0.0013	0.0045	0.3791	42 / 42
6610		0.0034	0.0001	0.0197	0.7478	33 / 33
6611		-0.0086	-0.0270	-0.0434	0.5416	40 / 40
6612		-0.0010	-0.0581	-0.0537	0.7160	38 / 38
6616		-0.0067	-0.0365	-0.0517	0.4725	33 / 33
6617		-0.0052	-0.0059	0.0130	0.6049	23 / 23
6618		-0.0047	-0.0036	-0.0204	0.5178	40 / 40
6619		0.0219	-0.0001	0.0085	0.2795	41 / 41
6620		0.0197	-0.0462	-0.0103	0.4141	38 / 38
6621		-0.0214	-0.0105	0.0097	0.2191	41 / 41
6622		0.0016	0.0080	0.0301	0.5521	37 / 37
6623		-0.0017	-0.0193	0.0260	0.6539	40 / 40
6624		0.0536	-0.0070	0.0262	0.6566	33 / 33
6625		-0.0430	-0.0243	-0.0175	0.5288	37 / 37
6626		-0.0120	-0.0057	-0.0535	0.4134	35 / 35
6627		-0.0235	-0.0196	-0.0190	0.5825	33 / 33
6628		-0.0302	-0.0285	-0.0388	0.4592	39 / 39
6629		-0.0446	-0.0241	-0.0370	0.4384	37 / 37
6630		-0.0543	-0.0085	-0.0275	0.3571	34 / 34
6631		-0.0297	-0.0246	-0.0337	0.5239	32 / 32
6632		-0.0484	-0.0150	-0.0172	0.5519	34 / 34
6633		-0.0112	-0.0162	-0.0241	0.4393	38 / 38
6634		-0.0160	0.0033	-0.0197	0.3937	35 / 35
6635		-0.0017	0.0046	-0.0173	0.4559	39 / 39
6636		-0.0432	-0.0306	-0.0258	0.4894	41 / 41
6637		-0.0046	-0.0095	-0.0064	0.5909	31 / 31
6638		-0.0236	-0.0098	-0.0103	0.4258	27 / 27
6639		-0.0498	-0.0038	-0.0174	0.5155	38 / 38
6640		-0.0373	-0.0219	-0.0213	0.4762	34 / 34
6641		-0.0480	-0.0148	-0.0192	0.5427	37 / 37
**Mean [m]**		-0.001640	-0.001975	-0.005499		
**Sigma [m]**		0.022614	0.025768	0.035491		
**RMS Error [m]**		0.022674	0.025844	0.035915		

Localisation accuracy per GCP and mean errors in the three coordinate directions. The last column counts the number of calibrated images where the GCP has been automatically verified vs. manually marked.

Initial Processing Details

System Information

Hardware	CPU: Intel(R) Core(TM) i9-7940X CPU @ 3.10GHz<br>RAM: 128GB<br>GPU: NVIDIA GeForce GTX 1080 Ti (Driver: 26.21.14.3615), RDPDD Chained DD (Driver: unknown), RDP Encoder Mirror Driver (Driver: unknown), RDP Reflector Display Driver (Driver: unknown)
Operating System	Windows 7 Ultimate, 64-bit

Coordinate Systems

Ground Control Point (GCP) Coordinate System	ETRS89 / Poland CS2000 zone 6 (EGM 2008 Geoid)
Output Coordinate System	ETRS89 / Poland CS2000 zone 6 (EGM 2008 Geoid)

Processing Options

Detected Template	No Template Available
Keypoints Image Scale	Full, Image Scale: 1
Advanced: Matching Image Pairs	Aerial Grid or Corridor
Advanced: Matching Strategy	Use Geometrically Verified Matching: no
Advanced: Keypoint Extraction	Targeted Number of Keypoints: Automatic
Advanced: Calibration	Calibration Method: Standard<br>Internal Parameters Optimization: All<br>External Parameters Optimization: All<br>Rematch: Auto, no
