# Supplementary material for: Multi-Variant Accuracy Evaluation of UAV Imaging Surveys: A Case Study on Investment Area
Source: Sensors (Basel). 2019 Nov 28;19(23):5229. doi: 10.3390/s19235229 (PMC6929115; doi:10.3390/s19235229)
Supplement: Supplementary file 1 [file sensors-19-05229-s001.zip › supplementary_files/App1_V1_report.pdf]

Quality Report

Generated with Pix4Ddiscovery version 4.3.31

Important: Click on the different icons for:

Help to analyze the results in the Quality Report

Additional information about the sections

Click here for additional tips to analyze the Quality Report

Summary

Project	Sosnowiec 1_nowa wersja
Processed	2019-02-08 04:05:19
Camera Model Name(s)	NEX-5T_0.0_4912x3264 (RGB)
Average Ground Sampling Distance (GSD)	4.58 cm / 1.80 in
Area Covered	1.035 km^2 / 103.5423 ha / 0.40 sq. mi. / 255.9911 acres
Time for Initial Processing (without report)	01h:16m:22s

Quality Check

**Images**	median of 45103 keypoints per image	✓
**Dataset**	858 out of 858 images calibrated (100%), all images enabled	✓
**Camera Optimization**	0.23% relative difference between initial and optimized internal camera parameters	✓
**Matching**	median of 19262.1 matches per calibrated image	✓
**Georeferencing**	yes, 8 GCPs (8 3D), mean RMS error = 0.016 m	✓

Preview

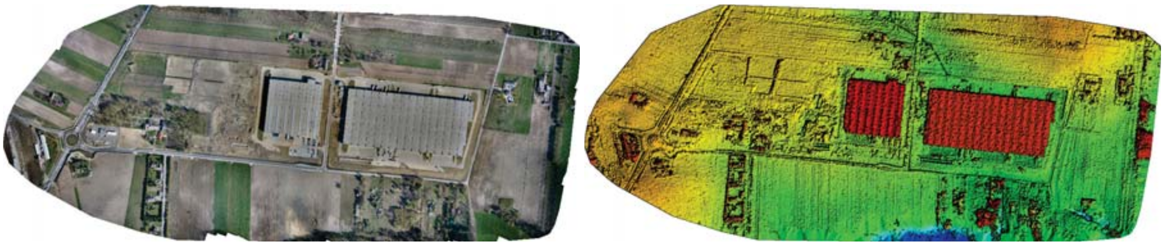

Figure 1: Orthomosaic and the corresponding sparse Digital Surface Model (DSM) before densification.

Calibration Details

Number of Calibrated Images	858 out of 858
Number of Geolocated Images	858 out of 858

Initial Image Positions

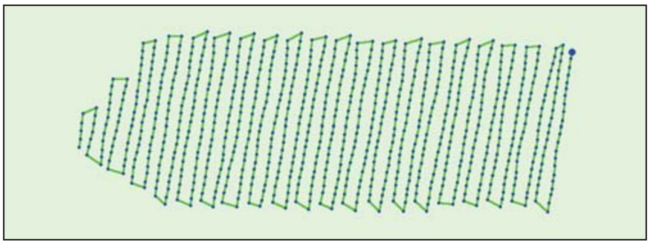

Figure 2: Top view of the initial image position. The green line follows the position of the images in time starting from the large blue dot.

Computed Image/GCPs/Manual Tie Points Positions

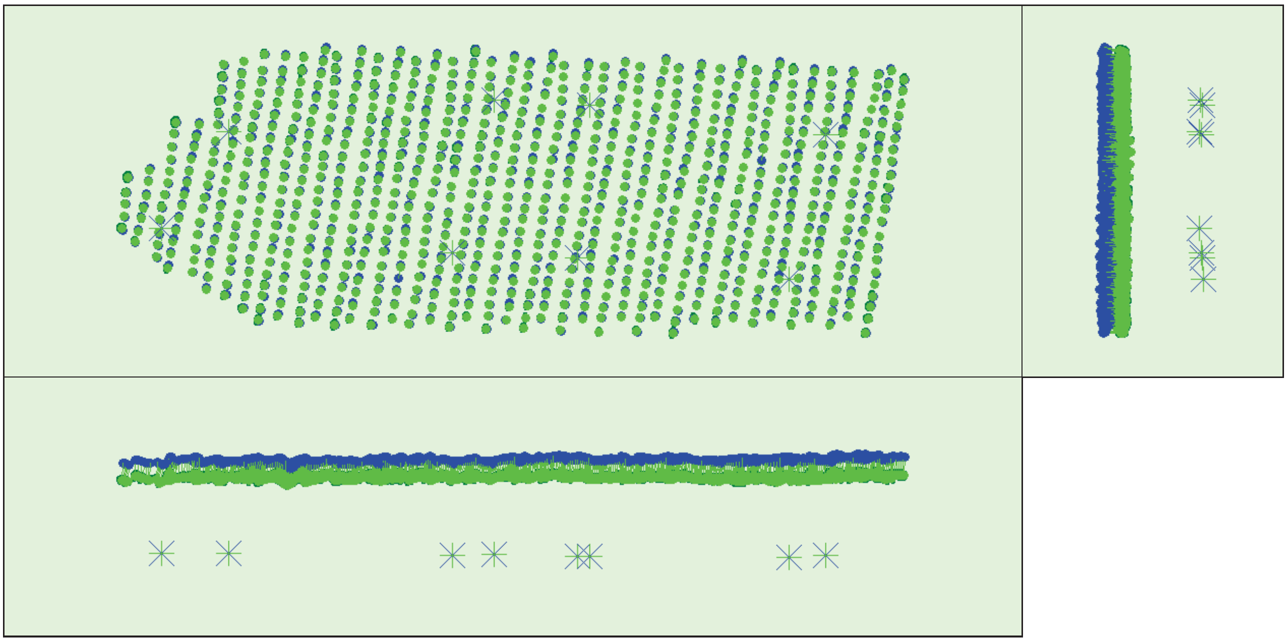

Uncertainty ellipses 100x magnified

Figure 3: Offset between initial (blue dots) and computed (green dots) image positions as well as the offset between the GCPs initial positions (blue crosses) and their computed positions (green crosses) in the top-view (XY plane), front-view (XZ plane), and side-view (YZ plane). Dark green ellipses indicate the absolute position uncertainty of the bundle block adjustment result.

Absolute camera position and orientation uncertainties

	X[m]	Y[m]	Z[m]	Omega [degree]	Phi [degree]	Kappa [degree]
Mean	0.048	0.060	0.081	0.021	0.016	0.006
Sigma	0.010	0.011	0.003	0.004	0.004	0.002

Overlap

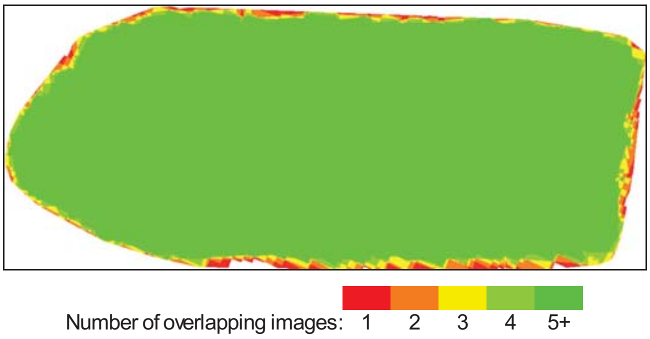

Figure 4: Number of overlapping images computed for each pixel of the orthomosaic.
Red and yellow areas indicate low overlap for which poor results may be generated. Green areas indicate an overlap of over 5 images for every pixel. Good quality results will be generated as long as the number of keypoint matches is also sufficient for these areas (see Figure 5 for keypoint matches).

Bundle Block Adjustment Details

Number of 2D Keypoint Observations for Bundle Block Adjustment	16090747
Number of 3D Points for Bundle Block Adjustment	3119086
Mean Reprojection Error [pixels]	0.246

Internal Camera Parameters

NEX-5T_0.0_4912x3264 (RGB). Sensor Dimensions: 23.400 [mm] x 15.549 [mm]

EXIF ID: NEX-5T_0.0_4912x3264

	Focal Length	Principal Point x	Principal Point y	R1	R2	R3	T1	T2
Initial Values	3256.554 [pixel] 15.514 [mm]	2456.002 [pixel] 11.700 [mm]	1632.003 [pixel] 7.775 [mm]	-0.043	0.028	-0.005	0.000	-0.001
Optimized Values	3248.877 [pixel] 15.477 [mm]	2466.764 [pixel] 11.751 [mm]	1589.886 [pixel] 7.574 [mm]	-0.047	0.035	-0.009	0.000	0.000
Uncertainties (Sigma)	1.659 [pixel] 0.008 [mm]	0.234 [pixel] 0.001 [mm]	0.248 [pixel] 0.001 [mm]	0.000	0.000	0.000	0.000	0.000

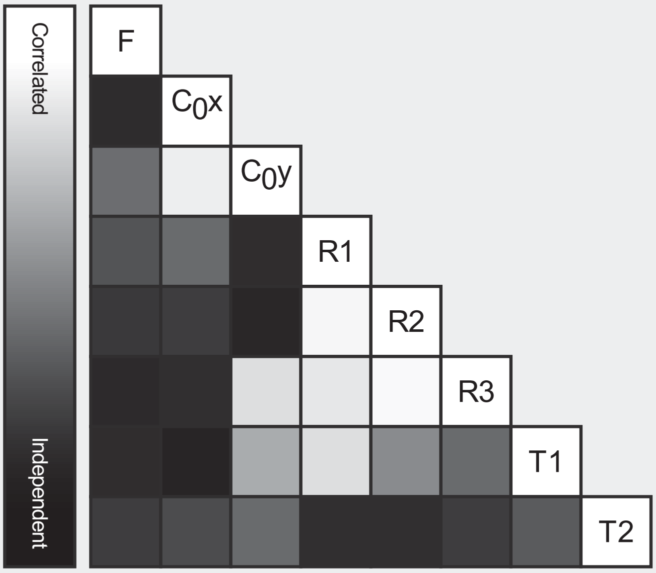

The correlation between camera internal parameters determined by the bundle adjustment. White indicates a full correlation between the parameters, ie. any change in one can be fully compensated by the other. Black indicates that the parameter is completely independent, and is not affected by other parameters.

The number of Automatic Tie Points (ATPs) per pixel, averaged over all images of the camera model, is color coded between black and white. White indicates that, on average, more than 16 ATPs have been extracted at the pixel location. Black indicates that, on average, 0 ATPs have been extracted at the pixel location. Click on the image to the see the average direction and magnitude of the re-projection error for each pixel. Note that the vectors are scaled for better visualization. The scale bar indicates the magnitude of 1 pixel error.

2D Keypoints Table

	Number of 2D Keypoints per Image	Number of Matched 2D Keypoints per Image
Median	45103	19262
Min	20002	5719
Max	68781	33649
Mean	42505	18754

3D Points from 2D Keypoint Matches

	Number of 3D Points Observed
In 2 Images	1320934

In 3 Images	533808
In 4 Images	296763
In 5 Images	191546
In 6 Images	133477
In 7 Images	99591
In 8 Images	77493
In 9 Images	61954
In 10 Images	51234
In 11 Images	41956
In 12 Images	36047
In 13 Images	30898
In 14 Images	27212
In 15 Images	23581
In 16 Images	21143
In 17 Images	18847
In 18 Images	16443
In 19 Images	14782
In 20 Images	13348
In 21 Images	11903
In 22 Images	10860
In 23 Images	9833
In 24 Images	8796
In 25 Images	8160
In 26 Images	7055
In 27 Images	6324
In 28 Images	5677
In 29 Images	5069
In 30 Images	4721
In 31 Images	4276
In 32 Images	3797
In 33 Images	3436
In 34 Images	3037
In 35 Images	2739
In 36 Images	2358
In 37 Images	2013
In 38 Images	1766
In 39 Images	1460
In 40 Images	1243
In 41 Images	990
In 42 Images	827
In 43 Images	634
In 44 Images	423
In 45 Images	285
In 46 Images	183
In 47 Images	88
In 48 Images	44
In 49 Images	21
In 50 Images	10
In 51 Images	1

2D Keypoint Matches

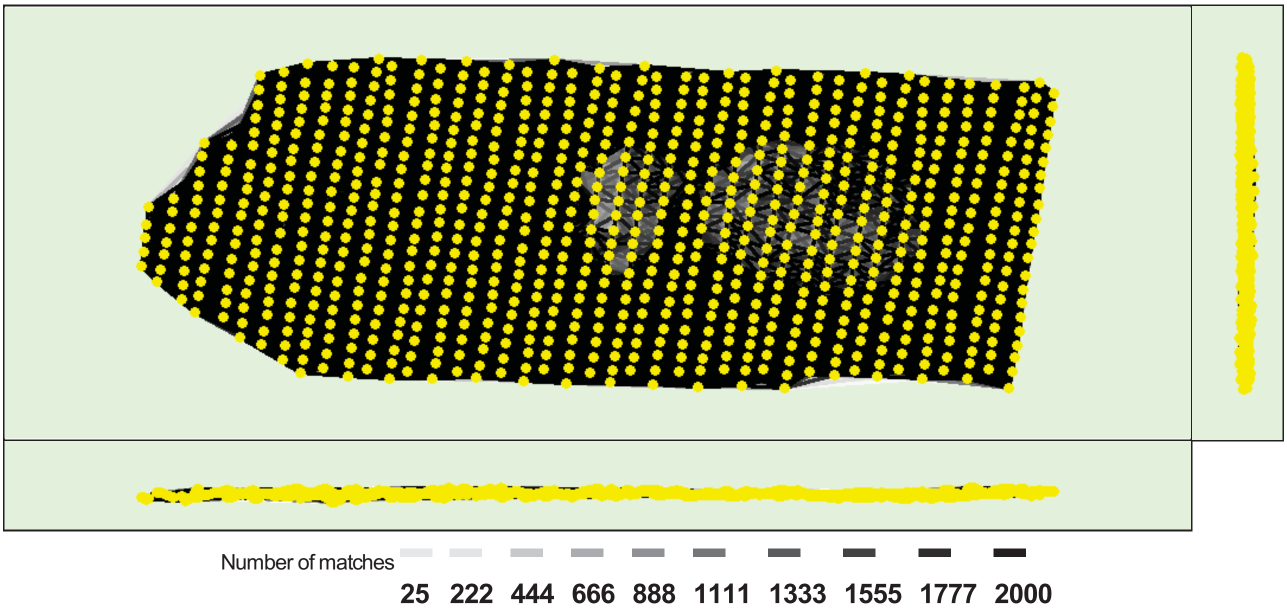

Figure 5: Computed image positions with links between matched images. The darkness of the links indicates the number of matched 2D keypoints between the images. Bright links indicate weak links and require manual tie points or more images.

Geolocation Details

Ground Control Points

GCP Name	Accuracy XY/Z [m]	Error X [m]	Error Y [m]	Error Z [m]	Projection Error [pixel]	Verified/Marked
103 (3D)	0.030/ 0.050	0.012	0.008	0.036	0.459	42 / 42
101 (3D)	0.030/ 0.050	0.012	0.010	0.003	0.436	38 / 38
100 (3D)	0.030/ 0.050	0.008	-0.022	-0.022	0.384	36 / 36
102 (3D)	0.030/ 0.050	-0.020	-0.018	0.022	0.358	43 / 43
201 (3D)	0.030/ 0.050	-0.006	-0.022	-0.001	0.820	34 / 34
202 (3D)	0.030/ 0.050	0.006	0.014	0.004	0.753	31 / 31
203 (3D)	0.030/ 0.050	-0.007	0.020	-0.002	0.779	39 / 39
204 (3D)	0.030/ 0.050	-0.004	0.009	-0.040	0.916	38 / 39
**Mean [m]**		0.000068	-0.000013	-0.000116		
**Sigma [m]**		0.010697	0.016228	0.021925		
**RMS Error [m]**		0.010697	0.016228	0.021926		

0 out of 210 check points have been labeled as inaccurate.

Check Point Name	Accuracy XY/Z [m]	Error X [m]	Error Y [m]	Error Z [m]	Projection Error [pixel]	Verified/Marked
104		-0.0491	-0.0113	0.0134	0.5338	39 / 39
k1		0.0308	-0.0544	0.1746	0.5073	38 / 38
k2		0.0004	0.0229	0.0523	0.4576	43 / 43
k3		0.0876	-0.0760	0.2243	0.6128	26 / 26
k4		0.0214	-0.0244	0.1237	0.4961	36 / 36
k5		-0.0248	0.0311	0.0280	0.4309	38 / 38
k6		-0.0285	0.0088	0.1029	0.7222	29 / 29
k7		-0.0782	0.0015	0.1946	0.5389	28 / 28
k8		-0.0412	-0.0337	0.1627	0.5960	30 / 30
k11		-0.0145	-0.0217	-0.0193	0.5367	43 / 43
k13		0.0131	0.0112	-0.0241	0.4319	29 / 29
k14		0.0172	0.0096	-0.0155	0.4956	38 / 38
10000		-0.0088	-0.0079	0.0284	0.6447	30 / 30
10001		0.0156	0.0043	0.0239	0.5974	35 / 35
10002		0.0182	-0.0245	-0.0406	0.4955	37 / 37
10003		0.0136	-0.0352	-0.0542	0.5460	40 / 40

10004		0.0260	-0.0121	0.0113	0.5243	39 / 39
10005		0.0317	-0.0191	0.0063	0.5870	43 / 43
10006		0.0056	-0.0212	0.0011	0.4264	42 / 42
10007		0.0052	-0.0183	-0.0105	0.4684	41 / 41
10008		0.0148	-0.0292	-0.0172	0.4256	38 / 38
10009		0.0075	-0.0272	0.0010	0.5282	42 / 42
10010		0.0062	-0.0278	-0.0102	0.5019	44 / 44
10011		0.0076	-0.0391	-0.0092	0.5661	33 / 33
10012		-0.0395	0.0091	0.0186	0.5771	38 / 38
10013		-0.0237	0.0006	0.0207	0.4310	38 / 38
10014		0.0006	0.0108	0.0344	0.3782	42 / 42
10015		0.0111	0.0026	0.0582	0.4333	40 / 40
10016		-0.0273	-0.0020	0.0493	0.5231	43 / 43
10017		0.0002	-0.0146	-0.0063	0.4372	42 / 42
10018		-0.0005	-0.0049	0.0123	0.3891	22 / 22
10019		-0.0178	0.0129	0.0800	0.6341	42 / 42
10020		-0.0312	0.0065	0.0521	0.4738	33 / 33
10021		-0.0056	-0.0055	0.0780	0.4336	45 / 45
10022		0.0144	0.0231	0.0532	0.5254	42 / 42
10023		0.0196	-0.0126	0.0422	0.4205	38 / 38
10024		-0.0135	-0.0070	0.0158	0.3963	46 / 46
10025		0.0028	-0.0071	0.0423	0.3900	44 / 44
10026		-0.0117	0.0159	0.0426	0.4601	36 / 36
10027		-0.0156	0.0112	0.0397	0.5078	41 / 41
10028		-0.0032	-0.0031	0.0516	0.4254	43 / 43
208		-0.0051	-0.0230	0.0396	0.6310	38 / 38
10030		-0.0078	0.0242	0.0285	0.3998	37 / 37
10031		0.0335	0.0203	0.0468	0.5323	27 / 27
10032		0.0434	0.0297	0.0656	0.4657	27 / 27
10033		0.0123	0.0242	0.0568	0.3854	40 / 40
10034		0.0360	-0.0141	0.0431	0.4012	38 / 38
10035		0.0223	-0.0312	0.0965	0.4042	35 / 35
10036		0.0255	-0.0111	0.0434	0.5656	27 / 27
10037		0.0133	-0.0170	0.0181	0.3970	39 / 39
10038		0.0116	0.0022	0.0102	0.3801	42 / 42
10039		0.0242	-0.0103	-0.0101	0.4426	43 / 43
10040		-0.0060	-0.0045	-0.0515	0.4989	28 / 28
10041		0.0012	-0.0174	-0.0547	0.5895	35 / 35
209		0.0313	-0.0136	-0.1007	0.4850	37 / 37
10044		0.0204	-0.0401	-0.0554	0.5606	38 / 38
10045		0.0285	-0.0355	-0.0774	0.4152	36 / 36
10046		-0.0178	-0.0107	-0.0586	0.3846	38 / 38
10047		-0.0365	-0.0051	0.1183	0.8202	31 / 31
10048		-0.0177	-0.0237	0.0774	0.6408	32 / 32
10049		-0.0254	0.0320	0.0815	1.0129	31 / 31
207		-0.0199	-0.0209	0.1124	0.4687	35 / 35
10051		-0.0037	0.0129	0.0562	0.4044	44 / 44
10052		-0.0056	0.0015	0.0639	0.5350	35 / 35
10053		-0.0168	0.0126	0.0357	0.4152	40 / 40
10054		-0.0157	-0.0174	0.0191	0.4315	38 / 38
10055		-0.0068	0.0060	0.0199	0.5799	39 / 39
10056		-0.0463	0.0065	0.0332	0.5292	38 / 38
10058		-0.0798	0.0827	0.0640	0.5498	43 / 43
10059		-0.0097	-0.0225	-0.0465	0.3855	40 / 40
10060		-0.0337	-0.0250	-0.0053	0.3626	43 / 43
10061		0.0031	0.0249	0.0673	0.4984	29 / 29
10062		-0.0076	-0.0251	0.0148	0.4873	41 / 41
10063		0.0127	-0.0462	-0.0114	0.4858	38 / 38
10064		0.0360	0.0120	0.0060	0.4596	43 / 43

10065		0.0302	0.0201	0.0241	0.3715	44 / 44
10066		0.0584	0.0214	0.0109	0.4471	41 / 41
10067		0.0331	0.0232	0.0215	0.3763	42 / 42
10068		0.0154	0.0937	0.0126	0.4569	43 / 43
10074		-0.0169	0.0388	-0.0004	0.6970	41 / 41
10075		-0.0103	0.0380	0.0438	0.3292	41 / 41
10076		0.0083	0.0137	0.0304	0.3582	40 / 40
10077		-0.0115	0.0463	0.0124	0.7409	38 / 38
10078		0.0352	0.0049	0.0049	0.3433	39 / 39
211		0.0329	0.0266	-0.0230	0.5359	44 / 44
10080		0.0153	-0.0616	-0.0839	0.7031	35 / 35
10081		-0.0236	-0.0251	0.0092	0.3653	37 / 37
10082		0.0101	-0.0382	-0.0210	0.4362	39 / 39
10083		0.0492	-0.0242	-0.0225	0.6024	37 / 37
10085		0.0271	-0.0337	0.0245	0.3449	44 / 44
15000		0.0126	-0.0382	-0.0436	0.3797	46 / 46
15001		-0.0230	-0.0408	-0.0434	0.3915	39 / 39
15002		0.0220	0.0093	-0.0592	0.4866	41 / 41
15003		0.0087	0.0273	0.0023	0.4866	45 / 45
15004		0.0113	0.0082	-0.0167	0.3841	43 / 43
15005		0.0145	0.0195	-0.0231	0.5887	38 / 38
15006		0.0257	0.0308	-0.0367	0.4703	43 / 43
15007		0.0299	-0.0039	-0.0037	0.4856	38 / 38
15008		-0.0033	0.0182	0.0309	0.8072	38 / 38
15009		0.0077	0.0131	0.0162	0.5705	40 / 40
15010		0.0219	0.0399	-0.0042	0.6733	38 / 38
20000		0.0051	0.0179	0.0041	0.7823	42 / 42
20001		0.0085	0.0077	0.0177	0.5397	38 / 38
20002		0.0188	0.0230	0.0129	0.4603	38 / 38
20003		-0.0072	-0.0040	0.0008	0.4551	38 / 38
20004		0.0122	-0.0079	-0.0170	0.4259	36 / 36
20005		-0.0517	-0.0268	0.0180	0.5385	35 / 35
20008		-0.0081	-0.0004	0.0256	0.4010	44 / 44
20009		0.0070	-0.0072	0.0060	0.3921	43 / 43
20010		-0.0294	0.0180	0.0250	0.4185	45 / 45
20011		-0.0232	-0.0072	0.0430	0.3890	47 / 47
20012		0.0028	-0.0071	0.0318	0.3520	44 / 44
20013		0.0057	-0.0072	0.0493	0.3601	40 / 41
20014		-0.0043	0.0007	0.0377	0.3517	43 / 43
20016		-0.0049	0.0082	-0.0037	0.3393	46 / 46
20017		0.0068	0.0176	0.0139	0.3735	40 / 40
20018		0.0184	0.0142	0.0275	0.3360	44 / 44
20019		0.0239	0.0056	0.0415	0.4181	39 / 39
20020		0.0086	0.0302	0.0397	0.4522	40 / 40
20021		0.0152	-0.0059	0.0505	0.2688	40 / 40
20022		-0.0077	-0.0577	0.0482	0.3180	42 / 42
20023		0.0102	-0.0033	0.0313	0.3492	45 / 45
20024		0.0053	0.0313	0.0047	0.4399	41 / 41
20025		0.0210	0.0394	0.0133	0.3549	42 / 42
20026		0.0218	0.0291	0.0143	0.4161	43 / 43
20027		0.0192	0.0640	0.0026	0.4008	40 / 40
20028		0.0109	0.0053	0.0118	0.4309	41 / 41
20029		0.0334	-0.0207	-0.0131	0.7924	41 / 41
20031		-0.0183	0.0174	0.0312	0.4513	45 / 45
25000		0.0345	0.0061	-0.0185	0.2592	40 / 40
25001		0.0235	0.0052	0.0053	0.3161	40 / 40
210		0.0154	-0.0019	0.0137	0.2992	43 / 43
25003		0.0196	-0.0133	-0.0333	0.3953	40 / 40
25004		0.0215	0.0078	-0.0633	0.3651	33 / 33

25005		0.0189	-0.0048	-0.0352	0.3207	39 / 39
25006		-0.0212	-0.0390	-0.0614	0.4998	38 / 38
25008		-0.0314	-0.0005	-0.0414	0.3583	38 / 38
25009		0.0282	-0.0503	-0.0467	0.3735	42 / 42
25010		0.0089	-0.0161	-0.0451	0.2858	41 / 41
25013		-0.0074	0.0162	-0.0143	0.4078	38 / 38
25014		-0.0158	-0.0072	-0.0272	0.3116	37 / 37
25015		-0.0293	-0.0091	-0.0215	0.4443	41 / 41
25016		-0.0300	-0.0080	-0.0179	0.2912	42 / 42
25017		0.0195	-0.0174	0.0680	0.4425	37 / 37
25018		0.0043	0.0004	0.0597	0.3993	38 / 38
25019		-0.0168	-0.0096	0.0495	0.4867	39 / 39
25020		-0.0129	0.0058	0.0177	0.3732	33 / 33
25021		0.0166	0.0240	0.0438	0.3759	36 / 36
25022		0.0142	0.0050	0.0574	0.4994	34 / 34
25023		-0.0044	0.0227	0.0472	0.4351	40 / 40
25024		0.0019	-0.0199	0.0366	0.3529	40 / 40
25029		0.0076	-0.0057	0.0230	0.3439	42 / 42
25030		-0.0130	-0.0098	0.0319	0.3946	44 / 44
25032		-0.0770	-0.0251	-0.0072	0.4291	36 / 36
25033		-0.0253	-0.0198	0.0254	0.3718	41 / 41
25034		-0.0189	-0.0195	0.0262	0.4158	36 / 36
206		0.0021	0.0270	0.0558	0.4193	35 / 35
40000		0.0231	-0.0238	-0.0848	0.3126	42 / 42
40001		0.0332	0.0100	0.0118	0.2932	43 / 43
40002		0.0236	0.0322	0.0048	0.3876	42 / 42
40003		0.0158	0.0382	0.0335	0.3930	39 / 39
40004		0.0125	-0.0000	0.0259	0.3056	37 / 37
40005		0.0225	0.0185	0.0102	0.2920	29 / 29
45000		0.0295	-0.0276	0.0265	0.4269	44 / 44
45001		-0.0085	0.0039	0.0281	0.4194	34 / 34
45002		0.0086	-0.0325	0.0253	0.5106	41 / 41
10043		-0.0021	-0.0011	-0.0076	0.6875	35 / 35
10084		-0.0012	-0.0027	0.0547	0.5652	30 / 30
20015		-0.0061	-0.0170	-0.0075	0.3374	45 / 45
20007		-0.0028	-0.0110	0.0258	0.3867	24 / 24
25027		-0.0081	-0.0152	-0.0098	0.3326	43 / 43
k9		-0.0102	-0.0162	-0.0143	0.6067	42 / 42
205		-0.0178	-0.0338	0.0165	0.9031	40 / 40
6600		0.0044	-0.0271	-0.0008	0.4270	41 / 41
6602		0.0017	0.0215	0.0137	0.3946	37 / 37
6603		-0.0035	-0.0259	0.0388	0.4450	40 / 40
6605		0.0094	0.0134	0.0531	0.4989	40 / 40
6606		0.0151	0.0271	0.0466	0.6054	23 / 23
6607		-0.0014	0.0035	0.0008	0.7332	35 / 35
6608		-0.0108	-0.0216	0.0433	0.6822	21 / 21
6609		-0.0221	-0.0016	0.0336	0.3908	42 / 42
6610		-0.0041	-0.0010	0.0516	0.7510	33 / 33
6611		-0.0230	-0.0262	-0.0473	0.5832	40 / 40
6612		-0.0154	-0.0581	-0.0577	0.7471	37 / 38
6616		0.0020	-0.0344	-0.0558	0.5211	33 / 33
6617		-0.0116	-0.0037	0.0385	0.5939	23 / 23
6618		-0.0114	-0.0025	0.0064	0.5145	40 / 40
6619		0.0152	0.0010	0.0319	0.2758	41 / 41
6620		0.0129	-0.0454	0.0128	0.4009	38 / 38
6621		-0.0258	-0.0089	0.0283	0.2369	41 / 41
6622		-0.0065	0.0068	-0.0085	0.6414	37 / 37
6623		-0.0094	-0.0185	-0.0220	0.7976	40 / 40
6624		0.0480	-0.0069	-0.0131	0.7967	32 / 32

6625		-0.0540	-0.0294	-0.0276	0.6044	37 / 37
6626		-0.0239	-0.0121	-0.0606	0.4158	35 / 35
6627		-0.0334	-0.0275	-0.0265	0.6220	33 / 33
6628		-0.0391	-0.0353	-0.0489	0.5279	39 / 39
6629		-0.0518	-0.0321	-0.0469	0.4834	37 / 37
6630		-0.0609	-0.0165	-0.0372	0.4283	34 / 34
6631		-0.0351	-0.0333	-0.0411	0.6086	32 / 32
6632		-0.0495	-0.0250	-0.0225	0.6295	34 / 34
6633		-0.0112	-0.0228	-0.0340	0.4433	38 / 38
6634		-0.0178	-0.0027	-0.0310	0.4217	35 / 35
6635		-0.0051	-0.0011	-0.0308	0.4881	39 / 39
6636		-0.0479	-0.0350	-0.0426	0.5606	41 / 41
6637		-0.0112	-0.0144	-0.0364	0.7188	27 / 27
6638		-0.0282	-0.0132	-0.0310	0.4565	27 / 27
6639		-0.0543	-0.0072	-0.0367	0.5828	38 / 38
6640		-0.0397	-0.0251	-0.0427	0.5258	34 / 34
6641		-0.0479	-0.0192	-0.0419	0.5835	37 / 37
**Mean [m]**		-0.000911	-0.004161	0.012537		
**Sigma [m]**		0.025056	0.024329	0.046599		
**RMS Error [m]**		0.025073	0.024683	0.048256		

Localisation accuracy per GCP and mean errors in the three coordinate directions. The last column counts the number of calibrated images where the GCP has been automatically verified vs. manually marked.

Absolute Geolocation Variance

Min Error [m]	Max Error [m]	Geolocation Error X [%]	Geolocation Error Y [%]	Geolocation Error Z [%]
-	-15.00	0.00	0.23	0.00
-15.00	-12.00	0.00	0.00	0.00
-12.00	-9.00	0.00	0.00	0.00
-9.00	-6.00	0.00	0.00	0.00
-6.00	-3.00	0.23	5.36	7.81
-3.00	0.00	47.20	47.20	39.98
0.00	3.00	52.45	38.00	45.45
3.00	6.00	0.12	8.97	6.76
6.00	9.00	0.00	0.00	0.00
9.00	12.00	0.00	0.00	0.00
12.00	15.00	0.00	0.00	0.00
15.00	-	0.00	0.23	0.00
**Mean [m]**		0.868188	1.677842	32.894031
**Sigma [m]**		1.114206	2.669680	2.140858
**RMS Error [m]**		1.412518	3.153149	32.963625

Min Error and Max Error represent geolocation error intervals between -1.5 and 1.5 times the maximum accuracy of all the images. Columns X, Y, Z show the percentage of images with geolocation errors within the predefined error intervals. The geolocation error is the difference between the initial and computed image positions. Note that the image geolocation errors do not correspond to the accuracy of the observed 3D points.

Geolocation Bias	X	Y	Z
Translation [m]	0.868194	1.677835	32.894032

Bias between image initial and computed geolocation given in output coordinate system.

Relative Geolocation Variance

Relative Geolocation Error	Images X [%]	Images Y [%]	Images Z [%]
[-1.00, 1.00]	100.00	98.72	100.00
[-2.00, 2.00]	100.00	99.53	100.00

[-3.00, 3.00]	100.00	99.53	100.00
**Mean of Geolocation Accuracy [m]**	5.000000	5.000000	10.000000
**Sigma of Geolocation Accuracy [m]**	0.000000	0.000000	0.000000

Images X, Y, Z represent the percentage of images with a relative geolocation error in X, Y, Z.

Initial Processing Details

System Information

Hardware	CPU: Intel(R) Core(TM) i9-7940X CPU @ 3.10GHz<br>RAM: 128GB<br>GPU: NVIDIA GeForce GTX 1080 Ti (Driver: 22.21.13.8541), RDPDD Chained DD (Driver: unknown), RDP Encoder Mirror Driver (Driver: unknown), RDP Reflector Display Driver (Driver: unknown)
Operating System	Windows 7 Ultimate, 64-bit

Coordinate Systems

Image Coordinate System	WGS 84 (EGM96 Geoid)
Ground Control Point (GCP) Coordinate System	ETRS89 / Poland CS2000 zone 6 (EGM96 Geoid)
Output Coordinate System	ETRS89 / Poland CS2000 zone 6 (EGM96 Geoid)

Processing Options

Detected Template	No Template Available
Keypoints Image Scale	Full, Image Scale: 1
Advanced: Matching Image Pairs	Aerial Grid or Corridor
Advanced: Matching Strategy	Use Geometrically Verified Matching: yes
Advanced: Keypoint Extraction	Targeted Number of Keypoints: Automatic
Advanced: Calibration	Calibration Method: Standard<br>Internal Parameters Optimization: All<br>External Parameters Optimization: All<br>Rematch: Auto, no

Point Cloud Densification details

Processing Options

Image Scale	multiscale, 1 (Original image size, Slow)
Point Density	Optimal
Minimum Number of Matches	3
3D Textured Mesh Generation	yes
3D Textured Mesh Settings:	Resolution: Medium Resolution (default)<br>Color Balancing: no
LOD	Generated: no
Advanced: 3D Textured Mesh Settings	Sample Density Divider: 1
Advanced: Image Groups	group1
Advanced: Use Processing Area	yes
Advanced: Use Annotations	yes
Time for Point Cloud Densification	04h:48m:11s
Time for Point Cloud Classification	NA
Time for 3D Textured Mesh Generation	27m:15s

Results

Number of Generated Tiles	10
Number of 3D Densified Points	271610389
Average Density (per m^3)	200.15
