# Supplementary material for: Multi-Variant Accuracy Evaluation of UAV Imaging Surveys: A Case Study on Investment Area
Source: Sensors (Basel). 2019 Nov 28;19(23):5229. doi: 10.3390/s19235229 (PMC6929115; doi:10.3390/s19235229)
Supplement: Supplementary file 1 [file sensors-19-05229-s001.zip › supplementary_files/App2_V2_report.pdf]

Quality Report

Generated with Pix4Ddiscovery version 4.3.31

Important: Click on the different icons for:

Help to analyze the results in the Quality Report

Additional information about the sections

Click here for additional tips to analyze the Quality Report

Summary

Project	Sosnowiec 2_nowa wersja
Processed	2019-02-08 02:36:57
Camera Model Name(s)	NEX-5T_0.0_4912x3264 (RGB)
Average Ground Sampling Distance (GSD)	4.58 cm / 1.80 in
Area Covered	1.035 km^2 / 103.5144 ha / 0.40 sq. mi. / 255.9219 acres

Quality Check

**Images**	median of 45103 keypoints per image	✓
**Dataset**	858 out of 858 images calibrated (100%), all images enabled	✓
**Camera Optimization**	0.23% relative difference between initial and optimized internal camera parameters	✓
**Matching**	median of 19317.1 matches per calibrated image	✓
**Georeferencing**	yes, 10 GCPs (10 3D), mean RMS error = 0.022 m	✓

Preview

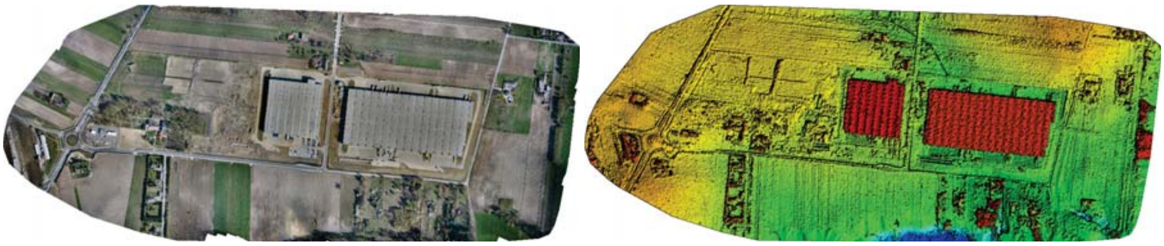

Figure 1: Orthomosaic and the corresponding sparse Digital Surface Model (DSM) before densification.

Calibration Details

Number of Calibrated Images	858 out of 858
Number of Geolocated Images	858 out of 858

Initial Image Positions

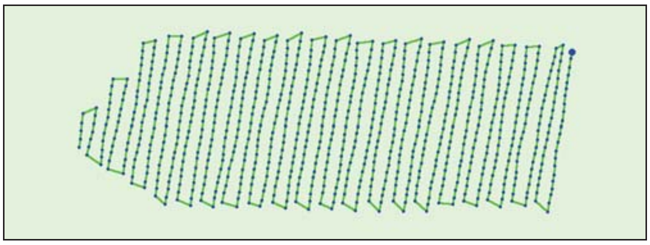

Figure 2: Top view of the initial image position. The green line follows the position of the images in time starting from the large blue dot.

Computed Image/GCPs/Manual Tie Points Positions

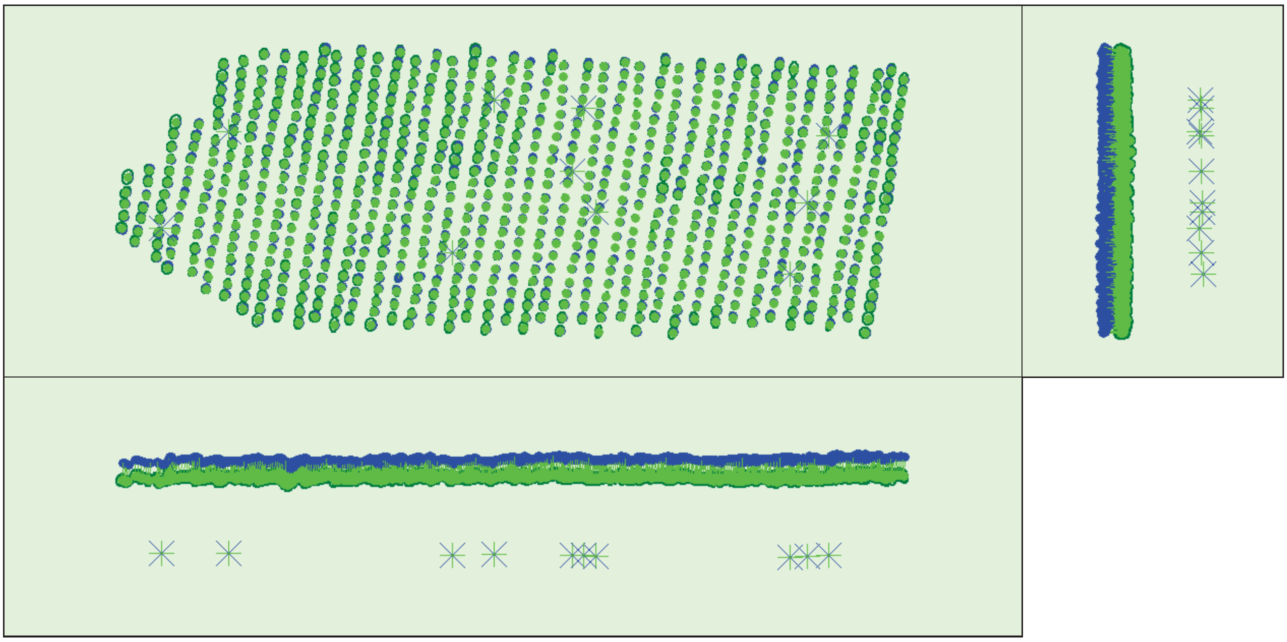

Uncertainty ellipses 500x magnified

Figure 3: Offset between initial (blue dots) and computed (green dots) image positions as well as the offset between the GCPs initial positions (blue crosses) and their computed positions (green crosses) in the top-view (XY plane), front-view (XZ plane), and side-view (YZ plane). Dark green ellipses indicate the absolute position uncertainty of the bundle block adjustment result.

Absolute camera position and orientation uncertainties

	X [m]	Y [m]	Z [m]	Omega [degree]	Phi [degree]	Kappa [degree]
Mean	0.013	0.017	0.022	0.006	0.004	0.002
Sigma	0.003	0.003	0.001	0.001	0.001	0.000

Overlap

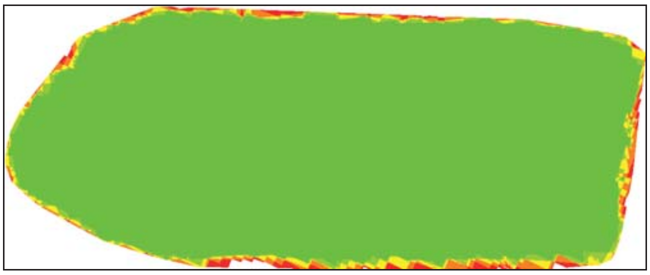

Number of overlapping images: 1 2 3 4 5+

Figure 4: Number of overlapping images computed for each pixel of the orthomosaic.
Red and yellow areas indicate low overlap for which poor results may be generated. Green areas indicate an overlap of over 5 images for every pixel. Good quality results will be generated as long as the number of keypoint matches is also sufficient for these areas (see Figure 5 for keypoint matches).

Bundle Block Adjustment Details

Number of 2D Keypoint Observations for Bundle Block Adjustment	16052530
Number of 3D Points for Bundle Block Adjustment	3111318
Mean Reprojection Error [pixels]	0.246

Internal Camera Parameters

NEX-5T_0.0_4912x3264 (RGB). Sensor Dimensions: 23.400 [mm] x 15.549 [mm]

EXIF ID: NEX-5T_0.0_4912x3264

	Focal Length	Principal Point x	Principal Point y	R1	R2	R3	T1	T2
Initial Values	3256.554 [pixel] 15.514 [mm]	2456.002 [pixel] 11.700 [mm]	1632.003 [pixel] 7.775 [mm]	-0.043	0.028	-0.005	0.000	-0.001
Optimized Values	3249.041 [pixel] 15.478 [mm]	2466.721 [pixel] 11.751 [mm]	1589.952 [pixel] 7.574 [mm]	-0.047	0.034	-0.009	0.000	0.000
Uncertainties (Sigma)	0.458 [pixel] 0.002 [mm]	0.065 [pixel] 0.000 [mm]	0.068 [pixel] 0.000 [mm]	0.000	0.000	0.000	0.000	0.000

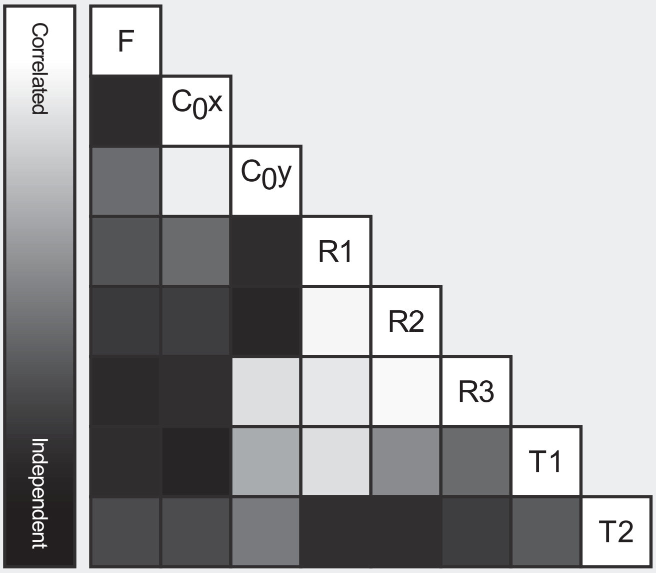

The correlation between camera internal parameters determined by the bundle adjustment. White indicates a full correlation between the parameters, ie. any change in one can be fully compensated by the other. Black indicates that the parameter is completely independent, and is not affected by other parameters.

The number of Automatic Tie Points (ATPs) per pixel, averaged over all images of the camera model, is color coded between black and white. White indicates that, on average, more than 16 ATPs have been extracted at the pixel location. Black indicates that, on average, 0 ATPs have been extracted at the pixel location. Click on the image to the see the average direction and magnitude of the re-projection error for each pixel. Note that the vectors are scaled for better visualization. The scale bar indicates the magnitude of 1 pixel error.

2D Keypoints Table

	Number of 2D Keypoints per Image	Number of Matched 2D Keypoints per Image
Median	45103	19317
Min	20002	5698
Max	68781	33639
Mean	42505	18709

3D Points from 2D Keypoint Matches

	Number of 3D Points Observed
In 2 Images	1319116

In 3 Images	532583
In 4 Images	295642
In 5 Images	190645
In 6 Images	132885
In 7 Images	99008
In 8 Images	77169
In 9 Images	61771
In 10 Images	50785
In 11 Images	41833
In 12 Images	35914
In 13 Images	30735
In 14 Images	27062
In 15 Images	23465
In 16 Images	21242
In 17 Images	18717
In 18 Images	16428
In 19 Images	14772
In 20 Images	13323
In 21 Images	11945
In 22 Images	10939
In 23 Images	9775
In 24 Images	8856
In 25 Images	8140
In 26 Images	7056
In 27 Images	6363
In 28 Images	5725
In 29 Images	5051
In 30 Images	4699
In 31 Images	4285
In 32 Images	3773
In 33 Images	3487
In 34 Images	3038
In 35 Images	2738
In 36 Images	2360
In 37 Images	2000
In 38 Images	1770
In 39 Images	1473
In 40 Images	1249
In 41 Images	984
In 42 Images	831
In 43 Images	634
In 44 Images	421
In 45 Images	286
In 46 Images	181
In 47 Images	87
In 48 Images	45
In 49 Images	21
In 50 Images	9
In 51 Images	2

2D Keypoint Matches

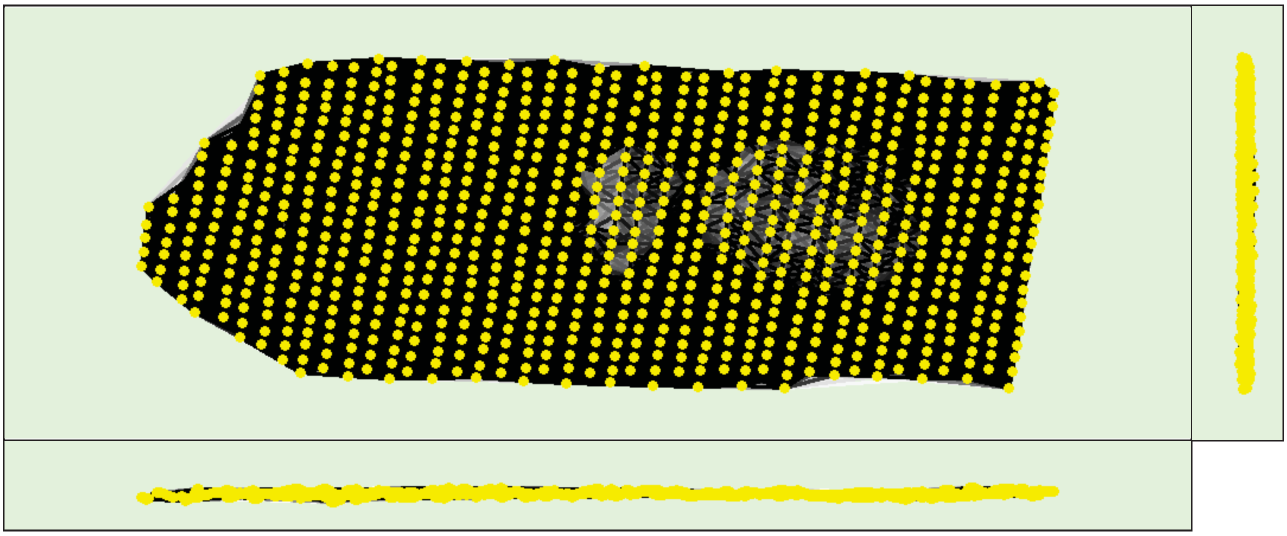

Figure 5: Computed image positions with links between matched images. The darkness of the links indicates the number of matched 2D keypoints between the images. Bright links indicate weak links and require manual tie points or more images.

Geolocation Details

Ground Control Points

GCP Name	Accuracy XY/Z [m]	Error X [m]	Error Y [m]	Error Z [m]	Projection Error [pixel]	Verified/Marked
201 (3D)	0.030/ 0.050	-0.005	-0.021	0.002	0.817	34 / 34
202 (3D)	0.030/ 0.050	0.006	0.013	0.010	0.750	31 / 31
203 (3D)	0.030/ 0.050	-0.005	0.019	-0.015	0.787	39 / 39
209 (3D)	0.030/ 0.050	0.016	-0.022	-0.044	0.490	37 / 37
207 (3D)	0.030/ 0.050	-0.019	-0.017	0.069	0.469	35 / 35
211 (3D)	0.030/ 0.050	0.015	0.027	-0.014	0.539	44 / 44
210 (3D)	0.030/ 0.050	-0.000	-0.005	0.033	0.302	43 / 43
206 (3D)	0.030/ 0.050	0.004	0.029	0.015	0.418	35 / 35
205 (3D)	0.030/ 0.050	-0.014	-0.034	0.008	0.864	40 / 40
204 (3D)	0.030/ 0.050	0.002	0.011	-0.062	0.913	38 / 39
**Mean [m]**		-0.000019	-0.000056	0.000192		
**Sigma [m]**		0.010521	0.021536	0.035269		
**RMS Error [m]**		0.010521	0.021536	0.035270		

0 out of 208 check points have been labeled as inaccurate.

Check Point Name	Accuracy XY/Z [m]	Error X [m]	Error Y [m]	Error Z [m]	Projection Error [pixel]	Verified/Marked
103		0.0155	0.0154	-0.0017	0.4630	42 / 42
101		-0.0059	0.0115	0.0115	0.4371	38 / 38
100		-0.0077	-0.0300	0.0327	0.3832	36 / 36
104		-0.0464	-0.0120	0.0015	0.5354	39 / 39
102		-0.0184	-0.0184	0.0104	0.3617	43 / 43
k1		0.0337	-0.0504	0.1307	0.5087	38 / 38
k2		0.0029	0.0261	0.0084	0.4655	43 / 43
k3		0.0895	-0.0734	0.1811	0.6206	26 / 26
k4		0.0237	-0.0219	0.0803	0.5097	36 / 36
k5		-0.0227	0.0335	-0.0154	0.4429	38 / 38
k6		-0.0273	0.0116	0.0613	0.7270	29 / 29
k7		-0.0770	0.0046	0.1529	0.5405	28 / 28
k8		-0.0398	-0.0312	0.1225	0.5937	30 / 30
k11		-0.0116	-0.0219	-0.0344	0.5373	43 / 43

k13		-0.0022	0.0075	0.0002	0.4308	29 / 29
k14		0.0018	0.0069	-0.0007	0.4969	38 / 38
10000		-0.0259	-0.0080	0.0381	0.6039	32 / 32
10001		0.0007	-0.0002	0.0541	0.5980	35 / 35
10002		0.0049	-0.0308	-0.0018	0.4984	37 / 37
10003		0.0021	-0.0407	-0.0258	0.5508	40 / 40
10004		0.0185	-0.0154	0.0209	0.5222	39 / 39
10005		0.0246	-0.0223	0.0148	0.5845	43 / 43
10006		-0.0006	-0.0235	0.0034	0.4274	42 / 42
10007		0.0002	-0.0201	-0.0146	0.4733	41 / 41
10008		0.0103	-0.0307	-0.0220	0.4252	38 / 38
10009		0.0042	-0.0282	-0.0079	0.5294	42 / 42
10010		0.0031	-0.0287	-0.0188	0.5013	44 / 44
10011		0.0062	-0.0397	-0.0215	0.5641	33 / 33
10012		-0.0383	0.0075	0.0060	0.5566	40 / 40
10013		-0.0230	0.0007	0.0036	0.4321	38 / 38
10014		0.0019	0.0109	0.0156	0.3815	42 / 42
10015		0.0135	0.0028	0.0364	0.4336	40 / 40
10016		-0.0244	-0.0020	0.0314	0.5236	43 / 43
10017		0.0032	-0.0144	-0.0228	0.4393	42 / 42
10018		0.0026	-0.0052	-0.0021	0.3953	22 / 22
10019		-0.0134	0.0148	0.0538	0.6278	42 / 42
10020		-0.0265	0.0089	0.0232	0.4708	33 / 33
10021		-0.0009	-0.0018	0.0385	0.4315	45 / 45
10022		0.0188	0.0278	0.0130	0.5252	42 / 42
10023		0.0238	-0.0079	0.0017	0.4201	38 / 38
10024		-0.0083	-0.0025	-0.0228	0.3985	46 / 46
10025		0.0079	-0.0023	0.0039	0.3904	44 / 44
10026		-0.0073	0.0213	0.0031	0.4584	36 / 36
10027		-0.0120	0.0163	-0.0019	0.5107	41 / 41
10028		0.0003	0.0019	0.0098	0.4264	43 / 43
208		-0.0026	-0.0171	-0.0017	0.6304	38 / 38
10030		-0.0059	0.0297	-0.0127	0.3964	37 / 37
10031		0.0353	0.0258	0.0058	0.5306	27 / 27
10032		0.0433	0.0353	0.0256	0.4658	27 / 27
10033		0.0085	0.0294	0.0228	0.3867	40 / 40
10034		0.0320	-0.0100	0.0090	0.4022	38 / 38
10035		0.0185	-0.0273	0.0625	0.4041	35 / 35
10036		0.0203	-0.0073	0.0118	0.5652	27 / 27
10037		0.0046	-0.0131	-0.0041	0.3948	39 / 39
10038		0.0008	0.0055	-0.0055	0.3828	42 / 42
10039		0.0098	-0.0087	-0.0143	0.4408	43 / 43
10040		-0.0232	-0.0116	0.0005	0.4975	31 / 31
10041		-0.0147	-0.0253	-0.0002	0.5962	35 / 35
10044		0.0086	-0.0469	-0.0238	0.5666	38 / 38
10045		0.0191	-0.0411	-0.0552	0.4126	36 / 36
10046		-0.0172	-0.0112	-0.0711	0.3860	38 / 38
10047		-0.0302	-0.0064	0.0748	0.7363	31 / 31
10048		-0.0163	-0.0230	0.0459	0.6473	32 / 32
10049		-0.0225	0.0273	0.0419	0.8337	32 / 32
10051		0.0001	0.0174	0.0146	0.3949	44 / 44
10052		-0.0040	0.0033	0.0239	0.5352	35 / 35
10053		-0.0148	0.0129	0.0135	0.4125	40 / 40
10054		-0.0135	-0.0172	-0.0023	0.4314	38 / 38
10055		-0.0009	0.0099	-0.0132	0.5769	39 / 39
10056		-0.0407	0.0093	0.0051	0.5312	38 / 38
10058		-0.0746	0.0855	0.0377	0.5441	44 / 44
10059		-0.0133	-0.0241	-0.0509	0.3864	40 / 40
10060		-0.0314	-0.0250	-0.0219	0.3626	43 / 43

10061		0.0059	0.0251	0.0461	0.4967	29 / 29
10062		-0.0050	-0.0251	-0.0021	0.4860	41 / 41
10063		0.0155	-0.0463	-0.0279	0.4856	38 / 38
10064		0.0370	0.0177	-0.0338	0.4592	43 / 43
10065		0.0288	0.0260	-0.0138	0.3690	44 / 44
10066		0.0565	0.0283	-0.0247	0.4491	41 / 41
10067		0.0311	0.0298	-0.0140	0.3805	42 / 42
10068		0.0132	0.1004	-0.0226	0.4592	43 / 43
10074		-0.0195	0.0454	-0.0350	0.6989	41 / 41
10075		-0.0141	0.0435	0.0099	0.3295	41 / 41
10076		0.0054	0.0203	-0.0039	0.3628	40 / 40
10077		-0.0172	0.0527	-0.0170	0.7435	38 / 38
10078		0.0262	0.0101	-0.0162	0.3460	39 / 39
10080		0.0135	-0.0629	-0.0919	0.7049	35 / 35
10081		-0.0250	-0.0262	0.0002	0.3669	37 / 37
10082		0.0087	-0.0391	-0.0316	0.4363	39 / 39
10083		0.0534	-0.0179	-0.0606	0.5986	37 / 37
10085		0.0294	-0.0337	0.0060	0.3464	44 / 44
15000		0.0062	-0.0416	-0.0371	0.3820	46 / 46
15001		-0.0284	-0.0433	-0.0422	0.3978	39 / 39
15002		0.0129	0.0146	-0.0797	0.4839	41 / 41
15003		-0.0010	0.0330	-0.0165	0.4874	45 / 45
15004		-0.0016	0.0128	-0.0272	0.3849	43 / 43
15005		-0.0011	0.0212	-0.0236	0.5528	38 / 38
15006		0.0084	0.0324	-0.0307	0.4756	43 / 43
15007		0.0129	-0.0047	0.0089	0.4900	38 / 38
15008		-0.0198	0.0166	0.0444	0.8099	38 / 38
15009		-0.0082	0.0103	0.0346	0.5779	40 / 40
15010		0.0063	0.0360	0.0219	0.6715	38 / 38
20000		-0.0121	0.0180	0.0115	0.7838	42 / 42
20001		-0.0076	0.0061	0.0286	0.5431	38 / 38
20002		0.0032	0.0204	0.0274	0.4634	38 / 38
20003		-0.0231	-0.0075	0.0203	0.4541	39 / 39
20004		-0.0028	-0.0124	0.0141	0.4279	36 / 36
20005		-0.0510	-0.0262	-0.0043	0.5380	35 / 35
20008		-0.0060	-0.0001	0.0044	0.3956	44 / 44
20009		0.0096	-0.0070	-0.0131	0.3922	43 / 43
20010		-0.0242	0.0220	-0.0132	0.4176	45 / 45
20011		-0.0182	-0.0029	0.0037	0.3902	47 / 47
20012		0.0073	-0.0025	-0.0083	0.3530	44 / 44
20013		0.0100	-0.0028	0.0085	0.3585	41 / 41
20014		-0.0005	0.0057	-0.0037	0.3529	43 / 43
20016		0.0005	0.0128	-0.0413	0.3416	46 / 46
20017		0.0118	0.0226	-0.0244	0.3727	40 / 40
20018		0.0231	0.0196	-0.0115	0.3360	44 / 44
20019		0.0281	0.0113	0.0019	0.4189	39 / 39
20020		0.0101	0.0359	-0.0012	0.4489	40 / 40
20021		0.0111	-0.0009	0.0171	0.2704	40 / 40
20022		-0.0123	-0.0528	0.0155	0.3191	42 / 42
20023		0.0051	0.0015	-0.0003	0.3504	45 / 45
20024		0.0012	0.0376	-0.0280	0.4395	41 / 41
20025		0.0169	0.0455	-0.0195	0.3590	42 / 42
20026		0.0168	0.0351	-0.0169	0.4191	43 / 43
20027		0.0107	0.0690	-0.0201	0.3947	40 / 40
20028		-0.0006	0.0094	-0.0026	0.4255	41 / 41
20029		0.0186	-0.0183	-0.0176	0.7863	41 / 41
20031		-0.0125	0.0204	-0.0019	0.4483	45 / 45
25000		0.0199	0.0078	-0.0223	0.2562	40 / 40
25001		0.0063	0.0047	0.0159	0.3189	40 / 40

25003		0.0037	-0.0202	0.0120	0.3962	40 / 40
25004		0.0058	-0.0002	-0.0089	0.3569	33 / 33
25005		0.0047	-0.0121	0.0078	0.3178	39 / 39
25006		-0.0313	-0.0447	-0.0374	0.4973	38 / 38
25008		-0.0367	-0.0028	-0.0417	0.3608	38 / 38
25009		0.0230	-0.0524	-0.0476	0.3770	42 / 42
25010		0.0036	-0.0185	-0.0449	0.2888	41 / 41
25013		-0.0064	0.0158	-0.0283	0.4099	38 / 38
25014		-0.0147	-0.0076	-0.0410	0.3106	37 / 37
25015		-0.0282	-0.0096	-0.0353	0.4441	41 / 41
25016		-0.0289	-0.0085	-0.0315	0.2908	42 / 42
25017		0.0205	-0.0168	0.0448	0.4461	37 / 37
25018		0.0054	0.0008	0.0310	0.3983	38 / 38
25019		-0.0156	-0.0092	0.0206	0.4793	39 / 39
25020		-0.0120	0.0068	-0.0085	0.3755	33 / 33
25021		0.0180	0.0259	0.0034	0.3734	36 / 36
25022		0.0152	0.0071	0.0176	0.4797	34 / 34
25023		-0.0033	0.0256	0.0027	0.4265	40 / 40
25024		0.0038	-0.0143	-0.0051	0.3517	40 / 40
25029		0.0105	-0.0056	0.0073	0.3447	42 / 42
25030		-0.0106	-0.0099	0.0145	0.3996	44 / 44
25032		-0.0747	-0.0251	-0.0266	0.4292	36 / 36
25033		-0.0236	-0.0197	0.0033	0.3789	41 / 41
25034		-0.0170	-0.0187	-0.0041	0.4164	36 / 36
40000		0.0115	-0.0195	-0.0991	0.3121	42 / 42
40001		0.0263	0.0158	-0.0157	0.2947	43 / 43
40002		0.0213	0.0388	-0.0305	0.3870	42 / 42
40003		0.0136	0.0435	-0.0021	0.3926	39 / 39
40004		0.0091	0.0047	-0.0090	0.3080	37 / 37
40005		0.0216	0.0241	-0.0287	0.2941	29 / 29
45000		0.0305	-0.0278	0.0105	0.4245	44 / 44
45001		-0.0076	0.0043	0.0068	0.4211	34 / 34
45002		0.0109	-0.0325	0.0068	0.5052	41 / 41
10043		-0.0161	-0.0087	0.0357	0.6836	35 / 35
10084		-0.0104	-0.0056	0.0633	0.5708	30 / 30
20015		-0.0005	-0.0127	-0.0443	0.3392	45 / 45
20007		-0.0009	-0.0077	-0.0087	0.3936	24 / 24
25027		-0.0030	-0.0114	-0.0418	0.3258	43 / 43
k9		-0.0078	-0.0161	-0.0263	0.6134	42 / 42
6600		0.0025	-0.0205	-0.0368	0.4278	41 / 41
6602		0.0035	0.0272	-0.0273	0.3956	37 / 37
6603		-0.0021	-0.0210	-0.0037	0.4456	40 / 40
6605		0.0111	0.0172	0.0087	0.4997	40 / 40
6606		0.0161	0.0292	0.0080	0.5779	25 / 25
6607		0.0026	0.0095	-0.0385	0.7322	35 / 35
6608		-0.0068	-0.0175	0.0020	0.6824	21 / 21
6609		-0.0166	0.0021	-0.0034	0.3968	42 / 42
6610		0.0010	0.0022	0.0179	0.7532	33 / 33
6611		-0.0191	-0.0252	-0.0674	0.5787	40 / 40
6612		-0.0111	-0.0559	-0.0767	0.7528	38 / 38
6616		-0.0057	-0.0389	-0.0421	0.5165	33 / 33
6617		-0.0112	-0.0040	0.0231	0.5943	23 / 23
6618		-0.0100	-0.0025	-0.0115	0.5166	40 / 40
6619		0.0171	0.0008	0.0161	0.2796	41 / 41
6620		0.0151	-0.0455	-0.0037	0.3984	38 / 38
6621		-0.0230	-0.0090	0.0114	0.2365	41 / 41
6622		-0.0038	0.0060	-0.0201	0.6412	37 / 37
6623		-0.0080	-0.0195	-0.0318	0.7970	40 / 40
6624		0.0481	-0.0088	-0.0191	0.7529	33 / 33

6625		-0.0528	-0.0305	-0.0334	0.6020	37 / 37
6626		-0.0225	-0.0134	-0.0660	0.4158	35 / 35
6627		-0.0320	-0.0286	-0.0316	0.6194	33 / 33
6628		-0.0379	-0.0363	-0.0539	0.5337	39 / 39
6629		-0.0506	-0.0330	-0.0514	0.4865	37 / 37
6630		-0.0597	-0.0174	-0.0413	0.4317	34 / 34
6631		-0.0338	-0.0343	-0.0445	0.6128	32 / 32
6632		-0.0483	-0.0257	-0.0248	0.6298	34 / 34
6633		-0.0107	-0.0236	-0.0364	0.4472	38 / 38
6634		-0.0171	-0.0036	-0.0343	0.4269	35 / 35
6635		-0.0044	-0.0017	-0.0348	0.4915	39 / 39
6636		-0.0472	-0.0356	-0.0476	0.5582	41 / 41
6637		-0.0088	-0.0140	-0.0287	0.6441	31 / 31
6638		-0.0275	-0.0141	-0.0349	0.4656	27 / 27
6639		-0.0538	-0.0079	-0.0423	0.5738	38 / 38
6640		-0.0392	-0.0257	-0.0468	0.5209	34 / 34
6641		-0.0477	-0.0198	-0.0449	0.5790	37 / 37
**Mean [m]**		-0.003198	-0.003045	-0.004155		
**Sigma [m]**		0.023301	0.025775	0.036500		
**RMS Error [m]**		0.023519	0.025954	0.036735		

Localisation accuracy per GCP and mean errors in the three coordinate directions. The last column counts the number of calibrated images where the GCP has been automatically verified vs. manually marked.

Absolute Geolocation Variance

Min Error [m]	Max Error [m]	Geolocation Error X [%]	Geolocation Error Y [%]	Geolocation Error Z [%]
-	-15.00	0.00	0.23	0.00
-15.00	-12.00	0.00	0.00	0.00
-12.00	-9.00	0.00	0.00	0.00
-9.00	-6.00	0.00	0.00	0.00
-6.00	-3.00	0.23	5.36	7.93
-3.00	0.00	47.32	47.20	39.74
0.00	3.00	52.33	38.00	45.45
3.00	6.00	0.12	8.97	6.88
6.00	9.00	0.00	0.00	0.00
9.00	12.00	0.00	0.00	0.00
12.00	15.00	0.00	0.00	0.00
15.00	-	0.00	0.23	0.00
**Mean [m]**		0.857247	1.669080	32.883897
**Sigma [m]**		1.118404	2.669237	2.146836
**RMS Error [m]**		1.409149	3.148119	32.953901

Min Error and Max Error represent geolocation error intervals between -1.5 and 1.5 times the maximum accuracy of all the images. Columns X, Y, Z show the percentage of images with geolocation errors within the predefined error intervals. The geolocation error is the difference between the initial and computed image positions. Note that the image geolocation errors do not correspond to the accuracy of the observed 3D points.

Geolocation Bias	X	Y	Z
Translation [m]	0.857253	1.669073	32.883898

Bias between image initial and computed geolocation given in output coordinate system.

Relative Geolocation Variance

Relative Geolocation Error	Images X [%]	Images Y [%]	Images Z [%]
[-1.00, 1.00]	100.00	98.72	100.00
[-2.00, 2.00]	100.00	99.53	100.00

[-3.00, 3.00]	100.00	99.53	100.00
**Mean of Geolocation Accuracy [m]**	5.000000	5.000000	10.000000
**Sigma of Geolocation Accuracy [m]**	0.000000	0.000000	0.000000

Images X, Y, Z represent the percentage of images with a relative geolocation error in X, Y, Z.

Initial Processing Details

System Information

Hardware	CPU: Intel(R) Core(TM) i9-7940X CPU @ 3.10GHz<br>RAM: 128GB<br>GPU: NVIDIA GeForce GTX 1080 Ti (Driver: 22.21.13.8541), RDPDD Chained DD (Driver: unknown), RDP Encoder Mirror Driver (Driver: unknown), RDP Reflector Display Driver (Driver: unknown)
Operating System	Windows 7 Ultimate, 64-bit

Coordinate Systems

Image Coordinate System	WGS 84 (EGM96 Geoid)
Ground Control Point (GCP) Coordinate System	ETRS89 / Poland CS2000 zone 6 (EGM96 Geoid)
Output Coordinate System	ETRS89 / Poland CS2000 zone 6 (EGM96 Geoid)

Processing Options

Detected Template	No Template Available
Keypoints Image Scale	Full, Image Scale: 1
Advanced: Matching Image Pairs	Aerial Grid or Corridor
Advanced: Matching Strategy	Use Geometrically Verified Matching: yes
Advanced: Keypoint Extraction	Targeted Number of Keypoints: Automatic
Advanced: Calibration	Calibration Method: Standard<br>Internal Parameters Optimization: All<br>External Parameters Optimization: All<br>Rematch: Auto, no

DSM, Orthomosaic and Index Details

Processing Options

DSM and Orthomosaic Resolution	1 x GSD (4.58 [cm/pixel])
DSM Filters	Noise Filtering: yes<br>Surface Smoothing: yes, Type: Sharp
