# Supplementary material for: Multi-Variant Accuracy Evaluation of UAV Imaging Surveys: A Case Study on Investment Area
Source: Sensors (Basel). 2019 Nov 28;19(23):5229. doi: 10.3390/s19235229 (PMC6929115; doi:10.3390/s19235229)
Supplement: Supplementary file 1 [file sensors-19-05229-s001.zip › supplementary_files/App3_V3_report.pdf]

Quality Report

Generated with Pix4Ddiscovery version 4.3.31

Important: Click on the different icons for:

Help to analyze the results in the Quality Report

Additional information about the sections

Click here for additional tips to analyze the Quality Report

Summary

Project	Sosnowiec 3_nowa wersja
Processed	2019-02-08 00:50:56
Camera Model Name(s)	NEX-5T_0.0_4912x3264 (RGB)
Average Ground Sampling Distance (GSD)	4.58 cm / 1.80 in
Area Covered	1.035 km^2 / 103.5069 ha / 0.40 sq. mi. / 255.9034 acres
Time for Initial Processing (without report)	56m:12s

Quality Check

**Images**	median of 45103 keypoints per image	✓
**Dataset**	858 out of 858 images calibrated (100%), all images enabled	✓
**Camera Optimization**	0.22% relative difference between initial and optimized internal camera parameters	✓
**Matching**	median of 19314.6 matches per calibrated image	✓
**Georeferencing**	yes, 10 GCPs (10 3D), mean RMS error = 0.019 m	✓

Preview

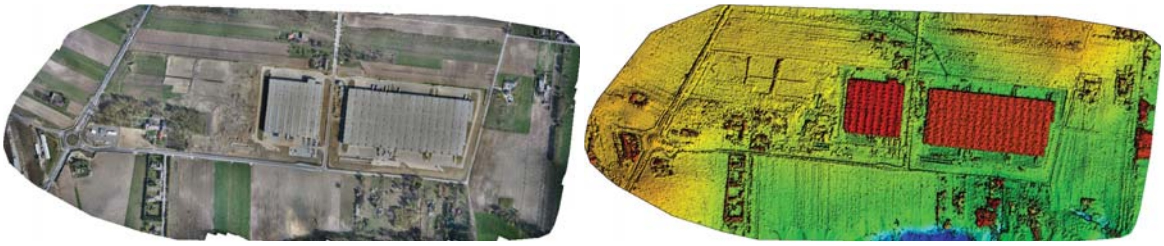

Figure 1: Orthomosaic and the corresponding sparse Digital Surface Model (DSM) before densification.

Calibration Details

Number of Calibrated Images	858 out of 858
Number of Geolocated Images	858 out of 858

Initial Image Positions

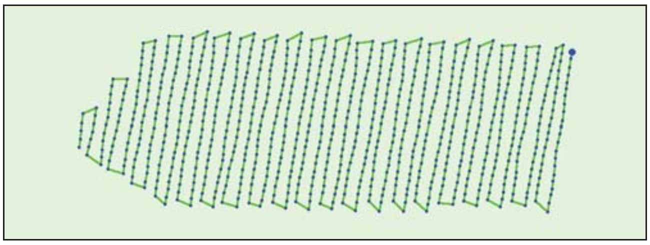

Figure 2: Top view of the initial image position. The green line follows the position of the images in time starting from the large blue dot.

Computed Image/GCPs/Manual Tie Points Positions

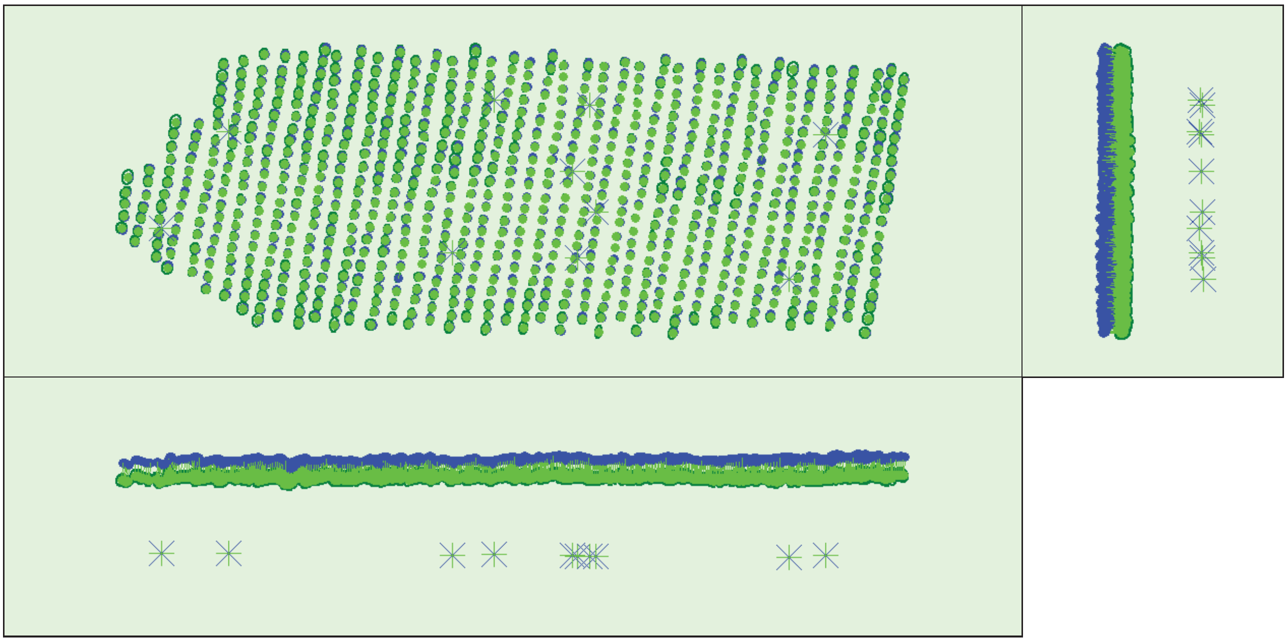

Uncertainty ellipses 500x magnified

Figure 3: Offset between initial (blue dots) and computed (green dots) image positions as well as the offset between the GCPs initial positions (blue crosses) and their computed positions (green crosses) in the top-view (XY plane), front-view (XZ plane), and side-view (YZ plane). Dark green ellipses indicate the absolute position uncertainty of the bundle block adjustment result.

Absolute camera position and orientation uncertainties

	X[m]	Y[m]	Z[m]	Omega [degree]	Phi [degree]	Kappa [degree]
Mean	0.013	0.017	0.022	0.006	0.004	0.002
Sigma	0.003	0.003	0.001	0.001	0.001	0.000

Overlap

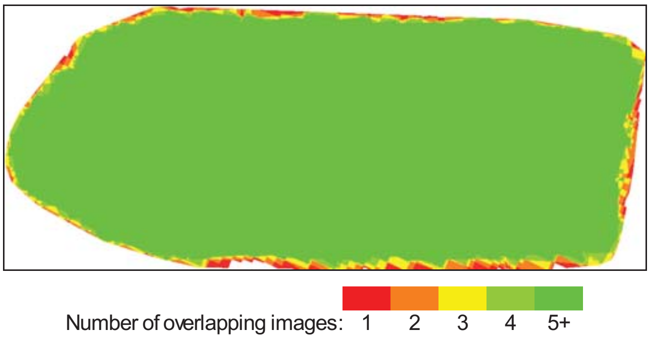

Figure 4: Number of overlapping images computed for each pixel of the orthomosaic.
Red and yellow areas indicate low overlap for which poor results may be generated. Green areas indicate an overlap of over 5 images for every pixel. Good quality results will be generated as long as the number of keypoint matches is also sufficient for these areas (see Figure 5 for keypoint matches).

Bundle Block Adjustment Details

Number of 2D Keypoint Observations for Bundle Block Adjustment	16052344
Number of 3D Points for Bundle Block Adjustment	3111314
Mean Reprojection Error [pixels]	0.246

Internal Camera Parameters

NEX-5T_0.0_4912x3264 (RGB). Sensor Dimensions: 23.400 [mm] x 15.549 [mm]

EXIF ID: NEX-5T_0.0_4912x3264

	Focal Length	Principal Point x	Principal Point y	R1	R2	R3	T1	T2
Initial Values	3256.554 [pixel]<br>15.514 [mm]	2456.002 [pixel]<br>11.700 [mm]	1632.003 [pixel]<br>7.775 [mm]	-0.043	0.028	-0.005	0.000	-0.001
Optimized Values	3249.096 [pixel]<br>15.478 [mm]	2466.738 [pixel]<br>11.751 [mm]	1589.944 [pixel]<br>7.574 [mm]	-0.047	0.035	-0.009	0.000	0.000
Uncertainties (Sigma)	0.458 [pixel]<br>0.002 [mm]	0.065 [pixel]<br>0.000 [mm]	0.068 [pixel]<br>0.000 [mm]	0.000	0.000	0.000	0.000	0.000

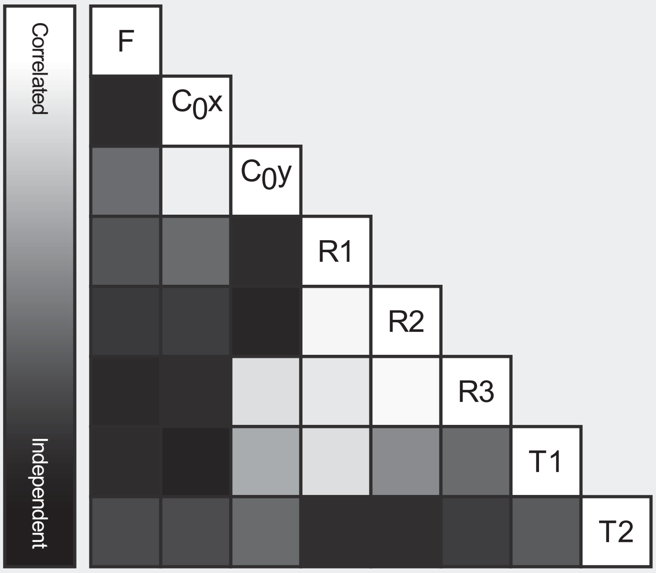

The correlation between camera internal parameters determined by the bundle adjustment. White indicates a full correlation between the parameters, ie. any change in one can be fully compensated by the other. Black indicates that the parameter is completely independent, and is not affected by other parameters.

The number of Automatic Tie Points (ATPs) per pixel, averaged over all images of the camera model, is color coded between black and white. White indicates that, on average, more than 16 ATPs have been extracted at the pixel location. Black indicates that, on average, 0 ATPs have been extracted at the pixel location. Click on the image to the see the average direction and magnitude of the re-projection error for each pixel. Note that the vectors are scaled for better visualization. The scale bar indicates the magnitude of 1 pixel error.

2D Keypoints Table

	Number of 2D Keypoints per Image	Number of Matched 2D Keypoints per Image
Median	45103	19315
Min	20002	5700
Max	68781	33641
Mean	42505	18709

3D Points from 2D Keypoint Matches

	Number of 3D Points Observed
In 2 Images	1319115

In 3 Images	532586
In 4 Images	295652
In 5 Images	190645
In 6 Images	132885
In 7 Images	99003
In 8 Images	77164
In 9 Images	61770
In 10 Images	50786
In 11 Images	41831
In 12 Images	35916
In 13 Images	30732
In 14 Images	27074
In 15 Images	23462
In 16 Images	21242
In 17 Images	18718
In 18 Images	16419
In 19 Images	14769
In 20 Images	13326
In 21 Images	11942
In 22 Images	10940
In 23 Images	9778
In 24 Images	8855
In 25 Images	8135
In 26 Images	7058
In 27 Images	6363
In 28 Images	5724
In 29 Images	5051
In 30 Images	4699
In 31 Images	4285
In 32 Images	3776
In 33 Images	3485
In 34 Images	3039
In 35 Images	2738
In 36 Images	2358
In 37 Images	2000
In 38 Images	1768
In 39 Images	1474
In 40 Images	1250
In 41 Images	985
In 42 Images	830
In 43 Images	634
In 44 Images	421
In 45 Images	286
In 46 Images	181
In 47 Images	87
In 48 Images	45
In 49 Images	21
In 50 Images	9
In 51 Images	2

2D Keypoint Matches

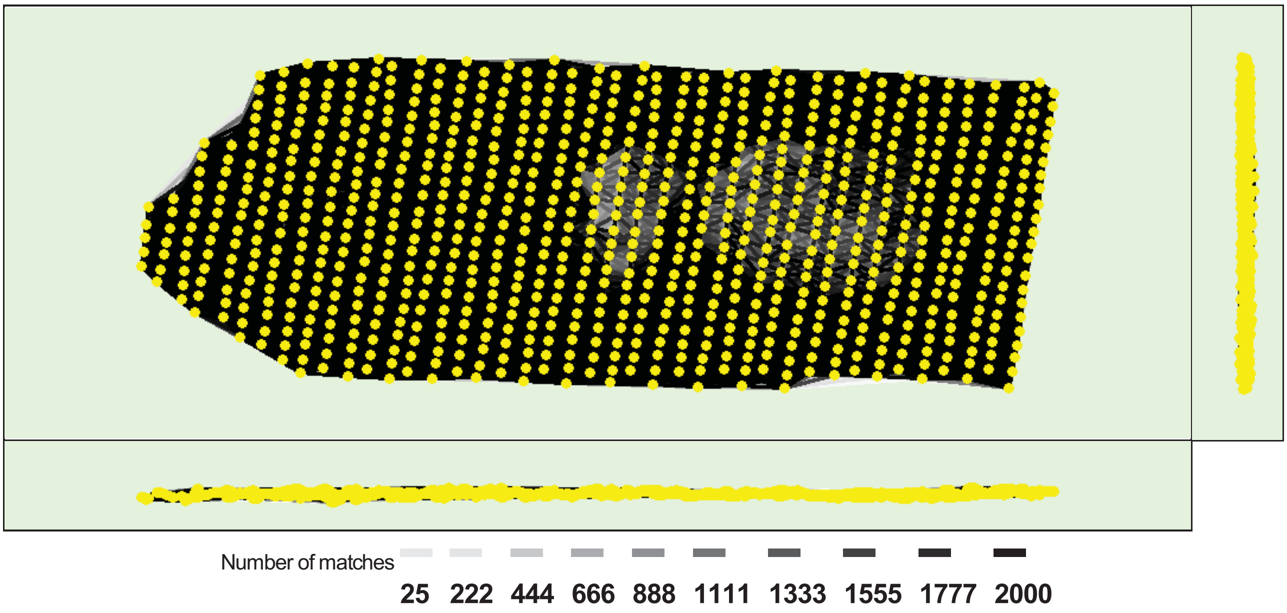

Figure 5: Computed image positions with links between matched images. The darkness of the links indicates the number of matched 2D keypoints between the images. Bright links indicate weak links and require manual tie points or more images.

Geolocation Details

Ground Control Points

GCP Name	Accuracy XY/Z [m]	Error X [m]	Error Y [m]	Error Z [m]	Projection Error [pixel]	Verified/Marked
103 (3D)	0.030/ 0.050	0.017	0.009	0.001	0.462	42 / 42
101 (3D)	0.030/ 0.050	0.012	0.010	-0.005	0.437	38 / 38
100 (3D)	0.030/ 0.050	0.008	-0.021	-0.021	0.383	36 / 36
102 (3D)	0.030/ 0.050	-0.018	-0.019	0.003	0.361	43 / 43
201 (3D)	0.030/ 0.050	-0.005	-0.022	0.001	0.817	34 / 34
202 (3D)	0.030/ 0.050	0.005	0.014	0.008	0.750	31 / 31
203 (3D)	0.030/ 0.050	-0.006	0.017	-0.015	0.787	39 / 39
207 (3D)	0.030/ 0.050	-0.017	-0.021	0.071	0.470	35 / 35
206 (3D)	0.030/ 0.050	0.005	0.026	0.016	0.418	35 / 35
204 (3D)	0.030/ 0.050	-0.001	0.006	-0.056	0.914	38 / 39
**Mean [m]**		-0.000012	-0.000043	0.000246		
**Sigma [m]**		0.010862	0.017627	0.030303		
**RMS Error [m]**		0.010862	0.017627	0.030304		

0 out of 208 check points have been labeled as inaccurate.

Check Point Name	Accuracy XY/Z [m]	Error X [m]	Error Y [m]	Error Z [m]	Projection Error [pixel]	Verified/Marked
104		-0.0492	-0.0143	0.0078	0.5353	39 / 39
k1		0.0344	-0.0544	0.1338	0.5083	38 / 38
k2		0.0037	0.0225	0.0109	0.4652	43 / 43
k3		0.0902	-0.0765	0.1832	0.6214	26 / 26
k4		0.0244	-0.0251	0.0824	0.5097	36 / 36
k5		-0.0219	0.0303	-0.0135	0.4427	38 / 38
k6		-0.0252	0.0087	0.0618	0.7268	29 / 29
k7		-0.0750	0.0014	0.1536	0.5403	28 / 28
k8		-0.0377	-0.0336	0.1221	0.5926	30 / 30
k11		-0.0125	-0.0238	-0.0363	0.5379	43 / 43
k13		0.0132	0.0122	-0.0312	0.4281	29 / 29
k14		0.0177	0.0097	-0.0242	0.4964	38 / 38
10000		-0.0090	-0.0083	0.0206	0.6040	32 / 32
10001		0.0159	0.0054	0.0179	0.5976	35 / 35

10002		0.0183	-0.0235	-0.0464	0.4976	37 / 37
10003		0.0137	-0.0341	-0.0650	0.5502	40 / 40
10004		0.0260	-0.0114	-0.0047	0.5224	39 / 39
10005		0.0316	-0.0184	-0.0099	0.5839	43 / 43
10006		0.0053	-0.0205	-0.0176	0.4269	42 / 42
10007		0.0045	-0.0180	-0.0313	0.4730	41 / 41
10008		0.0142	-0.0288	-0.0379	0.4255	38 / 38
10009		0.0072	-0.0270	-0.0211	0.5292	42 / 42
10010		0.0060	-0.0276	-0.0319	0.5011	44 / 44
10011		0.0084	-0.0392	-0.0322	0.5650	33 / 33
10012		-0.0370	0.0072	-0.0018	0.5567	40 / 40
10013		-0.0218	0.0002	-0.0032	0.4318	38 / 38
10014		0.0028	0.0101	0.0099	0.3812	42 / 42
10015		0.0137	0.0013	0.0334	0.4333	40 / 40
10016		-0.0249	-0.0038	0.0291	0.5236	43 / 43
10017		0.0024	-0.0165	-0.0242	0.4393	42 / 42
10018		0.0014	-0.0076	-0.0023	0.3953	22 / 22
10019		-0.0155	0.0109	0.0593	0.6275	42 / 42
10020		-0.0285	0.0048	0.0286	0.4710	33 / 33
10021		-0.0016	-0.0061	0.0425	0.4320	45 / 45
10022		0.0186	0.0229	0.0170	0.5256	42 / 42
10023		0.0237	-0.0127	0.0055	0.4195	38 / 38
10024		-0.0091	-0.0076	-0.0184	0.3985	46 / 46
10025		0.0072	-0.0076	0.0083	0.3904	44 / 44
10026		-0.0074	0.0158	0.0071	0.4584	36 / 36
10027		-0.0116	0.0113	0.0017	0.5106	41 / 41
10028		0.0008	-0.0030	0.0133	0.4265	43 / 43
208		-0.0014	-0.0226	0.0012	0.6305	38 / 38
10030		-0.0041	0.0245	-0.0101	0.3963	37 / 37
10031		0.0372	0.0205	0.0081	0.5308	27 / 27
10032		0.0469	0.0301	0.0265	0.4659	27 / 27
10033		0.0149	0.0247	0.0211	0.3868	40 / 40
10034		0.0382	-0.0137	0.0066	0.4029	38 / 38
10035		0.0246	-0.0309	0.0600	0.4049	35 / 35
10036		0.0274	-0.0107	0.0083	0.5660	27 / 27
10037		0.0151	-0.0164	-0.0107	0.3945	39 / 39
10038		0.0129	0.0028	-0.0144	0.3826	42 / 42
10039		0.0245	-0.0102	-0.0272	0.4399	43 / 43
10040		-0.0073	-0.0032	-0.0498	0.4979	31 / 31
10041		0.0012	-0.0165	-0.0531	0.5958	35 / 35
209		0.0314	-0.0124	-0.0994	0.4903	37 / 37
10044		0.0203	-0.0395	-0.0646	0.5661	38 / 38
10045		0.0286	-0.0350	-0.0895	0.4127	36 / 36
10046		-0.0162	-0.0113	-0.0799	0.3855	38 / 38
10047		-0.0285	-0.0078	0.0712	0.7361	31 / 31
10048		-0.0146	-0.0244	0.0425	0.6472	32 / 32
10049		-0.0207	0.0254	0.0404	0.8339	32 / 32
10051		0.0007	0.0129	0.0179	0.3949	44 / 44
10052		-0.0032	0.0008	0.0248	0.5351	35 / 35
10053		-0.0144	0.0115	0.0102	0.4125	40 / 40
10054		-0.0131	-0.0185	-0.0057	0.4311	38 / 38
10055		-0.0025	0.0047	-0.0083	0.5772	39 / 39
10056		-0.0429	0.0047	0.0106	0.5305	38 / 38
10058		-0.0767	0.0813	0.0432	0.5440	44 / 44
10059		-0.0101	-0.0223	-0.0662	0.3864	40 / 40
10060		-0.0313	-0.0261	-0.0269	0.3632	43 / 43
10061		0.0057	0.0233	0.0445	0.4960	29 / 29
10062		-0.0053	-0.0266	-0.0056	0.4859	41 / 41
10063		0.0149	-0.0480	-0.0308	0.4859	38 / 38

10064		0.0398	0.0121	-0.0323	0.4600		43 / 43
10065		0.0334	0.0206	-0.0136	0.3685		44 / 44
10066		0.0617	0.0220	-0.0248	0.4484		41 / 41
10067		0.0364	0.0238	-0.0143	0.3801		42 / 42
10068		0.0187	0.0943	-0.0230	0.4590		43 / 43
10074		-0.0138	0.0395	-0.0357	0.6990		41 / 41
10075		-0.0077	0.0385	0.0082	0.3294		41 / 41
10076		0.0114	0.0144	-0.0047	0.3623		40 / 40
10077		-0.0090	0.0471	-0.0200	0.7423		38 / 38
10078		0.0372	0.0057	-0.0225	0.3454		39 / 39
211		0.0327	0.0261	-0.0311	0.5380		44 / 44
10080		0.0157	-0.0619	-0.1046	0.7049		35 / 35
10081		-0.0230	-0.0254	-0.0117	0.3670		37 / 37
10082		0.0108	-0.0384	-0.0429	0.4360		39 / 39
10083		0.0536	-0.0240	-0.0570	0.5980		37 / 37
10085		0.0295	-0.0350	0.0020	0.3460		44 / 44
15000		0.0122	-0.0377	-0.0606	0.3816		46 / 46
15001		-0.0238	-0.0405	-0.0618	0.3975		39 / 39
15002		0.0241	0.0101	-0.0861	0.4839		41 / 41
15003		0.0107	0.0283	-0.0233	0.4871		45 / 45
15004		0.0122	0.0090	-0.0368	0.3849		43 / 43
15005		0.0149	0.0186	-0.0368	0.5523		38 / 38
15006		0.0256	0.0306	-0.0464	0.4751		43 / 43
15007		0.0299	-0.0042	-0.0109	0.4905		38 / 38
15008		-0.0030	0.0179	0.0233	0.8104		38 / 38
15009		0.0083	0.0132	0.0097	0.5775		40 / 40
15010		0.0224	0.0406	-0.0100	0.6724		38 / 38
20000		0.0048	0.0175	-0.0058	0.7834		42 / 42
20001		0.0088	0.0075	0.0084	0.5425		38 / 38
20002		0.0193	0.0230	0.0043	0.4633		38 / 38
20003		-0.0073	-0.0034	-0.0081	0.4538		39 / 39
20004		0.0125	-0.0068	-0.0225	0.4281		36 / 36
20005		-0.0495	-0.0271	-0.0094	0.5372		35 / 35
20008		-0.0057	-0.0015	0.0009	0.3956		44 / 44
20009		0.0094	-0.0087	-0.0159	0.3918		43 / 43
20010		-0.0251	0.0174	-0.0088	0.4177		45 / 45
20011		-0.0188	-0.0077	0.0079	0.3900		47 / 47
20012		0.0070	-0.0073	-0.0043	0.3527		44 / 44
20013		0.0100	-0.0076	0.0123	0.3584		41 / 41
20014		-0.0002	0.0008	-0.0001	0.3528		43 / 43
20016		-0.0004	0.0074	-0.0367	0.3422		46 / 46
20017		0.0112	0.0172	-0.0201	0.3735		40 / 40
20018		0.0228	0.0141	-0.0074	0.3362		44 / 44
20019		0.0282	0.0056	0.0058	0.4198		39 / 39
20020		0.0122	0.0305	0.0010	0.4486		40 / 40
20021		0.0177	-0.0054	0.0150	0.2703		40 / 40
20022		-0.0054	-0.0572	0.0129	0.3194		42 / 42
20023		0.0124	-0.0028	-0.0034	0.3504		45 / 45
20024		0.0080	0.0321	-0.0298	0.4393		41 / 41
20025		0.0238	0.0401	-0.0214	0.3580		42 / 42
20026		0.0244	0.0298	-0.0195	0.4185		43 / 43
20027		0.0213	0.0647	-0.0260	0.3950		40 / 40
20028		0.0121	0.0061	-0.0114	0.4254		41 / 41
20029		0.0335	-0.0205	-0.0298	0.7868		41 / 41
20031		-0.0142	0.0157	0.0032	0.4484		45 / 45
25000		0.0347	0.0060	-0.0351	0.2567		40 / 40
25001		0.0234	0.0049	-0.0030	0.3189		40 / 40
210		0.0159	-0.0016	0.0065	0.3019		43 / 43
25003		0.0195	-0.0124	-0.0350	0.3962		40 / 40

25004		0.0215	0.0089	-0.0625	0.3566	33 / 33
25005		0.0187	-0.0039	-0.0396	0.3171	39 / 39
25006		-0.0211	-0.0383	-0.0731	0.4977	38 / 38
25008		-0.0321	-0.0001	-0.0605	0.3602	38 / 38
25009		0.0274	-0.0499	-0.0659	0.3760	42 / 42
25010		0.0080	-0.0158	-0.0637	0.2887	41 / 41
25013		-0.0055	0.0155	-0.0360	0.4100	38 / 38
25014		-0.0138	-0.0079	-0.0489	0.3102	37 / 37
25015		-0.0273	-0.0099	-0.0430	0.4439	41 / 41
25016		-0.0280	-0.0088	-0.0393	0.2904	42 / 42
25017		0.0219	-0.0178	0.0401	0.4457	37 / 37
25018		0.0069	-0.0005	0.0273	0.3985	38 / 38
25019		-0.0141	-0.0104	0.0169	0.4797	39 / 39
25020		-0.0105	0.0056	-0.0124	0.3758	33 / 33
25021		0.0198	0.0235	0.0030	0.3727	36 / 36
25022		0.0170	0.0047	0.0171	0.4796	34 / 34
25023		-0.0014	0.0226	0.0034	0.4267	40 / 40
25024		0.0056	-0.0195	-0.0027	0.3517	40 / 40
25029		0.0096	-0.0077	0.0057	0.3449	42 / 42
25030		-0.0106	-0.0111	0.0101	0.3995	44 / 44
25032		-0.0746	-0.0264	-0.0305	0.4288	36 / 36
25033		-0.0231	-0.0210	-0.0001	0.3797	41 / 41
25034		-0.0162	-0.0204	-0.0056	0.4165	36 / 36
40000		0.0242	-0.0230	-0.1079	0.3125	42 / 42
40001		0.0354	0.0108	-0.0198	0.2946	43 / 43
40002		0.0267	0.0328	-0.0309	0.3872	42 / 42
40003		0.0190	0.0383	-0.0028	0.3924	39 / 39
40004		0.0150	0.0003	-0.0106	0.3081	37 / 37
40005		0.0257	0.0187	-0.0281	0.2942	29 / 29
45000		0.0315	-0.0283	0.0035	0.4241	44 / 44
45001		-0.0064	0.0035	0.0014	0.4210	34 / 34
45002		0.0110	-0.0337	0.0027	0.5050	41 / 41
10043		-0.0023	-0.0003	-0.0120	0.6832	35 / 35
10084		-0.0013	-0.0017	0.0378	0.5694	30 / 30
20015		-0.0016	-0.0180	-0.0396	0.3392	45 / 45
20007		0.0013	-0.0110	-0.0080	0.3937	24 / 24
25027		-0.0045	-0.0160	-0.0368	0.3259	43 / 43
k9		-0.0092	-0.0188	-0.0258	0.6130	42 / 42
205		-0.0139	-0.0352	0.0023	0.8646	40 / 40
6600		0.0076	-0.0265	-0.0369	0.4281	41 / 41
6602		0.0053	0.0218	-0.0248	0.3953	37 / 37
6603		0.0000	-0.0257	-0.0017	0.4457	40 / 40
6605		0.0126	0.0134	0.0106	0.5000	40 / 40
6606		0.0178	0.0266	0.0084	0.5781	25 / 25
6607		0.0029	0.0038	-0.0348	0.7326	35 / 35
6608		-0.0070	-0.0218	0.0057	0.6827	21 / 21
6609		-0.0178	-0.0026	0.0013	0.3967	42 / 42
6610		-0.0006	-0.0022	0.0230	0.7529	33 / 33
6611		-0.0216	-0.0286	-0.0615	0.5784	40 / 40
6612		-0.0136	-0.0592	-0.0707	0.7526	38 / 38
6616		0.0019	-0.0340	-0.0701	0.5162	33 / 33
6617		-0.0100	-0.0042	0.0153	0.5947	23 / 23
6618		-0.0091	-0.0033	-0.0175	0.5162	40 / 40
6619		0.0177	0.0001	0.0098	0.2799	41 / 41
6620		0.0153	-0.0465	-0.0091	0.3983	38 / 38
6621		-0.0235	-0.0108	0.0088	0.2363	41 / 41
6622		-0.0067	0.0038	-0.0138	0.6409	37 / 37
6623		-0.0107	-0.0212	-0.0257	0.7974	40 / 40
6624		0.0458	-0.0098	-0.0134	0.7532	33 / 33

6625		-0.0541	-0.0311	-0.0296	0.6020	37 / 37
6626		-0.0237	-0.0141	-0.0623	0.4158	35 / 35
6627		-0.0331	-0.0292	-0.0282	0.6194	33 / 33
6628		-0.0391	-0.0367	-0.0506	0.5337	39 / 39
6629		-0.0517	-0.0334	-0.0483	0.4865	37 / 37
6630		-0.0608	-0.0178	-0.0384	0.4318	34 / 34
6631		-0.0348	-0.0347	-0.0418	0.6129	32 / 32
6632		-0.0491	-0.0259	-0.0229	0.6296	34 / 34
6633		-0.0117	-0.0236	-0.0341	0.4472	38 / 38
6634		-0.0181	-0.0037	-0.0316	0.4269	35 / 35
6635		-0.0056	-0.0019	-0.0317	0.4916	39 / 39
6636		-0.0486	-0.0359	-0.0440	0.5583	41 / 41
6637		-0.0102	-0.0142	-0.0251	0.6441	31 / 31
6638		-0.0289	-0.0143	-0.0313	0.4655	27 / 27
6639		-0.0552	-0.0081	-0.0385	0.5737	38 / 38
6640		-0.0406	-0.0258	-0.0433	0.5207	34 / 34
6641		-0.0491	-0.0197	-0.0419	0.5790	37 / 37
**Mean [m]**		0.001043	-0.004590	-0.011186		
**Sigma [m]**		0.025189	0.024447	0.038668		
**RMS Error [m]**		0.025210	0.024874	0.040253		

Localisation accuracy per GCP and mean errors in the three coordinate directions. The last column counts the number of calibrated images where the GCP has been automatically verified vs. manually marked.

Absolute Geolocation Variance

Min Error [m]	Max Error [m]	Geolocation Error X [%]	Geolocation Error Y [%]	Geolocation Error Z [%]
-	-15.00	0.00	0.23	0.00
-15.00	-12.00	0.00	0.00	0.00
-12.00	-9.00	0.00	0.00	0.00
-9.00	-6.00	0.00	0.00	0.00
-6.00	-3.00	0.23	5.36	7.69
-3.00	0.00	47.20	47.20	39.98
0.00	3.00	52.45	38.00	45.57
3.00	6.00	0.12	8.97	6.76
6.00	9.00	0.00	0.00	0.00
9.00	12.00	0.00	0.00	0.00
12.00	15.00	0.00	0.00	0.00
15.00	-	0.00	0.23	0.00
**Mean [m]**		0.867064	1.675303	32.874461
**Sigma [m]**		1.114671	2.670310	2.137511
**RMS Error [m]**		1.412194	3.152332	32.943879

Min Error and Max Error represent geolocation error intervals between -1.5 and 1.5 times the maximum accuracy of all the images. Columns X, Y, Z show the percentage of images with geolocation errors within the predefined error intervals. The geolocation error is the difference between the initial and computed image positions. Note that the image geolocation errors do not correspond to the accuracy of the observed 3D points.

Geolocation Bias	X	Y	Z
Translation [m]	0.867069	1.675297	32.874462

Bias between image initial and computed geolocation given in output coordinate system.

Relative Geolocation Variance

Relative Geolocation Error	Images X [%]	Images Y [%]	Images Z [%]
[-1.00, 1.00]	100.00	98.72	100.00
[-2.00, 2.00]	100.00	99.53	100.00

[-3.00, 3.00]	100.00	99.53	100.00
**Mean of Geolocation Accuracy [m]**	5.000000	5.000000	10.000000
**Sigma of Geolocation Accuracy [m]**	0.000000	0.000000	0.000000

Images X, Y, Z represent the percentage of images with a relative geolocation error in X, Y, Z.

Initial Processing Details

System Information

Hardware	CPU: Intel(R) Core(TM) i9-7940X CPU @ 3.10GHz<br>RAM: 128GB<br>GPU: NVIDIA GeForce GTX 1080 Ti (Driver: 22.21.13.8541), RDPDD Chained DD (Driver: unknown), RDP Encoder Mirror Driver (Driver: unknown), RDP Reflector Display Driver (Driver: unknown)
Operating System	Windows 7 Ultimate, 64-bit

Coordinate Systems

Image Coordinate System	WGS 84 (EGM 96 Geoid)
Ground Control Point (GCP) Coordinate System	ETRS89 / Poland CS2000 zone 6 (EGM 96 Geoid)
Output Coordinate System	ETRS89 / Poland CS2000 zone 6 (EGM 96 Geoid)

Processing Options

Detected Template	No Template Available
Keypoints Image Scale	Full, Image Scale: 1
Advanced: Matching Image Pairs	Aerial Grid or Corridor
Advanced: Matching Strategy	Use Geometrically Verified Matching: yes
Advanced: Keypoint Extraction	Targeted Number of Keypoints: Automatic
Advanced: Calibration	Calibration Method: Standard<br>Internal Parameters Optimization: All<br>External Parameters Optimization: All<br>Rematch: Auto, no
