# Supplementary material for: Multi-Variant Accuracy Evaluation of UAV Imaging Surveys: A Case Study on Investment Area
Source: Sensors (Basel). 2019 Nov 28;19(23):5229. doi: 10.3390/s19235229 (PMC6929115; doi:10.3390/s19235229)
Supplement: Supplementary file 1 [file sensors-19-05229-s001.zip › supplementary_files/App4_V4_report.pdf]

Quality Report

Generated with Pix4Ddiscovery version 4.3.31

Important: Click on the different icons for:

Help to analyze the results in the Quality Report

Additional information about the sections

Click here for additional tips to analyze the Quality Report

Summary

Project	Sosnowiec_2_new_ver_8GCP
Processed	2019-02-22 14:21:26
Camera Model Name(s)	NEX-5T_0.0_4912x3264 (RGB)
Average Ground Sampling Distance (GSD)	4.59 cm / 1.81 in
Area Covered	1.029 km^2 / 102.8855 ha / 0.40 sq. mi. / 254.3672 acres
Time for Initial Processing (without report)	42m:52s

Quality Check

**Images**	median of 45097 keypoints per image	✓
**Dataset**	858 out of 858 images calibrated (100%), all images enabled	✓
**Camera Optimization**	0.19% relative difference between initial and optimized internal camera parameters	✓
**Matching**	median of 14882.3 matches per calibrated image	✓
**Georeferencing**	yes, 8 GCPs (8 3D), mean RMS error = 0.019 m	✓

Preview

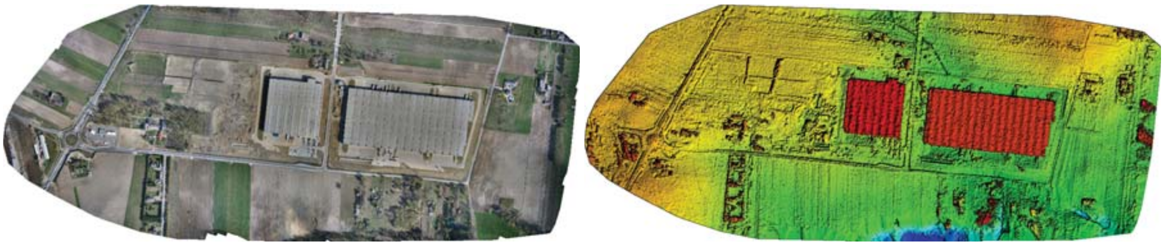

Figure 1: Orthomosaic and the corresponding sparse Digital Surface Model (DSM) before densification.

Calibration Details

Number of Calibrated Images	858 out of 858
Number of Geolocated Images	858 out of 858

Initial Image Positions

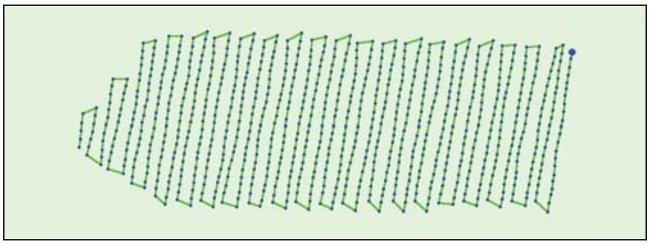

Figure 2: Top view of the initial image position. The green line follows the position of the images in time starting from the large blue dot.

Computed Image/GCPs/Manual Tie Points Positions

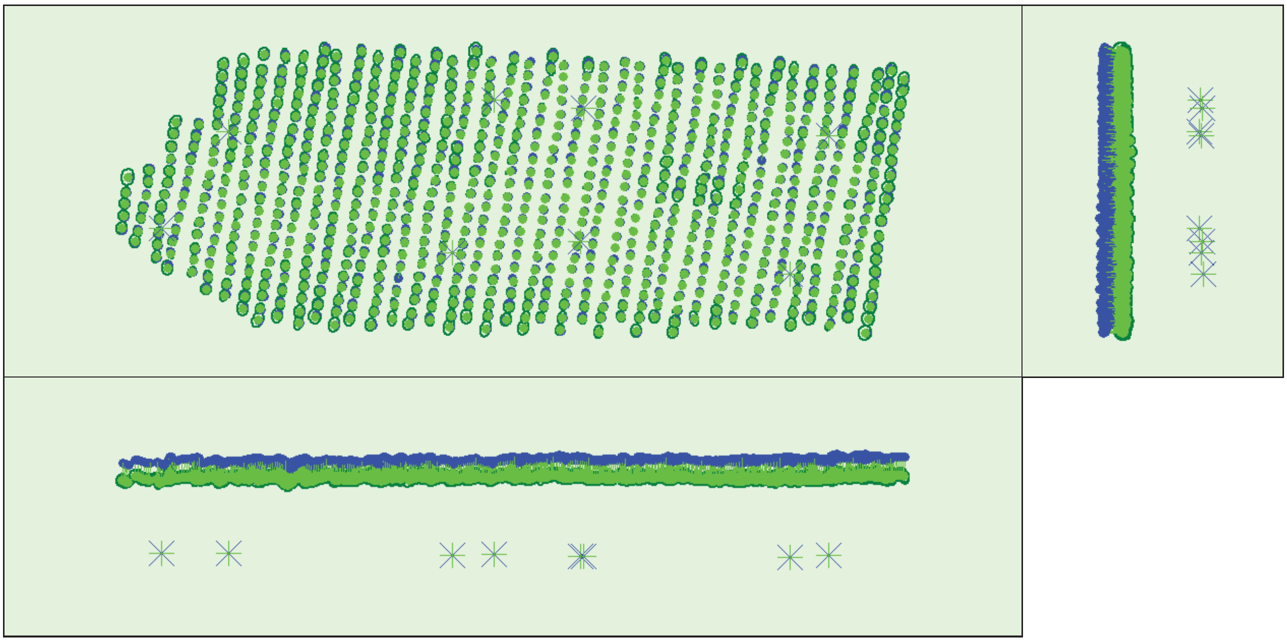

Uncertainty ellipses 500x magnified

Figure 3: Offset between initial (blue dots) and computed (green dots) image positions as well as the offset between the GCPs initial positions (blue crosses) and their computed positions (green crosses) in the top-view (XY plane), front-view (XZ plane), and side-view (YZ plane). Dark green ellipses indicate the absolute position uncertainty of the bundle block adjustment result.

Absolute camera position and orientation uncertainties

	X[m]	Y[m]	Z[m]	Omega [degree]	Phi [degree]	Kappa [degree]
Mean	0.014	0.018	0.023	0.006	0.005	0.002
Sigma	0.004	0.004	0.001	0.002	0.001	0.001

Overlap

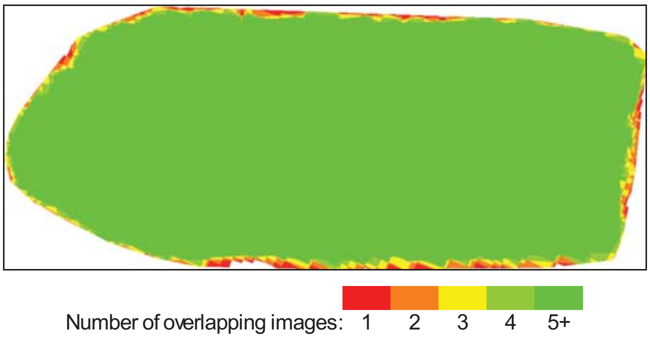

Figure 4: Number of overlapping images computed for each pixel of the orthomosaic.
Red and yellow areas indicate low overlap for which poor results may be generated. Green areas indicate an overlap of over 5 images for every pixel. Good quality results will be generated as long as the number of keypoint matches is also sufficient for these areas (see Figure 5 for keypoint matches).

Bundle Block Adjustment Details

Number of 2D Keypoint Observations for Bundle Block Adjustment	12681582
Number of 3D Points for Bundle Block Adjustment	3199085
Mean Reprojection Error [pixels]	0.188

Internal Camera Parameters

NEX-5T_0.0_4912x3264 (RGB). Sensor Dimensions: 23.400 [mm] x 15.549 [mm]

EXIF ID: NEX-5T_0.0_4912x3264

	Focal Length	Principal Point x	Principal Point y	R1	R2	R3	T1	T2
Initial Values	3256.554 [pixel]<br>15.514 [mm]	2456.002 [pixel]<br>11.700 [mm]	1632.003 [pixel]<br>7.775 [mm]	-0.043	0.028	-0.005	0.000	-0.001
Optimized Values	3250.195 [pixel]<br>15.483 [mm]	2467.000 [pixel]<br>11.752 [mm]	1589.780 [pixel]<br>7.573 [mm]	-0.047	0.035	-0.009	0.000	0.000
Uncertainties (Sigma)	0.478 [pixel]<br>0.002 [mm]	0.067 [pixel]<br>0.000 [mm]	0.071 [pixel]<br>0.000 [mm]	0.000	0.000	0.000	0.000	0.000

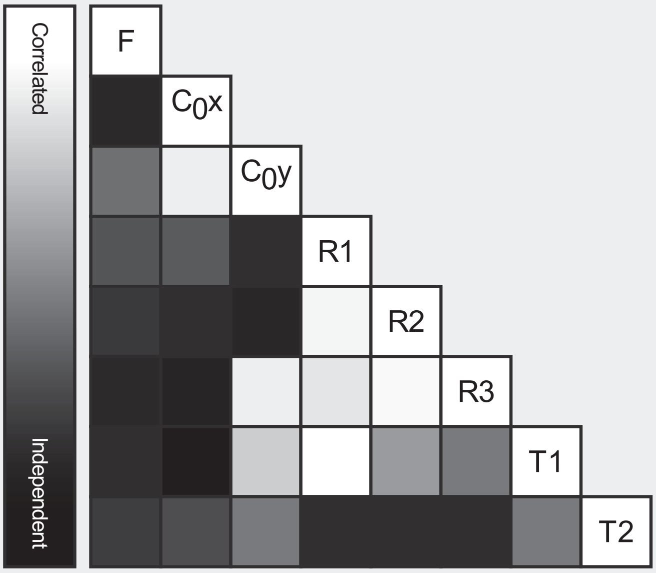

The correlation between camera internal parameters determined by the bundle adjustment. White indicates a full correlation between the parameters, ie. any change in one can be fully compensated by the other. Black indicates that the parameter is completely independent, and is not affected by other parameters.

The number of Automatic Tie Points (ATPs) per pixel, averaged over all images of the camera model, is color coded between black and white. White indicates that, on average, more than 16 ATPs have been extracted at the pixel location. Black indicates that, on average, 0 ATPs have been extracted at the pixel location. Click on the image to the see the average direction and magnitude of the re-projection error for each pixel. Note that the vectors are scaled for better visualization. The scale bar indicates the magnitude of 1 pixel error.

2D Keypoints Table

	Number of 2D Keypoints per Image	Number of Matched 2D Keypoints per Image
Median	45097	14882
Min	20000	2357
Max	68781	30864
Mean	42501	14780

3D Points from 2D Keypoint Matches

	Number of 3D Points Observed
In 2 Images	1628444

In 3 Images	591697
In 4 Images	295649
In 5 Images	174244
In 6 Images	113054
In 7 Images	77893
In 8 Images	57422
In 9 Images	43482
In 10 Images	34048
In 11 Images	27168
In 12 Images	22265
In 13 Images	18279
In 14 Images	15213
In 15 Images	12961
In 16 Images	10967
In 17 Images	9492
In 18 Images	8330
In 19 Images	7225
In 20 Images	6231
In 21 Images	5520
In 22 Images	4844
In 23 Images	4442
In 24 Images	3906
In 25 Images	3360
In 26 Images	3090
In 27 Images	2656
In 28 Images	2260
In 29 Images	2124
In 30 Images	1898
In 31 Images	1630
In 32 Images	1530
In 33 Images	1312
In 34 Images	1084
In 35 Images	968
In 36 Images	877
In 37 Images	752
In 38 Images	614
In 39 Images	555
In 40 Images	423
In 41 Images	367
In 42 Images	286
In 43 Images	206
In 44 Images	133
In 45 Images	95
In 46 Images	47
In 47 Images	22
In 48 Images	11
In 49 Images	5
In 50 Images	3
In 51 Images	1

2D Keypoint Matches

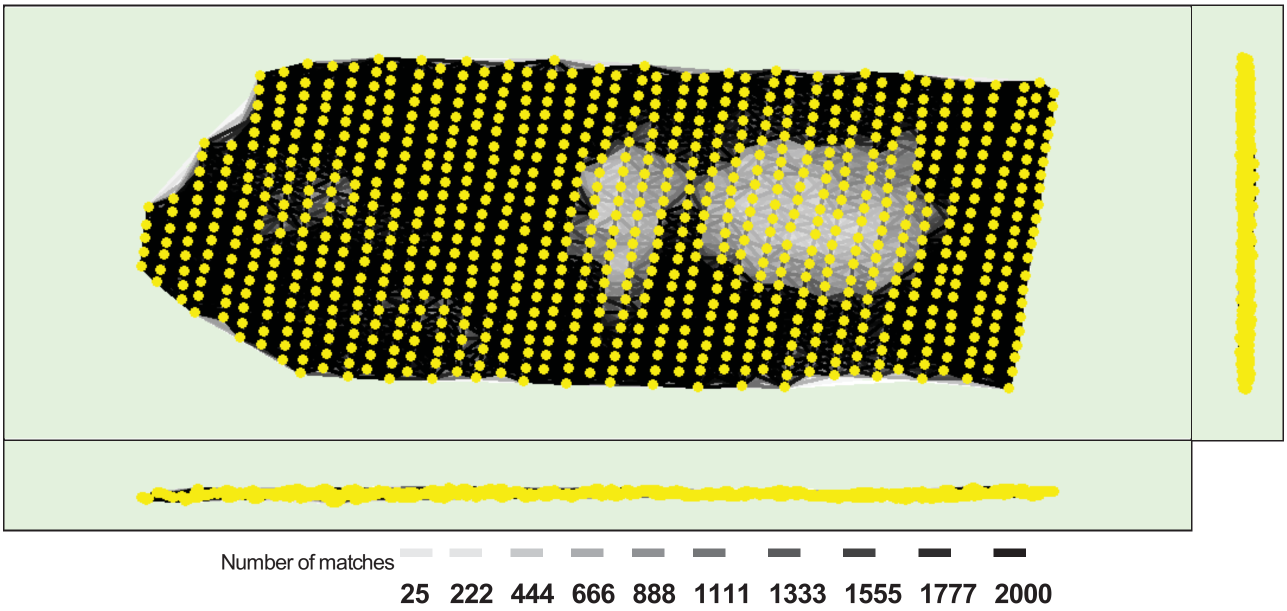

Figure 5: Computed image positions with links between matched images. The darkness of the links indicates the number of matched 2D keypoints between the images. Bright links indicate weak links and require manual tie points or more images.

Geolocation Details

Ground Control Points

GCP Name	Accuracy XY/Z [m]	Error X [m]	Error Y [m]	Error Z [m]	Projection Error [pixel]	Verified/Marked
201 (3D)	0.030/ 0.050	-0.005	-0.025	0.004	0.747	34 / 34
202 (3D)	0.030/ 0.050	0.002	0.019	0.002	0.697	31 / 31
203 (3D)	0.030/ 0.050	-0.006	0.024	-0.009	0.744	39 / 39
208 (3D)	0.030/ 0.050	-0.009	-0.014	0.035	0.617	38 / 38
209 (3D)	0.030/ 0.050	0.014	-0.019	-0.030	0.459	37 / 37
211 (3D)	0.030/ 0.050	0.011	0.029	0.012	0.509	44 / 44
205 (3D)	0.030/ 0.050	-0.013	-0.032	0.029	0.857	40 / 40
204 (3D)	0.030/ 0.050	0.004	0.017	-0.044	0.872	38 / 39
**Mean [m]**		0.000027	-0.000021	0.000016		
**Sigma [m]**		0.008977	0.023006	0.025311		
**RMS Error [m]**		0.008977	0.023006	0.025311		

0 out of 210 check points have been labeled as inaccurate.

Check Point Name	Accuracy XY/Z [m]	Error X [m]	Error Y [m]	Error Z [m]	Projection Error [pixel]	Verified/Marked
103		0.0079	0.0189	0.0302	0.4334	42 / 42
101		-0.0093	0.0142	0.0357	0.4056	38 / 38
100		-0.0089	-0.0277	0.0489	0.3572	36 / 36
104		-0.0387	-0.0079	0.0124	0.5068	39 / 39
102		-0.0163	-0.0154	0.0315	0.3556	43 / 43
k1		0.0302	-0.0477	0.1703	0.5051	38 / 38
k2		0.0002	0.0285	0.0492	0.4510	43 / 43
k3		0.0870	-0.0706	0.2185	0.6133	26 / 26
k4		0.0211	-0.0194	0.1190	0.4982	36 / 36
k5		-0.0252	0.0360	0.0232	0.4267	38 / 38
k6		-0.0308	0.0131	0.1040	0.7260	29 / 29
k7		-0.0810	0.0059	0.1955	0.5405	28 / 28
k8		-0.0425	-0.0299	0.1645	0.5802	30 / 30
k11		-0.0131	-0.0185	-0.0245	0.5108	43 / 43
k13		-0.0032	0.0110	0.0369	0.3988	29 / 29
k14		-0.0003	0.0102	0.0374	0.4787	38 / 38

10000		-0.0299	-0.0054	0.0676	0.5846	32 / 32
10001		0.0005	0.0021	0.0847	0.5753	35 / 35
10002		0.0065	-0.0290	0.0218	0.4532	37 / 37
10003		0.0021	-0.0394	0.0047	0.5173	40 / 40
10004		0.0172	-0.0134	0.0520	0.5025	39 / 39
10005		0.0234	-0.0202	0.0462	0.5580	43 / 43
10006		-0.0014	-0.0217	0.0358	0.3988	42 / 42
10007		0.0047	-0.0184	0.0208	0.3865	41 / 41
10008		0.0138	-0.0283	0.0151	0.3576	38 / 38
10009		0.0067	-0.0258	0.0278	0.4691	42 / 42
10010		0.0056	-0.0265	0.0168	0.4625	44 / 44
10011		0.0078	-0.0371	0.0131	0.5428	33 / 33
10012		-0.0361	0.0091	0.0337	0.5535	40 / 40
10013		-0.0210	0.0024	0.0302	0.4265	38 / 38
10014		0.0033	0.0130	0.0403	0.3772	42 / 42
10015		0.0130	0.0054	0.0556	0.4299	40 / 40
10016		-0.0256	0.0012	0.0453	0.5104	43 / 43
10017		0.0016	-0.0112	-0.0118	0.4221	42 / 42
10018		0.0014	-0.0008	0.0033	0.3695	22 / 22
10019		-0.0112	0.0191	0.0772	0.6164	42 / 42
10020		-0.0252	0.0134	0.0498	0.4556	33 / 33
10021		-0.0038	0.0022	0.0753	0.4142	45 / 45
10022		0.0152	0.0318	0.0493	0.5284	42 / 42
10023		0.0199	-0.0041	0.0381	0.4186	38 / 38
10024		-0.0117	0.0022	0.0112	0.3919	46 / 46
10025		0.0042	0.0023	0.0374	0.3842	44 / 44
10026		-0.0120	0.0253	0.0374	0.4547	36 / 36
10027		-0.0164	0.0199	0.0356	0.5052	41 / 41
10028		-0.0042	0.0056	0.0481	0.4245	43 / 43
10030		-0.0118	0.0330	0.0257	0.3873	37 / 37
10031		0.0292	0.0288	0.0440	0.5315	27 / 27
10032		0.0361	0.0374	0.0658	0.4585	27 / 27
10033		0.0007	0.0308	0.0633	0.3799	40 / 40
10034		0.0243	-0.0092	0.0513	0.4012	38 / 38
10035		0.0108	-0.0269	0.1057	0.3989	35 / 35
10036		0.0126	-0.0068	0.0550	0.5559	27 / 27
10037		-0.0032	-0.0110	0.0357	0.3916	39 / 39
10038		-0.0063	0.0076	0.0324	0.3804	42 / 42
10039		0.0035	-0.0058	0.0237	0.4155	43 / 43
10040		-0.0236	-0.0087	0.0191	0.4853	31 / 31
10041		-0.0155	-0.0230	0.0160	0.5556	35 / 35
10044		0.0101	-0.0444	-0.0026	0.4956	38 / 38
10045		0.0177	-0.0384	-0.0282	0.3838	36 / 36
10046		-0.0150	-0.0092	-0.0453	0.3799	38 / 38
10047		-0.0306	-0.0055	0.1087	0.7253	31 / 31
10048		-0.0169	-0.0220	0.0816	0.6418	32 / 32
10049		-0.0246	0.0287	0.0826	0.8368	32 / 32
207		-0.0231	-0.0151	0.1111	0.4728	35 / 35
10051		-0.0038	0.0203	0.0542	0.3852	44 / 44
10052		-0.0058	0.0064	0.0592	0.5289	35 / 35
10053		-0.0152	0.0155	0.0349	0.4073	40 / 40
10054		-0.0136	-0.0147	0.0186	0.4256	38 / 38
10055		-0.0025	0.0152	0.0146	0.5697	39 / 39
10056		-0.0394	0.0148	0.0286	0.5265	38 / 38
10058		-0.0724	0.0902	0.0616	0.5362	44 / 44
10059		-0.0117	-0.0232	-0.0180	0.3637	40 / 40
10060		-0.0307	-0.0222	-0.0025	0.3562	43 / 43
10061		0.0042	0.0280	0.0614	0.4851	29 / 29
10062		-0.0052	-0.0222	0.0138	0.4729	41 / 41

10063		0.0144	-0.0431	-0.0146	0.4781	38 / 38
10064		0.0303	0.0206	0.0053	0.4415	43 / 43
10065		0.0216	0.0277	0.0256	0.3664	44 / 44
10066		0.0489	0.0307	0.0126	0.4349	41 / 41
10067		0.0236	0.0320	0.0238	0.3618	42 / 42
10068		0.0055	0.1025	0.0153	0.4498	43 / 43
10074		-0.0274	0.0476	0.0030	0.6876	41 / 41
10075		-0.0222	0.0451	0.0494	0.3195	41 / 41
10076		-0.0025	0.0225	0.0339	0.3542	40 / 40
10077		-0.0255	0.0554	0.0207	0.7336	38 / 38
10078		0.0186	0.0131	0.0214	0.3331	39 / 39
10080		0.0153	-0.0613	-0.0598	0.6884	35 / 35
10081		-0.0233	-0.0245	0.0322	0.3513	37 / 37
10082		0.0103	-0.0373	0.0012	0.4179	39 / 39
10083		0.0472	-0.0140	-0.0285	0.5939	37 / 37
10085		0.0296	-0.0311	0.0249	0.3383	44 / 44
15000		0.0053	-0.0395	-0.0080	0.3470	46 / 46
15001		-0.0279	-0.0423	-0.0131	0.3668	39 / 39
15002		0.0054	0.0176	-0.0419	0.4704	41 / 41
15003		-0.0078	0.0363	0.0199	0.4719	45 / 45
15004		-0.0076	0.0154	0.0081	0.3686	43 / 43
15005		-0.0067	0.0232	0.0086	0.5341	38 / 38
15006		0.0044	0.0352	-0.0041	0.4414	43 / 43
15007		0.0092	-0.0017	0.0394	0.4780	38 / 38
15008		-0.0231	0.0199	0.0770	0.7866	38 / 38
15009		-0.0108	0.0133	0.0710	0.5510	40 / 40
15010		0.0050	0.0390	0.0556	0.6377	38 / 38
20000		-0.0160	0.0207	0.0410	0.7517	42 / 42
20001		-0.0112	0.0097	0.0637	0.5120	38 / 38
20002		0.0006	0.0240	0.0649	0.4326	38 / 38
20003		-0.0240	-0.0046	0.0569	0.4247	39 / 39
20004		-0.0032	-0.0101	0.0450	0.4034	36 / 36
20005		-0.0498	-0.0250	0.0253	0.5286	35 / 35
20008		-0.0060	0.0023	0.0253	0.3933	44 / 44
20009		0.0090	-0.0042	0.0034	0.3870	43 / 43
20010		-0.0269	0.0263	0.0221	0.4080	45 / 45
20011		-0.0212	0.0012	0.0397	0.3790	47 / 47
20012		0.0040	0.0015	0.0282	0.3497	44 / 44
20013		0.0062	0.0009	0.0452	0.3571	41 / 41
20014		-0.0046	0.0093	0.0338	0.3552	43 / 43
20016		-0.0030	0.0178	-0.0087	0.3263	46 / 46
20017		0.0077	0.0273	0.0084	0.3643	40 / 40
20018		0.0184	0.0241	0.0216	0.3295	44 / 44
20019		0.0230	0.0156	0.0366	0.4142	39 / 39
20020		0.0038	0.0389	0.0367	0.4326	40 / 40
20021		0.0031	0.0004	0.0576	0.2622	40 / 40
20022		-0.0203	-0.0515	0.0557	0.3091	42 / 42
20023		-0.0031	0.0028	0.0396	0.3416	45 / 45
20024		-0.0071	0.0396	0.0104	0.4330	41 / 41
20025		0.0087	0.0474	0.0193	0.3515	42 / 42
20026		0.0083	0.0373	0.0213	0.4053	43 / 43
20027		0.0030	0.0716	0.0187	0.3874	40 / 40
20028		-0.0073	0.0117	0.0331	0.4078	41 / 41
20029		0.0128	-0.0161	0.0180	0.7744	41 / 41
20031		-0.0132	0.0255	0.0270	0.4406	45 / 45
25000		0.0141	0.0104	0.0148	0.2533	40 / 40
25001		0.0026	0.0075	0.0459	0.3054	40 / 40
210		-0.0022	-0.0022	0.0703	0.2659	43 / 43
25003		0.0036	-0.0180	0.0344	0.3707	40 / 40

25004		0.0050	0.0021	0.0064	0.3427	33 / 33
25005		0.0063	-0.0099	0.0285	0.3008	39 / 39
25006		-0.0321	-0.0421	-0.0117	0.4641	38 / 38
25008		-0.0321	0.0002	-0.0016	0.2307	38 / 38
25009		0.0271	-0.0496	-0.0100	0.2575	42 / 42
25010		0.0045	-0.0176	-0.0152	0.2617	41 / 41
25013		-0.0043	0.0178	-0.0042	0.3988	38 / 38
25014		-0.0126	-0.0057	-0.0170	0.3040	37 / 37
25015		-0.0261	-0.0076	-0.0113	0.4329	41 / 41
25016		-0.0268	-0.0065	-0.0076	0.2831	42 / 42
25017		0.0215	-0.0153	0.0732	0.4320	37 / 37
25018		0.0056	0.0021	0.0623	0.3963	38 / 38
25019		-0.0156	-0.0080	0.0524	0.4789	39 / 39
25020		-0.0113	0.0086	0.0211	0.3721	33 / 33
25021		0.0153	0.0275	0.0459	0.3651	36 / 36
25022		0.0128	0.0086	0.0599	0.4856	34 / 34
25023		-0.0069	0.0269	0.0449	0.4252	40 / 40
25024		-0.0020	-0.0113	0.0341	0.3487	40 / 40
25029		0.0090	-0.0024	0.0178	0.3338	42 / 42
25030		-0.0104	-0.0071	0.0337	0.3977	44 / 44
25032		-0.0747	-0.0225	-0.0071	0.4239	36 / 36
25033		-0.0239	-0.0170	0.0254	0.3756	41 / 41
25034		-0.0179	-0.0163	0.0233	0.4029	36 / 36
206		0.0021	0.0317	0.0527	0.4057	35 / 35
40000		0.0049	-0.0171	-0.0635	0.3011	42 / 42
40001		0.0179	0.0181	0.0233	0.2916	43 / 43
40002		0.0136	0.0408	0.0077	0.3882	42 / 42
40003		0.0061	0.0451	0.0372	0.3921	39 / 39
40004		0.0013	0.0059	0.0318	0.2987	37 / 37
40005		0.0148	0.0258	0.0106	0.2855	29 / 29
45000		0.0325	-0.0260	0.0354	0.4157	44 / 44
45001		-0.0062	0.0062	0.0333	0.4077	34 / 34
45002		0.0111	-0.0298	0.0262	0.4964	41 / 41
10043		-0.0147	-0.0065	0.0563	0.6464	35 / 35
10084		-0.0116	-0.0034	0.1000	0.5580	30 / 30
20015		-0.0035	-0.0077	-0.0123	0.3291	45 / 45
20007		-0.0046	-0.0064	0.0333	0.3937	24 / 24
25027		-0.0041	-0.0066	-0.0102	0.3164	43 / 43
k9		-0.0064	-0.0114	-0.0239	0.5525	42 / 42
6600		-0.0050	-0.0182	0.0010	0.4093	41 / 41
6602		-0.0026	0.0305	0.0106	0.3825	37 / 37
6603		-0.0077	-0.0182	0.0381	0.4392	40 / 40
6605		0.0074	0.0192	0.0505	0.4936	40 / 40
6606		0.0134	0.0314	0.0480	0.5742	25 / 25
6607		-0.0030	0.0133	-0.0051	0.7231	35 / 35
6608		-0.0100	-0.0144	0.0402	0.6752	21 / 21
6609		-0.0187	0.0068	0.0306	0.3847	42 / 42
6610		0.0001	0.0064	0.0481	0.7426	33 / 33
6611		-0.0146	-0.0223	-0.0480	0.5636	40 / 40
6612		-0.0069	-0.0530	-0.0575	0.7124	38 / 38
6616		-0.0069	-0.0366	-0.0148	0.4978	33 / 33
6617		-0.0090	-0.0025	0.0492	0.5936	23 / 23
6618		-0.0086	-0.0003	0.0131	0.5131	40 / 40
6619		0.0183	0.0033	0.0392	0.2791	41 / 41
6620		0.0160	-0.0426	0.0164	0.4069	38 / 38
6621		-0.0241	-0.0059	0.0247	0.2319	41 / 41
6622		0.0044	0.0108	-0.0092	0.5950	37 / 37
6623		0.0008	-0.0124	-0.0270	0.7789	40 / 40
6624		0.0516	-0.0014	-0.0101	0.7113	33 / 33

6625		-0.0499	-0.0248	-0.0251	0.5843	37 / 37
6626		-0.0190	-0.0078	-0.0585	0.4226	35 / 35
6627		-0.0291	-0.0233	-0.0229	0.5998	33 / 33
6628		-0.0351	-0.0310	-0.0455	0.5069	39 / 39
6629		-0.0484	-0.0275	-0.0431	0.4748	37 / 37
6630		-0.0573	-0.0118	-0.0329	0.4030	34 / 34
6631		-0.0317	-0.0284	-0.0371	0.5763	32 / 32
6632		-0.0466	-0.0194	-0.0173	0.6042	34 / 34
6633		-0.0093	-0.0181	-0.0297	0.4360	38 / 38
6634		-0.0156	0.0021	-0.0275	0.4084	35 / 35
6635		-0.0029	0.0036	-0.0267	0.4710	39 / 39
6636		-0.0457	-0.0300	-0.0391	0.5373	41 / 41
6637		-0.0071	-0.0084	-0.0205	0.6340	31 / 31
6638		-0.0258	-0.0084	-0.0265	0.4519	27 / 27
6639		-0.0522	-0.0022	-0.0339	0.5539	38 / 38
6640		-0.0379	-0.0202	-0.0384	0.5107	34 / 34
6641		-0.0468	-0.0143	-0.0370	0.5692	37 / 37
**Mean [m]**		-0.005136	0.000023	0.025612		
**Sigma [m]**		0.021867	0.025739	0.041247		
**RMS Error [m]**		0.022462	0.025739	0.048552		

Localisation accuracy per GCP and mean errors in the three coordinate directions. The last column counts the number of calibrated images where the GCP has been automatically verified vs. manually marked.

Absolute Geolocation Variance

Min Error [m]	Max Error [m]	Geolocation Error X [%]	Geolocation Error Y [%]	Geolocation Error Z [%]
-	-15.00	0.00	0.23	0.00
-15.00	-12.00	0.00	0.00	0.00
-12.00	-9.00	0.00	0.00	0.00
-9.00	-6.00	0.00	0.00	0.00
-6.00	-3.00	0.23	5.36	8.04
-3.00	0.00	47.32	47.55	39.86
0.00	3.00	52.33	37.65	45.22
3.00	6.00	0.12	8.97	6.88
6.00	9.00	0.00	0.00	0.00
9.00	12.00	0.00	0.00	0.00
12.00	15.00	0.00	0.00	0.00
15.00	-	0.00	0.23	0.00
**Mean [m]**		0.857926	1.673402	32.663302
**Sigma [m]**		1.126015	2.666882	2.154561
**RMS Error [m]**		1.415608	3.148417	32.734285

Min Error and Max Error represent geolocation error intervals between -1.5 and 1.5 times the maximum accuracy of all the images. Columns X, Y, Z show the percentage of images with geolocation errors within the predefined error intervals. The geolocation error is the difference between the initial and computed image positions. Note that the image geolocation errors do not correspond to the accuracy of the observed 3D points.

Geolocation Bias	X	Y	Z
Translation [m]	0.857931	1.673396	32.663302

Bias between image initial and computed geolocation given in output coordinate system.

Relative Geolocation Variance

Relative Geolocation Error	Images X [%]	Images Y [%]	Images Z [%]
[-1.00, 1.00]	100.00	98.72	100.00
[-2.00, 2.00]	100.00	99.53	100.00

[-3.00, 3.00]	100.00	99.53	100.00
**Mean of Geolocation Accuracy [m]**	5.000000	5.000000	10.000000
**Sigma of Geolocation Accuracy [m]**	0.000000	0.000000	0.000000

Images X, Y, Z represent the percentage of images with a relative geolocation error in X, Y, Z.

Initial Processing Details

System Information

Hardware	CPU: Intel(R) Core(TM) i9-7940X CPU @ 3.10GHz<br>RAM: 128GB<br>GPU: NVIDIA GeForce GTX 1080 Ti (Driver: 22.21.13.8541), RDPDD Chained DD (Driver: unknown), RDP Encoder Mirror Driver (Driver: unknown), RDP Reflector Display Driver (Driver: unknown)
Operating System	Windows 7 Ultimate, 64-bit

Coordinate Systems

Image Coordinate System	WGS 84 (EGM 96 Geoid)
Ground Control Point (GCP) Coordinate System	ETRS89 / Poland CS2000 zone 6 (EGM 2008 Geoid)
Output Coordinate System	ETRS89 / Poland CS2000 zone 6 (EGM 2008 Geoid)

Processing Options

Detected Template	3D Maps
Keypoints Image Scale	Full, Image Scale: 1
Advanced: Matching Image Pairs	Aerial Grid or Corridor
Advanced: Matching Strategy	Use Geometrically Verified Matching: no
Advanced: Keypoint Extraction	Targeted Number of Keypoints: Automatic
Advanced: Calibration	Calibration Method: Standard<br>Internal Parameters Optimization: All<br>External Parameters Optimization: All<br>Rematch: Auto, no
