# Supplementary material for: Multi-Variant Accuracy Evaluation of UAV Imaging Surveys: A Case Study on Investment Area
Source: Sensors (Basel). 2019 Nov 28;19(23):5229. doi: 10.3390/s19235229 (PMC6929115; doi:10.3390/s19235229)
Supplement: Supplementary file 1 [file sensors-19-05229-s001.zip › supplementary_files/App5_V5_report.pdf]

Quality Report

Generated with Pix4Ddiscovery version 4.3.31

Important: Click on the different icons for:

Help to analyze the results in the Quality Report

Additional information about the sections

Click here for additional tips to analyze the Quality Report

Summary

Project	Sosnowiec_2_new_ver_10GCP
Processed	2019-02-22 11:22:23
Camera Model Name(s)	NEX-5T_0.0_4912x3264 (RGB)
Average Ground Sampling Distance (GSD)	4.59 cm / 1.81 in
Area Covered	1.029 km^2 / 102.8853 ha / 0.40 sq. mi. / 254.3666 acres
Time for Initial Processing (without report)	01h:26m:38s

Quality Check

**Images**	median of 45097 keypoints per image	✓
**Dataset**	858 out of 858 images calibrated (100%), all images enabled	✓
**Camera Optimization**	0.19% relative difference between initial and optimized internal camera parameters	✓
**Matching**	median of 14881.3 matches per calibrated image	✓
**Georeferencing**	yes, 10 GCPs (10 3D), mean RMS error = 0.021 m	✓

Preview

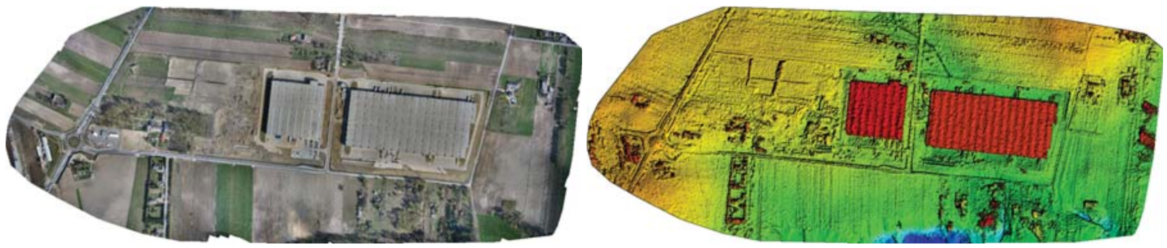

Figure 1: Orthomosaic and the corresponding sparse Digital Surface Model (DSM) before densification.

Calibration Details

Number of Calibrated Images	858 out of 858
Number of Geolocated Images	858 out of 858

Initial Image Positions

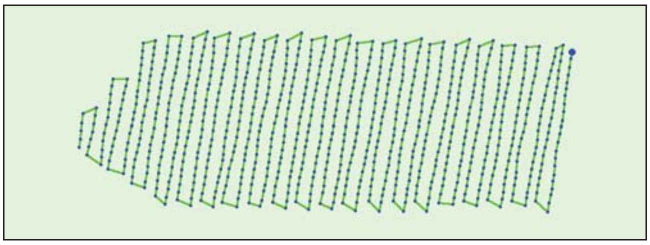

Figure 2: Top view of the initial image position. The green line follows the position of the images in time starting from the large blue dot.

Computed Image/GCPs/Manual Tie Points Positions

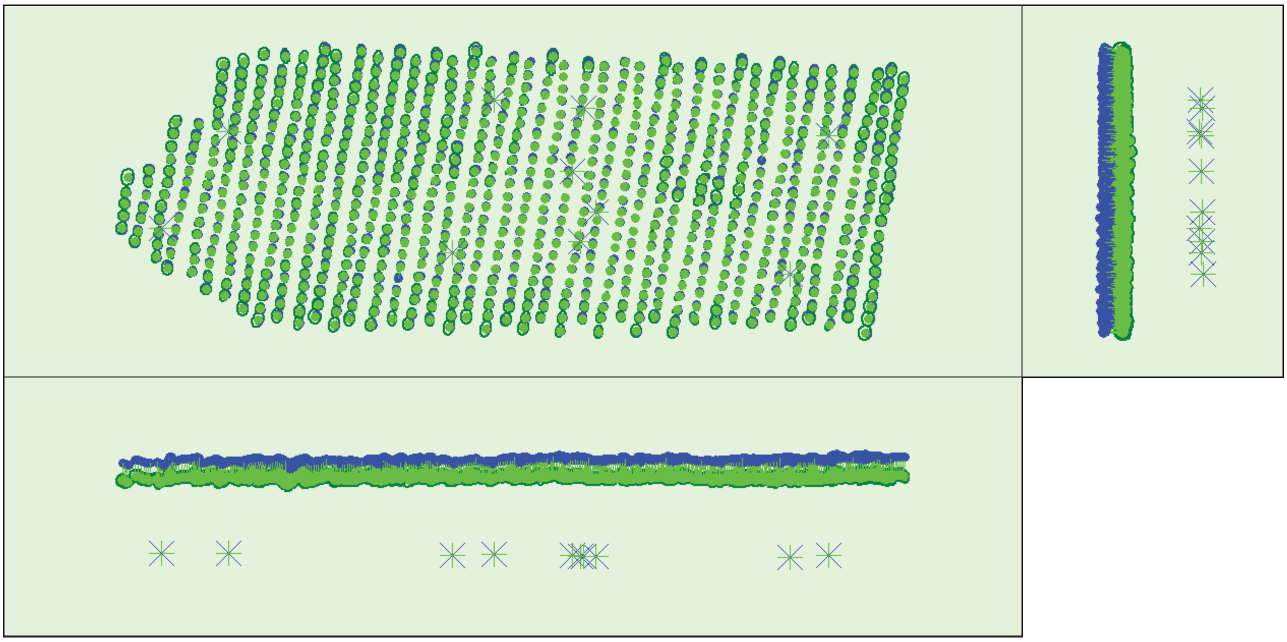

Uncertainty ellipses 500x magnified

Figure 3: Offset between initial (blue dots) and computed (green dots) image positions as well as the offset between the GCPs initial positions (blue crosses) and their computed positions (green crosses) in the top-view (XY plane), front-view (XZ plane), and side-view (YZ plane). Dark green ellipses indicate the absolute position uncertainty of the bundle block adjustment result.

Absolute camera position and orientation uncertainties

	X [m]	Y [m]	Z [m]	Omega [degree]	Phi [degree]	Kappa [degree]
Mean	0.014	0.018	0.023	0.006	0.005	0.002
Sigma	0.004	0.004	0.001	0.002	0.001	0.001

Overlap

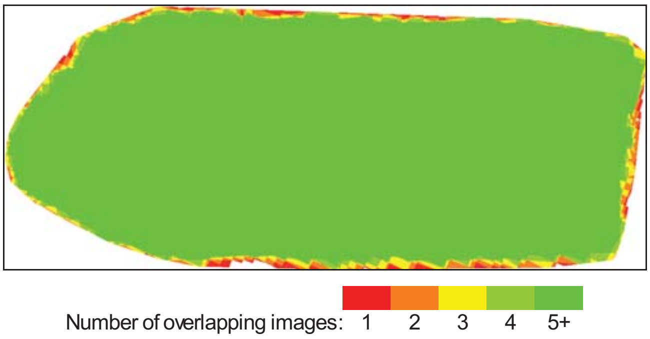

Figure 4: Number of overlapping images computed for each pixel of the orthomosaic.
Red and yellow areas indicate low overlap for which poor results may be generated. Green areas indicate an overlap of over 5 images for every pixel. Good quality results will be generated as long as the number of keypoint matches is also sufficient for these areas (see Figure 5 for keypoint matches).

Bundle Block Adjustment Details

Number of 2D Keypoint Observations for Bundle Block Adjustment	12681741
Number of 3D Points for Bundle Block Adjustment	3199093
Mean Reprojection Error [pixels]	0.188

Internal Camera Parameters

NEX-5T_0.0_4912x3264 (RGB). Sensor Dimensions: 23.400 [mm] x 15.549 [mm]

EXIF ID: NEX-5T_0.0_4912x3264

	Focal Length	Principal Point x	Principal Point y	R1	R2	R3	T1	T2
Initial Values	3256.554 [pixel] 15.514 [mm]	2456.002 [pixel] 11.700 [mm]	1632.003 [pixel] 7.775 [mm]	-0.043	0.028	-0.005	0.000	-0.001
Optimized Values	3250.191 [pixel] 15.483 [mm]	2466.996 [pixel] 11.752 [mm]	1589.788 [pixel] 7.573 [mm]	-0.047	0.035	-0.009	0.000	0.000
Uncertainties (Sigma)	0.478 [pixel] 0.002 [mm]	0.067 [pixel] 0.000 [mm]	0.071 [pixel] 0.000 [mm]	0.000	0.000	0.000	0.000	0.000

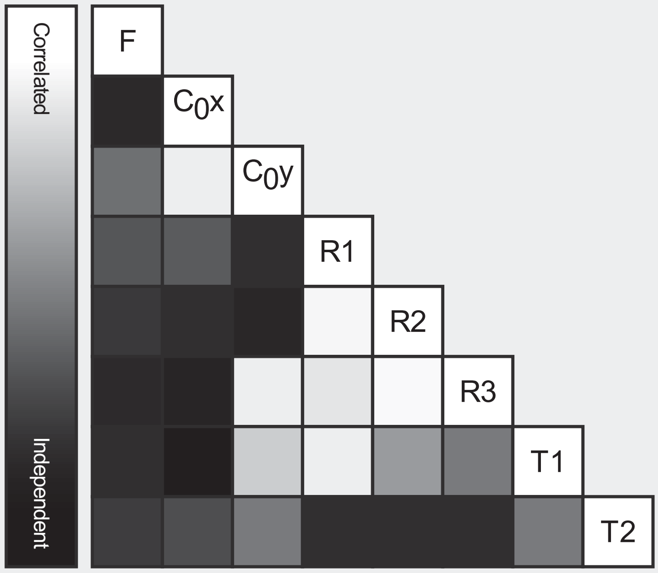

The correlation between camera internal parameters determined by the bundle adjustment. White indicates a full correlation between the parameters, ie. any change in one can be fully compensated by the other. Black indicates that the parameter is completely independent, and is not affected by other parameters.

The number of Automatic Tie Points (ATPs) per pixel, averaged over all images of the camera model, is color coded between black and white. White indicates that, on average, more than 16 ATPs have been extracted at the pixel location. Black indicates that, on average, 0 ATPs have been extracted at the pixel location. Click on the image to the see the average direction and magnitude of the re-projection error for each pixel. Note that the vectors are scaled for better visualization. The scale bar indicates the magnitude of 1 pixel error.

2D Keypoints Table

	Number of 2D Keypoints per Image	Number of Matched 2D Keypoints per Image
Median	45097	14881
Min	20000	2356
Max	68781	30870
Mean	42501	14781

3D Points from 2D Keypoint Matches

	Number of 3D Points Observed
In 2 Images	1628447

In 3 Images	591714
In 4 Images	295632
In 5 Images	174255
In 6 Images	113053
In 7 Images	77888
In 8 Images	57420
In 9 Images	43478
In 10 Images	34044
In 11 Images	27172
In 12 Images	22261
In 13 Images	18276
In 14 Images	15214
In 15 Images	12961
In 16 Images	10961
In 17 Images	9491
In 18 Images	8332
In 19 Images	7230
In 20 Images	6232
In 21 Images	5523
In 22 Images	4844
In 23 Images	4445
In 24 Images	3910
In 25 Images	3359
In 26 Images	3092
In 27 Images	2654
In 28 Images	2260
In 29 Images	2125
In 30 Images	1898
In 31 Images	1632
In 32 Images	1529
In 33 Images	1314
In 34 Images	1084
In 35 Images	970
In 36 Images	877
In 37 Images	751
In 38 Images	613
In 39 Images	554
In 40 Images	423
In 41 Images	367
In 42 Images	286
In 43 Images	205
In 44 Images	133
In 45 Images	95
In 46 Images	47
In 47 Images	22
In 48 Images	11
In 49 Images	5
In 50 Images	3
In 51 Images	1

2D Keypoint Matches

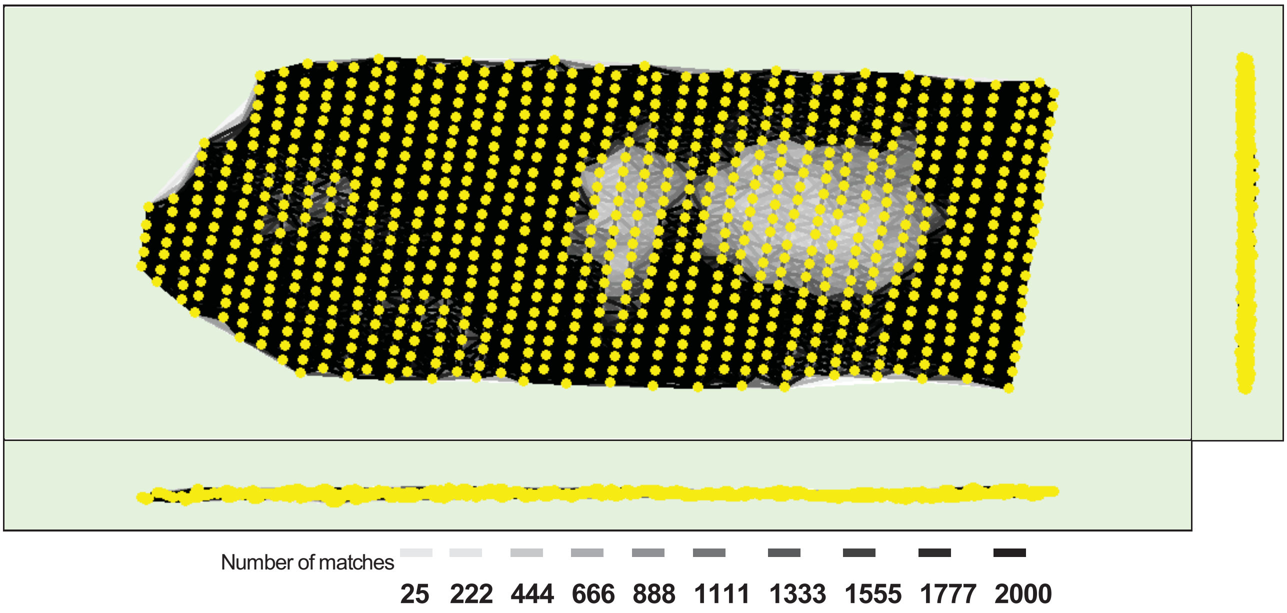

Figure 5: Computed image positions with links between matched images. The darkness of the links indicates the number of matched 2D keypoints between the images. Bright links indicate weak links and require manual tie points or more images.

Geolocation Details

Ground Control Points

GCP Name	Accuracy XY/Z [m]	Error X [m]	Error Y [m]	Error Z [m]	Projection Error [pixel]	Verified/Marked
201 (3D)	0.030/ 0.050	-0.004	-0.025	0.006	0.747	34 / 34
202 (3D)	0.030/ 0.050	0.001	0.018	0.007	0.697	31 / 31
203 (3D)	0.030/ 0.050	-0.003	0.020	-0.018	0.744	39 / 39
208 (3D)	0.030/ 0.050	-0.005	-0.016	-0.001	0.617	38 / 38
209 (3D)	0.030/ 0.050	0.014	-0.020	-0.029	0.459	37 / 37
207 (3D)	0.030/ 0.050	-0.020	-0.017	0.070	0.474	35 / 35
211 (3D)	0.030/ 0.050	0.013	0.030	0.004	0.508	44 / 44
206 (3D)	0.030/ 0.050	0.005	0.029	0.016	0.406	35 / 35
205 (3D)	0.030/ 0.050	-0.011	-0.034	0.010	0.857	40 / 40
204 (3D)	0.030/ 0.050	0.007	0.014	-0.062	0.870	38 / 39
**Mean [m]**		-0.000038	-0.000045	0.000231		
**Sigma [m]**		0.010029	0.023313	0.032052		
**RMS Error [m]**		0.010029	0.023313	0.032053		

0 out of 208 check points have been labeled as inaccurate.

Check Point Name	Accuracy XY/Z [m]	Error X [m]	Error Y [m]	Error Z [m]	Projection Error [pixel]	Verified/Marked
103		0.0119	0.0170	-0.0037	0.4334	42 / 42
101		-0.0073	0.0149	0.0279	0.4056	38 / 38
100		-0.0086	-0.0284	0.0492	0.3573	36 / 36
104		-0.0372	-0.0103	0.0033	0.5070	39 / 39
102		-0.0141	-0.0181	0.0127	0.3557	43 / 43
k1		0.0339	-0.0499	0.1319	0.5067	38 / 38
k2		0.0037	0.0262	0.0103	0.4516	43 / 43
k3		0.0902	-0.0733	0.1807	0.6141	26 / 26
k4		0.0244	-0.0221	0.0808	0.5001	36 / 36
k5		-0.0219	0.0334	-0.0150	0.4280	38 / 38
k6		-0.0277	0.0111	0.0630	0.7251	29 / 29
k7		-0.0778	0.0040	0.1544	0.5407	28 / 28
k8		-0.0395	-0.0322	0.1246	0.5816	30 / 30
k11		-0.0107	-0.0218	-0.0381	0.5108	43 / 43

k13		-0.0024	0.0107	0.0273	0.3994	29 / 29
k14		0.0007	0.0101	0.0258	0.4789	38 / 38
10000		-0.0283	-0.0049	0.0568	0.5844	32 / 32
10001		0.0013	0.0017	0.0765	0.5753	35 / 35
10002		0.0073	-0.0296	0.0145	0.4531	37 / 37
10003		0.0032	-0.0401	-0.0074	0.5173	40 / 40
10004		0.0187	-0.0146	0.0310	0.5024	39 / 39
10005		0.0250	-0.0215	0.0248	0.5574	43 / 43
10006		0.0003	-0.0232	0.0124	0.3991	42 / 42
10007		0.0066	-0.0201	-0.0040	0.3873	41 / 41
10008		0.0158	-0.0301	-0.0096	0.3576	38 / 38
10009		0.0087	-0.0277	0.0024	0.4694	42 / 42
10010		0.0076	-0.0285	-0.0083	0.4626	44 / 44
10011		0.0100	-0.0393	-0.0121	0.5425	33 / 33
10012		-0.0339	0.0067	0.0095	0.5534	40 / 40
10013		-0.0187	-0.0001	0.0056	0.4264	38 / 38
10014		0.0057	0.0103	0.0160	0.3772	42 / 42
10015		0.0154	0.0024	0.0333	0.4297	40 / 40
10016		-0.0233	-0.0019	0.0282	0.5110	43 / 43
10017		0.0040	-0.0142	-0.0268	0.4213	42 / 42
10018		0.0040	-0.0038	-0.0082	0.3696	22 / 22
10019		-0.0081	0.0164	0.0554	0.6172	42 / 42
10020		-0.0219	0.0105	0.0258	0.4548	33 / 33
10021		0.0000	-0.0005	0.0420	0.4155	45 / 45
10022		0.0190	0.0294	0.0150	0.5281	42 / 42
10023		0.0237	-0.0064	0.0031	0.4182	38 / 38
10024		-0.0079	-0.0003	-0.0208	0.3927	46 / 46
10025		0.0080	-0.0001	0.0054	0.3849	44 / 44
10026		-0.0081	0.0230	0.0035	0.4548	36 / 36
10027		-0.0126	0.0177	-0.0006	0.5052	41 / 41
10028		-0.0004	0.0034	0.0117	0.4245	43 / 43
10030		-0.0080	0.0310	-0.0117	0.3873	37 / 37
10031		0.0330	0.0269	0.0065	0.5315	27 / 27
10032		0.0399	0.0359	0.0278	0.4586	27 / 27
10033		0.0040	0.0297	0.0274	0.3791	40 / 40
10034		0.0274	-0.0105	0.0145	0.4006	38 / 38
10035		0.0139	-0.0282	0.0687	0.3993	35 / 35
10036		0.0155	-0.0079	0.0192	0.5564	27 / 27
10037		-0.0005	-0.0114	0.0068	0.3915	39 / 39
10038		-0.0038	0.0074	0.0071	0.3809	42 / 42
10039		0.0055	-0.0056	0.0052	0.4154	43 / 43
10040		-0.0232	-0.0093	0.0179	0.4854	31 / 31
10041		-0.0151	-0.0237	0.0161	0.5555	35 / 35
10044		0.0109	-0.0455	-0.0137	0.4958	38 / 38
10045		0.0188	-0.0397	-0.0439	0.3841	36 / 36
10046		-0.0127	-0.0116	-0.0671	0.3799	38 / 38
10047		-0.0281	-0.0080	0.0766	0.7257	31 / 31
10048		-0.0143	-0.0245	0.0486	0.6424	32 / 32
10049		-0.0217	0.0261	0.0449	0.8376	32 / 32
10051		0.0000	0.0182	0.0168	0.3849	44 / 44
10052		-0.0030	0.0033	0.0234	0.5285	35 / 35
10053		-0.0127	0.0126	0.0116	0.4073	40 / 40
10054		-0.0112	-0.0176	-0.0042	0.4258	38 / 38
10055		0.0011	0.0125	-0.0131	0.5688	39 / 39
10056		-0.0361	0.0121	0.0053	0.5275	38 / 38
10058		-0.0692	0.0874	0.0389	0.5362	44 / 44
10059		-0.0097	-0.0252	-0.0411	0.3644	40 / 40
10060		-0.0283	-0.0251	-0.0219	0.3562	43 / 43
10061		0.0066	0.0250	0.0409	0.4857	29 / 29

10062		-0.0029	-0.0253	-0.0036	0.4726	41 / 41
10063		0.0168	-0.0462	-0.0306	0.4777	38 / 38
10064		0.0341	0.0189	-0.0322	0.4420	43 / 43
10065		0.0252	0.0263	-0.0115	0.3673	44 / 44
10066		0.0527	0.0294	-0.0226	0.4353	41 / 41
10067		0.0273	0.0307	-0.0116	0.3625	42 / 42
10068		0.0092	0.1013	-0.0200	0.4500	43 / 43
10074		-0.0237	0.0464	-0.0321	0.6875	41 / 41
10075		-0.0188	0.0440	0.0141	0.3196	41 / 41
10076		0.0011	0.0214	-0.0009	0.3544	40 / 40
10077		-0.0221	0.0547	-0.0112	0.7348	38 / 38
10078		0.0216	0.0127	-0.0058	0.3335	39 / 39
10080		0.0174	-0.0634	-0.0828	0.6886	35 / 35
10081		-0.0211	-0.0267	0.0091	0.3514	37 / 37
10082		0.0125	-0.0395	-0.0229	0.4179	39 / 39
10083		0.0511	-0.0161	-0.0620	0.5945	37 / 37
10085		0.0319	-0.0341	0.0049	0.3383	44 / 44
15000		0.0069	-0.0412	-0.0291	0.3466	46 / 46
15001		-0.0260	-0.0442	-0.0355	0.3667	39 / 39
15002		0.0084	0.0173	-0.0688	0.4707	41 / 41
15003		-0.0047	0.0360	-0.0056	0.4720	45 / 45
15004		-0.0049	0.0156	-0.0122	0.3685	43 / 43
15005		-0.0044	0.0237	-0.0054	0.5341	38 / 38
15006		0.0065	0.0359	-0.0137	0.4414	43 / 43
15007		0.0106	-0.0012	0.0302	0.4778	38 / 38
15008		-0.0219	0.0203	0.0674	0.7868	38 / 38
15009		-0.0098	0.0133	0.0616	0.5511	40 / 40
15010		0.0058	0.0388	0.0479	0.6375	38 / 38
20000		-0.0144	0.0212	0.0303	0.7521	42 / 42
20001		-0.0099	0.0100	0.0522	0.5117	38 / 38
20002		0.0017	0.0240	0.0536	0.4325	38 / 38
20003		-0.0230	-0.0048	0.0469	0.4249	39 / 39
20004		-0.0024	-0.0105	0.0374	0.4032	36 / 36
20005		-0.0474	-0.0274	-0.0032	0.5271	35 / 35
20008		-0.0036	-0.0006	0.0027	0.3932	44 / 44
20009		0.0114	-0.0072	-0.0158	0.3869	43 / 43
20010		-0.0231	0.0237	-0.0099	0.4072	45 / 45
20011		-0.0173	-0.0012	0.0066	0.3795	47 / 47
20012		0.0078	-0.0009	-0.0059	0.3499	44 / 44
20013		0.0100	-0.0014	0.0101	0.3573	41 / 41
20014		-0.0008	0.0071	-0.0023	0.3557	43 / 43
20016		0.0008	0.0153	-0.0398	0.3268	46 / 46
20017		0.0116	0.0249	-0.0238	0.3644	40 / 40
20018		0.0223	0.0218	-0.0116	0.3295	44 / 44
20019		0.0269	0.0133	0.0026	0.4139	39 / 39
20020		0.0076	0.0370	-0.0008	0.4320	40 / 40
20021		0.0064	-0.0007	0.0218	0.2628	40 / 40
20022		-0.0172	-0.0526	0.0204	0.3089	42 / 42
20023		0.0000	0.0019	0.0047	0.3405	45 / 45
20024		-0.0036	0.0387	-0.0237	0.4333	41 / 41
20025		0.0121	0.0465	-0.0150	0.3518	42 / 42
20026		0.0117	0.0365	-0.0120	0.4054	43 / 43
20027		0.0060	0.0712	-0.0097	0.3877	40 / 40
20028		-0.0047	0.0117	0.0096	0.4080	41 / 41
20029		0.0150	-0.0158	0.0003	0.7745	41 / 41
20031		-0.0096	0.0228	-0.0002	0.4397	45 / 45
25000		0.0161	0.0106	-0.0032	0.2533	40 / 40
25001		0.0041	0.0080	0.0363	0.3053	40 / 40
210		-0.0012	-0.0022	0.0609	0.2657	43 / 43

25003		0.0041	-0.0185	0.0315	0.3707	40 / 40
25004		0.0053	0.0014	0.0064	0.3427	33 / 33
25005		0.0068	-0.0107	0.0231	0.3012	39 / 39
25006		-0.0311	-0.0434	-0.0264	0.4639	38 / 38
25008		-0.0302	-0.0016	-0.0247	0.2306	38 / 38
25009		0.0290	-0.0514	-0.0332	0.2574	42 / 42
25010		0.0064	-0.0195	-0.0379	0.2619	41 / 41
25013		-0.0020	0.0152	-0.0261	0.3988	38 / 38
25014		-0.0103	-0.0082	-0.0389	0.3040	37 / 37
25015		-0.0239	-0.0101	-0.0330	0.4327	41 / 41
25016		-0.0245	-0.0091	-0.0293	0.2831	42 / 42
25017		0.0240	-0.0178	0.0445	0.4319	37 / 37
25018		0.0081	-0.0005	0.0313	0.3971	38 / 38
25019		-0.0131	-0.0105	0.0214	0.4784	39 / 39
25020		-0.0088	0.0060	-0.0092	0.3724	33 / 33
25021		0.0183	0.0250	0.0069	0.3654	36 / 36
25022		0.0157	0.0061	0.0208	0.4867	34 / 34
25023		-0.0037	0.0248	0.0039	0.4242	40 / 40
25024		0.0018	-0.0131	-0.0040	0.3480	40 / 40
25029		0.0114	-0.0055	0.0038	0.3335	42 / 42
25030		-0.0080	-0.0101	0.0141	0.3979	44 / 44
25032		-0.0723	-0.0254	-0.0280	0.4236	36 / 36
25033		-0.0214	-0.0199	0.0014	0.3757	41 / 41
25034		-0.0154	-0.0192	-0.0056	0.4027	36 / 36
40000		0.0074	-0.0171	-0.0869	0.3010	42 / 42
40001		0.0210	0.0176	-0.0076	0.2914	43 / 43
40002		0.0173	0.0396	-0.0277	0.3881	42 / 42
40003		0.0096	0.0438	0.0005	0.3925	39 / 39
40004		0.0047	0.0047	-0.0049	0.2982	37 / 37
40005		0.0185	0.0243	-0.0269	0.2857	29 / 29
45000		0.0348	-0.0285	0.0122	0.4154	44 / 44
45001		-0.0038	0.0036	0.0067	0.4082	34 / 34
45002		0.0135	-0.0328	0.0060	0.4960	41 / 41
10043		-0.0143	-0.0074	0.0510	0.6463	35 / 35
10084		-0.0103	-0.0043	0.0787	0.5572	30 / 30
20015		0.0003	-0.0102	-0.0425	0.3293	45 / 45
20007		-0.0012	-0.0083	-0.0083	0.3915	24 / 24
25027		-0.0005	-0.0093	-0.0383	0.3163	43 / 43
k9		-0.0038	-0.0145	-0.0318	0.5536	42 / 42
6600		-0.0013	-0.0194	-0.0345	0.4096	41 / 41
6602		0.0012	0.0285	-0.0266	0.3827	37 / 37
6603		-0.0040	-0.0200	-0.0010	0.4394	40 / 40
6605		0.0108	0.0172	0.0101	0.4952	40 / 40
6606		0.0165	0.0289	0.0091	0.5742	25 / 25
6607		0.0010	0.0112	-0.0392	0.7231	35 / 35
6608		-0.0063	-0.0168	0.0049	0.6749	21 / 21
6609		-0.0149	0.0041	0.0004	0.3857	42 / 42
6610		0.0037	0.0037	0.0198	0.7437	33 / 33
6611		-0.0122	-0.0249	-0.0635	0.5630	40 / 40
6612		-0.0044	-0.0556	-0.0732	0.7125	38 / 38
6616		-0.0055	-0.0381	-0.0337	0.4981	33 / 33
6617		-0.0068	-0.0050	0.0254	0.5940	23 / 23
6618		-0.0063	-0.0030	-0.0102	0.5128	40 / 40
6619		0.0206	0.0005	0.0176	0.2793	41 / 41
6620		0.0183	-0.0455	-0.0031	0.4069	38 / 38
6621		-0.0217	-0.0089	0.0083	0.2317	41 / 41
6622		0.0057	0.0085	-0.0180	0.5965	37 / 37
6623		0.0017	-0.0146	-0.0339	0.7773	40 / 40
6624		0.0519	-0.0033	-0.0159	0.7110	33 / 33

6625		-0.0497	-0.0260	-0.0292	0.5842	37 / 37
6626		-0.0187	-0.0089	-0.0627	0.4227	35 / 35
6627		-0.0288	-0.0243	-0.0266	0.5998	33 / 33
6628		-0.0349	-0.0321	-0.0490	0.5067	39 / 39
6629		-0.0483	-0.0285	-0.0463	0.4746	37 / 37
6630		-0.0571	-0.0128	-0.0358	0.4028	34 / 34
6631		-0.0316	-0.0293	-0.0396	0.5762	32 / 32
6632		-0.0466	-0.0202	-0.0187	0.6045	34 / 34
6633		-0.0095	-0.0190	-0.0312	0.4360	38 / 38
6634		-0.0157	0.0011	-0.0295	0.4083	35 / 35
6635		-0.0030	0.0026	-0.0293	0.4708	39 / 39
6636		-0.0458	-0.0311	-0.0423	0.5373	41 / 41
6637		-0.0072	-0.0095	-0.0237	0.6340	31 / 31
6638		-0.0260	-0.0096	-0.0296	0.4520	27 / 27
6639		-0.0524	-0.0034	-0.0371	0.5538	38 / 38
6640		-0.0381	-0.0213	-0.0410	0.5108	34 / 34
6641		-0.0471	-0.0154	-0.0390	0.5691	37 / 37
**Mean [m]**		-0.002707	-0.001755	0.001815		
**Sigma [m]**		0.022301	0.025904	0.037384		
**RMS Error [m]**		0.022465	0.025963	0.037428		

Localisation accuracy per GCP and mean errors in the three coordinate directions. The last column counts the number of calibrated images where the GCP has been automatically verified vs. manually marked.

Absolute Geolocation Variance

Min Error [m]	Max Error [m]	Geolocation Error X [%]	Geolocation Error Y [%]	Geolocation Error Z [%]
-	-15.00	0.00	0.23	0.00
-15.00	-12.00	0.00	0.00	0.00
-12.00	-9.00	0.00	0.00	0.00
-9.00	-6.00	0.00	0.00	0.00
-6.00	-3.00	0.23	5.24	8.04
-3.00	0.00	47.32	47.67	39.51
0.00	3.00	52.33	37.65	45.45
3.00	6.00	0.12	8.97	6.99
6.00	9.00	0.00	0.00	0.00
9.00	12.00	0.00	0.00	0.00
12.00	15.00	0.00	0.00	0.00
15.00	-	0.00	0.23	0.00
**Mean [m]**		0.858177	1.668415	32.652735
**Sigma [m]**		1.128382	2.666315	2.151081
**RMS Error [m]**		1.417644	3.145289	32.723513

Min Error and Max Error represent geolocation error intervals between -1.5 and 1.5 times the maximum accuracy of all the images. Columns X, Y, Z show the percentage of images with geolocation errors within the predefined error intervals. The geolocation error is the difference between the initial and computed image positions. Note that the image geolocation errors do not correspond to the accuracy of the observed 3D points.

Geolocation Bias	X	Y	Z
Translation [m]	0.858181	1.668409	32.652736

Bias between image initial and computed geolocation given in output coordinate system.

Relative Geolocation Variance

Relative Geolocation Error	Images X [%]	Images Y [%]	Images Z [%]
[-1.00, 1.00]	100.00	98.72	100.00
[-2.00, 2.00]	100.00	99.53	100.00

[-3.00, 3.00]	100.00	99.53	100.00
**Mean of Geolocation Accuracy [m]**	5.000000	5.000000	10.000000
**Sigma of Geolocation Accuracy [m]**	0.000000	0.000000	0.000000

Images X, Y, Z represent the percentage of images with a relative geolocation error in X, Y, Z.

Initial Processing Details

System Information

Hardware	CPU: Intel(R) Core(TM) i9-7940X CPU @ 3.10GHz<br>RAM: 128GB<br>GPU: NVIDIA GeForce GTX 1080 Ti (Driver: 22.21.13.8541), RDPDD Chained DD (Driver: unknown), RDP Encoder Mirror Driver (Driver: unknown), RDP Reflector Display Driver (Driver: unknown)
Operating System	Windows 7 Ultimate, 64-bit

Coordinate Systems

Image Coordinate System	WGS 84 (EGM96 Geoid)
Ground Control Point (GCP) Coordinate System	ETRS89 / Poland CS2000 zone 6 (EGM2008 Geoid)
Output Coordinate System	ETRS89 / Poland CS2000 zone 6 (EGM2008 Geoid)

Processing Options

Detected Template	3D Maps
Keypoints Image Scale	Full, Image Scale: 1
Advanced: Matching Image Pairs	Aerial Grid or Corridor
Advanced: Matching Strategy	Use Geometrically Verified Matching: no
Advanced: Keypoint Extraction	Targeted Number of Keypoints: Automatic
Advanced: Calibration	Calibration Method: Standard<br>Internal Parameters Optimization: All<br>External Parameters Optimization: All<br>Rematch: Auto, no
