# Supplementary material for: Multi-Variant Accuracy Evaluation of UAV Imaging Surveys: A Case Study on Investment Area
Source: Sensors (Basel). 2019 Nov 28;19(23):5229. doi: 10.3390/s19235229 (PMC6929115; doi:10.3390/s19235229)
Supplement: Supplementary file 1 [file sensors-19-05229-s001.zip › supplementary_files/App6_V6_report.pdf]

Quality Report

Generated with Pix4Ddiscovery version 4.3.31

Important: Click on the different icons for:

Help to analyze the results in the Quality Report

Additional information about the sections

Click here for additional tips to analyze the Quality Report

Summary

Project	Sosnowiec_2_new_ver_11GCP
Processed	2019-02-22 09:00:16
Camera Model Name(s)	NEX-5T_0.0_4912x3264 (RGB)
Average Ground Sampling Distance (GSD)	4.59 cm / 1.81 in
Area Covered	1.029 km^2 / 102.8861 ha / 0.40 sq. mi. / 254.3686 acres
Time for Initial Processing (without report)	01h:17m:10s

Quality Check

**Images**	median of 45097 keypoints per image	✓
**Dataset**	858 out of 858 images calibrated (100%), all images enabled	✓
**Camera Optimization**	0.19% relative difference between initial and optimized internal camera parameters	✓
**Matching**	median of 14882.8 matches per calibrated image	✓
**Georeferencing**	yes, 11 GCPs (11 3D), mean RMS error = 0.021 m	✓

Preview

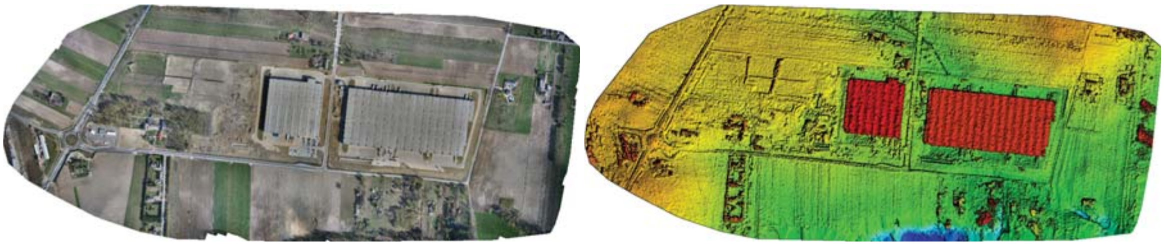

Figure 1: Orthomosaic and the corresponding sparse Digital Surface Model (DSM) before densification.

Calibration Details

Number of Calibrated Images	858 out of 858
Number of Geolocated Images	858 out of 858

Initial Image Positions

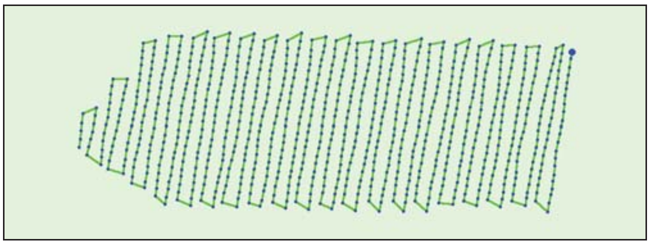

Figure 2: Top view of the initial image position. The green line follows the position of the images in time starting from the large blue dot.

Computed Image/GCPs/Manual Tie Points Positions

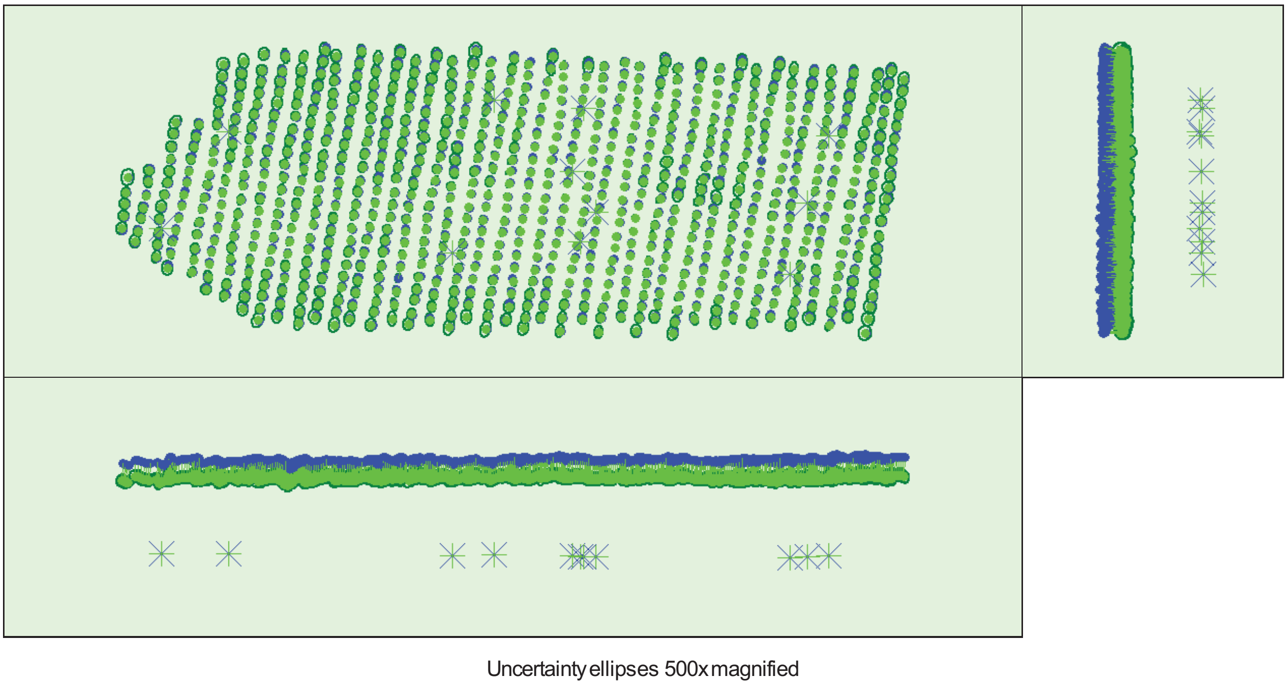

Figure 3: Offset between initial (blue dots) and computed (green dots) image positions as well as the offset between the GCPs initial positions (blue crosses) and their computed positions (green crosses) in the top-view (XY plane), front-view (XZ plane), and side-view (YZ plane). Dark green ellipses indicate the absolute position uncertainty of the bundle block adjustment result.

Absolute camera position and orientation uncertainties

	X[m]	Y[m]	Z[m]	Omega [degree]	Phi [degree]	Kappa [degree]
Mean	0.014	0.018	0.023	0.006	0.005	0.002
Sigma	0.004	0.004	0.001	0.002	0.001	0.001

Overlap

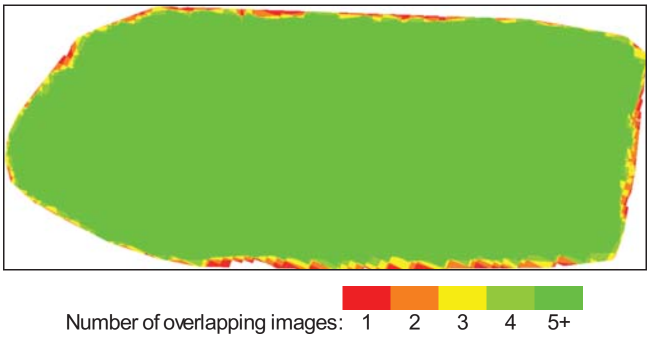

Figure 4: Number of overlapping images computed for each pixel of the orthomosaic.
Red and yellow areas indicate low overlap for which poor results may be generated. Green areas indicate an overlap of over 5 images for every pixel. Good quality results will be generated as long as the number of keypoint matches is also sufficient for these areas (see Figure 5 for keypoint matches).

Bundle Block Adjustment Details

Number of 2D Keypoint Observations for Bundle Block Adjustment	12681720
Number of 3D Points for Bundle Block Adjustment	3199095
Mean Reprojection Error [pixels]	0.188

Internal Camera Parameters

NEX-5T_0.0_4912x3264 (RGB). Sensor Dimensions: 23.400 [mm] x 15.549 [mm]

EXIF ID: NEX-5T_0.0_4912x3264

	Focal Length	Principal Point x	Principal Point y	R1	R2	R3	T1	T2
Initial Values	3256.554 [pixel] 15.514 [mm]	2456.002 [pixel] 11.700 [mm]	1632.003 [pixel] 7.775 [mm]	-0.043	0.028	-0.005	0.000	-0.001
Optimized Values	3250.161 [pixel] 15.483 [mm]	2466.995 [pixel] 11.752 [mm]	1589.790 [pixel] 7.574 [mm]	-0.047	0.035	-0.009	0.000	0.000
Uncertainties (Sigma)	0.478 [pixel] 0.002 [mm]	0.067 [pixel] 0.000 [mm]	0.071 [pixel] 0.000 [mm]	0.000	0.000	0.000	0.000	0.000

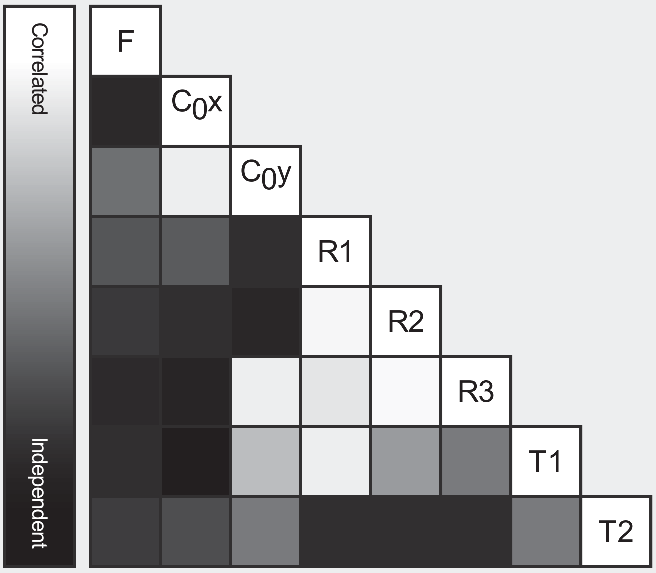

The correlation between camera internal parameters determined by the bundle adjustment. White indicates a full correlation between the parameters, ie. any change in one can be fully compensated by the other. Black indicates that the parameter is completely independent, and is not affected by other parameters.

The number of Automatic Tie Points (ATPs) per pixel, averaged over all images of the camera model, is color coded between black and white. White indicates that, on average, more than 16 ATPs have been extracted at the pixel location. Black indicates that, on average, 0 ATPs have been extracted at the pixel location. Click on the image to the see the average direction and magnitude of the re-projection error for each pixel. Note that the vectors are scaled for better visualization. The scale bar indicates the magnitude of 1 pixel error.

2D Keypoints Table

	Number of 2D Keypoints per Image	Number of Matched 2D Keypoints per Image
Median	45097	14883
Min	20000	2356
Max	68781	30865
Mean	42501	14781

3D Points from 2D Keypoint Matches

	Number of 3D Points Observed
In 2 Images	1628444

In 3 Images	591720
In 4 Images	295623
In 5 Images	174244
In 6 Images	113055
In 7 Images	77897
In 8 Images	57427
In 9 Images	43478
In 10 Images	34051
In 11 Images	27169
In 12 Images	22258
In 13 Images	18279
In 14 Images	15218
In 15 Images	12962
In 16 Images	10963
In 17 Images	9492
In 18 Images	8328
In 19 Images	7230
In 20 Images	6229
In 21 Images	5522
In 22 Images	4844
In 23 Images	4444
In 24 Images	3910
In 25 Images	3359
In 26 Images	3092
In 27 Images	2654
In 28 Images	2258
In 29 Images	2126
In 30 Images	1898
In 31 Images	1631
In 32 Images	1529
In 33 Images	1313
In 34 Images	1085
In 35 Images	970
In 36 Images	877
In 37 Images	751
In 38 Images	613
In 39 Images	554
In 40 Images	423
In 41 Images	367
In 42 Images	286
In 43 Images	205
In 44 Images	133
In 45 Images	95
In 46 Images	47
In 47 Images	22
In 48 Images	11
In 49 Images	5
In 50 Images	3
In 51 Images	1

2D Keypoint Matches

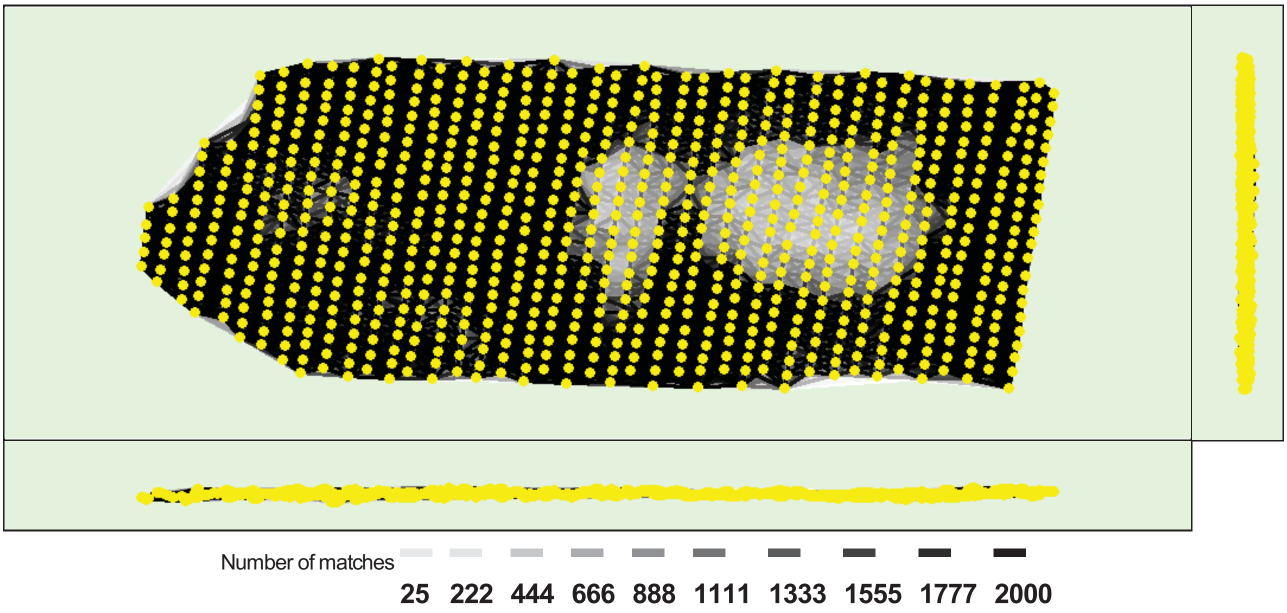

Figure 5: Computed image positions with links between matched images. The darkness of the links indicates the number of matched 2D keypoints between the images. Bright links indicate weak links and require manual tie points or more images.

Geolocation Details

Ground Control Points

GCP Name	Accuracy XY/Z [m]	Error X [m]	Error Y [m]	Error Z [m]	Projection Error [pixel]	Verified/Marked
201 (3D)	0.030/ 0.050	-0.004	-0.025	0.006	0.747	34 / 34
202 (3D)	0.030/ 0.050	0.001	0.018	0.007	0.697	31 / 31
203 (3D)	0.030/ 0.050	-0.003	0.020	-0.017	0.744	39 / 39
208 (3D)	0.030/ 0.050	-0.005	-0.016	-0.002	0.617	38 / 38
209 (3D)	0.030/ 0.050	0.015	-0.019	-0.046	0.459	37 / 37
207 (3D)	0.030/ 0.050	-0.019	-0.017	0.068	0.474	35 / 35
211 (3D)	0.030/ 0.050	0.014	0.031	-0.013	0.509	44 / 44
210 (3D)	0.030/ 0.050	-0.001	-0.001	0.035	0.266	43 / 43
206 (3D)	0.030/ 0.050	0.005	0.029	0.014	0.406	35 / 35
205 (3D)	0.030/ 0.050	-0.011	-0.034	0.008	0.857	40 / 40
204 (3D)	0.030/ 0.050	0.007	0.014	-0.060	0.870	38 / 39
**Mean [m]**		-0.000029	-0.000044	0.000181		
**Sigma [m]**		0.009626	0.022241	0.033349		
**RMS Error [m]**		0.009626	0.022241	0.033350		

0 out of 207 check points have been labeled as inaccurate.

Check Point Name	Accuracy XY/Z [m]	Error X [m]	Error Y [m]	Error Z [m]	Projection Error [pixel]	Verified/Marked
103		0.0121	0.0170	-0.0046	0.4332	42 / 42
101		-0.0068	0.0155	0.0123	0.4060	38 / 38
100		-0.0084	-0.0279	0.0325	0.3575	36 / 36
104		-0.0373	-0.0103	0.0058	0.5070	39 / 39
102		-0.0141	-0.0179	0.0113	0.3557	43 / 43
k1		0.0340	-0.0499	0.1309	0.5068	38 / 38
k2		0.0038	0.0262	0.0092	0.4515	43 / 43
k3		0.0903	-0.0732	0.1796	0.6142	26 / 26
k4		0.0245	-0.0220	0.0797	0.5001	36 / 36
k5		-0.0218	0.0334	-0.0163	0.4280	38 / 38
k6		-0.0275	0.0112	0.0604	0.7250	29 / 29
k7		-0.0776	0.0041	0.1518	0.5407	28 / 28
k8		-0.0393	-0.0320	0.1217	0.5816	30 / 30

k11		-0.0108	-0.0217	-0.0374	0.5109	43 / 43
k13		-0.0021	0.0114	0.0042	0.4025	29 / 29
k14		0.0010	0.0110	0.0015	0.4795	38 / 38
10000		-0.0278	-0.0042	0.0393	0.5846	32 / 32
10001		0.0017	0.0024	0.0552	0.5758	35 / 35
10002		0.0075	-0.0289	-0.0013	0.4536	37 / 37
10003		0.0034	-0.0395	-0.0214	0.5175	40 / 40
10004		0.0190	-0.0141	0.0212	0.5025	39 / 39
10005		0.0252	-0.0210	0.0156	0.5578	43 / 43
10006		0.0005	-0.0227	0.0044	0.3989	42 / 42
10007		0.0068	-0.0197	-0.0104	0.3871	41 / 41
10008		0.0160	-0.0297	-0.0155	0.3574	38 / 38
10009		0.0089	-0.0274	-0.0027	0.4695	42 / 42
10010		0.0078	-0.0281	-0.0132	0.4624	44 / 44
10011		0.0101	-0.0389	-0.0161	0.5425	33 / 33
10012		-0.0338	0.0070	0.0067	0.5535	40 / 40
10013		-0.0187	0.0002	0.0031	0.4266	38 / 38
10014		0.0057	0.0105	0.0140	0.3772	42 / 42
10015		0.0154	0.0026	0.0324	0.4297	40 / 40
10016		-0.0233	-0.0018	0.0283	0.5111	43 / 43
10017		0.0039	-0.0141	-0.0262	0.4211	42 / 42
10018		0.0039	-0.0038	-0.0070	0.3696	22 / 22
10019		-0.0081	0.0163	0.0573	0.6172	42 / 42
10020		-0.0219	0.0105	0.0275	0.4548	33 / 33
10021		0.0001	-0.0005	0.0425	0.4154	45 / 45
10022		0.0191	0.0295	0.0150	0.5280	42 / 42
10023		0.0238	-0.0064	0.0029	0.4182	38 / 38
10024		-0.0078	-0.0003	-0.0202	0.3927	46 / 46
10025		0.0081	-0.0001	0.0059	0.3848	44 / 44
10026		-0.0079	0.0230	0.0034	0.4548	36 / 36
10027		-0.0124	0.0177	-0.0011	0.5053	41 / 41
10028		-0.0002	0.0034	0.0111	0.4245	43 / 43
10030		-0.0078	0.0311	-0.0134	0.3873	37 / 37
10031		0.0332	0.0270	0.0046	0.5315	27 / 27
10032		0.0401	0.0359	0.0245	0.4586	27 / 27
10033		0.0044	0.0298	0.0218	0.3789	40 / 40
10034		0.0279	-0.0104	0.0084	0.4007	38 / 38
10035		0.0144	-0.0280	0.0625	0.3995	35 / 35
10036		0.0161	-0.0077	0.0122	0.5564	27 / 27
10037		0.0001	-0.0112	-0.0029	0.3915	39 / 39
10038		-0.0033	0.0077	-0.0043	0.3805	42 / 42
10039		0.0061	-0.0051	-0.0091	0.4156	43 / 43
10040		-0.0230	-0.0087	-0.0005	0.4855	31 / 31
10041		-0.0149	-0.0231	-0.0014	0.5556	35 / 35
10044		0.0112	-0.0450	-0.0268	0.4963	38 / 38
10045		0.0190	-0.0392	-0.0550	0.3842	36 / 36
10046		-0.0128	-0.0114	-0.0696	0.3799	38 / 38
10047		-0.0279	-0.0077	0.0738	0.7255	31 / 31
10048		-0.0142	-0.0243	0.0457	0.6426	32 / 32
10049		-0.0215	0.0263	0.0420	0.8374	32 / 32
10051		0.0002	0.0182	0.0161	0.3849	44 / 44
10052		-0.0029	0.0034	0.0220	0.5285	35 / 35
10053		-0.0128	0.0128	0.0104	0.4073	40 / 40
10054		-0.0112	-0.0174	-0.0053	0.4258	38 / 38
10055		0.0012	0.0125	-0.0119	0.5687	39 / 39
10056		-0.0361	0.0120	0.0070	0.5277	38 / 38
10058		-0.0692	0.0873	0.0408	0.5360	44 / 44
10059		-0.0096	-0.0248	-0.0462	0.3643	40 / 40
10060		-0.0284	-0.0249	-0.0228	0.3562	43 / 43

10061		0.0065	0.0251	0.0407	0.4858	29 / 29
10062		-0.0029	-0.0252	-0.0038	0.4726	41 / 41
10063		0.0167	-0.0461	-0.0304	0.4778	38 / 38
10064		0.0344	0.0189	-0.0346	0.4422	43 / 43
10065		0.0256	0.0264	-0.0154	0.3670	44 / 44
10066		0.0530	0.0295	-0.0265	0.4350	41 / 41
10067		0.0276	0.0308	-0.0158	0.3624	42 / 42
10068		0.0096	0.1014	-0.0242	0.4499	43 / 43
10074		-0.0233	0.0465	-0.0366	0.6874	41 / 41
10075		-0.0184	0.0441	0.0085	0.3196	41 / 41
10076		0.0015	0.0215	-0.0055	0.3543	40 / 40
10077		-0.0217	0.0548	-0.0176	0.7346	38 / 38
10078		0.0220	0.0130	-0.0147	0.3333	39 / 39
10080		0.0175	-0.0631	-0.0868	0.6886	35 / 35
10081		-0.0210	-0.0264	0.0054	0.3515	37 / 37
10082		0.0126	-0.0392	-0.0267	0.4180	39 / 39
10083		0.0513	-0.0161	-0.0624	0.5946	37 / 37
10085		0.0319	-0.0339	0.0041	0.3382	44 / 44
15000		0.0071	-0.0408	-0.0369	0.3468	46 / 46
15001		-0.0258	-0.0438	-0.0420	0.3669	39 / 39
15002		0.0088	0.0176	-0.0779	0.4706	41 / 41
15003		-0.0044	0.0363	-0.0147	0.4722	45 / 45
15004		-0.0045	0.0159	-0.0234	0.3686	43 / 43
15005		-0.0040	0.0242	-0.0188	0.5339	38 / 38
15006		0.0069	0.0365	-0.0286	0.4415	43 / 43
15007		0.0112	-0.0004	0.0103	0.4768	38 / 38
15008		-0.0214	0.0211	0.0455	0.7863	38 / 38
15009		-0.0095	0.0142	0.0365	0.5516	40 / 40
15010		0.0062	0.0396	0.0234	0.6365	38 / 38
20000		-0.0138	0.0219	0.0130	0.7519	42 / 42
20001		-0.0095	0.0107	0.0307	0.5125	38 / 38
20002		0.0021	0.0248	0.0294	0.4330	38 / 38
20003		-0.0227	-0.0041	0.0224	0.4250	39 / 39
20004		-0.0021	-0.0097	0.0156	0.4028	36 / 36
20005		-0.0473	-0.0272	-0.0061	0.5278	35 / 35
20008		-0.0036	-0.0004	0.0016	0.3931	44 / 44
20009		0.0113	-0.0071	-0.0161	0.3870	43 / 43
20010		-0.0230	0.0237	-0.0092	0.4073	45 / 45
20011		-0.0173	-0.0012	0.0070	0.3795	47 / 47
20012		0.0079	-0.0009	-0.0057	0.3499	44 / 44
20013		0.0101	-0.0014	0.0100	0.3572	41 / 41
20014		-0.0007	0.0071	-0.0028	0.3558	43 / 43
20016		0.0009	0.0153	-0.0392	0.3269	46 / 46
20017		0.0117	0.0249	-0.0234	0.3643	40 / 40
20018		0.0224	0.0218	-0.0115	0.3295	44 / 44
20019		0.0270	0.0134	0.0024	0.4138	39 / 39
20020		0.0078	0.0370	-0.0028	0.4321	40 / 40
20021		0.0068	-0.0006	0.0159	0.2628	40 / 40
20022		-0.0167	-0.0524	0.0141	0.3089	42 / 42
20023		0.0005	0.0020	-0.0019	0.3408	45 / 45
20024		-0.0032	0.0388	-0.0292	0.4336	41 / 41
20025		0.0125	0.0466	-0.0206	0.3517	42 / 42
20026		0.0121	0.0366	-0.0182	0.4052	43 / 43
20027		0.0064	0.0715	-0.0185	0.3874	40 / 40
20028		-0.0042	0.0120	-0.0014	0.4081	41 / 41
20029		0.0155	-0.0153	-0.0131	0.7744	41 / 41
20031		-0.0096	0.0228	0.0012	0.4395	45 / 45
25000		0.0166	0.0111	-0.0174	0.2531	40 / 40
25001		0.0046	0.0088	0.0173	0.3054	40 / 40

25003		0.0043	-0.0180	0.0123	0.3702	40 / 40
25004		0.0055	0.0019	-0.0104	0.3427	33 / 33
25005		0.0070	-0.0102	0.0074	0.3015	39 / 39
25006		-0.0309	-0.0429	-0.0381	0.4639	38 / 38
25008		-0.0300	-0.0012	-0.0313	0.2307	38 / 38
25009		0.0291	-0.0510	-0.0396	0.2575	42 / 42
25010		0.0065	-0.0191	-0.0442	0.2618	41 / 41
25013		-0.0020	0.0155	-0.0283	0.3988	38 / 38
25014		-0.0104	-0.0080	-0.0411	0.3040	37 / 37
25015		-0.0239	-0.0099	-0.0352	0.4327	41 / 41
25016		-0.0245	-0.0088	-0.0314	0.2831	42 / 42
25017		0.0240	-0.0176	0.0419	0.4320	37 / 37
25018		0.0082	-0.0003	0.0286	0.3972	38 / 38
25019		-0.0129	-0.0103	0.0187	0.4786	39 / 39
25020		-0.0087	0.0062	-0.0119	0.3722	33 / 33
25021		0.0185	0.0252	0.0042	0.3656	36 / 36
25022		0.0159	0.0062	0.0181	0.4867	34 / 34
25023		-0.0035	0.0249	0.0013	0.4241	40 / 40
25024		0.0020	-0.0131	-0.0059	0.3478	40 / 40
25029		0.0112	-0.0054	0.0045	0.3334	42 / 42
25030		-0.0081	-0.0099	0.0133	0.3979	44 / 44
25032		-0.0724	-0.0253	-0.0289	0.4236	36 / 36
25033		-0.0214	-0.0198	0.0001	0.3758	41 / 41
25034		-0.0153	-0.0191	-0.0070	0.4026	36 / 36
40000		0.0079	-0.0167	-0.0978	0.3010	42 / 42
40001		0.0215	0.0178	-0.0150	0.2915	43 / 43
40002		0.0177	0.0397	-0.0320	0.3882	42 / 42
40003		0.0100	0.0439	-0.0041	0.3922	39 / 39
40004		0.0051	0.0048	-0.0104	0.2983	37 / 37
40005		0.0188	0.0244	-0.0305	0.2856	29 / 29
45000		0.0348	-0.0283	0.0100	0.4153	44 / 44
45001		-0.0038	0.0038	0.0043	0.4082	34 / 34
45002		0.0134	-0.0326	0.0051	0.4960	41 / 41
10043		-0.0141	-0.0069	0.0359	0.6464	35 / 35
10084		-0.0098	-0.0038	0.0661	0.5581	30 / 30
20015		0.0004	-0.0102	-0.0416	0.3293	45 / 45
20007		-0.0011	-0.0082	-0.0109	0.3915	24 / 24
25027		-0.0005	-0.0093	-0.0370	0.3163	43 / 43
k9		-0.0039	-0.0145	-0.0301	0.5537	42 / 42
6600		-0.0010	-0.0193	-0.0385	0.4095	41 / 41
6602		0.0014	0.0286	-0.0284	0.3828	37 / 37
6603		-0.0038	-0.0200	-0.0031	0.4393	40 / 40
6605		0.0110	0.0173	0.0083	0.4952	40 / 40
6606		0.0167	0.0290	0.0069	0.5743	25 / 25
6607		0.0011	0.0112	-0.0396	0.7231	35 / 35
6608		-0.0062	-0.0168	0.0047	0.6749	21 / 21
6609		-0.0149	0.0041	0.0014	0.3856	42 / 42
6610		0.0037	0.0037	0.0210	0.7437	33 / 33
6611		-0.0123	-0.0249	-0.0611	0.5629	40 / 40
6612		-0.0044	-0.0556	-0.0709	0.7125	38 / 38
6616		-0.0053	-0.0377	-0.0430	0.4981	33 / 33
6617		-0.0067	-0.0047	0.0227	0.5941	23 / 23
6618		-0.0063	-0.0028	-0.0122	0.5127	40 / 40
6619		0.0206	0.0007	0.0159	0.2794	41 / 41
6620		0.0182	-0.0453	-0.0042	0.4070	38 / 38
6621		-0.0218	-0.0089	0.0085	0.2315	41 / 41
6622		0.0056	0.0084	-0.0155	0.5966	37 / 37
6623		0.0016	-0.0146	-0.0315	0.7773	40 / 40
6624		0.0518	-0.0033	-0.0137	0.7110	33 / 33

6625		-0.0497	-0.0260	-0.0278	0.5842	37 / 37
6626		-0.0187	-0.0089	-0.0613	0.4227	35 / 35
6627		-0.0289	-0.0243	-0.0254	0.5997	33 / 33
6628		-0.0350	-0.0321	-0.0477	0.5067	39 / 39
6629		-0.0483	-0.0285	-0.0451	0.4746	37 / 37
6630		-0.0572	-0.0128	-0.0347	0.4028	34 / 34
6631		-0.0317	-0.0293	-0.0386	0.5762	32 / 32
6632		-0.0466	-0.0202	-0.0179	0.6045	34 / 34
6633		-0.0096	-0.0190	-0.0303	0.4359	38 / 38
6634		-0.0158	0.0011	-0.0285	0.4083	35 / 35
6635		-0.0030	0.0026	-0.0282	0.4707	39 / 39
6636		-0.0459	-0.0311	-0.0410	0.5373	41 / 41
6637		-0.0073	-0.0095	-0.0224	0.6340	31 / 31
6638		-0.0260	-0.0096	-0.0283	0.4520	27 / 27
6639		-0.0524	-0.0034	-0.0357	0.5538	38 / 38
6640		-0.0382	-0.0213	-0.0398	0.5107	34 / 34
6641		-0.0472	-0.0154	-0.0378	0.5691	37 / 37
**Mean [m]**		-0.002553	-0.001547	-0.003204		
**Sigma [m]**		0.022400	0.025943	0.035699		
**RMS Error [m]**		0.022545	0.025989	0.035842		

Localisation accuracy per GCP and mean errors in the three coordinate directions. The last column counts the number of calibrated images where the GCP has been automatically verified vs. manually marked.

Absolute Geolocation Variance

Min Error [m]	Max Error [m]	Geolocation Error X [%]	Geolocation Error Y [%]	Geolocation Error Z [%]
-	-15.00	0.00	0.23	0.00
-15.00	-12.00	0.00	0.00	0.00
-12.00	-9.00	0.00	0.00	0.00
-9.00	-6.00	0.00	0.00	0.00
-6.00	-3.00	0.23	5.24	7.69
-3.00	0.00	47.32	47.67	39.86
0.00	3.00	52.33	37.65	45.57
3.00	6.00	0.12	8.97	6.88
6.00	9.00	0.00	0.00	0.00
9.00	12.00	0.00	0.00	0.00
12.00	15.00	0.00	0.00	0.00
15.00	-	0.00	0.23	0.00
**Mean [m]**		0.860815	1.668171	32.649788
**Sigma [m]**		1.127527	2.666174	2.145108
**RMS Error [m]**		1.418562	3.145040	32.720179

Min Error and Max Error represent geolocation error intervals between -1.5 and 1.5 times the maximum accuracy of all the images. Columns X, Y, Z show the percentage of images with geolocation errors within the predefined error intervals. The geolocation error is the difference between the initial and computed image positions. Note that the image geolocation errors do not correspond to the accuracy of the observed 3D points.

Geolocation Bias	X	Y	Z
Translation [m]	0.860820	1.668165	32.649788

Bias between image initial and computed geolocation given in output coordinate system.

Relative Geolocation Variance

Relative Geolocation Error	Images X [%]	Images Y [%]	Images Z [%]
[-1.00, 1.00]	100.00	98.72	100.00
[-2.00, 2.00]	100.00	99.53	100.00

[-3.00, 3.00]	100.00	99.53	100.00
**Mean of Geolocation Accuracy [m]**	5.000000	5.000000	10.000000
**Sigma of Geolocation Accuracy [m]**	0.000000	0.000000	0.000000

Images X, Y, Z represent the percentage of images with a relative geolocation error in X, Y, Z.

Initial Processing Details

System Information

Hardware	CPU: Intel(R) Core(TM) i9-7940X CPU @ 3.10GHz<br>RAM: 128GB<br>GPU: NVIDIA GeForce GTX 1080 Ti (Driver: 22.21.13.8541), RDPDD Chained DD (Driver: unknown), RDP Encoder Mirror Driver (Driver: unknown), RDP Reflector Display Driver (Driver: unknown)
Operating System	Windows 7 Ultimate, 64-bit

Coordinate Systems

Image Coordinate System	WGS 84 (EGM 96 Geoid)
Ground Control Point (GCP) Coordinate System	ETRS89 / Poland CS2000 zone 6 (EGM 2008 Geoid)
Output Coordinate System	ETRS89 / Poland CS2000 zone 6 (EGM 2008 Geoid)

Processing Options

Detected Template	3D Maps
Keypoints Image Scale	Full, Image Scale: 1
Advanced: Matching Image Pairs	Aerial Grid or Corridor
Advanced: Matching Strategy	Use Geometrically Verified Matching: no
Advanced: Keypoint Extraction	Targeted Number of Keypoints: Automatic
Advanced: Calibration	Calibration Method: Standard<br>Internal Parameters Optimization: All<br>External Parameters Optimization: All<br>Rematch: Auto, no

Point Cloud Densification details

Processing Options

Image Scale	multiscale, 1 (Original image size, Slow)
Point Density	Optimal
Minimum Number of Matches	3
3D Textured Mesh Generation	yes
3D Textured Mesh Settings:	Resolution: Medium Resolution (default)<br>Color Balancing: no
LOD	Generated: no
Advanced: 3D Textured Mesh Settings	Sample Density Divider: 1
Advanced: Image Groups	group1
Advanced: Use Processing Area	yes
Advanced: Use Annotations	yes
Time for Point Cloud Densification	04h:22m:08s
Time for Point Cloud Classification	NA
Time for 3D Textured Mesh Generation	26m:21s

Results

Number of Generated Tiles	10
Number of 3D Densified Points	271393007
Average Density (per m^3)	198.87

DSM, Orthomosaic and Index Details

Processing Options

DSM and Orthomosaic Resolution	1 x GSD (4.59 [cm/pixel])
DSM Filters	Noise Filtering: yes<br>Surface Smoothing: yes, Type: Sharp
Raster DSM	Generated: yes<br>Method: Inverse Distance Weighting<br>Merge Tiles: yes
Orthomosaic	Generated: yes<br>Merge Tiles: yes<br>GeoTIFF Without Transparency: yes<br>Google Maps Tiles and KML: no
Time for DSM Generation	01h:11m:47s
Time for Orthomosaic Generation	32m:09s
Time for DTM Generation	00s
Time for Contour Lines Generation	00s
Time for Reflectance Map Generation	00s
Time for Index Map Generation	00s
