# Supplementary material for: Multi-Variant Accuracy Evaluation of UAV Imaging Surveys: A Case Study on Investment Area
Source: Sensors (Basel). 2019 Nov 28;19(23):5229. doi: 10.3390/s19235229 (PMC6929115; doi:10.3390/s19235229)
Supplement: Supplementary file 1 [file sensors-19-05229-s001.zip › supplementary_files/App7_V6_report_PS.pdf]

Agisoft Metashape

Processing Report

01 March 2019

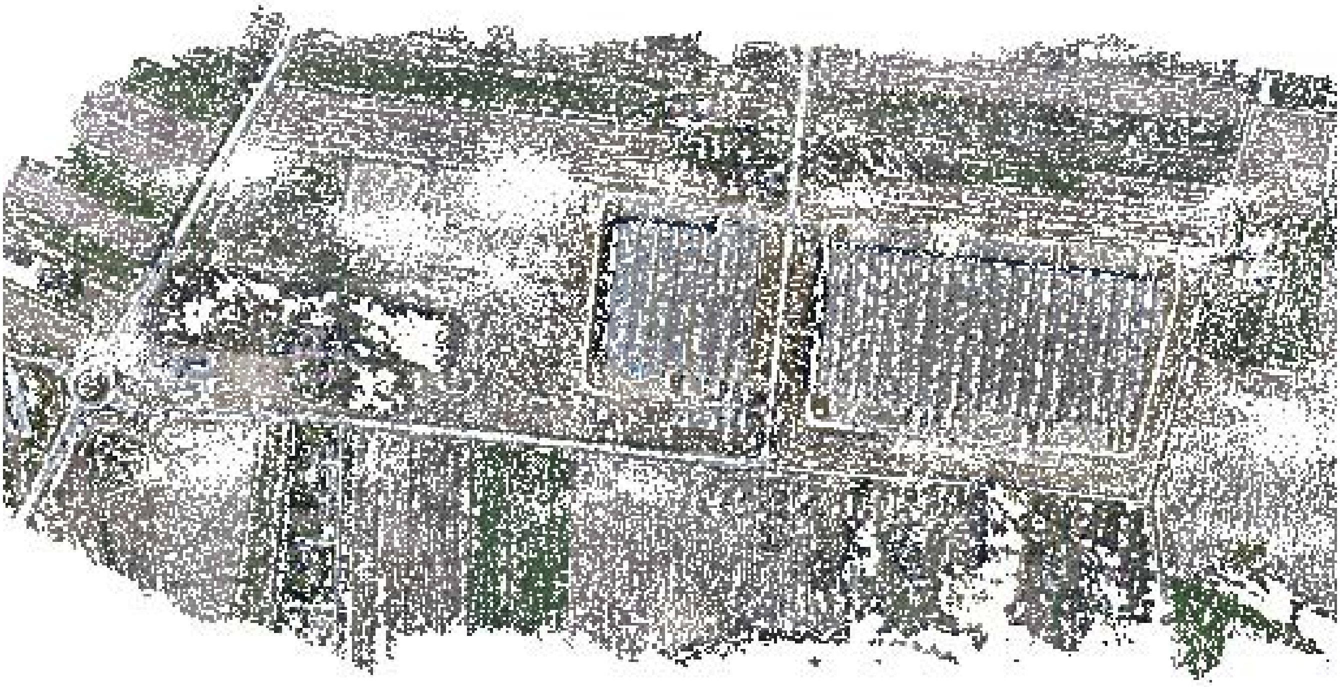

Survey Data

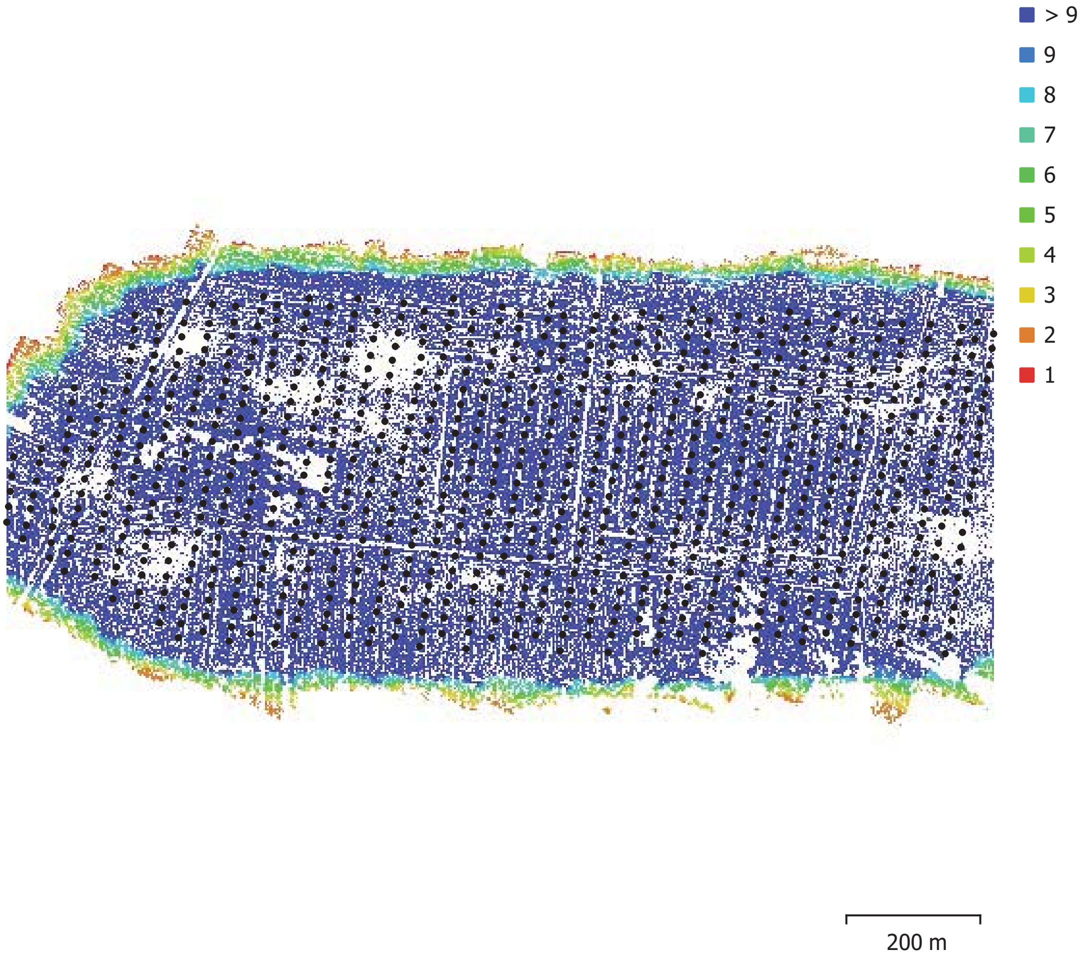

Fig. 1. Camera locations and image overlap.

Number of images:	858	Camera stations:	858
Flying altitude:	161 m	Tie points:	204,589
Ground resolution:	4.96 cm/pix	Projections:	2,672,777
Coverage area:	0.578 km²	Reprojection error:	0.851 pix

Camera Model	Resolution	Focal Length	Pixel Size	Precalibrated
NEX-5T (15.5mm)	4912 x 3264	15.5 mm	4.78 x 4.78 μm	No

Table 1. Cameras.

Camera Calibration

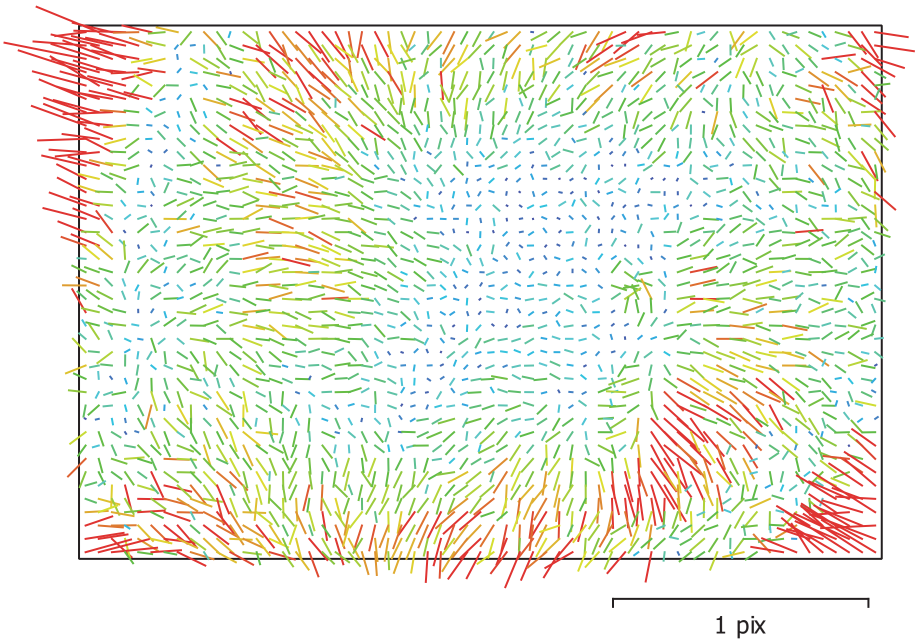

Fig. 2. Image residuals for NEX-5T (15.5mm).

NEX-5T (15.5mm)

858 images

Type	Resolution	Focal Length	Pixel Size
**Frame**	**4912 x 3264**	**15.5 mm**	**4.78 x 4.78 µm**

	Value	Error	F	Cx	Cy	B1	B2	K1	K2	K3	P1	P2
**F**	**3251.07**	0.11	1.00	0.12	-0.39	0.05	0.06	-0.27	0.16	-0.09	0.02	0.11
**Cx**	**9.53833**	0.016		1.00	-0.05	0.02	0.06	-0.03	0.02	-0.01	0.33	0.00
**Cy**	**-44.5403**	0.017			1.00	-0.07	-0.02	0.10	-0.06	0.03	-0.02	0.04
**B1**	**0.131279**	0.0034				1.00	0.00	0.05	-0.07	0.08	0.02	0.03
**B2**	**0.0601348**	0.0032					1.00	-0.01	0.01	-0.00	-0.01	0.01
**K1**	**-0.0468564**	1.4e-005						1.00	-0.96	0.91	0.00	-0.04
**K2**	**0.0352007**	3.7e-005							1.00	-0.98	-0.00	0.02
**K3**	**-0.00960072**	3.1e-005								1.00	0.01	-0.01
**P1**	**0.00033511**	8.4e-007									1.00	-0.02
**P2**	**0.00048511**	5.9e-007										1.00

Table 2. Calibration coefficients and correlation matrix.

Ground Control Points

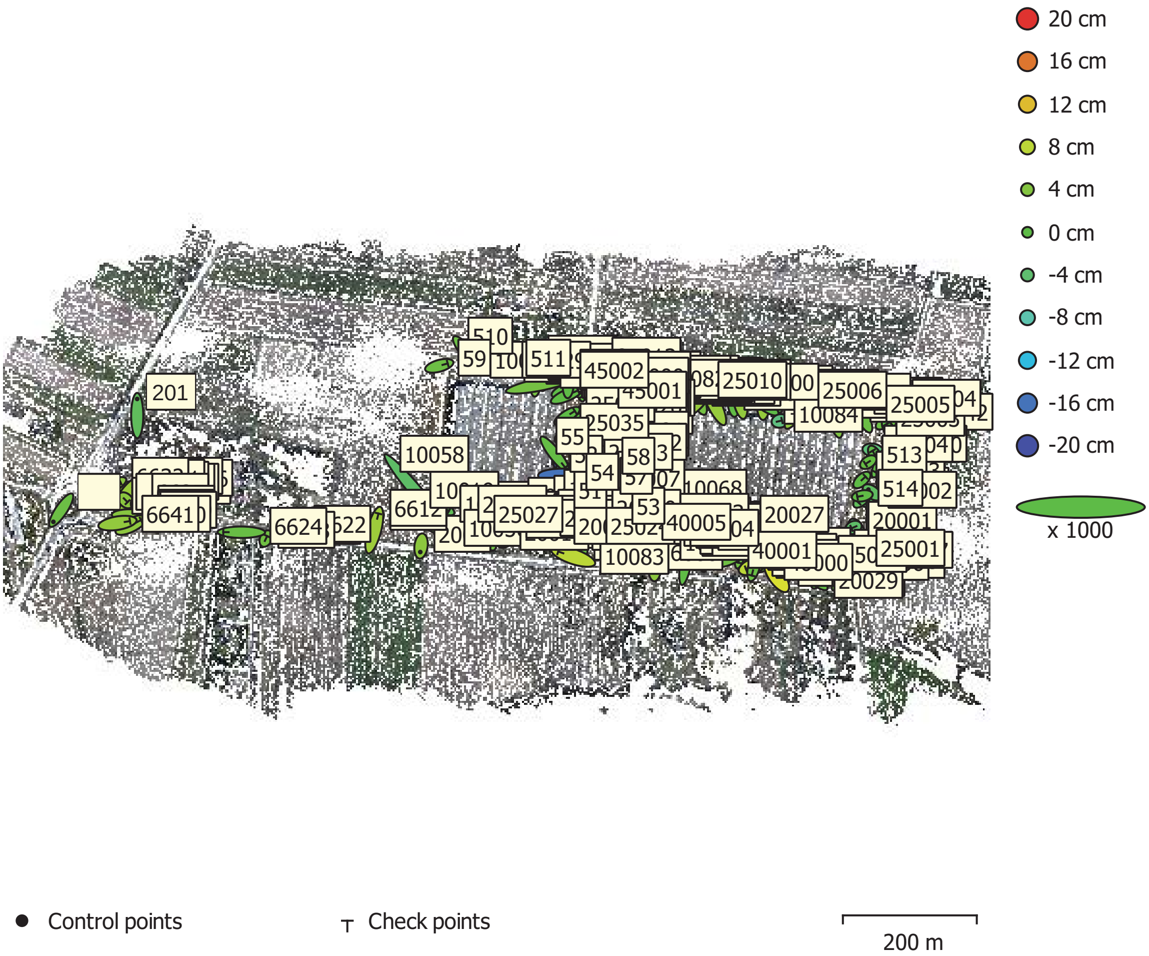

Fig. 3. GCP locations and error estimates.
Z error is represented by ellipse color. X,Y errors are represented by ellipse shape.
Estimated GCP locations are marked with a dot or crossing.

Count	X error (cm)	Y error (cm)	Z error (cm)	XY error (cm)	Total (cm)
11	1.92786	2.67572	3.44739	3.2979	4.77081

Table 3. Control points RMSE.
X - Easting, Y - Northing, Z - Altitude.

Count	X error (cm)	Y error (cm)	Z error (cm)	XY error (cm)	Total (cm)
207	2.45889	2.42462	3.98856	3.45325	5.27575

Table 4. Check points RMSE.
X - Easting, Y - Northing, Z - Altitude.

Label	X error (cm)	Y error (cm)	Z error (cm)	Total (cm)	Image (pix)
205	2.42415	2.61527	-0.664678	3.62739	1.004 (40)
204	-0.228777	-1.7937	4.82068	5.14866	0.958 (39)
208	1.21143	1.50393	1.31084	2.33403	0.744 (38)
209	-2.44278	2.06105	5.88276	6.69492	0.659 (37)
207	2.66441	1.46248	-6.39343	7.07911	0.529 (35)
211	-2.83165	-2.02661	-0.462688	3.51276	0.556 (44)
210	-1.11936	0.828232	-3.0928	3.3918	0.351 (43)
206	0.350534	-3.25589	-2.45568	4.09317	0.409 (35)
201	-0.0121607	4.95478	-2.76517	5.67417	0.856 (34)
202	-2.33929	-3.46564	1.80462	4.55407	0.648 (31)
203	2.3567	-2.84504	1.77062	4.09675	0.789 (39)
**Total**	**1.92786**	**2.67572**	**3.44739**	**4.77081**	**0.711**

Table 5. Control points.
X - Easting, Y - Northing, Z - Altitude.

Label	X error (cm)	Y error (cm)	Z error (cm)	Total (cm)	Image (pix)
103	-0.692711	-1.47297	1.56479	2.25789	0.487 (42)
101	-0.613046	-0.341611	-3.28506	3.35919	0.419 (38)
100	-0.0765699	2.91179	-1.8885	3.47143	0.608 (36)
104	4.55742	0.140874	-0.704322	4.61367	0.431 (39)
102	2.84654	1.04321	-0.992625	3.19004	0.435 (43)
6600	0.0888369	2.36448	4.81348	5.3636	0.528 (41)
6602	0.541977	-2.80862	3.45903	4.48854	0.460 (37)
6603	1.10552	2.0208	0.962832	2.49657	0.598 (40)
6605	-0.216019	-2.01815	-0.454979	2.08005	0.588 (40)
6606	-0.843656	-3.25484	-1.94826	3.88606	0.699 (25)
6607	0.700514	-1.27242	5.68218	5.86489	0.830 (35)
6608	1.66211	1.04468	0.452813	2.0147	0.821 (21)
6609	2.31252	-1.06305	1.37979	2.89511	0.478 (42)
6610	0.520577	-1.18896	-1.81143	2.22843	0.887 (33)
6611	1.9768	1.93819	5.18671	5.87931	0.596 (40)

Label	X error (cm)	Y error (cm)	Z error (cm)	Total (cm)	Image (pix)
6612	1.26914	4.94265	6.0436	7.90985	0.722 (38)
6616	0.140689	3.39831	5.38241	6.36699	0.539 (33)
6617	1.98514	-0.0114083	-2.76521	3.40402	0.714 (23)
6618	1.93741	-0.237768	0.713486	2.07826	0.610 (40)
6619	-0.697442	-0.68606	-1.92552	2.1598	0.347 (41)
6620	-0.447039	3.68681	0.385478	3.73376	0.507 (38)
6621	4.03399	-0.25027	-1.09866	4.1884	0.373 (41)
6622	0.204049	-1.56593	1.53816	2.20448	0.714 (37)
6623	0.352954	0.740346	2.26638	2.41022	0.794 (40)
6624	-5.39847	0.029155	1.40673	5.57882	0.881 (33)
6625	4.65482	2.22822	3.20611	6.07548	0.574 (37)
6626	1.684	0.556208	6.3934	6.63481	0.492 (35)
6627	2.52818	2.19167	2.70223	4.30083	0.735 (33)
6628	2.98758	2.94077	5.41143	6.84524	0.615 (39)
6629	4.24094	2.62604	5.44353	7.38333	0.624 (37)
6630	4.96949	1.14442	4.72559	6.95246	0.565 (34)
6631	2.25068	2.82996	5.73217	6.77731	0.744 (32)
6632	3.57943	1.78512	4.52917	6.04255	0.812 (34)
6633	-0.112712	1.3632	5.23934	5.41495	0.639 (38)
6634	0.536739	-0.549063	4.67629	4.73891	0.489 (35)
6635	-0.61656	-0.691869	4.0046	4.11043	0.573 (39)
6636	3.81022	2.58925	5.07154	6.85146	0.618 (41)
6637	-0.0432379	0.406127	3.02805	3.05547	0.754 (31)
6638	1.84315	0.249347	4.00735	4.41794	0.519 (27)
6639	4.46651	-0.231893	4.33715	6.23012	0.642 (38)
6640	2.87365	1.45847	4.94614	5.90333	0.571 (34)
6641	3.73034	0.612514	5.22141	6.44621	0.569 (37)
10000	1.11541	1.44647	-4.7362	5.07622	0.755 (32)
10001	-1.16328	0.18102	-4.61477	4.76257	0.611 (35)
10002	-1.29452	2.65057	1.67301	3.3912	0.655 (37)
10003	-0.85112	3.77123	3.61823	5.29511	0.671 (40)
10004	-2.28501	1.22401	-0.819847	2.71876	0.584 (39)

Label	X error (cm)	Y error (cm)	Z error (cm)	Total (cm)	Image (pix)
10005	-2.87654	2.00466	-0.413968	3.53051	0.713 (43)
10006	-0.251952	2.10883	0.59053	2.2044	0.574 (42)
10007	-0.129859	1.53268	1.55822	2.18953	0.665 (41)
10008	-1.22241	2.61638	2.65642	3.92381	0.592 (38)
10009	-0.250018	2.38669	1.30815	2.73314	0.625 (42)
10010	-0.161041	2.39785	2.41509	3.40709	0.622 (44)
10011	-0.000143004	3.53272	2.14849	4.13475	0.739 (33)
10012	4.72677	-1.07252	-1.29033	5.01574	0.686 (40)
10013	3.30428	-0.431743	-0.917109	3.45627	0.500 (38)
10014	0.760496	-1.50183	-2.25278	2.81227	0.522 (42)
10015	-0.267562	-0.984021	-3.46738	3.61423	0.566 (40)
10016	4.11792	-0.91957	-3.05579	5.20968	0.673 (43)
10017	1.60718	0.336109	2.12482	2.6853	0.690 (42)
10018	1.98085	-0.305926	-0.362524	2.03686	0.453 (22)
10019	1.95205	-2.20574	-5.80745	6.5117	0.671 (42)
10020	3.14008	-1.78102	-2.53158	4.4092	0.571 (33)
10021	0.95295	-0.558827	-3.13404	3.32304	0.552 (45)
10022	-0.907202	-3.34721	0.242368	3.47643	0.660 (42)
10023	-1.43367	0.239709	1.06086	1.79953	0.543 (38)
10024	1.70177	-0.44231	3.50833	3.92429	0.512 (46)
10025	0.0427298	-0.4453	0.82443	0.937978	0.469 (44)
10026	1.6434	-2.64557	1.19552	3.33603	0.549 (36)
10027	2.1431	-2.10148	1.73315	3.46597	0.660 (41)
10028	0.953465	-0.62616	0.530123	1.25786	0.565 (43)
10030	1.47905	-3.07269	1.96264	3.93459	0.500 (37)
10031	-2.76469	-2.6845	-0.130945	3.8558	0.597 (27)
10032	-3.87172	-3.33215	-1.11951	5.22941	0.588 (27)
10033	-0.87966	-2.72763	-1.22767	3.11784	0.531 (40)
10034	-3.23853	1.10179	0.448598	3.45011	0.491 (38)
10035	-1.98483	2.80204	-4.81387	5.91307	0.513 (35)
10036	-2.27528	0.864681	-0.289851	2.45125	0.657 (27)
10037	-0.923696	1.80056	0.427361	2.0683	0.400 (39)

Label	X error (cm)	Y error (cm)	Z error (cm)	Total (cm)	Image (pix)
10038	-0.731853	-0.105489	0.516772	0.902103	0.429 (42)
10039	-2.09438	1.40596	1.37589	2.87336	0.488 (43)
10040	1.54998	1.10762	1.78904	2.61341	0.770 (31)
10041	0.549313	2.39546	1.83724	3.06846	0.973 (35)
10043	0.730747	0.420492	-1.87348	2.05444	0.844 (35)
10044	-1.59756	4.0983	4.06733	5.99094	0.684 (38)
10045	-2.3613	3.66948	6.49759	7.82684	0.435 (36)
10046	2.67476	0.556012	6.82024	7.34706	0.514 (38)
10047	3.67984	0.420072	-8.85847	9.60157	0.885 (31)
10048	2.28871	2.08148	-5.87409	6.63895	0.751 (32)
10049	3.04281	-2.99186	-5.39745	6.88058	1.008 (32)
10051	0.995518	-2.22528	-0.352234	2.46313	0.485 (44)
10052	1.06044	-0.581356	-3.27777	3.49374	0.591 (35)
10053	2.43976	-1.86772	-1.7107	3.51672	0.508 (40)
10054	2.27336	1.02778	0.0791628	2.49615	0.537 (38)
10055	0.780817	-1.80217	2.24939	2.98618	0.609 (39)
10056	4.53766	-1.78871	0.157469	4.88002	0.587 (38)
10058	7.96927	-9.446	-3.43546	12.8273	0.606 (44)
10059	1.64102	1.88085	5.86346	6.37265	0.491 (40)
10060	4.36201	1.61573	2.16397	5.13035	0.450 (43)
10061	0.949982	-3.52546	-4.67429	5.9313	0.594 (29)
10062	2.05821	1.4325	0.129005	2.51096	0.586 (41)
10063	0.149077	3.47483	2.69951	4.40273	0.645 (38)
10064	-3.04473	-1.78354	4.56749	5.77178	0.593 (43)
10065	-2.54208	-2.38071	2.73174	4.42633	0.453 (44)
10066	-5.29414	-2.50578	3.53295	6.84022	0.538 (41)
10067	-2.78405	-2.68832	2.52873	4.62304	0.473 (42)
10068	-1.02022	-9.71141	3.29662	10.3063	0.567 (43)
10074	2.24456	-4.19425	4.52304	6.56412	0.802 (41)
10075	1.3042	-3.98712	-0.226841	4.20113	0.550 (41)
10076	-0.264857	-1.65622	1.38295	2.17388	0.464 (40)
10077	1.66106	-4.8066	2.06459	5.48863	0.911 (38)

Label	X error (cm)	Y error (cm)	Z error (cm)	Total (cm)	Image (pix)
10078	-3.16263	-0.284787	1.56275	3.53914	0.455 (39)
10080	-0.687364	5.81658	9.10965	10.8301	0.766 (35)
10081	3.22419	2.17589	-0.0478921	3.89001	0.446 (37)
10082	-0.16935	3.56751	3.17391	4.77802	0.529 (39)
10083	-4.4524	1.49838	7.95102	9.23514	0.679 (37)
10084	0.557528	0.12843	-5.00768	5.04026	0.559 (30)
10085	-1.77653	2.50468	-0.566867	3.12263	0.448 (44)
15000	-0.729751	3.66071	5.25276	6.44398	0.566 (46)
15001	2.91685	3.74726	5.49916	7.26572	0.530 (39)
15002	-1.90867	-0.725306	7.97975	8.23684	0.509 (41)
15003	-0.647899	-2.48915	1.57904	3.01811	0.597 (45)
15004	-0.675583	-0.575769	2.22475	2.3953	0.477 (43)
15005	-0.883293	-1.45465	0.717033	1.84672	0.698 (38)
15006	-1.92623	-2.50751	1.3015	3.41934	0.521 (43)
15007	-2.69968	0.989095	-1.95909	3.47917	0.560 (38)
15008	0.69681	-1.25485	-5.3845	5.57252	0.970 (38)
15009	-0.371117	-0.621951	-3.68339	3.75392	0.643 (40)
15010	-1.82179	-3.34887	-1.33911	4.04067	0.664 (38)
20000	-0.209849	-1.26349	-2.2545	2.59291	0.861 (42)
20001	-0.600511	-0.257509	-3.5446	3.60432	0.547 (38)
20002	-1.56819	-1.61104	-2.72489	3.53266	0.502 (38)
20003	1.07654	0.996703	-1.10829	1.83865	0.549 (39)
20004	-0.826348	1.40694	-0.691291	1.77206	0.462 (36)
20005	5.86894	2.32418	-0.530224	6.33462	0.700 (35)
20007	0.944553	0.568922	0.44799	1.19019	0.480 (24)
20008	1.57678	-0.700726	-0.503253	1.79737	0.521 (44)
20009	0.482207	-0.225106	1.37343	1.47293	0.486 (43)
20010	3.24143	-3.06019	2.17354	4.95943	0.529 (45)
20011	2.75089	-0.418448	0.898458	2.92399	0.511 (47)
20012	0.255963	-0.348414	2.27016	2.31096	0.478 (44)
20013	-0.0464143	-0.272521	0.443396	0.522515	0.452 (41)
20014	1.02079	-1.09156	1.72991	2.28607	0.455 (43)

Label	X error (cm)	Y error (cm)	Z error (cm)	Total (cm)	Image (pix)
20015	0.830353	0.516278	5.56341	5.64867	0.414 (45)
20016	0.794946	-2.00749	5.24945	5.67615	0.439 (46)
20017	-0.327741	-2.94895	3.67932	4.72663	0.443 (40)
20018	-1.30865	-2.53665	2.9662	4.11649	0.420 (44)
20019	-1.77591	-1.58766	1.81183	2.99286	0.560 (39)
20020	-0.177082	-3.5729	1.12388	3.74968	0.590 (40)
20021	-1.2412	0.353636	-0.741218	1.4883	0.408 (40)
20022	0.983553	5.54316	-0.827816	5.69028	0.473 (42)
20023	-0.760373	0.10435	0.457433	0.893477	0.506 (45)
20024	-0.143571	-3.39474	3.6492	4.98613	0.557 (41)
20025	-1.74375	-4.20445	2.84974	5.3702	0.465 (42)
20026	-1.90067	-3.13733	1.66876	4.02991	0.568 (43)
20027	-1.44406	-6.25638	2.26917	6.81005	0.539 (40)
20028	-0.577156	-0.323792	0.135032	0.675414	0.489 (41)
20029	-2.93258	2.43014	0.997806	3.93716	0.887 (41)
20031	1.8402	-2.94789	0.580823	3.52331	0.513 (45)
25000	-3.1707	-0.253278	1.92182	3.7163	0.325 (40)
25001	-1.99557	0.0194981	-2.76733	3.41186	0.336 (40)
25003	-1.31402	2.2006	-0.372215	2.58994	0.482 (40)
25004	-1.35689	-0.21088	1.99266	2.41998	0.575 (33)
25005	-1.32552	0.821882	0.753617	1.73218	0.490 (39)
25006	2.51516	4.00975	5.02361	6.90224	0.574 (38)
25008	3.59345	-0.347973	4.66008	5.89494	0.558 (38)
25009	-2.30284	4.65662	5.61361	7.64852	0.537 (42)
25010	-0.237525	1.33689	5.74985	5.908	0.465 (41)
25013	1.59264	-2.12332	2.33257	3.53354	0.483 (38)
25014	2.44074	0.211302	3.76691	4.49349	0.408 (37)
25015	3.80549	0.388636	3.1084	4.92899	0.540 (41)
25016	3.84852	0.282811	2.6963	4.70756	0.401 (42)
25017	-1.206	1.45614	-5.72932	6.03323	0.562 (37)
25018	0.186412	-0.33003	-4.50825	4.52416	0.481 (38)
25019	2.31435	0.623992	-3.37514	4.13971	0.589 (39)

Label	X error (cm)	Y error (cm)	Z error (cm)	Total (cm)	Image (pix)
25020	1.90048	-0.91731	-0.378705	2.14399	0.411 (33)
25021	-1.00434	-2.8425	-1.33114	3.29551	0.516 (36)
25022	-0.69378	-0.939017	-2.6509	2.89661	0.573 (34)
25023	1.0754	-2.72376	-0.903518	3.06459	0.548 (40)
25024	0.553459	1.31083	1.35466	1.96461	0.455 (40)
25027	0.882538	0.173107	4.52319	4.61173	0.413 (43)
25029	0.888608	-0.548661	-0.797671	1.31413	0.511 (42)
25030	2.27599	0.0959173	-1.4596	2.7055	0.493 (44)
25032	8.55333	1.69237	2.64425	9.11129	0.522 (36)
25033	3.26839	1.4218	-0.973745	3.69487	0.506 (41)
25034	2.33551	1.49634	-0.310218	2.79104	0.466 (36)
40000	-1.82852	2.5854	9.69013	10.1944	0.425 (42)
40001	-2.90846	-0.927925	2.27325	3.80629	0.462 (43)
40002	-1.81578	-3.58974	4.15987	5.78686	0.507 (42)
40003	-1.14966	-4.15453	1.55178	4.58147	0.527 (39)
40004	-0.872251	-0.275852	2.08233	2.27442	0.414 (37)
40005	-1.68755	-2.37082	4.29959	5.19183	0.425 (29)
45000	-2.0849	2.33264	-1.6192	3.52276	0.547 (44)
45001	1.59794	-0.732331	-1.4949	2.30748	0.542 (34)
45002	0.110989	2.38783	-0.612573	2.46765	0.579 (41)
51	-2.44912	4.50116	-12.3584	13.3787	0.587 (38)
511	3.00156	0.975428	3.68366	4.85079	0.635 (43)
513	-0.865125	-0.557239	1.17858	1.56461	0.500 (29)
514	-1.49179	-0.27234	0.506745	1.59887	0.535 (38)
52	0.633415	-3.20982	-0.696909	3.34512	0.547 (43)
53	-8.18686	6.72417	-19.0917	21.8342	0.666 (26)
54	-1.62537	1.46282	-8.37215	8.65301	0.591 (36)
55	3.07544	-3.93595	0.983366	5.09088	0.524 (38)
56	3.67163	-1.48172	-7.08421	8.11556	0.913 (29)
57	8.60926	-0.792436	-16.0589	18.2383	0.624 (28)
58	4.89795	2.9649	-13.5985	14.7546	0.723 (30)
59	2.51933	0.698928	1.98997	3.28565	0.693 (42)

Label	X error (cm)	Y error (cm)	Z error (cm)	Total (cm)	Image (pix)
Total	2.45889	2.42462	3.98856	5.27575	0.582

Table 6. Check points.

X - Easting, Y - Northing, Z - Altitude.

Digital Elevation Model

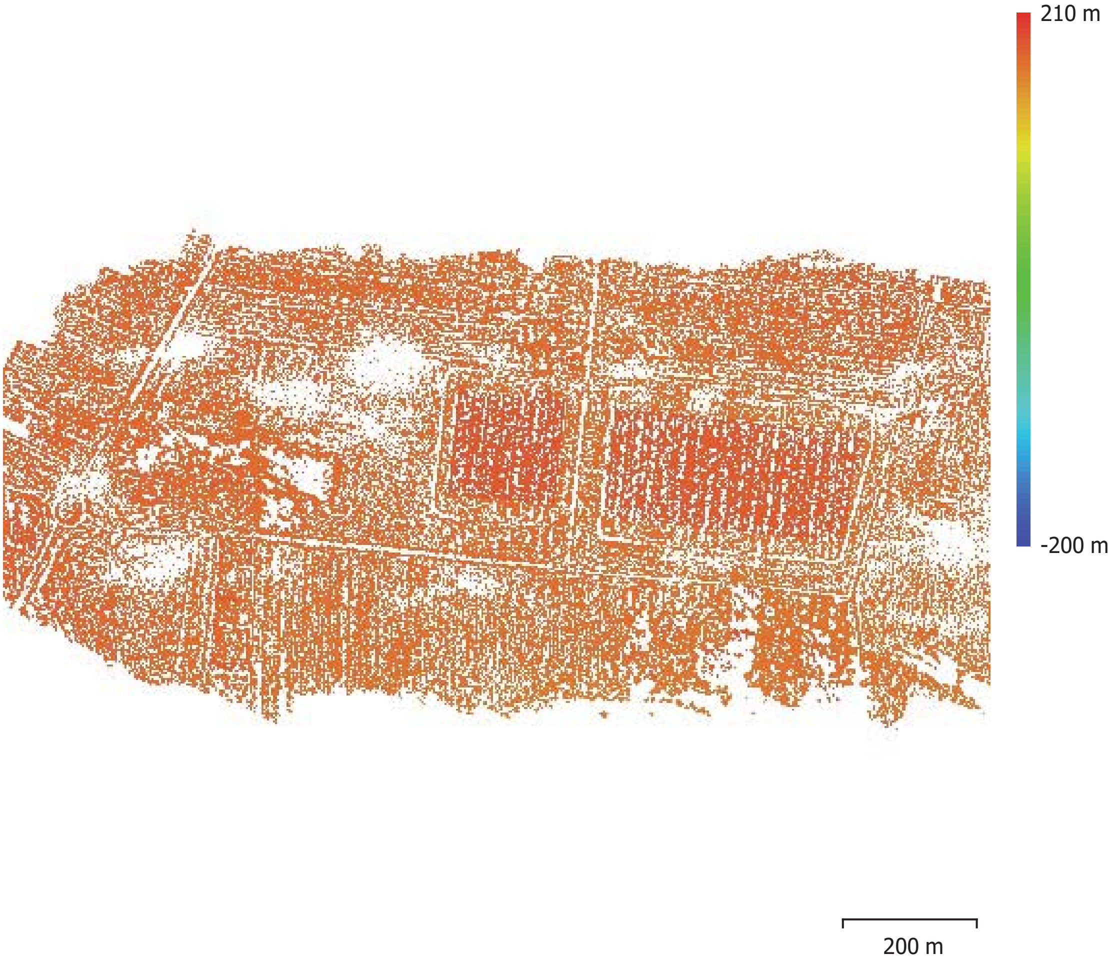

Fig. 4. Reconstructed digital elevation model.

Resolution:	unknown
Point density:	unknown

Processing Parameters

**General**	
Cameras	858
Aligned cameras	858
Markers	218
Coordinate system	ETRS89 / Poland CS2000 zone 6 (EPSG::2177)
Rotation angles	Yaw, Pitch, Roll
**Point Cloud**	
Points	204,589 of 263,610
RMS reprojection error	0.273209 (0.851175 pix)
Max reprojection error	1.43392 (38.9841 pix)
Mean key point size	3.07065 pix
Point colors	3 bands, uint8
Key points	Yes
Average tie point multiplicity	14.956
**Alignment parameters**	
Accuracy	High
Generic preselection	No
Reference preselection	Yes
Key point limit	40,000
Tie point limit	4,000
Adaptive camera model fitting	Yes
Matching time	16 minutes 50 seconds
Alignment time	3 minutes 32 seconds
**Optimization parameters**	
Parameters	f, b1, b2, cx, cy, k1-k3, p1, p2
Adaptive camera model fitting	No
Optimization time	13 seconds
**Software**	
Version	1.5.1 build 7618
Platform	Windows 64
