# Supplementary material for: Multi-Variant Accuracy Evaluation of UAV Imaging Surveys: A Case Study on Investment Area
Source: Sensors (Basel). 2019 Nov 28;19(23):5229. doi: 10.3390/s19235229 (PMC6929115; doi:10.3390/s19235229)
Supplement: Supplementary file 1 [file sensors-19-05229-s001.zip › supplementary_files/App8_V6_p65q45_GPS_report.pdf]

Quality Report

Generated with Pix4Ddiscovery version 4.3.31

Important: Click on the different icons for:

Help to analyze the results in the Quality Report

Additional information about the sections

Click here for additional tips to analyze the Quality Report

Summary

Project	V6_p65q45_GPS
Processed	2019-10-17 00:32:08
Camera Model Name(s)	NEX-5T_0.0_4912x3264 (RGB)
Average Ground Sampling Distance (GSD)	4.57 cm / 1.80 in
Area Covered	0.822 km^2 / 82.1668 ha / 0.32 sq. mi. / 203.1436 acres
Time for Initial Processing (without report)	05m:56s

Quality Check

**Images**	median of 45797 keypoints per image	✓
**Dataset**	138 out of 138 images calibrated (100%), all images enabled	✓
**Camera Optimization**	0.28% relative difference between initial and optimized internal camera parameters	✓
**Matching**	median of 15333.2 matches per calibrated image	✓
**Georeferencing**	yes, 11 GCPs (11 3D), mean RMS error = 0.016 m	✓

Preview

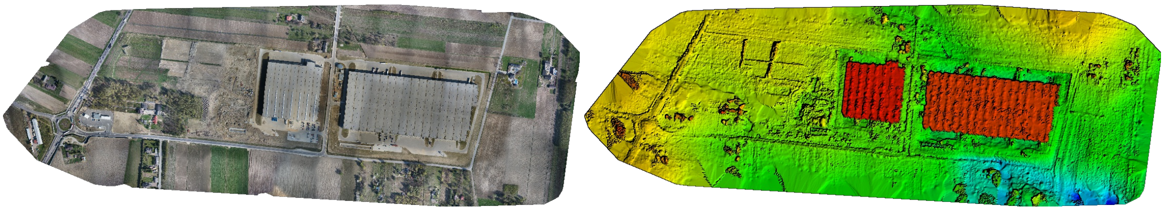

Figure 1: Orthomosaic and the corresponding sparse Digital Surface Model (DSM) before densification.

Calibration Details

Number of Calibrated Images	138 out of 138
Number of Geolocated Images	138 out of 138

Initial Image Positions

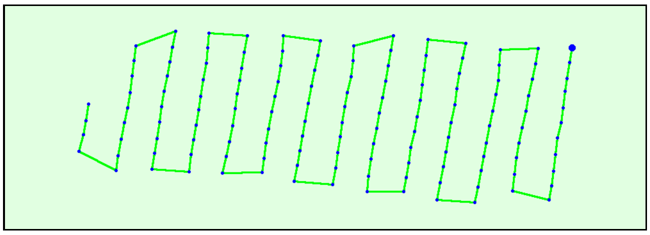

Figure 2: Top view of the initial image position. The green line follows the position of the images in time starting from the large blue dot.

Computed Image/GCPs/Manual Tie Points Positions

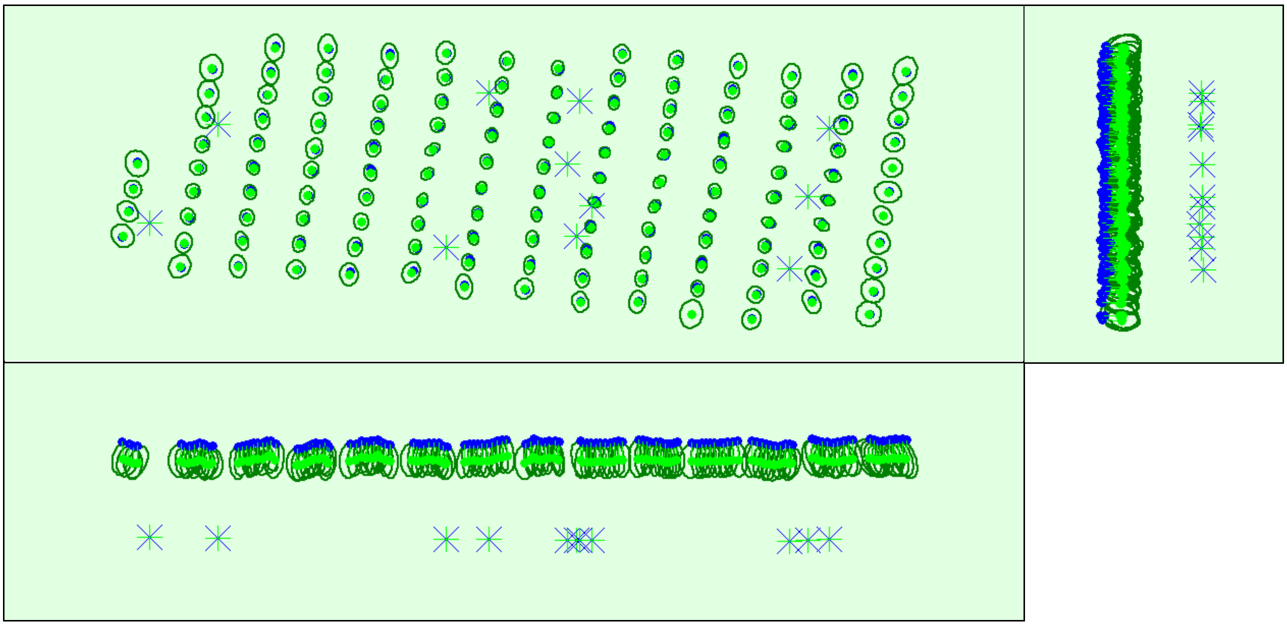

Uncertainty ellipses 1000x magnified

Figure 3: Offset between initial (blue dots) and computed (green dots) image positions as well as the offset between the GCPs initial positions (blue crosses) and their computed positions (green crosses) in the top-view (XY plane), front-view (XZ plane), and side-view (YZ plane). Dark green ellipses indicate the absolute position uncertainty of the bundle block adjustment result.

Absolute camera position and orientation uncertainties

	X [m]	Y [m]	Z [m]	Omega [degree]	Phi [degree]	Kappa [degree]
Mean	0.014	0.016	0.030	0.006	0.005	0.002
Sigma	0.003	0.004	0.001	0.001	0.001	0.001

Overlap

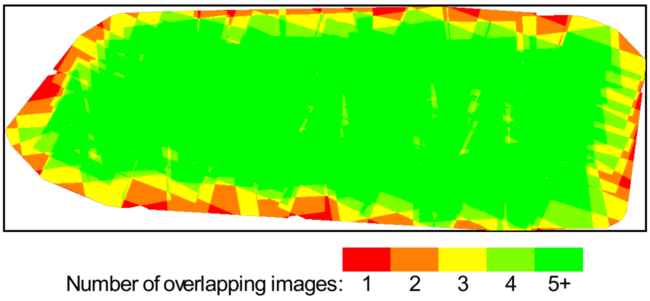

Figure 4: Number of overlapping images computed for each pixel of the orthomosaic.
Red and yellow areas indicate low overlap for which poor results may be generated. Green areas indicate an overlap of over 5 images for every pixel. Good quality results will be generated as long as the number of keypoint matches is also sufficient for these areas (see Figure 5 for keypoint matches).

Bundle Block Adjustment Details

Number of 2D Keypoint Observations for Bundle Block Adjustment	2198445
Number of 3D Points for Bundle Block Adjustment	916451
Mean Reprojection Error [pixels]	0.158

Internal Camera Parameters

NEX-5T_0.0_4912x3264 (RGB). Sensor Dimensions: 23.400 [mm] x 15.549 [mm]

EXIF ID: NEX-5T_0.0_4912x3264

	Focal Length	Principal Point x	Principal Point y	R1	R2	R3	T1	T2
Initial Values	3256.554 [pixel] 15.514 [mm]	2456.002 [pixel] 11.700 [mm]	1632.003 [pixel] 7.775 [mm]	-0.043	0.028	-0.005	0.000	-0.001
Optimized Values	3247.296 [pixel] 15.470 [mm]	2467.335 [pixel] 11.754 [mm]	1589.229 [pixel] 7.571 [mm]	-0.047	0.035	-0.010	0.000	0.000
Uncertainties (Sigma)	0.642 [pixel] 0.003 [mm]	0.129 [pixel] 0.001 [mm]	0.109 [pixel] 0.001 [mm]	0.000	0.000	0.000	0.000	0.000

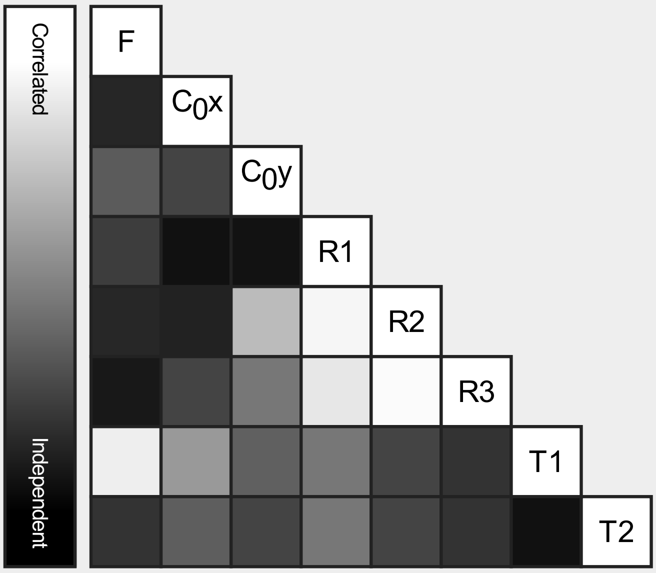

The correlation between camera internal parameters determined by the bundle adjustment. White indicates a full correlation between the parameters, ie. any change in one can be fully compensated by the other. Black indicates that the parameter is completely independent, and is not affected by other parameters.

The number of Automatic Tie Points (ATPs) per pixel, averaged over all images of the camera model, is color coded between black and white. White indicates that, on average, more than 16 ATPs have been extracted at the pixel location. Black indicates that, on average, 0 ATPs have been extracted at the pixel location. Click on the image to the see the average direction and magnitude of the re-projection error for each pixel. Note that the vectors are scaled for better visualization. The scale bar indicates the magnitude of 1 pixel error.

2D Keypoints Table

	Number of 2D Keypoints per Image	Number of Matched 2D Keypoints per Image
Median	45797	15333
Min	20057	4444
Max	68781	32908
Mean	42868	15931

3D Points from 2D Keypoint Matches

	Number of 3D Points Observed
In 2 Images	669050
In 3 Images	173109

In 4 Images	46864
In 5 Images	15782
In 6 Images	7865
In 7 Images	2902
In 8 Images	775
In 9 Images	92
In 10 Images	12

2D Keypoint Matches

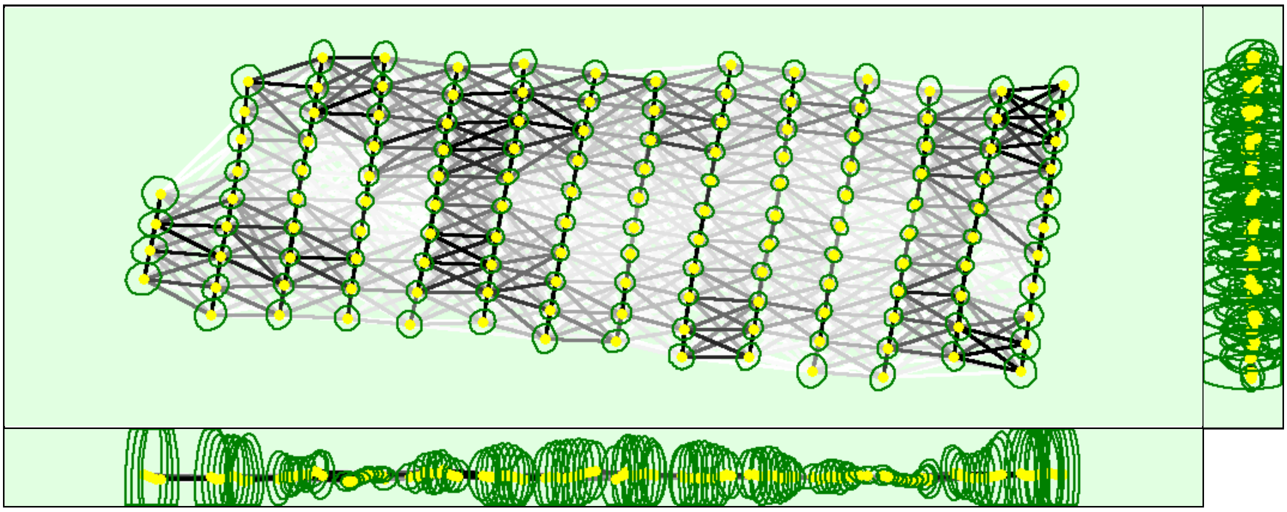

Uncertainty ellipses 1000x magnified

Number of matches 25 222 444 666 888 1111 1333 1555 1777 2000

Figure 5: Computed image positions with links between matched images. The darkness of the links indicates the number of matched 2D keypoints between the images. Bright links indicate weak links and require manual tie points or more images. Dark green ellipses indicate the relative camera position uncertainty of the bundle block adjustment result.

Relative camera position and orientation uncertainties

	X [m]	Y [m]	Z [m]	Omega [degree]	Phi [degree]	Kappa [degree]
Mean	0.017	0.017	0.045	0.010	0.013	0.003
Sigma	0.003	0.004	0.026	0.005	0.006	0.001

Geolocation Details

Ground Control Points

GCP Name	Accuracy XY/Z [m]	Error X [m]	Error Y [m]	Error Z [m]	Projection Error [pixel]	Verified/Marked
201 (3D)	0.030/ 0.050	0.001	-0.008	0.007	0.372	5 / 5
202 (3D)	0.030/ 0.050	0.000	0.004	-0.003	0.689	6 / 6
203 (3D)	0.030/ 0.050	-0.004	0.011	0.004	0.789	7 / 7
208 (3D)	0.030/ 0.050	0.012	-0.008	-0.027	0.470	6 / 6
209 (3D)	0.030/ 0.050	0.012	-0.001	-0.015	0.429	7 / 7
207 (3D)	0.030/ 0.050	-0.027	-0.002	0.059	0.413	6 / 6
211 (3D)	0.030/ 0.050	0.009	0.013	-0.015	0.614	7 / 7
210 (3D)	0.030/ 0.050	-0.005	-0.006	0.029	0.180	6 / 6
206 (3D)	0.030/ 0.050	0.010	0.017	-0.004	0.336	8 / 8
205 (3D)	0.030/ 0.050	-0.015	-0.037	-0.023	0.907	7 / 7
204 (3D)	0.030/ 0.050	-0.005	0.011	-0.007	0.842	6 / 6

**Mean [m]**		-0.001008	-0.000495	0.000500		
**Sigma [m]**		0.011540	0.014310	0.023762		
**RMS Error [m]**		0.011584	0.014319	0.023767		

0 out of 207 check points have been labeled as inaccurate.

Check Point Name	Accuracy XY/Z [m]	Error X [m]	Error Y [m]	Error Z [m]	Projection Error [pixel]	Verified/Marked
103		0.0197	0.0263	-0.0090	0.2026	6 / 6
101		-0.0124	0.0006	0.0094	0.4027	7 / 7
100		-0.0059	-0.0052	0.0517	0.2959	7 / 7
104		-0.0570	-0.0028	0.0549	0.5398	6 / 6
102		-0.0178	-0.0239	0.0031	0.3785	7 / 7
k1		0.0433	-0.0625	0.1254	0.4283	6 / 6
k2		0.0051	0.0218	-0.0315	0.4651	8 / 8
k3		0.0847	-0.0916	0.1461	0.3406	6 / 6
k4		0.0298	-0.0313	0.0689	0.3730	6 / 6
k5		-0.0247	0.0262	-0.0498	0.3971	6 / 6
k6		-0.0295	0.0202	0.0611	0.5403	5 / 5
k7		-0.0968	0.0158	0.1608	0.3752	4 / 4
k8		-0.0502	-0.0320	0.1508	0.4894	5 / 5
k11		-0.0056	-0.0482	-0.1036	0.6386	8 / 8
k13		-0.0072	0.0247	0.0305	0.3029	6 / 6
k14		-0.0026	0.0003	-0.0047	0.6024	6 / 6
10000		-0.0294	-0.0256	0.0138	0.6908	6 / 6
10001		-0.0057	0.0060	0.1136	0.7019	6 / 6
10002		0.0056	-0.0231	0.0264	0.3743	5 / 5
10003		0.0027	-0.0304	-0.0121	0.5968	8 / 8
10004		-0.0002	0.0121	0.0201	0.5004	7 / 7
10005		0.0105	-0.0026	0.0122	0.7097	7 / 7
10006		-0.0069	-0.0125	0.0110	0.4301	7 / 7
10007		0.0176	-0.0072	-0.0328	0.3829	7 / 7
10008		0.0226	-0.0242	-0.0519	0.3072	8 / 8
10009		0.0101	-0.0255	-0.0420	0.4761	7 / 7
10010		0.0169	-0.0381	-0.0489	0.3777	6 / 6
10011		0.0054	-0.0449	-0.0409	0.7477	6 / 6
10012		-0.0574	0.0055	0.0232	0.4523	8 / 8
10013		-0.0303	-0.0083	-0.0055	0.4299	7 / 7
10014		0.0117	0.0056	-0.0092	0.4302	7 / 7
10015		0.0194	-0.0185	0.0132	0.4610	8 / 8
10016		-0.0084	-0.0251	-0.0158	0.4369	7 / 7
10017		0.0090	-0.0393	-0.0604	0.6224	8 / 8
10018		0.0094	-0.0288	-0.0126	0.4375	3 / 3
10019		-0.0417	0.0028	0.0689	0.8055	7 / 7
10020		-0.0600	-0.0108	0.0419	0.6098	7 / 7
10021		-0.0068	-0.0077	-0.0160	0.3187	9 / 9
10022		0.0302	0.0231	-0.0737	0.3759	8 / 8
10023		0.0259	-0.0057	-0.0643	0.3922	7 / 7
10024		-0.0153	0.0000	-0.0967	0.4997	9 / 9
10025		-0.0029	-0.0010	-0.0723	0.4694	8 / 8
10026		-0.0055	0.0179	-0.0636	0.4098	7 / 7
10027		-0.0022	0.0117	-0.0352	0.5124	7 / 7
10028		0.0112	0.0029	-0.0224	0.4674	7 / 7
10030		0.0003	0.0405	-0.0367	0.4262	7 / 7
10031		0.0342	0.0322	-0.0045	0.7101	4 / 4
10032		0.0464	0.0577	0.0081	0.2416	3 / 3
10033		0.0247	0.0526	0.0012	0.3269	7 / 7
10034		0.0327	0.0163	-0.0192	0.4731	7 / 7
10035		0.0259	-0.0098	0.0476	0.4238	6 / 6
10036		0.0294	0.0071	0.0117	0.3399	4 / 4
10037		-0.0219	0.0204	-0.0126	0.2734	6 / 6
10038		-0.0161	0.0315	-0.0047	0.4187	8 / 8

10039		-0.0047	0.0073	-0.0068	0.4519	7 / 7
10040		-0.0207	0.0131	0.0140	0.1985	6 / 6
10041		-0.0199	-0.0004	0.0295	0.6358	8 / 8
10044		0.0039	-0.0396	-0.0106	0.3299	6 / 6
10045		0.0186	-0.0253	-0.0909	0.5682	8 / 8
10046		-0.0284	-0.0175	-0.0874	0.3653	6 / 6
10047		-0.0429	-0.0086	0.0868	0.5566	6 / 6
10048		-0.0326	-0.0364	0.0798	0.7110	7 / 7
10049		-0.0178	0.0286	-0.0166	0.8509	7 / 7
10051		0.0103	0.0168	0.0038	0.3903	7 / 7
10052		0.0020	-0.0067	0.0172	0.5260	7 / 7
10053		-0.0045	-0.0042	-0.0333	0.4997	7 / 7
10054		0.0030	-0.0454	-0.0076	0.5064	7 / 7
10055		-0.0423	-0.0090	-0.0705	0.4489	6 / 6
10056		-0.0788	-0.0134	0.0041	0.5603	7 / 7
10058		-0.1056	0.0647	0.0638	0.7188	8 / 8
10059		-0.0083	-0.0177	-0.0745	0.4266	7 / 7
10060		-0.0184	-0.0365	-0.0159	0.4194	7 / 7
10061		0.0384	-0.0078	-0.0279	0.5061	4 / 4
10062		0.0074	-0.0547	-0.0389	0.3302	9 / 9
10063		0.0276	-0.0699	-0.0672	0.5315	8 / 8
10064		0.0414	0.0363	-0.0635	0.5287	7 / 7
10065		0.0396	0.0562	-0.0380	0.2712	6 / 6
10066		0.0699	0.0626	-0.0699	0.5338	6 / 6
10067		0.0342	0.0617	-0.0731	0.3742	5 / 5
10068		0.0215	0.1318	-0.0599	0.2312	6 / 6
10074		-0.0058	0.0722	-0.0475	0.5643	7 / 7
10075		-0.0006	0.0731	0.0003	0.4104	7 / 7
10076		0.0102	0.0548	-0.0501	0.5186	6 / 6
10077		-0.0252	0.0956	-0.0650	0.6420	6 / 6
10078		-0.0049	0.0521	-0.0359	0.1618	6 / 6
10080		0.0127	-0.0551	-0.1420	0.9353	7 / 7
10081		-0.0348	-0.0291	-0.0301	0.4515	8 / 8
10082		0.0024	-0.0355	-0.0684	0.5139	9 / 9
10083		0.0558	-0.0122	-0.1146	0.2979	6 / 6
10085		0.0503	-0.0570	-0.0174	0.3909	8 / 8
15000		-0.0030	-0.0309	-0.0247	0.3305	7 / 7
15001		-0.0238	-0.0330	-0.0407	0.3631	6 / 6
15002		-0.0154	0.0514	-0.1007	0.2932	6 / 6
15003		-0.0277	0.0676	-0.0287	0.3568	9 / 9
15004		-0.0253	0.0289	-0.0492	0.2340	7 / 7
15005		-0.0246	0.0447	-0.0537	0.8536	6 / 6
15006		-0.0021	0.0340	-0.0321	0.3304	7 / 7
15007		0.0141	-0.0197	-0.0105	0.3567	5 / 5
15008		-0.0331	0.0158	0.0974	1.0818	6 / 6
15009		-0.0087	0.0012	0.0264	0.6398	6 / 6
15010		0.0082	0.0494	0.0604	0.6757	6 / 6
20000		-0.0008	0.0213	0.0318	0.7657	6 / 6
20001		-0.0079	-0.0015	0.0511	0.3503	6 / 6
20002		0.0014	0.0132	0.0366	0.3706	6 / 6
20003		-0.0277	-0.0077	0.0128	0.5328	7 / 7
20004		-0.0079	-0.0134	0.0462	0.4547	6 / 6
20005		-0.0651	-0.0352	0.0260	0.3311	6 / 6
20008		0.0037	-0.0149	-0.0120	0.5506	8 / 8
20009		0.0288	-0.0347	-0.0497	0.3901	8 / 8
20010		-0.0345	0.0164	-0.0790	0.4911	9 / 9
20011		-0.0196	-0.0069	-0.0680	0.3800	10 / 10
20012		0.0165	-0.0083	-0.0789	0.3307	8 / 8
20013		0.0220	-0.0103	-0.0370	0.3842	7 / 7

20014		0.0185	0.0025	-0.0305	0.3081	7 / 7
20016		-0.0081	0.0041	-0.1269	0.2456	8 / 8
20017		0.0007	0.0199	-0.0976	0.3554	6 / 6
20018		0.0228	0.0213	-0.0769	0.3186	7 / 7
20019		0.0329	0.0173	-0.0506	0.2606	7 / 7
20020		0.0198	0.0478	-0.0075	0.5754	7 / 7
20021		0.0159	0.0210	-0.0058	0.1735	6 / 6
20022		-0.0115	-0.0265	0.0017	0.2280	6 / 6
20023		-0.0014	0.0266	-0.0250	0.1621	6 / 6
20024		0.0051	0.0719	-0.0439	0.4304	7 / 7
20025		0.0249	0.0750	-0.0400	0.2699	7 / 7
20026		0.0229	0.0717	-0.0194	0.4770	8 / 8
20027		-0.0337	0.0988	-0.0413	0.4674	5 / 5
20028		-0.0232	0.0376	-0.0174	0.3290	6 / 6
20029		0.0063	-0.0124	-0.0536	0.8621	8 / 8
20031		-0.0473	0.0076	-0.0482	0.4895	6 / 6
25000		0.0048	0.0258	-0.0251	0.3010	8 / 8
25001		0.0025	0.0017	0.0055	0.1982	5 / 5
25003		0.0024	-0.0135	0.0423	0.4455	7 / 7
25004		0.0031	0.0147	0.0071	0.4151	6 / 6
25005		0.0035	-0.0038	0.0471	0.2726	6 / 6
25006		-0.0293	-0.0264	-0.0907	0.5151	7 / 7
25008		-0.0230	0.0093	-0.0448	0.1442	6 / 6
25009		0.0343	-0.0403	-0.0448	0.3054	7 / 7
25010		0.0069	-0.0104	-0.0568	0.2627	7 / 7
25013		-0.0078	0.0160	-0.0325	0.3099	6 / 6
25014		-0.0247	-0.0154	-0.0419	0.2930	6 / 6
25015		-0.0394	-0.0161	-0.0369	0.3476	6 / 6
25016		-0.0332	-0.0152	-0.0422	0.2842	6 / 6
25017		0.0291	-0.0367	0.0308	0.3753	6 / 6
25018		0.0066	-0.0084	0.0326	0.3641	7 / 7
25019		-0.0149	-0.0254	0.0220	0.3004	6 / 6
25020		-0.0139	-0.0061	-0.0480	0.1512	4 / 4
25021		-0.0009	0.0110	0.0090	0.2918	6 / 6
25022		-0.0093	0.0068	0.0373	0.4949	6 / 6
25023		-0.0047	0.0228	-0.0215	0.2270	6 / 6
25024		0.0123	-0.0071	-0.0469	0.2508	6 / 6
25029		0.0195	-0.0302	-0.0414	0.4142	8 / 8
25030		0.0007	-0.0301	0.0161	0.3656	6 / 6
25032		-0.0553	-0.0298	-0.0170	0.4987	6 / 6
25033		-0.0104	-0.0398	-0.0225	0.3743	8 / 8
25034		-0.0020	-0.0323	-0.0063	0.4146	7 / 7
40000		-0.0160	0.0039	-0.0821	0.2607	6 / 6
40001		0.0117	0.0532	-0.0256	0.3721	8 / 8
40002		0.0308	0.0718	-0.0611	0.4138	6 / 6
40003		0.0225	0.0775	-0.0336	0.2091	5 / 5
40004		0.0207	0.0295	-0.0293	0.3362	6 / 6
40005		0.0262	0.0490	-0.0525	0.2377	3 / 3
45000		0.0102	-0.0344	0.0098	0.4098	7 / 7
45001		-0.0218	0.0138	-0.0033	0.1545	4 / 4
45002		0.0371	-0.0545	0.0051	0.6561	6 / 6
10043		-0.0195	0.0019	0.0340	0.9102	6 / 6
10084		-0.0237	-0.0042	-0.0243	0.5346	4 / 4
20015		-0.0230	-0.0292	-0.1127	0.2156	6 / 6
20007		-0.0063	-0.0024	-0.0421	0.3670	4 / 4
25027		-0.0412	-0.0252	-0.0752	0.3284	7 / 7
k9		0.0079	-0.0288	-0.0213	0.5989	7 / 7
6600		0.0113	0.0171	-0.0905	0.3745	6 / 6
6602		0.0127	0.0364	-0.0333	0.4344	7 / 7

6603		-0.0026	-0.0032	-0.0192	0.3484	6 / 6
6605		0.0147	0.0217	-0.0103	0.4988	6 / 6
6606		0.0036	0.0089	-0.0313	0.5807	4 / 4
6607		0.0253	0.0167	-0.0713	0.2607	6 / 6
6608		-0.0042	-0.0617	-0.1103	0.4285	3 / 3
6609		-0.0477	-0.0045	-0.0665	0.3070	7 / 7
6610		-0.0566	-0.0122	0.0188	0.2907	4 / 4
6611		-0.0529	-0.0406	-0.0758	0.5776	8 / 8
6612		-0.0361	-0.0658	-0.0496	1.0069	7 / 7
6616		-0.0350	-0.0079	-0.0457	0.4426	5 / 5
6617		-0.0132	-0.0096	0.0025	0.5819	6 / 6
6618		-0.0097	-0.0206	-0.0200	0.4406	7 / 7
6619		0.0162	-0.0131	0.0005	0.2000	6 / 6
6620		0.0180	-0.0619	-0.0307	0.4195	6 / 6
6621		-0.0040	-0.0351	-0.0224	0.3304	8 / 8
6622		-0.0165	0.0125	0.0447	0.7543	6 / 6
6623		-0.0054	-0.0113	0.0433	0.7665	5 / 5
6624		0.0532	-0.0142	0.0067	1.1260	4 / 4
6625		-0.1177	-0.0102	0.0591	0.4805	5 / 5
6626		-0.0875	-0.0046	-0.0095	0.3335	5 / 5
6627		-0.1035	-0.0332	0.0781	0.6715	4 / 4
6628		-0.0964	-0.0319	0.0295	0.5545	6 / 6
6629		-0.1069	-0.0263	0.0424	0.3748	6 / 6
6630		-0.1182	-0.0054	0.0584	0.3368	6 / 6
6631		-0.0892	-0.0280	0.0686	0.2770	5 / 5
6632		-0.0985	-0.0220	0.0780	0.3847	5 / 5
6633		-0.0408	-0.0097	0.0581	0.3729	8 / 8
6634		-0.0597	0.0053	0.0699	0.3752	7 / 7
6635		-0.0496	0.0155	0.0800	0.3257	7 / 7
6636		-0.1036	-0.0136	0.0708	0.3018	6 / 6
6637		-0.0727	0.0086	0.0853	0.6519	5 / 5
6638		-0.0815	0.0130	0.1042	0.3433	4 / 4
6639		-0.1020	0.0240	0.0778	0.4521	6 / 6
6640		-0.0925	-0.0057	0.0633	0.3735	5 / 5
6641		-0.0572	0.0065	0.1038	0.4928	6 / 6
**Mean [m]**		-0.009685	0.000622	-0.010563		
**Sigma [m]**		0.035972	0.034174	0.054067		
**RMS Error [m]**		0.037253	0.034180	0.055089		

Localisation accuracy per GCP and mean errors in the three coordinate directions. The last column counts the number of calibrated images where the GCP has been automatically verified vs. manually marked.

Absolute Geolocation Variance

Min Error [m]	Max Error [m]	Geolocation Error X [%]	Geolocation Error Y [%]	Geolocation Error Z [%]
-	-15.00	0.00	0.00	0.00
-15.00	-12.00	0.00	0.00	0.00
-12.00	-9.00	0.00	0.00	0.00
-9.00	-6.00	0.00	0.00	0.00
-6.00	-3.00	0.00	9.42	10.87
-3.00	0.00	47.10	39.13	36.96
0.00	3.00	52.90	42.03	46.38
3.00	6.00	0.00	9.42	5.80
6.00	9.00	0.00	0.00	0.00
9.00	12.00	0.00	0.00	0.00
12.00	15.00	0.00	0.00	0.00
15.00	-	0.00	0.00	0.00

**Mean [m]**	0.892871	1.626872	32.990974
**Sigma [m]**	1.062337	2.241611	2.116960
**RMS Error [m]**	1.387724	2.769753	33.058825

Min Error and Max Error represent geolocation error intervals between -1.5 and 1.5 times the maximum accuracy of all the images. Columns X, Y, Z show the percentage of images with geolocation errors within the predefined error intervals. The geolocation error is the difference between the initial and computed image positions. Note that the image geolocation errors do not correspond to the accuracy of the observed 3D points.

Geolocation Bias	X	Y	Z
Translation [m]	0.892871	1.626872	32.990974

Bias between image initial and computed geolocation given in output coordinate system.

Relative Geolocation Variance

Relative Geolocation Error	Images X [%]	Images Y [%]	Images Z [%]
[-1.00, 1.00]	100.00	100.00	100.00
[-2.00, 2.00]	100.00	100.00	100.00
[-3.00, 3.00]	100.00	100.00	100.00
**Mean of Geolocation Accuracy [m]**	5.000000	5.000000	10.000000
**Sigma of Geolocation Accuracy [m]**	0.000000	0.000000	0.000000

Images X, Y, Z represent the percentage of images with a relative geolocation error in X, Y, Z.

Initial Processing Details

System Information

Hardware	CPU: Intel(R) Core(TM) i9-7940X CPU @ 3.10GHz<br>RAM: 128GB<br>GPU: NVIDIA GeForce GTX 1080 Ti (Driver: 26.21.14.3615), RDPDD Chained DD (Driver: unknown), RDP Encoder Mirror Driver (Driver: unknown), RDP Reflector Display Driver (Driver: unknown)
Operating System	Windows 7 Ultimate, 64-bit

Coordinate Systems

Image Coordinate System	WGS 84 (EGM96 Geoid)
Ground Control Point (GCP) Coordinate System	ETRS89 / Poland CS2000 zone 6 (EGM2008 Geoid)
Output Coordinate System	ETRS89 / Poland CS2000 zone 6 (EGM2008 Geoid)

Processing Options

Detected Template	No Template Available
Keypoints Image Scale	Full, Image Scale: 1
Advanced: Matching Image Pairs	Aerial Grid or Corridor
Advanced: Matching Strategy	Use Geometrically Verified Matching: no
Advanced: Keypoint Extraction	Targeted Number of Keypoints: Automatic
Advanced: Calibration	Calibration Method: Standard<br>Internal Parameters Optimization: All<br>External Parameters Optimization: All<br>Rematch: Auto, yes
