# Supplementary material for: Multi-Variant Accuracy Evaluation of UAV Imaging Surveys: A Case Study on Investment Area
Source: Sensors (Basel). 2019 Nov 28;19(23):5229. doi: 10.3390/s19235229 (PMC6929115; doi:10.3390/s19235229)
Supplement: Supplementary file 1 [file sensors-19-05229-s001.zip › supplementary_files/App9_V6_p65q45_noGPS_report.pdf]

Quality Report

Generated with Pix4Ddiscovery version 4.3.31

Important: Click on the different icons for:

Help to analyze the results in the Quality Report

Additional information about the sections

Click here for additional tips to analyze the Quality Report

Summary

Project	V6_p65q45_noGPS
Processed	2019-10-12 21:07:01
Camera Model Name(s)	NEX-5T_0.0_4912x3264 (RGB)
Average Ground Sampling Distance (GSD)	4.57 cm / 1.80 in
Area Covered	0.827 km^2 / 82.6740 ha / 0.32 sq. mi. / 204.3976 acres
Time for Initial Processing (without report)	24m:46s

Quality Check

**Images**	median of 45797 keypoints per image	
**Dataset**	138 out of 138 images calibrated (100%), all images enabled	
**Camera Optimization**	0.5% relative difference between initial and optimized internal camera parameters	
**Matching**	median of 15593.4 matches per calibrated image	
**Georeferencing**	yes, 11 GCPs (11 3D), mean RMS error = 0.017 m	

Preview

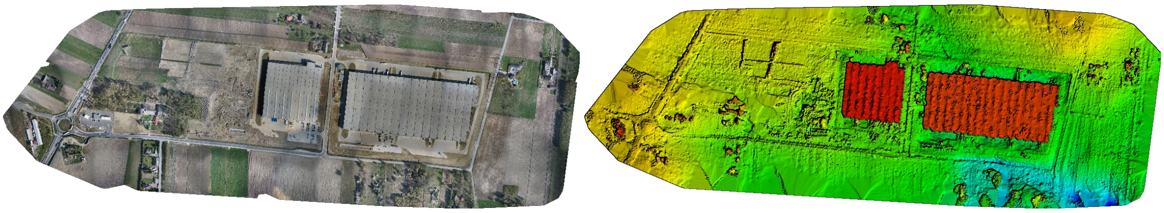

Figure 1: Orthomosaic and the corresponding sparse Digital Surface Model (DSM) before densification.

Calibration Details

Number of Calibrated Images	138 out of 138
Number of Geolocated Images	0 out of 138

Initial Image Positions

The preview is not generated for images without geolocation.

Computed Image/GCPs/Manual Tie Points Positions

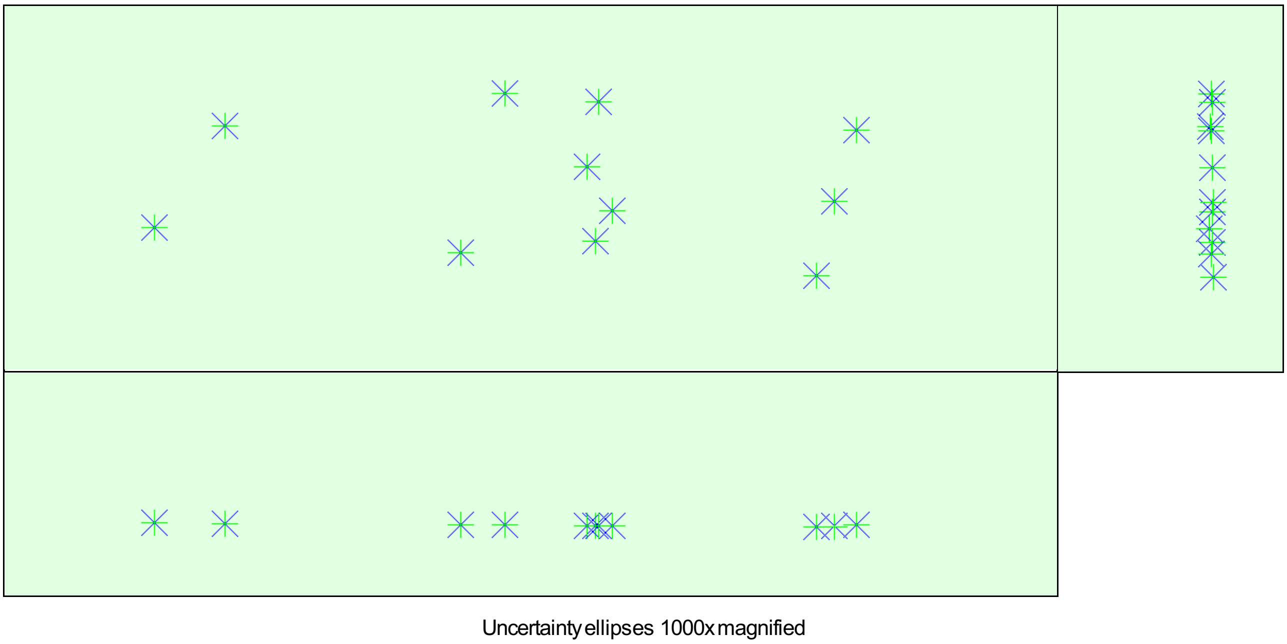

Figure 3: Offset between initial (blue dots) and computed (green dots) image positions as well as the offset between the GCPs initial positions (blue crosses) and their computed positions (green crosses) in the top-view (XY plane), front-view (XZ plane), and side-view (YZ plane). Dark green ellipses indicate the absolute position uncertainty of the bundle block adjustment result.

Absolute camera position and orientation uncertainties

	X [m]	Y [m]	Z [m]	Omega [degree]	Phi [degree]	Kappa [degree]
Mean	0.014	0.017	0.034	0.006	0.005	0.002
Sigma	0.003	0.004	0.001	0.001	0.001	0.001

Overlap

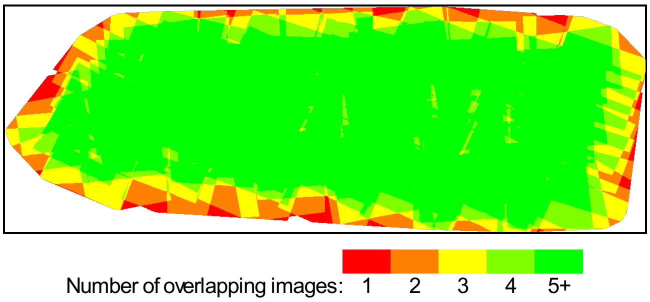

Figure 4: Number of overlapping images computed for each pixel of the orthomosaic.
Red and yellow areas indicate low overlap for which poor results may be generated. Green areas indicate an overlap of over 5 images for every pixel. Good quality results will be generated as long as the number of keypoint matches is also sufficient for these areas (see Figure 5 for keypoint matches).

Bundle Block Adjustment Details

Number of 2D Keypoint Observations for Bundle Block Adjustment	2258193
Number of 3D Points for Bundle Block Adjustment	917862
Mean Reprojection Error [pixels]	0.162

Internal Camera Parameters

NEX-5T_0.0_4912x3264 (RGB). Sensor Dimensions: 23.400 [mm] x 15.549 [mm]

EXIF ID: NEX-5T_0.0_4912x3264

	Focal Length	Principal Point x	Principal Point y	R1	R2	R3	T1	T2
Initial Values	3256.554 [pixel]<br>15.514 [mm]	2456.002 [pixel]<br>11.700 [mm]	1632.003 [pixel]<br>7.775 [mm]	-0.043	0.028	-0.005	0.000	-0.001
Optimized Values	3240.041 [pixel]<br>15.435 [mm]	2467.401 [pixel]<br>11.754 [mm]	1591.641 [pixel]<br>7.582 [mm]	-0.047	0.035	-0.010	0.000	0.000
Uncertainties (Sigma)	0.734 [pixel]<br>0.003 [mm]	0.139 [pixel]<br>0.001 [mm]	0.130 [pixel]<br>0.001 [mm]	0.000	0.000	0.000	0.000	0.000

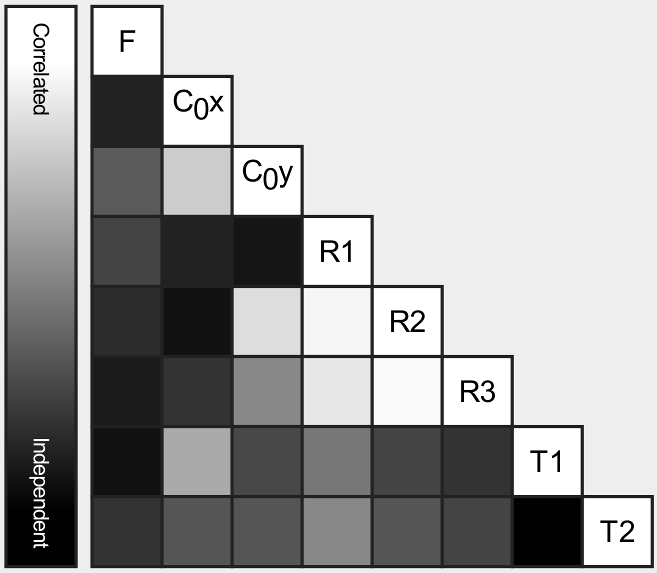

The correlation between camera internal parameters determined by the bundle adjustment. White indicates a full correlation between the parameters, ie. any change in one can be fully compensated by the other. Black indicates that the parameter is completely independent, and is not affected by other parameters.

The number of Automatic Tie Points (ATPs) per pixel, averaged over all images of the camera model, is color coded between black and white. White indicates that, on average, more than 16 ATPs have been extracted at the pixel location. Black indicates that, on average, 0 ATPs have been extracted at the pixel location. Click on the image to the see the average direction and magnitude of the re-projection error for each pixel. Note that the vectors are scaled for better visualization. The scale bar indicates the magnitude of 1 pixel error.

2D Keypoints Table

	Number of 2D Keypoints per Image	Number of Matched 2D Keypoints per Image
Median	45797	15593
Min	20057	4547
Max	68781	33756
Mean	42868	16364

3D Points from 2D Keypoint Matches

	Number of 3D Points Observed
In 2 Images	649779
In 3 Images	174468
In 4 Images	55809
In 5 Images	21703
In 6 Images	10782
In 7 Images	3999
In 8 Images	1128
In 9 Images	169
In 10 Images	25

2D Keypoint Matches

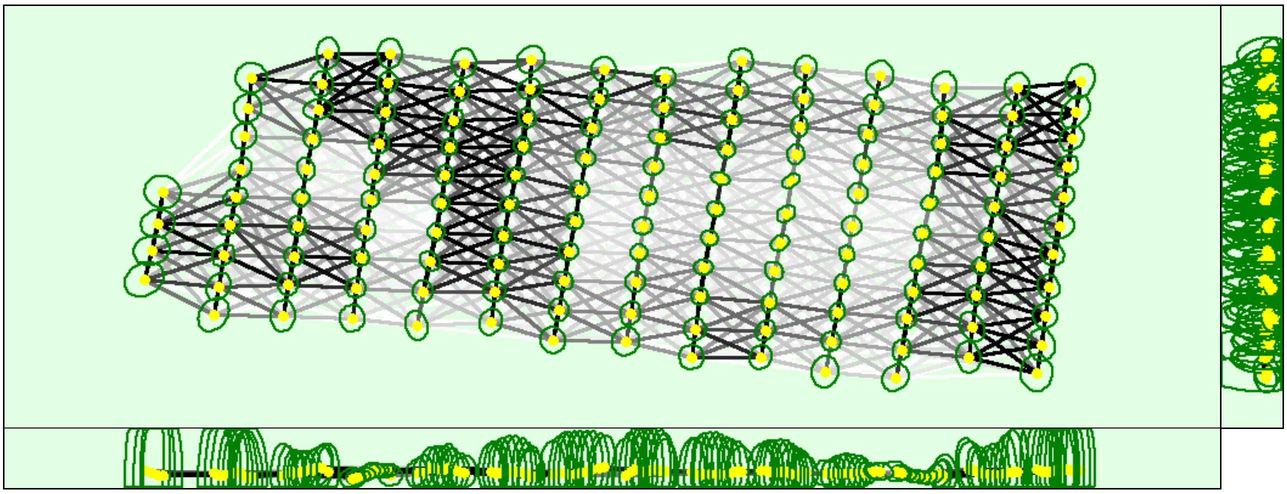

Figure 5: Computed image positions with links between matched images. The darkness of the links indicates the number of matched 2D keypoints between the images. Bright links indicate weak links and require manual tie points or more images. Dark green ellipses indicate the relative camera position uncertainty of the bundle block adjustment result.

Relative camera position and orientation uncertainties

	X [m]	Y [m]	Z [m]	Omega [degree]	Phi [degree]	Kappa [degree]
Mean	0.017	0.017	0.050	0.011	0.014	0.003
Sigma	0.003	0.004	0.029	0.005	0.007	0.001

Geolocation Details

Ground Control Points

GCP Name	Accuracy XY/Z [m]	Error X [m]	Error Y [m]	Error Z [m]	Projection Error [pixel]	Verified/Marked
201 (3D)	0.030/ 0.050	-0.000	-0.009	0.010	0.374	5 / 5
202 (3D)	0.030/ 0.050	0.001	0.005	-0.004	0.680	6 / 6
203 (3D)	0.030/ 0.050	-0.002	0.011	-0.010	0.816	7 / 7
208 (3D)	0.030/ 0.050	0.008	-0.006	-0.020	0.475	6 / 6
209 (3D)	0.030/ 0.050	0.017	-0.002	-0.006	0.431	7 / 7
207 (3D)	0.030/ 0.050	-0.028	-0.003	0.064	0.405	6 / 6
211 (3D)	0.030/ 0.050	0.010	0.018	-0.020	0.619	7 / 7
210 (3D)	0.030/ 0.050	-0.009	-0.009	0.017	0.209	6 / 6
206 (3D)	0.030/ 0.050	0.010	0.015	0.004	0.334	8 / 8
205 (3D)	0.030/ 0.050	-0.014	-0.040	-0.023	0.904	7 / 7
204 (3D)	0.030/ 0.050	-0.005	0.015	-0.008	0.863	6 / 6
**Mean [m]**		-0.000989	-0.000573	0.000409		
**Sigma [m]**		0.012277	0.015885	0.023368		
**RMS Error [m]**		0.012316	0.015896	0.023371		

0 out of 207 check points have been labeled as inaccurate.

Check Point Name	Accuracy XY/Z [m]	Error X [m]	Error Y [m]	Error Z [m]	Projection Error [pixel]	Verified/Marked
103		0.0162	0.0285	-0.0007	0.2343	6 / 6
101		-0.0092	0.0070	0.0031	0.4173	7 / 7
100		-0.0002	-0.0062	0.0608	0.2883	7 / 7
104		-0.0555	0.0035	0.0493	0.5517	6 / 6
102		-0.0173	-0.0277	0.0013	0.3731	7 / 7

k1		0.0421	-0.0633	0.1328	0.4662	6 / 6
k2		0.0041	0.0212	-0.0288	0.4935	8 / 8
k3		0.0840	-0.0916	0.1501	0.3538	6 / 6
k4		0.0291	-0.0313	0.0730	0.3881	6 / 6
k5		-0.0253	0.0261	-0.0452	0.4082	6 / 6
k6		-0.0287	0.0204	0.0676	0.5591	5 / 5
k7		-0.0965	0.0156	0.1692	0.3477	4 / 4
k8		-0.0487	-0.0326	0.1655	0.4717	5 / 5
k11		0.0013	-0.0498	-0.1153	0.6624	8 / 8
k13		-0.0088	0.0244	0.0236	0.2951	6 / 6
k14		-0.0064	-0.0032	-0.0117	0.6463	6 / 6
10000		-0.0301	-0.0223	0.0178	0.6796	6 / 6
10001		-0.0061	0.0057	0.1106	0.7431	6 / 6
10002		0.0089	-0.0224	0.0277	0.4091	5 / 5
10003		0.0034	-0.0311	-0.0082	0.5906	8 / 8
10004		-0.0001	0.0080	0.0411	0.5284	7 / 7
10005		0.0105	-0.0067	0.0323	0.7560	7 / 7
10006		-0.0080	-0.0166	0.0283	0.4356	7 / 7
10007		0.0165	-0.0107	-0.0162	0.4026	7 / 7
10008		0.0210	-0.0277	-0.0360	0.3083	8 / 8
10009		0.0103	-0.0288	-0.0303	0.4726	7 / 7
10010		0.0175	-0.0413	-0.0381	0.3840	6 / 6
10011		0.0072	-0.0473	-0.0352	0.7718	6 / 6
10012		-0.0554	0.0024	0.0260	0.4865	8 / 8
10013		-0.0306	-0.0105	0.0036	0.4281	7 / 7
10014		0.0113	0.0037	-0.0002	0.4313	7 / 7
10015		0.0190	-0.0192	0.0186	0.4679	8 / 8
10016		-0.0040	-0.0270	-0.0224	0.4427	7 / 7
10017		0.0157	-0.0409	-0.0724	0.6392	8 / 8
10018		0.0128	-0.0275	-0.0349	0.4488	3 / 3
10019		-0.0413	0.0043	0.0658	0.8300	7 / 7
10020		-0.0603	-0.0099	0.0369	0.5935	7 / 7
10021		-0.0076	-0.0077	-0.0209	0.3481	9 / 9
10022		0.0278	0.0240	-0.0751	0.3817	8 / 8
10023		0.0251	-0.0052	-0.0608	0.4076	7 / 7
10024		-0.0175	0.0017	-0.1003	0.4873	9 / 9
10025		-0.0059	0.0011	-0.0765	0.4457	8 / 8
10026		-0.0068	0.0194	-0.0596	0.4598	7 / 7
10027		-0.0037	0.0119	-0.0291	0.5415	7 / 7
10028		0.0096	0.0031	-0.0161	0.4993	7 / 7
10030		-0.0027	0.0420	-0.0329	0.4415	7 / 7
10031		0.0304	0.0338	0.0014	0.7239	4 / 4
10032		0.0430	0.0589	0.0136	0.2231	3 / 3
10033		0.0244	0.0531	0.0103	0.3238	7 / 7
10034		0.0311	0.0177	-0.0096	0.4339	7 / 7
10035		0.0246	-0.0094	0.0602	0.3733	6 / 6
10036		0.0280	0.0074	0.0240	0.2994	4 / 4
10037		-0.0258	0.0184	-0.0001	0.2824	6 / 6
10038		-0.0206	0.0292	0.0028	0.4255	8 / 8
10039		-0.0042	0.0066	-0.0061	0.4947	7 / 7
10040		-0.0143	0.0127	0.0140	0.2484	6 / 6
10041		-0.0141	-0.0019	0.0360	0.6628	8 / 8
10044		0.0043	-0.0410	0.0049	0.3435	6 / 6
10045		0.0205	-0.0277	-0.0765	0.5440	8 / 8
10046		-0.0287	-0.0204	-0.0839	0.3572	6 / 6
10047		-0.0416	-0.0108	0.1002	0.5624	6 / 6
10048		-0.0317	-0.0389	0.0918	0.7304	7 / 7
10049		-0.0165	0.0271	-0.0051	0.8547	7 / 7
10051		0.0086	0.0163	0.0101	0.4146	7 / 7

10052		0.0012	-0.0075	0.0245	0.5073	7 / 7
10053		-0.0046	-0.0057	-0.0253	0.4955	7 / 7
10054		0.0030	-0.0469	-0.0003	0.4946	7 / 7
10055		-0.0423	-0.0068	-0.0750	0.4864	6 / 6
10056		-0.0781	-0.0112	-0.0017	0.5662	7 / 7
10058		-0.1062	0.0666	0.0624	0.7346	8 / 8
10059		-0.0091	-0.0216	-0.0618	0.4231	7 / 7
10060		-0.0181	-0.0387	-0.0175	0.3966	7 / 7
10061		0.0414	-0.0092	-0.0308	0.4939	4 / 4
10062		0.0095	-0.0571	-0.0406	0.3570	9 / 9
10063		0.0324	-0.0720	-0.0739	0.5280	8 / 8
10064		0.0381	0.0387	-0.0578	0.5300	7 / 7
10065		0.0371	0.0574	-0.0333	0.2916	6 / 6
10066		0.0685	0.0627	-0.0705	0.5235	6 / 6
10067		0.0330	0.0616	-0.0727	0.3740	5 / 5
10068		0.0210	0.1317	-0.0608	0.2254	6 / 6
10074		-0.0065	0.0722	-0.0475	0.5601	7 / 7
10075		-0.0008	0.0734	0.0082	0.4138	7 / 7
10076		0.0094	0.0552	-0.0505	0.5232	6 / 6
10077		-0.0254	0.0963	-0.0605	0.6672	6 / 6
10078		-0.0086	0.0514	-0.0292	0.1975	6 / 6
10080		0.0138	-0.0586	-0.1341	0.9652	7 / 7
10081		-0.0337	-0.0324	-0.0247	0.4701	8 / 8
10082		0.0037	-0.0386	-0.0622	0.5248	9 / 9
10083		0.0533	-0.0103	-0.1070	0.3446	6 / 6
10085		0.0506	-0.0588	-0.0176	0.3988	8 / 8
15000		-0.0045	-0.0348	-0.0041	0.3472	7 / 7
15001		-0.0267	-0.0378	-0.0202	0.3653	6 / 6
15002		-0.0190	0.0508	-0.0951	0.3186	6 / 6
15003		-0.0311	0.0669	-0.0304	0.3458	9 / 9
15004		-0.0254	0.0294	-0.0554	0.2486	7 / 7
15005		-0.0204	0.0472	-0.0615	0.9022	6 / 6
15006		0.0024	0.0393	-0.0375	0.3448	7 / 7
15007		0.0121	-0.0194	-0.0154	0.3625	5 / 5
15008		-0.0362	0.0138	0.0901	1.0961	6 / 6
15009		-0.0128	-0.0028	0.0144	0.6714	6 / 6
15010		0.0072	0.0477	0.0492	0.6980	6 / 6
20000		-0.0010	0.0249	0.0350	0.8125	6 / 6
20001		-0.0102	-0.0044	0.0478	0.3728	6 / 6
20002		-0.0024	0.0098	0.0281	0.4319	6 / 6
20003		-0.0314	-0.0102	0.0033	0.5844	7 / 7
20004		-0.0080	-0.0141	0.0417	0.4754	6 / 6
20005		-0.0648	-0.0367	0.0395	0.3297	6 / 6
20008		0.0031	-0.0157	-0.0074	0.5581	8 / 8
20009		0.0303	-0.0369	-0.0506	0.4151	8 / 8
20010		-0.0352	0.0170	-0.0836	0.5098	9 / 9
20011		-0.0216	-0.0062	-0.0718	0.3997	10 / 10
20012		0.0142	-0.0077	-0.0815	0.3701	8 / 8
20013		0.0212	-0.0099	-0.0338	0.3764	7 / 7
20014		0.0173	0.0027	-0.0255	0.3194	7 / 7
20016		-0.0107	0.0064	-0.1309	0.2416	8 / 8
20017		-0.0028	0.0221	-0.1007	0.4078	6 / 6
20018		0.0217	0.0231	-0.0747	0.3542	7 / 7
20019		0.0313	0.0190	-0.0457	0.2922	7 / 7
20020		0.0168	0.0494	-0.0043	0.5699	7 / 7
20021		0.0152	0.0211	0.0031	0.1576	6 / 6
20022		-0.0121	-0.0263	0.0116	0.2179	6 / 6
20023		-0.0020	0.0268	-0.0138	0.1895	6 / 6
20024		0.0051	0.0721	-0.0397	0.4405	7 / 7

20025		0.0249	0.0752	-0.0354	0.2912	7 / 7
20026		0.0230	0.0715	-0.0122	0.4952	8 / 8
20027		-0.0374	0.0967	-0.0299	0.4821	5 / 5
20028		-0.0256	0.0372	-0.0171	0.3775	6 / 6
20029		0.0072	-0.0123	-0.0549	0.9065	8 / 8
20031		-0.0461	0.0093	-0.0542	0.5114	6 / 6
25000		0.0054	0.0257	-0.0241	0.3416	8 / 8
25001		0.0014	0.0027	0.0023	0.2052	5 / 5
25003		0.0069	-0.0133	0.0429	0.4585	7 / 7
25004		0.0091	0.0135	0.0145	0.4260	6 / 6
25005		0.0075	-0.0036	0.0520	0.2976	6 / 6
25006		-0.0272	-0.0289	-0.0763	0.5325	7 / 7
25008		-0.0256	0.0046	-0.0257	0.1577	6 / 6
25009		0.0316	-0.0441	-0.0263	0.3265	7 / 7
25010		0.0037	-0.0144	-0.0373	0.2788	7 / 7
25013		-0.0081	0.0133	-0.0284	0.3150	6 / 6
25014		-0.0250	-0.0182	-0.0380	0.3002	6 / 6
25015		-0.0397	-0.0189	-0.0331	0.3490	6 / 6
25016		-0.0335	-0.0181	-0.0387	0.3005	6 / 6
25017		0.0292	-0.0381	0.0441	0.3853	6 / 6
25018		0.0070	-0.0108	0.0460	0.3417	7 / 7
25019		-0.0136	-0.0276	0.0340	0.3161	6 / 6
25020		-0.0145	-0.0093	-0.0387	0.1807	4 / 4
25021		0.0005	0.0099	0.0213	0.3133	6 / 6
25022		-0.0078	0.0057	0.0496	0.5023	6 / 6
25023		-0.0037	0.0222	-0.0122	0.2371	6 / 6
25024		0.0092	-0.0053	-0.0422	0.2605	6 / 6
25029		0.0266	-0.0318	-0.0547	0.4297	8 / 8
25030		0.0012	-0.0323	0.0156	0.3800	6 / 6
25032		-0.0550	-0.0315	-0.0156	0.5096	6 / 6
25033		-0.0106	-0.0410	-0.0144	0.3599	8 / 8
25034		-0.0036	-0.0341	0.0036	0.3938	7 / 7
40000		-0.0183	0.0035	-0.0820	0.3117	6 / 6
40001		0.0107	0.0533	-0.0150	0.3616	8 / 8
40002		0.0303	0.0717	-0.0618	0.4088	6 / 6
40003		0.0207	0.0776	-0.0297	0.2063	5 / 5
40004		0.0194	0.0298	-0.0208	0.3026	6 / 6
40005		0.0248	0.0507	-0.0440	0.2312	3 / 3
45000		0.0098	-0.0366	0.0168	0.4130	7 / 7
45001		-0.0214	0.0118	0.0102	0.1790	4 / 4
45002		0.0374	-0.0564	0.0059	0.6775	6 / 6
10043		-0.0159	0.0021	0.0417	0.9440	6 / 6
10084		-0.0242	-0.0042	-0.0253	0.5272	4 / 4
20015		-0.0237	-0.0275	-0.1189	0.2263	6 / 6
20007		-0.0061	-0.0026	-0.0333	0.3332	4 / 4
25027		-0.0401	-0.0243	-0.0807	0.3476	7 / 7
k9		0.0095	-0.0289	-0.0388	0.5844	7 / 7
6600		0.0097	0.0173	-0.0900	0.3727	6 / 6
6602		0.0098	0.0381	-0.0297	0.4469	7 / 7
6603		-0.0063	-0.0019	-0.0145	0.3378	6 / 6
6605		0.0138	0.0201	-0.0011	0.5562	6 / 6
6606		0.0044	0.0087	-0.0196	0.5863	4 / 4
6607		0.0233	0.0175	-0.0619	0.2878	6 / 6
6608		-0.0046	-0.0639	-0.1122	0.4153	3 / 3
6609		-0.0475	-0.0039	-0.0713	0.3348	7 / 7
6610		-0.0562	-0.0113	0.0148	0.3016	4 / 4
6611		-0.0509	-0.0384	-0.0716	0.6042	8 / 8
6612		-0.0337	-0.0634	-0.0464	1.0381	7 / 7
6616		-0.0355	-0.0114	-0.0156	0.4502	5 / 5

6617		-0.0133	-0.0119	0.0100	0.5892	6 / 6
6618		-0.0101	-0.0225	-0.0123	0.4354	7 / 7
6619		0.0161	-0.0156	0.0041	0.2043	6 / 6
6620		0.0186	-0.0648	-0.0307	0.4304	6 / 6
6621		0.0009	-0.0371	-0.0293	0.3348	8 / 8
6622		-0.0154	0.0189	0.0380	0.7761	6 / 6
6623		-0.0014	-0.0047	0.0311	0.8346	5 / 5
6624		0.0555	-0.0070	0.0044	1.1828	4 / 4
6625		-0.1199	-0.0065	0.0735	0.4778	5 / 5
6626		-0.0898	-0.0001	0.0044	0.3161	5 / 5
6627		-0.1065	-0.0294	0.0915	0.6795	4 / 4
6628		-0.0994	-0.0285	0.0429	0.5547	6 / 6
6629		-0.1101	-0.0231	0.0556	0.3714	6 / 6
6630		-0.1218	-0.0023	0.0714	0.3339	6 / 6
6631		-0.0932	-0.0251	0.0818	0.2915	5 / 5
6632		-0.1039	-0.0201	0.0918	0.4127	5 / 5
6633		-0.0445	-0.0082	0.0694	0.3746	8 / 8
6634		-0.0632	0.0073	0.0811	0.3610	7 / 7
6635		-0.0527	0.0178	0.0915	0.3406	7 / 7
6636		-0.1050	-0.0106	0.0797	0.3040	6 / 6
6637		-0.0741	0.0117	0.0943	0.6393	5 / 5
6638		-0.0822	0.0161	0.1103	0.3541	4 / 4
6639		-0.1029	0.0271	0.0860	0.4572	6 / 6
6640		-0.0930	-0.0028	0.0719	0.4003	5 / 5
6641		-0.0583	0.0089	0.1110	0.4944	6 / 6
**Mean [m]**		-0.010027	0.000407	-0.006323		
**Sigma [m]**		0.036257	0.034761	0.055813		
**RMS Error [m]**		0.037618	0.034763	0.056170		

Localisation accuracy per GCP and mean errors in the three coordinate directions. The last column counts the number of calibrated images where the GCP has been automatically verified vs. manually marked.

Initial Processing Details

System Information

Hardware	CPU: Intel(R) Core(TM) i9-7940X CPU @ 3.10GHz<br>RAM: 128GB<br>GPU: NVIDIA GeForce GTX 1080 Ti (Driver: 26.21.14.3615), RDPDD Chained DD (Driver: unknown), RDP Encoder Mirror Driver (Driver: unknown), RDP Reflector Display Driver (Driver: unknown)
Operating System	Windows 7 Ultimate, 64-bit

Coordinate Systems

Ground Control Point (GCP) Coordinate System	ETRS89 / Poland CS2000 zone 6 (EGM 2008 Geoid)
Output Coordinate System	ETRS89 / Poland CS2000 zone 6 (EGM 2008 Geoid)

Processing Options

Detected Template	No Template Available
Keypoints Image Scale	Full, Image Scale: 1
Advanced: Matching Image Pairs	Aerial Grid or Corridor
Advanced: Matching Strategy	Use Geometrically Verified Matching: no
Advanced: Keypoint Extraction	Targeted Number of Keypoints: Automatic

Advanced: Calibration	Calibration Method: Standard<br>Internal Parameters Optimization: All<br>External Parameters Optimization: All<br>Rematch: Auto, yes
